# Lactone-to-Lactam Editing Alters the Pharmacology of Bilobalide

**DOI:** 10.1021/jacsau.4c00416

**Published:** 2024-07-16

**Authors:** Xiaoding Jiang, Xu He, Jonathan Wong, Stephan Scheeff, Sam Chun-Kit Hau, Tak Hin Wong, Yao Qin, Chi Hang Fan, Bowen Ma, Ngai Lam Chung, Junzhe Huang, Jiajia Zhao, Yu Yan, Min Xiao, Xueqin Song, Tony K. C. Hui, Zhong Zuo, William Ka-Kei Wu, Ho Ko, Kim Hei-Man Chow, Billy Wai-Lung Ng

**Affiliations:** † School of Pharmacy, Faculty of Medicine, The Chinese University of Hong Kong, Shatin, New Territories, Hong Kong SAR, China; ‡ Department of Chemistry, Faculty of Science, The Chinese University of Hong Kong, Hong Kong SAR, China; § Department of Biochemistry, University of Oxford, Oxford OX1 3QU, United Kingdom; ∥ Division of Neurology, Department of Medicine and Therapeutics, Margaret K.L. Cheung Research Centre for Management of Parkinsonism, Faculty of Medicine, The Chinese University of Hong Kong, Shatin, New Territories, Hong Kong SAR, China; ⊥ Primemax Biotech Ltd., Wayson Commercial House, 68-70 Lockhard Road, Wan Chai, Hong Kong SAR, China; # Department of Anaesthesia and Intensive Care and Peter Hung Pain Research Institute, The Chinese University of Hong Kong, Shatin, New Territories, Hong Kong SAR, China; ∇ Li Ka Shing Institute of Health Sciences, Faculty of Medicine, The Chinese University of Hong Kong, Shatin, New Territories, Hong Kong SAR, China; ○ Peter Hung Pain Research Institute, Faculty of Medicine, The Chinese University of Hong Kong, Shatin, New Territories, Hong Kong SAR, China; ◆ School of Life Sciences, Faculty of Science, The Chinese University of Hong Kong, Shatin, New Territories, Hong Kong SAR, China; ( Gerald Choa Neuroscience Institute, The Chinese University of Hong Kong, Hong Kong SAR, China

**Keywords:** bilobalide, pharmacology, molecular editing, natural product, drug discovery

## Abstract

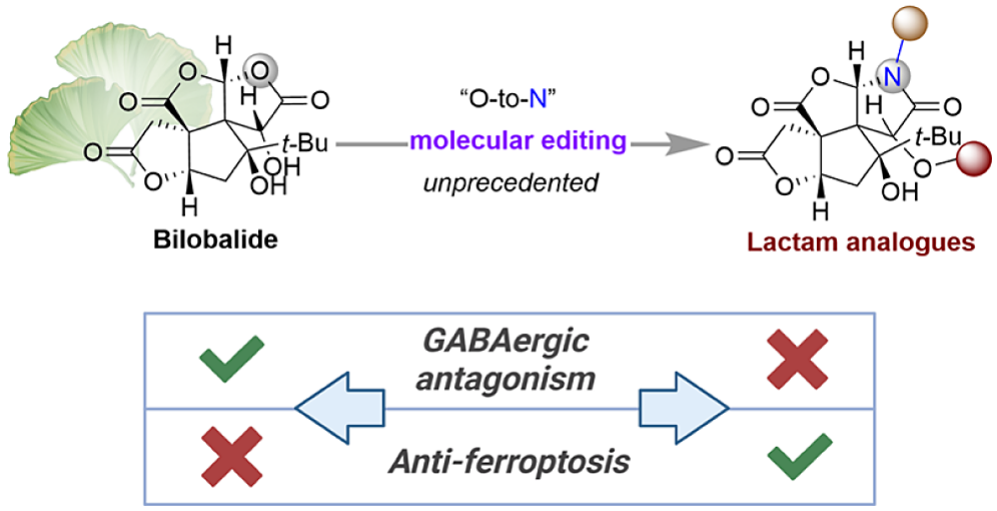

Precise transformations of natural products (NPs) can fine-tune their physicochemical properties while preserving inherently complex and evolutionarily optimized parent scaffolds. Here, we report an unprecedented lactone-to-lactam transformation on bilobalide, thus improving its stability and paving the way for biological exploration of previously inaccessible chemical space that is highly representative of the parent structure. This late-stage molecular editing of bilobalide enables facile access to a unique library of lactam analogues with altered pharmacology. Through phenotypic screening, we identify **BB10** as a hit compound with unexpected inhibition of ferroptotic cell death. We further reveal that **BB10** suppresses ferroptosis by restoring the expression of glutathione peroxidase 4 (GPX4) in brain cells. This study highlights that even subtle changes on NP scaffolds can confer new pharmacological properties, inspiring the exploration of simple yet critical transformations on complex NPs.

## Introduction

Natural products (NPs) are frequently endowed with bioactivities through evolution.^1^ The structural complexity and diversity of NPs make them invaluable and attractive reservoirs of inspiration for drug discovery.^2^ Although there is a seemingly inexhaustible abundance of NPs in nature, there are bottlenecks associated with their isolation and structural identification. For rapid accessibility to value-added NP-inspired compound libraries, several complementary strategies have been proposed, such as diversity-oriented synthesis (DOS),^3^ biology-oriented synthesis (BIOS),^4,5^ complexity-to-diversity approaches (CtD),^6,7^ and the pseudonatural product (PNP) principle.^8−11^ Certainly, these strategies have substantially expanded our reach into unprecedented biologically relevant chemical spaces that nature and existing biosynthetic pathways do not cover.^12^ However, the development of precise and accessible transformations for manipulating the core skeletal structure is still limited. Recently, molecular editing has emerged as an appealing strategy for late-stage modification of complex ring systems and possesses immense potential to facilitate the exploration of novel chemical spaces without laborious *de novo* synthetic sequences.^13−18^

*Ginkgo biloba* L., often known as a “living fossil”,^19,20^ is one of the oldest living species and has a long history of use in traditional Chinese medicine. The terpene trilactones (TTLs), namely, ginkgolides and bilobalide, are polyoxygenated diterpenoids, and the most structurally unique constituents extracted from the *Ginkgo biloba* L.^21,22^ Thereinto, bilobalide was first isolated from the leaves of *Ginkgo biloba* L. in 1967^19^ and is the most abundant TTL in the EGb 761 extract.^23^ It has good oral bioavailability^24^ and exhibits a broad range of pharmacological effects, mainly including neuroprotective,^25−27^ anti-inflammatory,^28^ anticonvulsant,^29^ and antiapoptotic^30^ effects. Mechanistically, bilobalide is postulated to act as an antagonist of gamma-aminobutyric acid A receptors (GABA_A_Rs).^31−33^ Although the underlying molecular mechanism remains largely unsubstantiated, the high information density^34^ and versatile properties^35^ of bilobalide render it an extremely attractive starting point for drug discovery.

**Figure 1:**
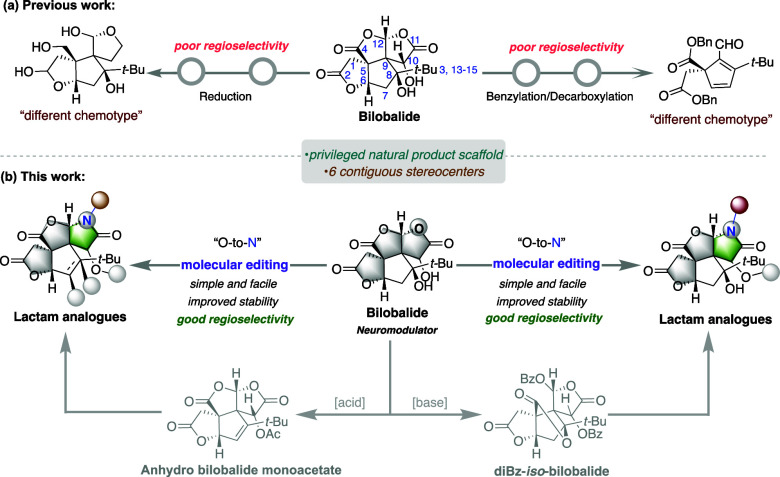
(a) Conceptual overview of previous approaches to access the derivatives of bilobalide. (b) Molecular editing as a strategy for late-stage modification of bilobalide in our research work.

Due to the high fraction of *sp*^3^-hybridized centers and abundant stereogenicity, bilobalide has attracted intense interest as targets for total synthesis, with significant achievements made in the past decades.^21,36−38^ By contrast, the late-stage modification of bilobalide is formidably challenging because of the steric congestion and intrinsic instability^39^ of the highly strained lactone rings. To date, only a handful of functionalizations on bilobalide have been disclosed, largely limited to the rapid translactonization^40,41^ into *iso*-bilobalide simply upon treatment with base.^34,38^ To the best of our knowledge, however, there has been no report of regioselective modification of any of the three lactone rings. Previous attempts to synthesize stable analogues of bilobalide were typically inefficient and harsh, thus leading to completely different chemotypes (Figure 1a) and making it difficult to exploit the privileged NP scaffold of bilobalide.^41,42^ As such, it becomes imperative to devise a handy strategy to efficiently broaden the chemical space of bilobalide by leveraging its inherent complexity for drug discovery initiatives.

Herein, we report the facile and regioselective conversion of one of the lactone rings of bilobalide into a lactam to furnish lactam-type derivatives with improved chemical stability (Figure 1b). These subtle tweaks also grant new opportunities to explore the biologically relevant chemical space of bilobalide. Further biological investigation of the analogues identified a hit compound effective against ferroptosis, a nonapoptotic form of cell death characterized by lipid peroxidation and implicated in neurodegenerative diseases.^43^

## Results and Discussion

### Pilot Molecular Editing of Bilobalide

**Scheme 1:**
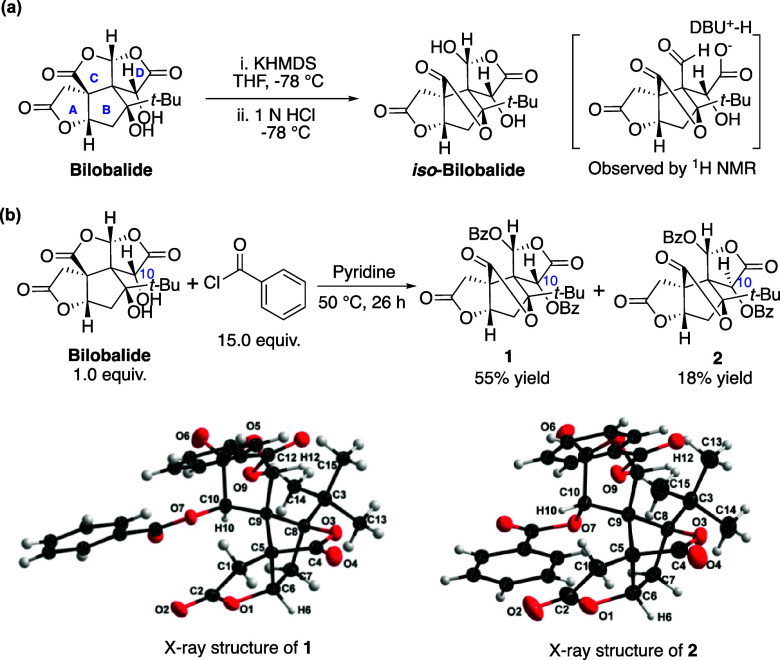
(a) Skeleton Rearrangement to Form *iso***-**Bilobalide^34,38^ and (b) Benzoylation of Bilobalide to Afford diBz-*iso*-Bilobalides

Bilobalide has been a highly challenging target for chemical modification, as it irreversibly decomposed through lactone opening^39,44^ and its instability might stem from the rapid skeleton rearrangement to *iso*-bilobalide.^34,38^ For example, the Shenvi group found that intramolecular translactonization of bilobalide occurred upon treatment with potassium bis(trimethylsilyl)amide (KHMDS) followed by subsequent acidfication, as evidenced by ^1^H NMR in the presence of 1,8-diazabicyclo[5.4.0] undec-7-ene (DBU) (Scheme 1a).^34,38^

To expand into a new chemical space for bilobalide while preserving its evolutionarily optimized structural complexity, we began by identifying scaffold(s) that could enhance the chemical stability of bilobalide. We hypothesized that the irreversible lactone opening might provide a valuable opportunity for functionalizations of bilobalide, such as amide formation and subsequent lactamization, thereby stabilizing its core skeleton. However, initial attempts at direct lactamization of bilobalide with aniline or benzylamine were unsuccessful. We then explored dibenzoylated *iso*-bilobalide (diBz-*iso*-bilobalide) **1** and **2** for further transformations. Through a modified procedure,^40,41^ we obtained two epimeric diBz-*iso*-bilobalides **1** and **2** as superb intermediates as potential synthetic intermediates (Scheme 1b). The epimerization at the C10 position may occur *via* keto–enol tautomerization and proton exchanges.^34^ The structures of epimeric intermediates **1** and **2** were unambiguously confirmed by X-ray crystallography analysis (Figures S1 and S2).

**Scheme 2:**
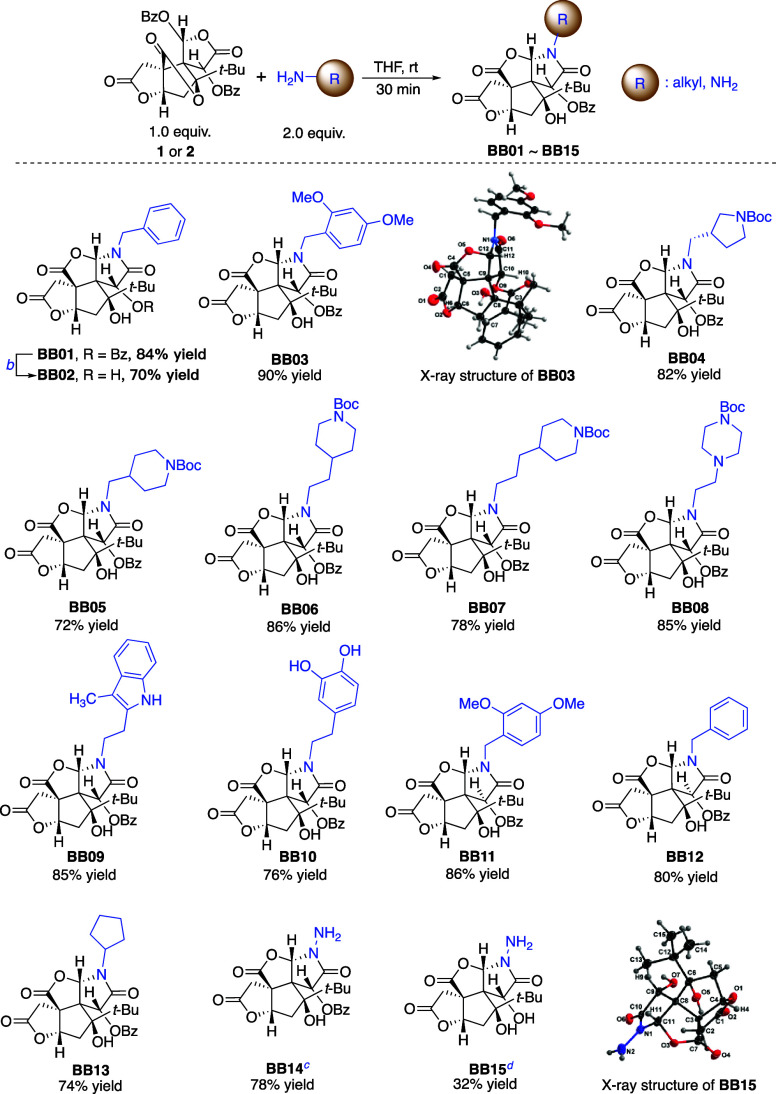
Regioselective Lactamization of 1/2 with Alkylated Amines and Dinucleophile Hydrazine Hydrate Yields denote isolated yields. K_2_CO_3_, MeOH, rt. Reaction was conducted at −10 °C. 5.0 equiv of hydrazine hydrate was used.

Although the synthesis of diBz-*iso*-bilobalides **1** and **2** has been reported, their reactivity profile remains unexplored. While the nucleophilic attack of aniline with intermediate **1** failed, stronger nucleophiles (such as benzylamine or 2,4-dimethoxybenzylamine) unexpectedly triggered a cascade reaction to give the unprecedented bilobalide analogues **BB01** and **BB03**, each featuring a lactam ring (Scheme 2). This represents a facile molecular editing approach that harnesses the unusual reactivity of these intermediates. Driven by this unique reactivity, we explored the scope of the reaction using substituted alkylamines (Scheme 2), aiming to construct a diverse library of novel bilobalide analogues. Under mild conditions, various alkylamines reacted regioselectively and smoothly with intermediate **1** to give a collection of lactam products, which included those appended with pyrrolidine (**BB04**), piperidines (**BB05**–**BB07**), piperazine (**BB08**), indole (**BB09**), and 3,4-dihydroxyphenyl (**BB10**), highlighting the utility of this molecular editing approach in broadening the chemical and potentially biological space of bilobalide. Additionally, the *tert*-butoxycarbonyl (Boc) protecting group in the products would provide a handle for further functionalizations. Besides, as exemplified by **BB02**, the resulting products are also amenable to debenzoylation with potassium carbonate as the base in methanol.

Similarly, intermediate **2** also served as an effective substrate to furnish **BB11** and **BB12** with the (*S*) configuration at C10, allowing for convenient access to the related epimers. Nucleophiles with α-branched amine like cyclopentanamine are also compatible with this regioselective transformation, affording the corresponding analogue **BB13**. In an attempt to construct analogues with ring expansion, the dinucleophile hydrazine hydrate was also utilized as a substrate; however, instead of an expanded 6-membered ring, the reaction exclusively yielded the 5-membered ring product **BB14**, which could be further debenzoylated in the excess of hydrazine hydrate to yield **BB15**. The X-ray crystallographic analysis of **BB03** and **BB15** confirmed the retention of configuration at all stereochemical centers and also the unique regioselectivity of the reactions (Figures S3 and S4).

### Proposed Reaction Mechanism of the Nontrivial Lactamization Cascade

**Figure 2:**
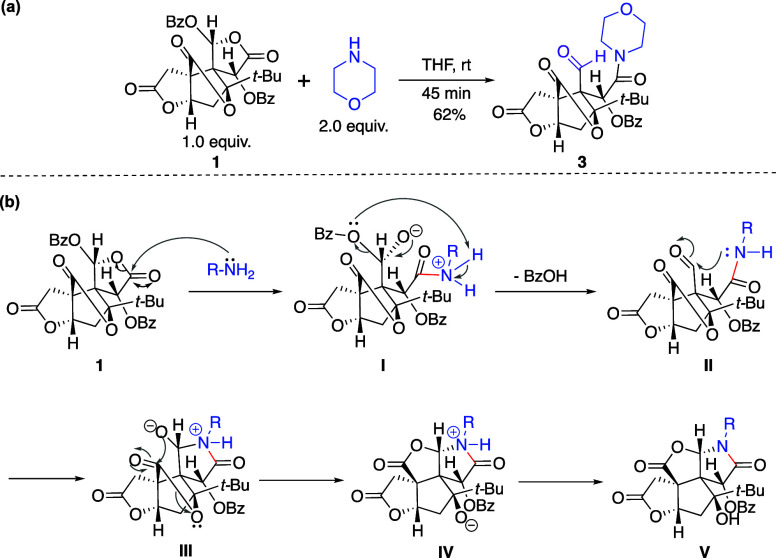
(a) Control experiment to trap the intermediate. (b) Proposed mechanism for the nontrivial lactamization cascade.

To rationalize the mechanism of this transformation, we reacted intermediate **1** with the secondary amine morpholine to trap the reaction intermediate (Figure 2a). The isolation of an aldehyde intermediate **3** supports a possible reaction mechanism, as depicted in Figure 2b. First, the nucleophilic ring opening of lactone D gives amide **I**. This regioselectivity is presumably due to the presence of an electron-withdrawing benzoate group at C10, which renders the C11 position of lactone D more electrophilic. Subsequent elimination of the benzoate group in **I** yields the key aldehyde intermediate **II**, which undergoes intramolecular nucleophilic addition by the adjacent amide to give lactam **III**. Finally, translactonization reconstitutes the cyclized scaffold **IV**, which affords **V***via* proton transfer.

### Comparison of Chemical Stability of Bilobalide and Its Lactam Analogue

**Figure 3:**
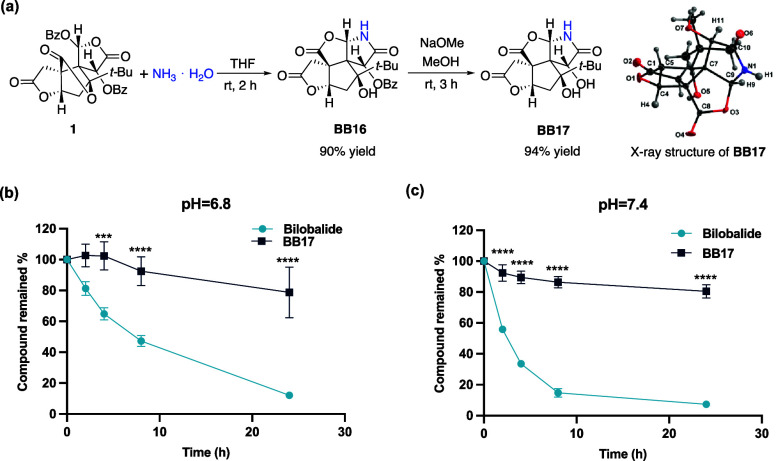
(a) Synthesis of lactam analouge **BB17**. (b) Comparison of the relative stability between bilobalide and **BB17** in an aqueous medium. Compound remained % indicated the ratio of the compound remained at a certain time point relative to *t* = 0. Data are plotted as mean ± s.d., *n* = 3 independent experiments. Statistical analyses were performed by two-way ANOVA with multiple comparisons using Bonferroni’s multiple comparisons test. ***p < 0.001 and ****p < 0.0001.

We used ammonia solution as the nucleophile in the lactamization cascade to generate **BB16**, which was then hydrolyzed to give the debenzoylated lactam analogue **BB17** (Figures 3a and S5). To compare the relative chemical stabilities of bilobalide and its lactam counterpart **BB17**, we monitored their hydrolytic profiles in buffer solutions with physiologically relevant pH values (pH = 7.4 or 6.8)^45^ using LC-MS/MS over 24 h. In line with our hypothesis that lactamized analogues could stabilize the core skeleton of bilobalide, **BB17** exhibited a much higher hydrolytic stability with respect to its parental compound bilobalide (Figure 3b). Detailed X-ray crystallographic analysis further indicated that the lactamization of the D-ring lactone likely contributes to stabilizing the C-ring lactone in bilobalide (refer to Table S1 for details). This enhanced stability of lactam analogues may therefore facilitate further chemical modifications, allowing the development of potential drug candidates and the elucidation of their mechanisms of action (MOA).

### Construction of *N*-Arylated Bilobalide Analogues

**Scheme 3:**
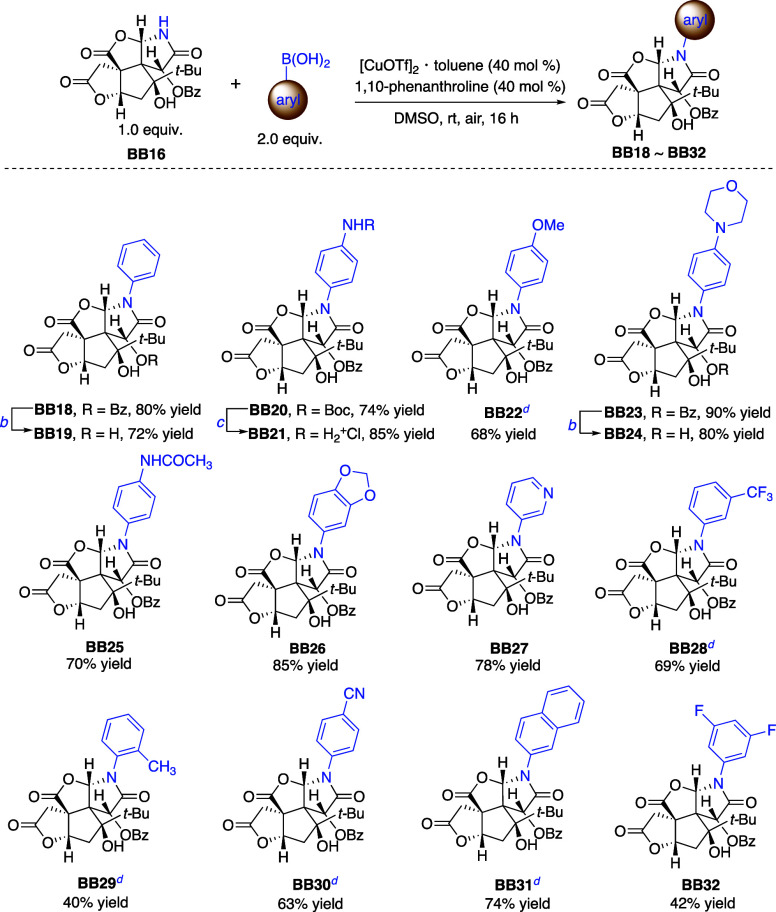
Chan–Lam Coupling Reactions of BB16 with Aryl and Heteroaryl Boronic Acids Yields denote isolated yields. K_2_CO_3_, MeOH, rt. Acetyl chloride, MeOH, rt. Reactions were conducted using [CuOTf]_2_·toluene (20 mol %), and 1,10-phenanthroline was not used.

As aniline failed to act as a substrate in the lactamization cascade, we utilized the copper-promoted Chan–Evans–Lam coupling^46^ of **BB16** to synthesize the *N*-arylated lactam analogues. A range of aryl boronic acids with various electronic properties could be incorporated, forming the desired *N*-arylated analogues **BB18**–**BB32** (Scheme 3). Basic debenzoylation of the resulting analogues such as **BB18** and **BB23** furnished the lactams **BB19** and **BB24**, respectively, with an additional free hydroxyl group. Both electron-rich and electron-deficient aryl boronic acids are utilized to deliver the corresponding products, including those containing NHBoc (**BB20**), methoxy (**BB22**), morpholinyl (**BB23**), and NHCOCH_3_ substituents (**BB25**). Mild removal of Boc in **BB21** with acetyl chloride–methanol gave **BB21** as a salt in an 85% yield. Aryl boronic acid substrates bearing substituents at the *ortho*- (**BB29**), *meta*- (**BB28**), *para*-positions (**BB22** and **BB30**) and disubstitution (**BB32**) are all compatible with the coupling reactions to produce the corresponding analogues, albeit *ortho*-substituent (as in **BB29**) gave a lower yield. The reaction with fused arenes proved to be effective, as demonstrated by the successful synthesis of coupled products **BB26** and **BB31**, both of which achieved good yields. Heterocyclic systems like 3-pyridyl are also reliably engaged in this transformation, resulting in the functionalized product **BB27**. The broad scope and versatility of this reaction underscore its potential to diversify bilobalide, facilitating the exploration of the yet-unknown biological spaces of its biomolecular targets.

### Lactamization of Acyl Derivatives of Bilobalide

**Scheme 4:**
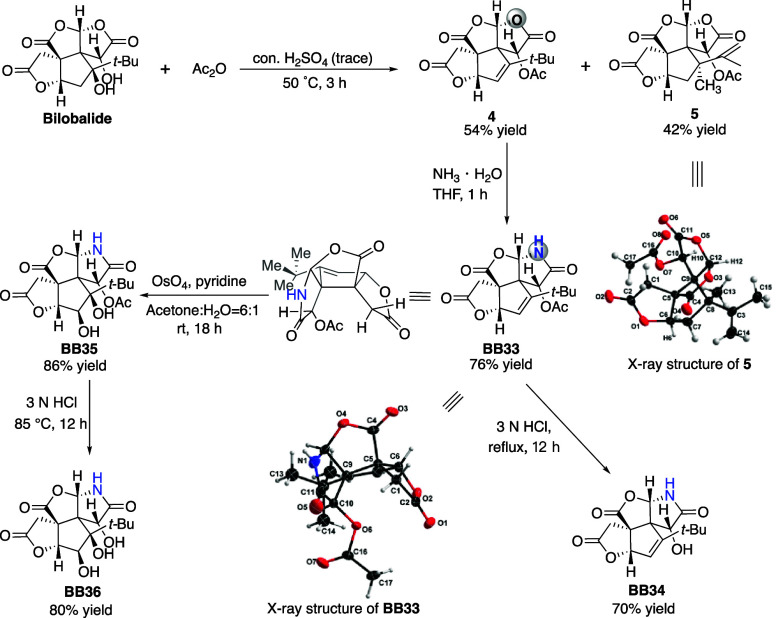
Investigation of the Reactivity of Acyl Derivatives of Bilobalide

Acetylation and dehydration of bilobalide could generate an alternative scaffold for lactamization. To this end, bilobalide was heated in acetic anhydride with a catalytic amount of concentrated sulfuric acid,^40^ resulting in anhydro bilobalide monoacetate **4**, which is devoid of the tertiary hydroxyl group at C8 (Scheme 4). Notably, we also observed the formation of a previously unknown side product **5**, resulting from a Wagner–Meerwein rearrangement followed by an E1 elimination reaction. The structure of the rearranged product **5** was conclusively determined using X-ray crystallographic analysis (Figure S6). Intriguingly, ammonium hydroxide attacked the reactive intermediate **4** with unexpected regioselectivity, transforming the D-ring lactone directly into a lactam and efficiently producing the molecular edited analogue **BB33**, the absolute configuration of which was also determined by X-ray crystallographic analysis (Figure S7; see Figure S8 for the proposed mechanism). Under acidic conditions, **BB33** could be easily deacetylated to give **BB34**. Further late-stage dihydroxylation of the olefin **BB33** with osmium tetroxide and pyridine afforded diol **BB35**, of which the stereochemistry was assigned by analogy with a reported lactone analogue.^36^ Subsequent acid hydrolysis of the acetate group of **BB35** generated the triol **BB36**. These scaffolds would allow for future peripheral editing to enrich the structural diversity of our bilobalide libraries.

### Evaluation of the GABAergic Antagonism Effect

**Figure 4:**
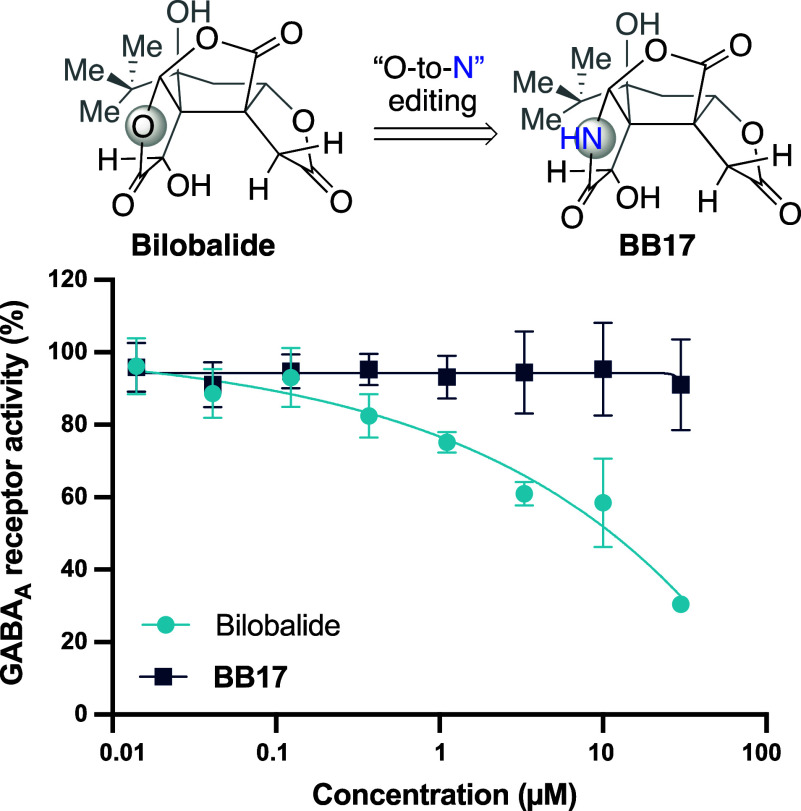
Antagonistic effect of bilobalide and **BB17** on GABA_A_ receptors. Receptors were activated with GABA (5.2 μM) in the presence of bilobalide or **BB17** (0–30 μM). Inhibition of GABA_A_ receptors was evaluated using membrane potential dye. Data are plotted as mean ± SEM, *n* = 4 technical replicates.

Since the GABAergic antagonism effect of bilobalide has been well-established,^31^ we first tested the antagonistic effect of the bilobalide analogue **BB17** on the GABA_A_ receptor. To our surprise, the lactam analogue **BB17** showed no antagonistic effect on the GABAergic receptor, while bilobalide exhibited GABAergic antagonism with an IC_50_ of 9.96 μM (Figure 4). This observation suggests that the pharmacological profile of bilobalide has been significantly altered by subtly modifying one of its lactone rings. This finding prompted a comprehensive evaluation using phenotypic screening assays pertinent to neurodegenerative conditions, aiming to uncover the pharmacological profile of these unprecedented bilobalide analogues.

### Antiferroptosis Phenotypic Screening and Assessments

Ferroptosis, a form of cell death driven by iron-dependent lipid peroxidation, has been associated with various neurodegenerative diseases^47^ such as Parkinson’s Disease^48^ or Alzheimer’s Disease.^49^ It was well documented that ferroptosis inhibitors significantly protected against neurodegeneration in preclinical models, highlighting the therapeutic potential of targeting ferroptosis.^50,51^ Besides, phenotypic screening is emerging as a powerful means in neuroprotective drug development, which has identified promising compounds advancing toward clinical trials.^52^ Given the capability of bilobalide to cross the blood–brain barrier,^53^ we investigated whether our bilobalide analogues could serve as effective ferroptosis inhibitors in cellular models of neurodegeneration.

**Figure 5:**
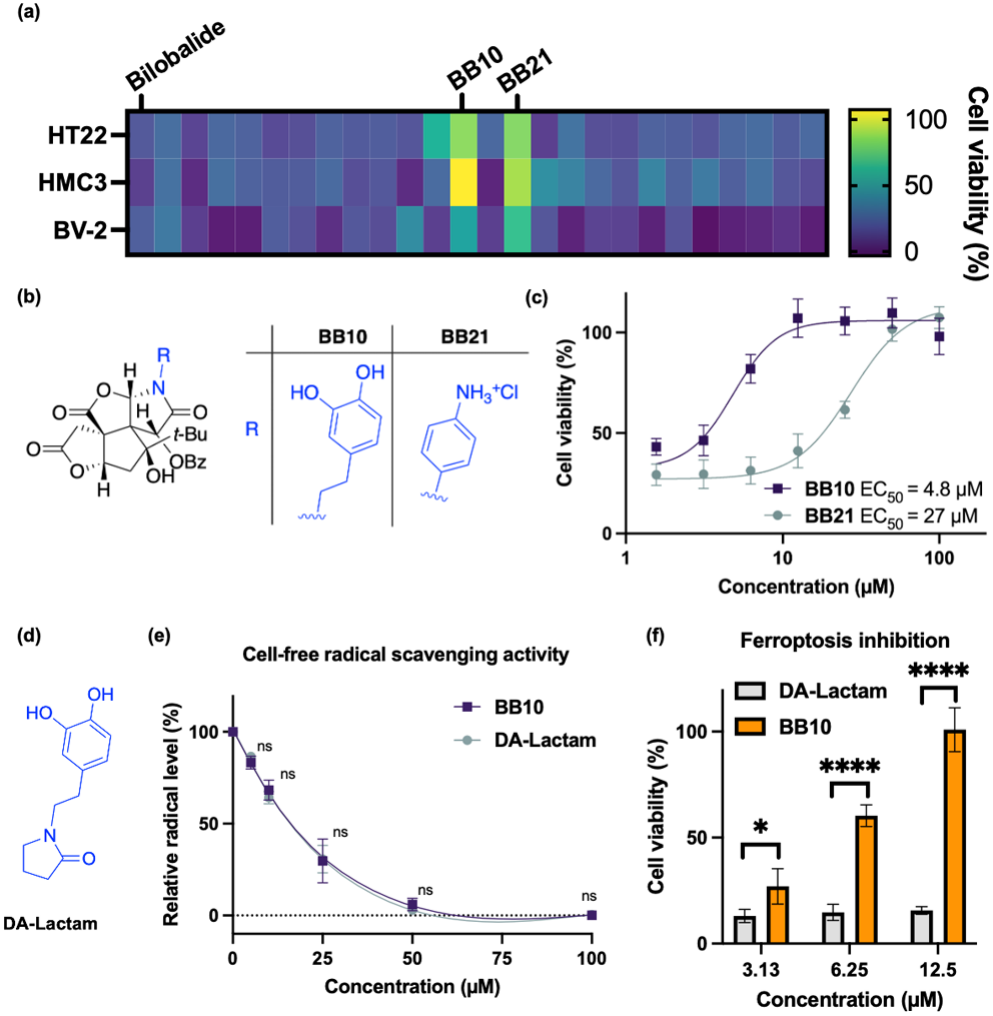
*N*-Arylated bilobalide analogue **BB10** is an unexpected ferroptosis inhibitor. (a) Phenotypic screening against ferroptotic cell death across three cell lines. The cells were pretreated with RSL3 (200 nM for HT22 and HMC3, 500 nM for BV-2) for 2 h, followed by the treatment of bilobalide analogues (50 μM) for 22 h; *n* = 5 technical replicates. (b) Chemical structures of **BB10** and **BB21**. (c) Dose-dependent curves of **BB10** and **BB21**. HMC3 cells were pretreated with 200 nM RSL3 for 2 h, followed by the treatment of **BB10** or **BB21** for 22 h. Data are plotted as mean ± s.d.; *n* = 3 technical replicates. (d) Chemical structures of DA-Lactam. (e) Radical scavenging activities of **BB10** and DA-Lactam are similar, as determined by ABTS [2,2′-azinobis(3-ethylbenzothiazoline-6-sulfonate)] assay. Data are plotted as mean ± s.d., *n* = 3 technical replicates. (f) Ferroptosis inhibition of **BB10** and DA-Lactam. HMC3 cells were pretreated with 200 nM RSL3 for 2 h, followed by the treatment of **BB10** or DA-Lactam for 22 h. Data are plotted as mean ± s.d.; *n* = 3 technical replicates. Statistical analyses were performed by two-way ANOVA with multiple comparisons; ns, no significance; *p < 0.05; ****p < 0.0001 *versus* the DA-Lactam group at the same concentration.

Using the known ferroptosis inducer RSL3,^54^ we established brain cell line-based assays to screen for antiferroptotic activity of our analogues (Figure S9).^55^ All three brain cell lines, including the hippocampal cell line HT22, along with the microglial cell lines HMC3 and BV-2, are highly sensitive to RSL3-induced lethality (Figure 5a), which is consistent with the reported vulnerability of brain cells toward ferroptotic cell death.^52,56^ To our delight, the *N*-arylated analogues **BB10** and **BB21** (Figure 5a,b) could effectively inhibit RSL3-induced ferroptosis across all three cell lines (Figure 5c and Table S2). Intriguingly, bilobalide itself demonstrated no antiferroptotic activity (Figure 5a). **BB10** and **BB21** displayed their antiferroptotic activity in a dose-dependent manner (Figure 5c) and did not show toxicity (Figure S10). The EC_50_ value of **BB10** (4.8 μM) was superior to that of **BB21** (27.3 μM); therefore, **BB10** was selected as the hit compound for subsequent investigations.

**BB10** contains an O-to-N edited bilobalide skeleton and a dihydroxyphenyl moiety, known for its antioxidative properties.^57^ To determine the role of the dihydroxyphenyl group in the antiferroptotic activity of **BB10**, we compared its antioxidation and ferroptosis inhibitory activities with those of a simple lactam analogue containing a dihydroxyphenyl moiety (DA-Lactam, Figure 5d). Interestingly, both compounds exhibited similar antioxidation activity in a free-radical scavenging assay^58^ (Figure 5e). However, **BB10** showed significant protective effects against ferroptosis, while DA-Lactam had considerably lower activity at the same concentration (Figure 5f). To further explore its MOA, we assessed whether **BB10** functions as an iron chelator, a known class of ferroptosis inhibitors.^59^ However, **BB10** showed no significant iron chelation activity (Figure S11). These data suggest that while the effectiveness of **BB10** extends beyond its radical scavenging properties, other MOA likely contribute to its antiferroptotic effects and warrant further investigation.

**Figure 6:**
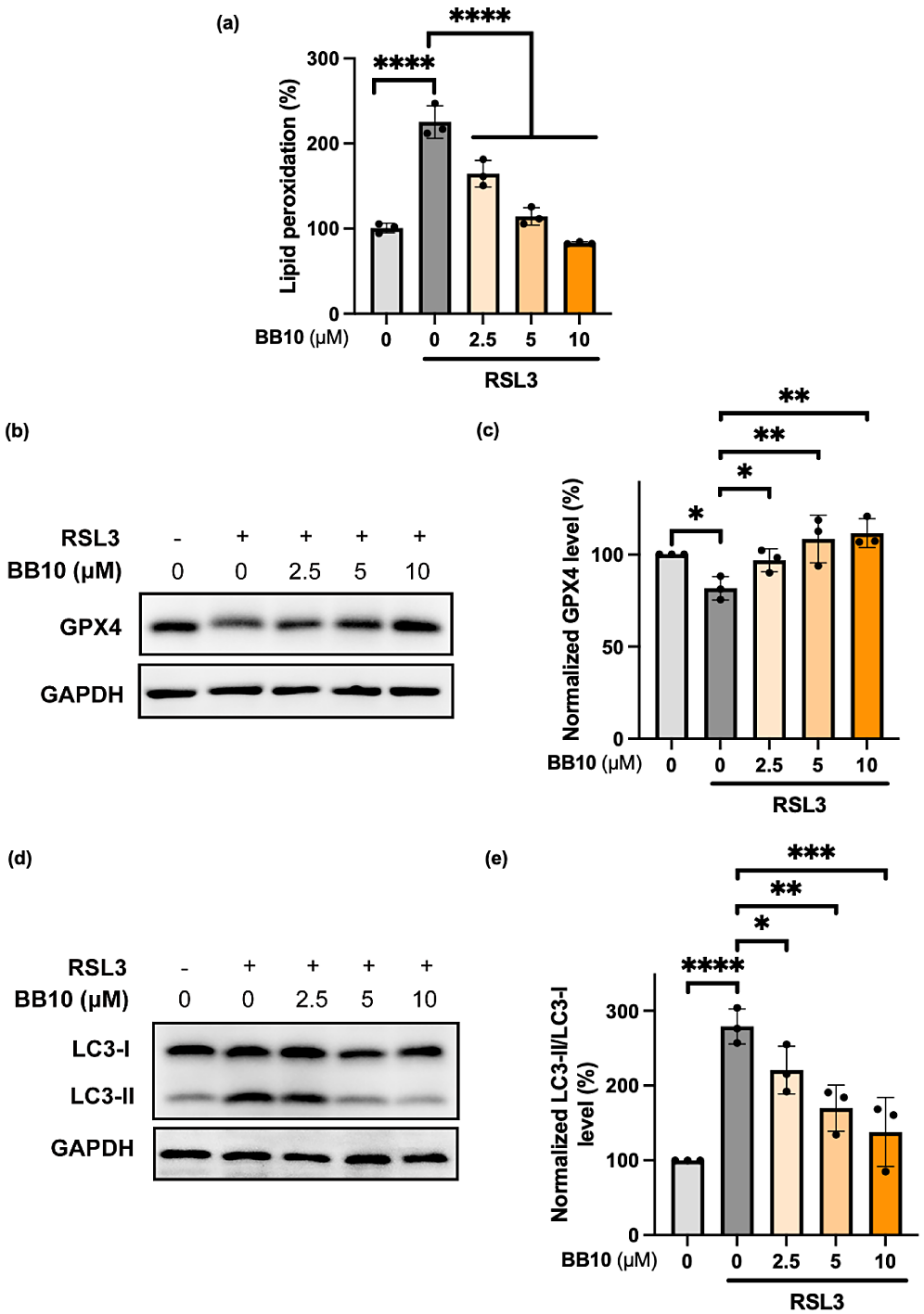
Unprecedented ferroptosis inhibitor **BB10** regulates the GPX4 pathway. (a) Evaluation of lipid peroxidation. HMC3 cells were pretreated with or without 50 nM RSL3 for 2 h and subsequently treated with **BB10** for 3 h. Lipid peroxidation was measured using C11-BODIPY, *n* = 3 biologically independent experiments; data are plotted as mean ± s.d.; statistical analyses were performed by one-way ANOVA with multiple comparisons, ****p < 0.0001 *versus* the RSL3 only treated group. (b) **BB10** restored GPX4 levels against RSL3-induced degradation. HMC3 cells were treated with or without 1 μM RSL3 and **BB10** for 3 h. Protein levels were measured by Western blotting using the indicated antibodies. (c) Normalized GPX4 levels against GAPDH of panel (b). (d) **BB10** downregulated the RSL3-induced LC3-II level. HMC3 cells were treated with or without 1 μM RSL3 and **BB10** for 3 h. Protein levels were measured by Western blotting using the indicated antibodies. (e) Normalized LC3-II/LC3-I levels against GAPDH of panel (d). For (c) and (e), data are plotted as mean ± s.d., *n* = 3 biologically independent experiments. Statistical analyses were performed by one-way ANOVA with multiple comparisons; *p < 0.05, **p < 0.01, ***p < 0.001, ****p < 0.0001 *versus* the RSL3 only treated group.

We next evaluated the ability of **BB10** to modulate lipid peroxidation, a hallmark of ferroptosis. Using the C11-BODIPY lipid peroxidation sensor,^60^ flow cytometry analysis showed that **BB10** dose-dependently reduced RSL3-induced lipid peroxide accumulation (Figure 6a). RSL3, a widely used ferroptosis inducer, covalently inhibits and degrades glutathione peroxidase 4 (GPX4), an enzyme crucial for reducing lipid peroxidation during ferroptosis.^54^ To gain insights into the MOA of **BB10** against RSL3-induced ferroptosis, we performed immunoblotting to evaluate the GPX4 level using the HMC3 cell model. We found that **BB10** could rescue RSL3-induced degradation of GPX4 in a dose-dependent manner (Figures 6b,c and S12). Additionally, **BB10** significantly inhibited RSL3-induced autophagy, as indicated by the level of the key autophagy marker microtubule-associated protein 1 light chain 3 beta-II (LC3-II)^61^ (Figures 6d,e and S13). This suggests that **BB10** may counteract ferroptotic death by modulating the autophagic degradation of GPX4.^62^

To further explore the efficacy of **BB10**, we established cellular rescue assays using well-studied ferroptosis inducers that target different stages of ferroptosis. **BB10** effectively suppressed cell death induced by FIN56 (Figure S14a), a distinct ferroptosis inducer that depletes GPX4 levels,^60^ and significantly alleviated the lethality of covalent GPX4 inhibitors ML162^61^ (Figure S14b) and ML210^63^ (Figure S14c). System X^c–^ inhibitors, such as erastin, were reported to induce ferroptotic cell death^54^ and deplete intracellular glutathione (GSH).^63^ In our cellular assays, **BB10** also strongly suppressed erastin-induced lethality (Figure S14d). Collectively, **BB10** demonstrated effective inhibition of ferroptosis induced by diverse inducers. Further exploration of the in-depth MOA pertaining to **BB10** is warranted.

Given the increasing recognition of the role of ferroptosis in neurodegenerative disorders such as Alzheimer’s and Parkinson’s disease,^43,64,65^ novel antiferroptotic compounds represent promising disease-modifying agents to slow the progression of these conditions.^66^ Antiferroptotic hit compounds like **BB10** merit further hit-to-lead optimization and evaluation in models more relevant to these diseases. Target identification experiments are currently underway in our laboratory, with the aim of gaining more in-depth insights into the molecular target(s) of **BB10**. Nevertheless, our study should catalyze further downstream mechanistic studies and future medicinal chemistry optimizations.

## Conclusion

In conclusion, we have successfully transformed bilobalide into a series of unique lactam analogues with improved stability and altered pharmacology. These molecular editing strategies hinge on the discovery of the simple yet unexpected reactivity of the synthetic intermediates, ultimately enabling the construction of valuable libraries that originated from bilobalide. Phenotypic screening led to the discovery of **BB10** with antiferroptotic activity across multiple brain cell lines. **BB10** significantly reduced key ferroptosis markers and notably counteracted GPX4 degradation induced by RSL3. We are actively elucidating its MOA, which remains incompletely understood in this study and will be reported in due course. Meanwhile, our current findings will inform further optimization and in-depth evaluation of antiferroptotic bilobalide analogues like **BB10** in models that more closely resemble neurodegenerative diseases. More generally, this work demonstrates how subtle molecular editing can breed new profiles of natural products and enable the exploration of previously unknown chemical and biological spaces.

## Methods

### Materials and Reagents

Unless otherwise stated, all syntheses and manipulations of air- and moisture-sensitive materials were carried out under a nitrogen atmosphere using standard Schlenk techniques. All glassware was oven-dried immediately prior to use. Authentic (−)-bilobalide was purchased from Chengdu Must Bio-Technology Co., Ltd. All commercially available solvents or reagents were directly used without further purification unless otherwise noted. Nuclear magnetic resonance (NMR) spectra were recorded on a Bruker Ultrashield 400 Plus NMR spectrometer or Bruker Ascend 500 NMR spectrometer at ambient temperature. High-resolution mass spectra were obtained on a Thermo Q Exactive Focus Hybrid Quadrupole-Orbitrap Mass Spectrometer. X-ray crystallographic analysis was performed on a Bruker D8 Venture Diffractometer. The following antibodies were used: rabbit anti-GPX4 (52455, Cell Signaling Technology), mouse anti-GAPDH (sc-32233, Santa Cruz Biotechnology), rabbit anti-LC3B (A19665, ABclonal Technology), goat antirabbit IgG HRP-linked antibody (7074, Cell Signaling Technology), and goat antimouse IgG (H+L) secondary antibody DyLight 488 (35502, Invitrogen).

### General Procedure for the Synthesis of *N*-Alkylated Bilobalide Analogues and BB14/BB15

To an oven-dried round-bottom flask with a magnetic stir bar, was added **1** or **2** (50 mg, 0.094 mmol, 1.0 equiv), followed by the addition of anhydrous tetrahydrofuran (5 mL). Substituted alkyl amines or dinucleophile hydrazine hydrate (0.188 mmol, 2.0 equiv) was then added to the flask at 0 °C. The resulting solution was warmed to room temperature and stirred for 30 min. Upon completion indicated by TLC, the reaction solution was concentrated *in vacuo*. The residue was dissolved in dichloromethane, and the organic layer was washed with brine. The combined organic layers were dried over Na_2_SO_4_, filtered and concentrated under reduced pressure. The crude product was purified *via* column chromatography to provide the corresponding products **BB01**–**BB15**.

### General Procedure for the Synthesis of *N*-Arylated Bilobalide Analogues

To an oven-dried round-bottom flask containing a magnetic stir bar, were added **BB17** (100 mg, 1 equiv, 0.233 mmol) and aryl/hetero aryl boronic acid (2.0 equiv, 0.466 mmol). [CuOTf]2·toluene (48 mg, 0.093 mmol, 40 mol %), 1,10-phenanthroline (17 mg, 0.093 mmol, 40 mol %), and DMSO (10 mL) were then added sequentially. The reaction mixture was stirred at room temperature in open air for 16 h. Once the starting material was fully consumed, the reaction solution was diluted with 20 mL of ice-cold water and extracted with ethyl acetate (3 × 15 mL). The combined organic layers were washed three times with brine, dried over anhydrous Na_2_SO_4_, and concentrated under reduced pressure. The crude product was purified by column chromatography to provide the desired products **BB18**–**BB32**.

### Cell Viability Assay

HT22, HMC3, or BV-2 cells (5,000 cells per well) were seeded into a 96-well plate and allowed to adhere overnight at 37 °C in a humidified incubator with 5% CO_2_. Afterward, cells were pretreated with 200 nM (on HT22, HMC3) or 500 nM (on BV-2) of RSL3 (Bidepharm) for 2 h, respectively. The medium was then aspirated, and the cells were treated with the indicated compound for 22 h. Cell viability was measured using a Cell Counting Kit-8 assay according to the manufacturer’s instructions.

### Lipid Peroxidation Assay

HMC3 cells (50,000 cells per well) were seeded into a 24-well plate and allowed to adhere overnight at 37 °C in a humidified incubator with 5% CO_2_. The cells were then pretreated with 50 nM RSL3 for 2 h. After RSL3 treatment, the medium was aspirated, and the cells were treated with DMSO (0.1%) or **BB10** (10, 5, and 2.5 μM) for 3 h, after which the medium was replaced with BODIPY 581/591 C11 (10 μM, Invitrogen) for 30 min. The cells were then washed three times with PBS and collected. Fluorescence was measured by the BD FACSymphony A5.2 SORP Flow Cell Analyzer (BD Biosciences).

### Radical Scavenger Activity Evaluation

10 mM of ABTS was mixed with 2.45 mM of ammonium persulfate, and the mixture was incubated for 16 h at room temperature to form the ABTS^•+^ radical. ABTS^•+^ was subsequently diluted by ethanol before testing. 1 μL of the indicated compound was mixed with 99 μL of ABTS^•+^, and the mixture was incubated in the dark for 30 min. The absorbance was then measured on a CLARIOstar plate reader at 734 nm.

### Western Blot Assay

HMC3 cells (100,000 cells per well) were seeded into a 12-well plate and allowed to adhere overnight at 37 °C in a humidified incubator with 5% CO_2_. For cotreatment, ferroptosis inducers RSL3 (1 μM) and **BB10** (10, 5, and 2.5 μM) were added at the same time, and the cells were then incubated for 3 h. After incubation, the cells were washed three times with ice-cold PBS and lysed in RIPA buffer supplemented with 1× protease inhibitor cocktail (MCE) and nuclease (Beyotime). All samples were centrifuged and then quantified by bicinchoninic acid (Pierce). Cell lysates were diluted with Laemmli sample buffer (Bio-Rad) and heated at 95 °C for 5 min. Samples were separated by SDS-PAGE (Vazyme) and transferred to a polyvinylidene difluoride (PVDF) membrane (Thermo Fisher). Transferred membranes were blocked in 3% bovine serum albumin (Sigma-Aldrich) for 1 h and incubated with the indicated primary antibody overnight at 4 °C. The membrane was then incubated with corresponding secondary antibodies for 1 h at room temperature. Blots were washed and visualized using Clarity Western ECL Substrate (Bio-Rad) on the ChemiDoc MP Imaging System (Bio-Rad). Band intensity was normalized by ImageJ software.

## Supporting Information Available

Comparison of X-ray crystallographic structures of bilobalide and BB17 (PDF)
Raw data for antiferroptosis phenotypic screening results (XLSX)
Supporting figures, experimental details and methods, chemical synthesis and characterization data, NMR spectra (PDF)

## Dedication

In memory of Professor Koji Nakanishi (1925–2019).
