# Supplementary material for: Lactone-to-Lactam Editing Alters the Pharmacology of Bilobalide
Source: JACS Au. 2024 Jul 16;4(9):3537–46. doi: 10.1021/jacsau.4c00416 (PMC11423332; doi:10.1021/jacsau.4c00416)
Supplement: Supplementary file 1 — au4c00416_si_008.pdf [file au4c00416_si_008.pdf]

Supporting Information for

Lactone-to-Lactam Editing Alters the Pharmacology of Bilobalide

Xiaoding Jiang,[†#] Xu He,[†#] Jonathan Wong,[†#] Stephan Scheeff,[†] Sam Chun-Kit Hau,[‡] Tak Hin Wong,[†] Yao Qin,[†] Chi Hang Fan,[†] Bowen Ma,[†] Ngai Lam Chung,[†τ] Junzhe Huang,[⊥] Jiajia Zhao,[†] Yu Yan,[†] Min Xiao,[†] Xueqin Song,[†] Tony K.C. Hui,[ξ] Zhong Zuo,[†] William Ka-Kei Wu,[ζΨ] Ho Ko,[⊥ΨΓϕ] Kim Hei-Man Chow,[δϕ] and Billy Wai-Lung Ng*[†ΨΓ]

[†] *School of Pharmacy, Faculty of Medicine, The Chinese University of Hong Kong, Shatin, New Territories, Hong Kong SAR 999077, China*

[‡] *Department of Chemistry, Faculty of Science, The Chinese University of Hong Kong, Shatin, New Territories, Hong Kong SAR 999077, China*

[τ] *Department of Biochemistry, University of Oxford, Oxford OX1 3QU, United Kingdom*

[⊥] *Division of Neurology, Department of Medicine and Therapeutics, Margaret K.L. Cheung Research Centre for Management of Parkinsonism, Faculty of Medicine, The Chinese University of Hong Kong, Shatin, New Territories, Hong Kong SAR 999077, China*

[ξ] *Primemax Biotech Ltd., Wayson Commercial House, 68-70 Lockhard Road, Wan Chai, Hong Kong SAR 999077, China*

[ζ] *Department of Anaesthesia and Intensive Care and Peter Hung Pain Research Institute, The Chinese University of Hong Kong, Shatin, New Territories, Hong Kong SAR 999077, China*

[δ] *School of Life Sciences, Faculty of Science, The Chinese University of Hong Kong, Shatin, New Territories, Hong Kong SAR 999077, China*

[Ψ] *Li Ka Shing Institute of Health Sciences, Faculty of Medicine, The Chinese University of Hong Kong, Shatin, New Territories, Hong Kong SAR 999077, China*

[Γ] *Peter Hung Pain Research Institute, Faculty of Medicine, The Chinese University of Hong Kong, Shatin, New Territories, Hong Kong SAR 999077, China*

[ϕ] *Gerald Choa Neuroscience Institute, The Chinese University of Hong Kong, Shatin, New Territories, Hong Kong SAR 999077, China*

* *Email: billyng@cuhk.edu.hk*

[#] *These authors contributed equally to this work.*

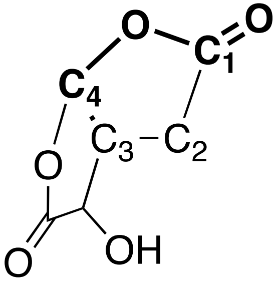

Bilobalide

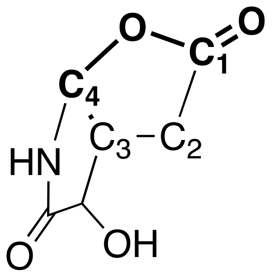

BB17

	Bond Angle (°)		Bond Length (Å)		Bond Angle (°)		Bond Length (Å)
**∠O-C_1-C_2**	**109.7(1)**	**O–C_1**	**1.3704(2)**	**∠O-C_1-C_2**	**110.4(6)**	**O–C_1**	**1.3451(1)**
∠C_1-C_2-C_3	103.0(1)	C_1–C_2	1.5143(3)	∠C_1-C_2-C_3	104.1(6)	C_1–C_2	1.5214(1)
∠C_2-C_3-C_4	100.4(1)	C_2–C_3	1.5569(2)	∠C_2-C_3-C_4	101.7(6)	C_2–C_3	1.5643(1)
**∠C_3-C_4-O**	**109.3(1)**	**C_3–C_4**	**1.5341(3)**	**∠C_3-C_4-O**	**104.1(6)**	**C_3–C_4**	**1.5582(1)**
**∠C_4-O-C_1**	**110.3(1)**	**C_4–O**	**1.3946(2)**	**∠C_4-O-C_1**	**111.2(6)**	**C_4–O**	**1.4362(1)**

*Note: the shortened O-C1 bond of C-ring lactone indicates a stronger O-C1 bond in **BB17**, making it less susceptible to hydrolysis or translactonization. Only C-ring and D ring parts of the structures (bilobalide and **BB17**) are kept for clarity.*
