# Supplementary material for: Lactone-to-Lactam Editing Alters the Pharmacology of Bilobalide
Source: JACS Au. 2024 Jul 16;4(9):3537–46. doi: 10.1021/jacsau.4c00416 (PMC11423332; doi:10.1021/jacsau.4c00416)
Supplement: Supplementary file 3 — au4c00416_si_010.pdf [file au4c00416_si_010.pdf]

Supporting Information for

Lactone-to-Lactam Editing Alters the Pharmacology of Bilobalide

Xiaoding Jiang,[†#] Xu He,[†#] Jonathan Wong,[†#] Stephan Scheeff,[†] Sam Chun-Kit Hau,[‡] Tak Hin Wong,[†] Yao Qin,[†] Chi Hang Fan,[†] Bowen Ma,[†] Ngai Lam Chung,[†τ] Junzhe Huang,[⊥] Jiajia Zhao,[†] Yu Yan,[†] Min Xiao,[†] Xueqin Song,[†] Tony K.C. Hui,[ξ] Zhong Zuo,[†] William Ka-Kei Wu,[ζΨ] Ho Ko,[⊥ΨΓϕ] Kim Hei-Man Chow,[δϕ] and Billy Wai-Lung Ng*[†ΨΓ]

[†] *School of Pharmacy, Faculty of Medicine, The Chinese University of Hong Kong, Shatin, New Territories, Hong Kong SAR 999077, China*

[‡] *Department of Chemistry, Faculty of Science, The Chinese University of Hong Kong, Shatin, New Territories, Hong Kong SAR 999077, China*

[τ] *Department of Biochemistry, University of Oxford, Oxford OX1 3QU, United Kingdom*

[⊥] *Division of Neurology, Department of Medicine and Therapeutics, Margaret K.L. Cheung Research Centre for Management of Parkinsonism, Faculty of Medicine, The Chinese University of Hong Kong, Shatin, New Territories, Hong Kong SAR 999077, China*

[ξ] *Primemax Biotech Ltd., Wayson Commercial House, 68-70 Lockhard Road, Wan Chai, Hong Kong SAR 999077, China*

[ζ] *Department of Anaesthesia and Intensive Care and Peter Hung Pain Research Institute, The Chinese University of Hong Kong, Shatin, New Territories, Hong Kong SAR 999077, China*

[δ] *School of Life Sciences, Faculty of Science, The Chinese University of Hong Kong, Shatin, New Territories, Hong Kong SAR 999077, China*

[Ψ] *Li Ka Shing Institute of Health Sciences, Faculty of Medicine, The Chinese University of Hong Kong, Shatin, New Territories, Hong Kong SAR 999077, China*

[Γ] *Peter Hung Pain Research Institute, Faculty of Medicine, The Chinese University of Hong Kong, Shatin, New Territories, Hong Kong SAR 999077, China*

[ϕ] *Gerald Choa Neuroscience Institute, The Chinese University of Hong Kong, Shatin, New Territories, Hong Kong SAR 999077, China*

** Email: billyng@cuhk.edu.hk*

[#] *These authors contributed equally to this work.*

Table of Contents

1. General considerations..........S3

2. Chemistry part..........S4

2.1 Chemical synthesis..........S4

2.1.1 Benzoylation of bilobalide..........S4

2.1.2 General procedure for the synthesis of *N*-alkylated analogues and BB14/BB15..........S6

2.1.3 Synthesis of 3..........S15

2.1.4 Synthesis of BB16 and BB17..........S16

2.1.5 General procedure for the synthesis of *N*-arylated analogues..........S17

2.1.6 Synthesis of 4 and 5..........S27

2.1.7 Synthesis of BB33..........S28

2.1.8 Synthesis of BB34..........S29

2.1.9 Synthesis of BB35..........S30

2.1.10 Synthesis of BB36..........S31

2.2 X-ray Crystallographic Data..........S33

2.3 Copies of NMR Spectra..........S47

2.4 Method for the investigation of chemical stability..........S91

2.5 HPLC analysis method and spectrum of BB10 and BB21..........S92

3. Biology part..........S93

3.1 General methods..........S93

3.1.1 Cell culture..........S93

3.1.2 Cell viability assay..........S93

3.1.3 Lipid peroxidation assay..........S93

3.1.4 Western blot..........S94

3.1.5 Antagonistic activity on GABAA receptor..........S94

3.1.6 Iron chelation activity evaluation..........S95

3.1.7 Radical scavenger activity evaluation..........S95

3.2 Supplementary figures..........S96

1. General considerations

Unless otherwise stated, all syntheses and manipulations of air- and moisture-sensitive materials were carried out under nitrogen atmosphere using standard Schlenk techniques. All glassware was oven-dried immediately prior to use. Authentic (–)-bilobalide was purchased from Chengdu Must Bio-Technology Co., LTD. Other commercially available solvents or reagents were directly used without further purification unless otherwise noted. Reactions were magnetically stirred and monitored by analytical thin-layer chromatography (TLC). TLC was performed on Merck silica gel 60 F254 TLC glass plates and visualized by exposure to ultraviolet light. Organic solutions were concentrated by rotary evaporation at 20 – 45 °C.

Chromatographic purification of products was accomplished using forced-flow chromatography on silica gel (200 – 300 mesh). Melting points were measured on a OptiMelt using open glass capillaries, and the data is uncorrected. The purity of the resulting compounds was determined by Agilent 1260 high performance liquid chromatography (HPLC) (Agilent Technologies, Santa Clara, CA, United States) and was over 95%. Since there is no ultraviolet absorption of compounds **BB15**, **BB17**, **BB33**~**BB36** under 254 nm, their purity was determined by Agilent 6430 triple quadrupole liquid chromatography/tandem mass spectrometry (LC-MS/MS) (Agilent Technologies, Santa Clara, CA, United States) and was higher than 95%. ^{1}H and ^{13}C{^{1}H} NMR spectra were recorded on a Bruker Ultrashield 400 Plus NMR spectrometer or Bruker Ascend 500 NMR spectrometer at ambient temperature. ^{1}H NMR spectra are referred to the tetramethylsilane signal (δ = 0 ppm). The data for ^{1}H NMR is represented as follows: chemical shift (δ, ppm), multiplicity (s = singlet, d = doublet, t = triplet, m = multiplet, br = broad singlet, coupling constant (s) in Hz, integration). ^{13}C{^{1}H} spectra are internally referenced to residual solvent signals. Data for ^{13}C{^{1}H} are expressed in terms of chemical shift (δ, ppm). High-resolution mass spectra (HRMS) were obtained on a Thermo Q Exactive^{TM} Focus Hybrid Quadrupole-Orbitrap^{TM} Mass Spectrometer. X-ray crystallographic analysis was performed on Bruker D8 Venture Diffractometer. Crystal structural data were collected by the single-crystal X-ray diffraction method with a Bruker D8-Venture system.

2. Chemistry part

2.1 Chemical synthesis

2.1.1 Benzoylation of bilobalide

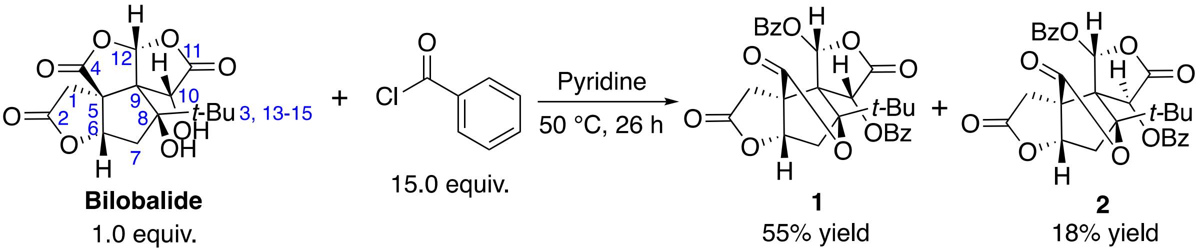

To an oven-dried flask containing a magnetic stirrer bar was added bilobalide (0.3 g, 0.920 mmol, 1.0 equiv.) and benzoyl chloride (1.940 g, 13.800 mmol, 15 equiv.), followed by the addition of anhydrous pyridine (10.0 mL). The reaction was then heated to 50 ^{o}C and stirred for 26 h under nitrogen atmosphere. The reaction was monitored by TLC and upon completion was cooled to room temperature. The reaction solution was diluted with ethyl acetate and transferred to a separatory funnel. The organic layer was washed 3 times with saturated copper sulfate aqueous solution and subsequently washed 3 times with saturated sodium bicarbonate aqueous solution. After that, the combined organic layers were washed 3 times with brine, dried over anhydrous Na_2SO_4, filtered and concentrated *in vacuo*. The crude product was then purified by normal-phase silica gel column chromatography with elution system (hexane/EtOAc= 6:1→hexane/acetone = 6:1, v/v) to afforded **1** and **2** as white powder. *Note: The minor product obtained under similar reaction condition was assigned as 8,10-Di-O-(p-bromobenzoyl)bilobalie previously (see: Liebigs Ann. Chem. 1987, 1079- 1085); but the actual structure of* ***2*** *was confirmed by us using X-ray crystallography, showing that it only differs in the configuration at C-10 compared to the main product* ***1****.*

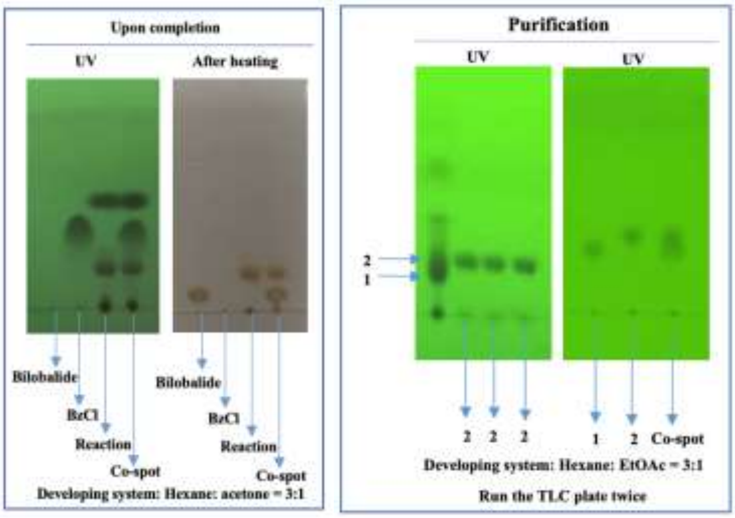

Notes on the purification of 1 and 2 from the reaction: As indicated by the above TLC plates, the polarity of the two products was very similar, so it took us some efforts to get the two products separated. To make the purification easier, we first ran the column with elution system (hexane/EtOAc = 6:1) to elute **2** and then changed the elution system to hexane/acetone = 6:1 to obtain **1**.

(2*R*,3a'*S*,4*S*,6'*R*,7a'*S*)-6'-(*tert*-Butyl)-2',4',5-trioxohexahydro-4'*H*,6'*H*-spiro[furan-3,8'-[3a,6]methanofuro[3,2-*c*]pyran]-2,4-diyl dibenzoate (1)

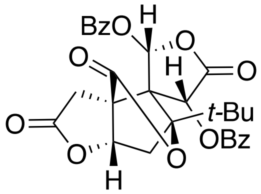

1

White powder, 55% yield, 0.27 g, R_f = 0.41 (hexane/EtOAc = 3:1, UV detection on TLC plate); m.p.: 171.5 - 172.0 °C. ^{1}H NMR (500 MHz, $CDCl_3$) δ 8.12 – 8.06 (m, 2H), 8.05 – 8.01 (m, 2H), 7.76 – 7.71 (m, 1H), 7.70 – 7.64 (m, 1H), 7.61 – 7.55 (m, 2H), 7.54 – 7.48 (m, 2H), 7.36 (s, 1H), 6.23 (s, 1H), 4.46 (dd, *J* = 7.6, 4.5 Hz, 1H), 3.37 (d, *J* = 18.0 Hz, 1H), 3.09 (d, *J* = 18.0 Hz, 1H), 2.94 – 2.74 (m, 2H), 1.28 (s, 9H). ^{13}C NMR (126 MHz, $CDCl_3$) δ 169.60, 165.74, 165.52, 160.63, 160.59, 133.20, 132.87, 128.72, 128.62, 127.76, 127.30, 126.71, 126.31, 100.79, 94.46, 79.55, 69.00, 65.56, 64.44, 38.75, 37.85, 35.49, 34.27, 29.92. HRMS (ESI): Calcd for $C_{29}H_{26}O_{10}$ $[M+Na]^+$: 557.14182, found: 557.14142.

(2*R*,3a'*S*,4*S*,6'*R*,7a'*S*)-6'-(*tert*-Butyl)-2',4',5-trioxohexahydro-4'*H*,6'*H*-spiro[furan-3,8'-[3a,6]methanofuro[3,2-*c*]pyran]-2,4-diyl dibenzoate (2)

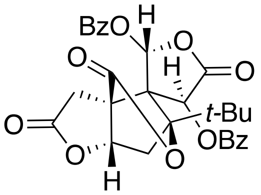

2

White powder, 18% yield, 88 mg, R_f = 0.43 (Hexane/EtOAc = 3:1, UV detection on TLC plate); m.p.: 172.4 - 173.0 °C. ^{1}H NMR (500 MHz, $CDCl_3$) δ 8.06 (d, *J* = 7.7 Hz, 2H), 8.01 (d, *J* = 7.8 Hz, 2H), 7.75 – 7.65 (m, 2H), 7.61 – 7.50 (m, 4H), 7.35 (s, 1H), 6.38 (s, 1H), 4.46 (dd, *J* = 8.2, 3.6 Hz, 1H), 3.27 (s, 2H), 3.05 (dd, *J* = 14.9, 3.6 Hz, 1H), 2.64 (dd, *J* = 14.9, 8.2 Hz, 1H), 1.34 (s, 9H). ^{13}C NMR (126 MHz, $CDCl_3$) δ 172.16, 169.07, 168.72, 163.91, 163.59, 134.84, 134.41, 130.17, 129.97, 129.16, 128.99, 127.68, 127.15, 101.45, 92.86, 79.03, 63.93,

62.75, 61.32, 35.34, 35.03, 30.16, 26.87. HRMS (ESI): Calcd for $C_{29}H_{26}O_{10}$ $[M+Na]^+$: 557.14182, found: 557.14182.

2.1.2 General procedure for the synthesis of *N*-alkylated analogues and BB14/BB15

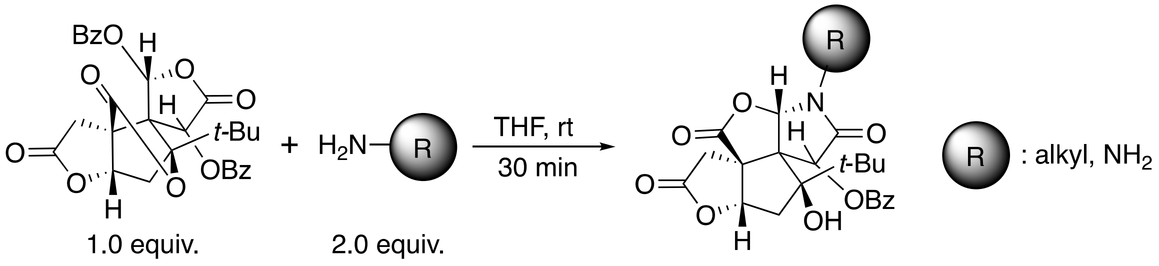

To an oven-dried round bottom flask with a magnetic stirbar was added **1** or **2** (50 mg, 0.094 mmol, 1.0 equiv.), followed by the addition of anhydrous tetrahydrofuran (5 mL). Substituted alkyl amines or dinucleophile hydrazine hydrate (0.188 mmol, 2.0 equiv.) was then added to the flask at 0 °C. The resulting solution was warmed to room temperature and stirred for 30 min. Upon completion indicated by TLC, the reaction solution was concentrated *in vacuo*. The residue was dissolved with dichloromethane and the organic layer was washed with brine. The combined organic layers were dried over Na_2SO_4, filtered and concentrated *in vacuo*. The crude product was purified via column chromatography to provide the corresponding products **BB01 ~ BB15** as a white powder.

Note: 2.0 equivalence of substituted alkyl amines or dinucleophile hydrazine hydrate is enough to the full conversion of the intermediates **1** or **2** in 30 min. If the equivalence exceeds too much and the reaction is run too long, the debenzoylation easily occurs.

(3a*S*,5a*S*,8*R*,9*R*,10a*S*)-6-Benzyl-9-(*tert*-butyl)-9-hydroxy-2,4,7-trioxooctahydro-4*H*,9*H*-furo[3'',2'':2',3']cyclopenta[1',2':3,4]furo[2,3-*b*]pyrrol-8-yl benzoate (BB01)

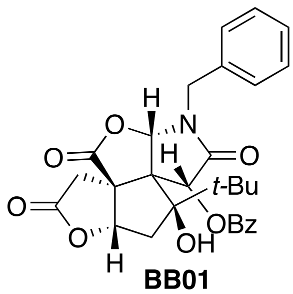

BB01

White powder, 84% yield, 41 mg; R_f = 0.30 (hexane: EtOAc = 2:1, UV detection on TLC plate) ; Melting point: 181.4 – 182.3 °C. ^{1}H NMR (400 MHz, $CDCl_3$) δ 8.16 – 7.90 (m, 2H), 7.69 – 7.60 (m, 1H), 7.56 – 7.45 (m, 2H), 7.43 – 7.31 (m, 5H), 6.36 (s, 1H), 5.73 (s, 1H), 5.20 (t, *J* = 7.1 Hz, 1H), 5.02 (d, *J* = 14.5 Hz, 1H), 4.21 (d, *J* = 14.5 Hz, 1H), 3.20 (d, *J* = 18.9 Hz,

1H), 2.87 (d, J = 18.9 Hz, 1H), 2.62 (dd, J = 14.0, 7.2 Hz, 1H), 2.23 (dd, J = 14.0, 7.1 Hz, 1H), 1.00 (s, 9H). ^{13}C NMR (101 MHz, $CDCl_3$) δ 178.17, 173.80, 166.94, 165.32, 134.32, 134.21, 133.67, 130.16, 130.12, 129.12, 128.80, 128.60, 128.51, 127.76, 87.17, 87.00, 83.48, 70.97, 62.51, 59.40, 44.61, 42.71, 37.21, 36.40, 26.28. HRMS (ESI): Calcd for $C_{29}H_{29}NO_8$ $[M+Na]^+$: 542.17854, found: 542.17839.

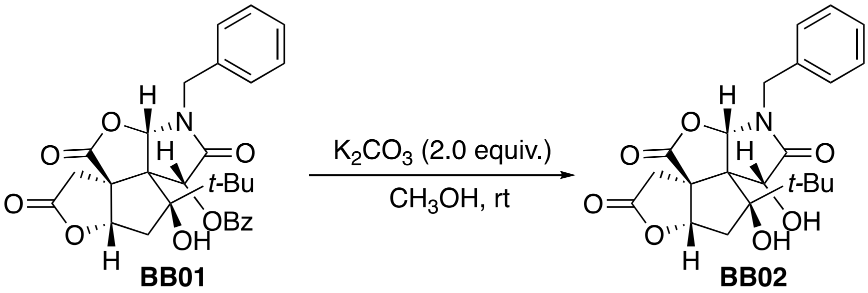

To an oven-dried flask containing a magnetic stirbar was added **BB01** (50 mg, 1 equiv., 0.096 mmol) and methanol (10 mL) , followed by the addition of potassium carbonate (2.0 equiv., 26.6 mg, 0.192 mmol). The resulting solution was allowed to be stirred at room temperature for 4 h. Once the starting material was fully consumed, the reaction solution was concentrated under reduced pressure and then the residue was resuspended with water. The pH value of the resuspension was adjusted to 7.0 using 3 N HCl aqueous solution. The mixture was extracted 3 times with ethyl acetate and washed 3 times with brine, respectively. The combined organic layers were dried over anhydrous sodium sulfate, filtered and concentrated under reduced pressure. The crude product was purified by column chromatography with elution system (hexane: ethyl acetate =1:2, v/v) to give **BB02** as white powder.

(3a*S*,5a*S*,8*R*,9*R*,10a*S*)-6-Benzyl-9-(*tert*-butyl)-8,9-dihydroxytetrahydro-4*H*,9*H*-furo[3'',2'':2',3']cyclopenta[1',2':3,4]furo[2,3-*b*]pyrrole-2,4,7(3*H*,8*H*)-trione (BB02)

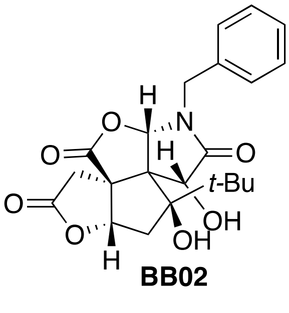

R$_f$ = 0.15 (hexane:EtOAc = 1:2, UV detection on TLC plate); Melting point: 191.2-191.8°C; ^{1}H NMR (500 MHz, Methanol-d_4) δ 7.39 – 7.34 (m, 2H), 7.33 – 7.28 (m, 3H), 5.59 (s, 1H), 5.02 (t, J = 6.9 Hz, 1H), 4.94 – 4.87 (m, 2H), 4.21 (d, J = 14.7 Hz, 1H), 2.99 (dd, J = 17.9, 1.3 Hz, 1H), 2.65 (dd, J = 18.0, 1.4 Hz, 1H), 2.58 (dd, J = 13.5, 7.2 Hz, 1H), 2.29 (dd, J = 13.5, 6.8 Hz, 1H), 0.99 (s, 9H); ^{13}C NMR (126 MHz, Methanol-d_4) δ 180.17, 176.43, 174.65, 136.72,

130.10, 129.67, 129.34, 88.52, 87.43, 85.41, 71.07, 65.32, 61.01, 45.38, 43.15, 38.48, 38.37, 27.3. HRMS (ESI): Calcd for $C_{22}H_{25}NO_7$ $[M+Na]^+$: 438.15232, found: 438.15233.

(3a*S*,5a*S*,8*S*,9*R*,10a*S*)-9-(*tert*-Butyl)-6-(2,4-dimethoxybenzyl)-9-hydroxy-2,4,7-trioxooctahydro-4*H*,9*H*-furo[3'',2'':2',3']cyclopenta[1',2':3,4]furo[2,3-*b*]pyrrol-8-yl benzoate (BB03)

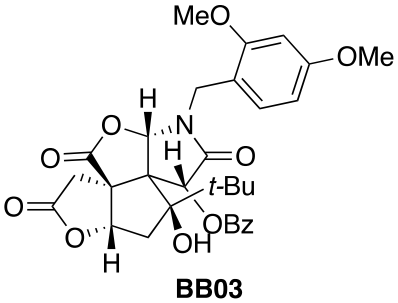

BB03

White powder, 90% yield, 49 mg; R_f = 0.2 (hexane : EtOAc = 3:1, UV detection on TLC plate); Melting point: 102.3 – 102.9 °C. ^{1}H NMR(500 MHz, $CDCl_3$) δ 7.99 (d, *J* = 7.7 Hz, 2H), 7.62 (t, *J* = 7.3 Hz, 1H), 7.48 (t, *J* = 7.7 Hz, 2H), 7.21 (d, *J* = 8.8 Hz, 1H), 6.51 – 6.43 (m, 2H), 6.24 (s, 1H), 5.72 (s, 1H), 5.17 (t, *J* = 7.1 Hz, 1H), 4.97 (d, *J* = 14.3 Hz, 1H), 4.08 (d, *J* = 14.4 Hz, 1H), 3.85 (s, 3H), 3.82 (s, 3H), 3.21 (d, *J* = 19.0 Hz, 1H), 2.90 (d, *J* = 19.0 Hz, 1H), 2.62 (dd, *J* = 13.9, 7.1 Hz, 1H), 2.15 (dd, *J* = 13.9, 7.2 Hz, 1H), 0.95 (s, 9H). ^{13}C NMR (126 MHz, $CDCl_3$) δ 178.45, 173.79, 166.90, 165.26, 161.38, 158.68, 134.14, 132.23, 130.06, 128.72, 127.92, 114.19, 104.10, 98.69, 87.25, 87.12, 83.44, 70.90, 61.84, 59.46, 55.45, 55.37, 42.77, 41.11, 37.14, 36.45, 26.14. HRMS (ESI): Calcd for $C_{31}H_{33}NO_{10}$ $[M+Na]^+$: 602.19967, found: 602.19967.

***tert*-Butyl (3*R*)-3-(((3a*S*,5a*S*,8*R*,9*R*,10a*S*)-8-(benzoyloxy)-9-(*tert*-butyl)-9-hydroxy-2,4,7-trioxohexahydro-4*H*,9*H*-furo[3'',2'':2',3']cyclopenta[1',2':3,4]furo[2,3-*b*]pyrrol-6(5a*H*)-yl)methyl)pyrrolidine-1-carboxylate (BB04)**

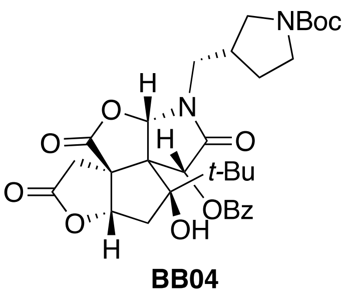

BB04

White powder, 82% yield, 48 mg; R_f = 0.2 (hexane: EtOAc = 3:1, UV detection on TLC plate); Melting point: 126.7 – 127.2 °C. ^{1}H NMR (500 MHz, $CDCl_3$) δ 8.11 – 7.88 (m, 2H), 7.61 (t, *J*

= 7.5 Hz, 2H), 7.48 (t, *J* = 7.7 Hz, 2H), 6.30 (s, 1H), 5.96 (s, 1H), 5.03 (t, *J* = 7.0 Hz, 1H), 3.87 (dd, *J* = 14.2, 4.8 Hz, 1H), 3.59 – 3.34 (m, 3H), 3.29 – 3.01 (m, 3H), 2.96 – 2.46 (m, 3H), 2.24 – 2.09 (m, 1H), 2.01 – 1.90 (m, 1H), 1.79 – 1.58 (m, 1H), 1.47 (s, 9H), 1.12 (s, 9H). ^{13}C NMR (126 MHz, CDCl$_3$) δ 178.33, 173.77, 167.25, 165.28, 155.44, 134.20, 130.06, 128.76, 127.86, 87.96, 86.23, 83.76, 80.14, 70.79, 63.11, 59.24, 49.73, 45.77, 43.17, 42.24, 37.57, 37.32, 36.44, 29.17, 28.58, 28.50, 26.57. HRMS (ESI): Calcd for $C_{32}H_{40}N_2O_{10}$ [M+Na]$^+$: 635.25752, found: 635.25714.

***tert*-Butyl 4-(((3a*S*,5a*S*,8*S*,9*R*,10a*S*)-8-(benzoyloxy)-9-(*tert*-butyl)-9-hydroxy-2,4,7-trioxo-hexahydro-4*H*,9*H*-furo[3'',2'':2',3']cyclopenta[1',2':3,4]furo[2,3-*b*]pyrrol-6(5a*H*)-yl)me-thyl)piperidine-1-carboxylate (BB05)**

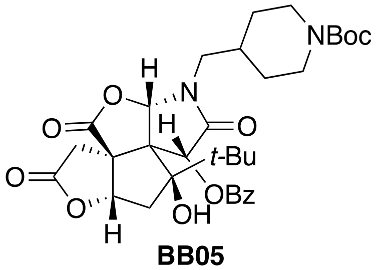

BB05

White powder, 72% yield, 42 mg; R_f = 0.2 (hexane: EtOAc = 2:1, UV detection on TLC plate); Melting point: 156.7 – 157.4 °C. ^{1}H NMR (500 MHz, CDCl$_3$) δ 7.98 (d, *J* = 7.7 Hz, 2H), 7.63 (t, *J* = 7.5 Hz, 1H), 7.49 (t, *J* = 7.7 Hz, 2H), 6.36 (s, 1H), 5.95 (s, 1H), 5.19 (t, *J* = 7.2 Hz, 1H), 4.21 – 3.92 (m, 3H), 3.45 (d, *J* = 12.5 Hz, 1H), 3.23 – 2.95 (m, 3H), 2.87 (d, *J* = 18.8 Hz, 1H), 2.76 – 2.61 (m, 3H), 2.26 – 2.16 (m, 1H), 1.95 (q, *J* = 10.5, 9.9 Hz, 1H), 1.85 – 1.71 (m, 1H), 1.57 (m, 1H), 1.45 (d, *J* = 2.0 Hz, 9H), 1.10 (s, 9H). ^{13}C NMR (126 MHz, CDCl$_3$) δ 177.89, 173.49, 173.46, 167.45, 165.26, 154.74, 134.27, 130.07, 128.77, 127.75, 87.21, 87.19, 87.17, 83.36, 83.34, 79.75, 70.67, 62.73, 62.71, 59.27, 42.87, 42.85, 42.83, 37.42, 36.46, 34.37, 29.81, 28.42, 26.46. HRMS (ESI): Calcd for $C_{33}H_{42}N_2O_{10}$ [M+Na]$^+$: 649.27317, found: 649.27258.

***tert*-Butyl 4-(2-((3a*S*,5a*S*,8*S*,9*R*,10a*S*)-8-(benzoyloxy)-9-(*tert*-butyl)-9-hydroxy-2,4,7-trio-xohexahydro-4*H*,9*H*-furo[3'',2'':2',3']cyclopenta[1',2':3,4]furo[2,3-*b*]pyrrol-6(5a*H*)-yl)e-thyl)piperidine-1-carboxylate (BB06)**

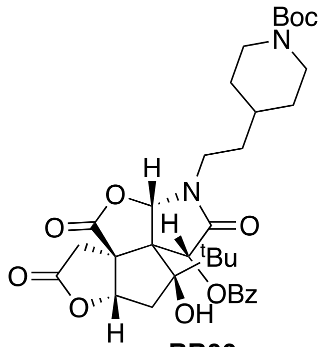

BB06

White powder, 86% yield, 51 mg; R_f = 0.2 (hexane: EtOAc = 2:1, UV detection on TLC plate) Melting point: 136.7 – 137.4 °C. ^{1}H NMR (400 MHz, $CDCl_3$) δ 8.04 – 7.91 (m, 2H), 7.67 – 7.56 (m, 1H), 7.55 – 7.41 (m, 2H), 6.32 (s, 1H), 5.95 (s, 1H), 5.20 (t, *J* = 7.1 Hz, 1H), 4.20 – 3.98 (m, 2H), 3.60 (ddd, *J* = 13.8, 9.5, 6.2 Hz, 1H), 3.32 (ddd, *J* = 14.3, 9.4, 5.7 Hz, 1H), 3.16 (d, *J* = 18.8 Hz, 1H), 2.87 (d, *J* = 18.8 Hz, 1H), 2.76 – 2.57 (m, 4H), 2.27 – 2.19 (m, 1H), 2.18 (s, 1H), 1.68 (d, *J* = 8.2 Hz, 3H), 1.65 – 1.49 (m, 2H), 1.45 (s, 9H), 1.10 (s, 9H). ^{13}C NMR (101 MHz, $CDCl_3$) δ 178.01, 173.70, 167.15, 165.29, 154.91, 134.25, 130.06, 128.77, 127.81, 87.81, 86.96, 83.56, 79.61, 70.93, 62.87, 59.31, 42.60, 38.89, 37.41, 36.44, 33.81, 33.71, 31.72, 28.45, 26.49. HRMS (ESI): Calcd for $C_{34}H_{44}N_2O_{10}$ [M+H]$^+$: 663.28882, found: 663.28809.

***tert*-Butyl 4-(3-((3a*S*,5a*S*,8*S*,9*R*,10a*S*)-8-(benzoyloxy)-9-(*tert*-butyl)-9-hydroxy-2,4,7-trioxohexahydro-4*H*,9*H*-furo[3'',2'':2',3']cyclopenta[1',2':3,4]furo[2,3-*b*]pyrrol-6(5a*H*)-yl)propyl)piperidine-1-carboxylate (BB07)**

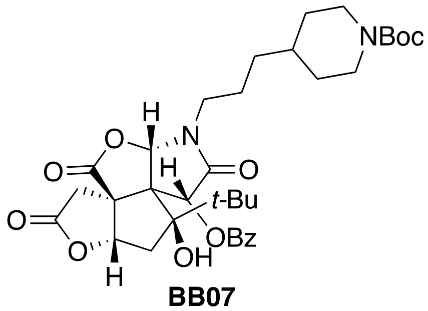

BB07

White powder, 78% yield, 48 mg; R_f = 0.2 (hexane: EtOAc = 2:1, UV detection on TLC plate); Melting point: 127.8 – 128.6 °C. ^{1}H NMR (400 MHz, $CDCl_3$) δ 8.04 – 7.91 (m, 2H), 7.67 – 7.58 (m, 1H), 7.49 (t, *J* = 7.8 Hz, 2H), 6.33 (s, 1H), 5.97 (s, 1H), 5.20 (t, *J* = 7.1 Hz, 1H), 4.05 (d, *J* = 13.1 Hz, 2H), 3.60 – 3.47 (m, 1H), 3.33 – 3.21 (m, 1H), 3.16 (d, *J* = 18.9 Hz, 1H), 3.03 – 2.95 (m, 1H), 2.87 (d, *J* = 18.7 Hz, 1H), 2.75 – 2.54 (m, 2H), 2.22 (dd, *J* = 13.9, 7.1 Hz, 1H), 1.83 – 1.77 (m, 1H), 1.73 – 1.53 (m, 4H), 1.45 (s, 9H), 1.42 – 1.35 (m, 1H), 1.26 – 1.20 (m, 2H), 1.10 (s, 9H). ^{13}C NMR (101 MHz, $CDCl_3$) δ 178.08, 173.80, 167.22, 165.35, 155.00, 134.26, 130.07, 128.77, 127.81, 87.88, 86.95, 83.60, 79.58, 70.97, 62.85, 60.57, 59.34, 42.62,

41.31, 37.41, 36.45, 35.49, 33.51, 32.02, 28.46, 26.50, 24.33. HRMS (ESI): Calcd for $C_{35}H_{46}N_2O_{10}$ $[M+Na]^+$: 677.30447, found: 677.30410.

***tert*-Butyl 4-(2-((3a*S*,5a*S*,8*S*,9*R*,10a*S*)-8-(benzoyloxy)-9-(*tert*-butyl)-9-hydroxy-2,4,7-trioxohexahydro-4*H*,9*H*-furo[3'',2'':2',3']cyclopenta[1',2':3,4]furo[2,3-*b*]pyrrol-6(5a*H*)-yl)ethyl)piperazine-1-carboxylate (BB08)**

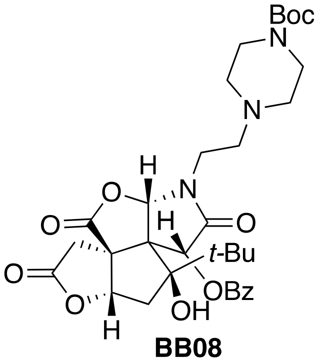

BB08

White powder, 85% yield, 51 mg; R_f = 0.1 (hexane: EtOAc = 2:1, UV detection on TLC plate); Melting point: 134.4 – 135.1 °C. ^{1}H NMR (400 MHz, $CDCl_3$) δ 8.03 – 7.95 (m, 2H), 7.62 (t, *J* = 7.4 Hz, 1H), 7.49 (t, *J* = 7.8 Hz, 2H), 6.36 (s, 1H), 6.20 (s, 1H), 5.21 (t, *J* = 7.1 Hz, 1H), 3.80 (dt, *J* = 14.7, 5.5 Hz, 1H), 3.60 – 3.21 (m, 5H), 3.17 (d, *J* = 18.9 Hz, 1H), 2.90 (d, *J* = 18.9 Hz, 1H), 2.81 (s, 1H), 2.71 – 2.58 (m, 3H), 2.55 – 2.30 (m, 5H), 2.23 (dd, *J* = 13.9, 7.1 Hz, 1H), 1.46 (s, 9H), 1.11 (s, 9H). ^{13}C NMR (101 MHz, $CDCl_3$) δ 178.28, 173.68, 167.09, 165.30, 154.79, 134.20, 130.08, 128.75, 127.85, 88.37, 87.13, 83.45, 79.97, 70.91, 62.48, 59.31, 55.33, 52.68, 42.84, 37.41, 36.75, 36.57, 28.40, 26.51. HRMS (ESI): Calcd for $C_{33}H_{43}N_3O_{10}$ $[M+H]^+$: 642.30212, found: 642.30203.

(3a*S*,5a*S*,8*S*,9*R*,10a*S*)-9-(*tert*-Butyl)-9-hydroxy-6-(2-(3-methyl-1*H*-indol-2-yl)ethyl)-2,4,7-trioxooctahydro-4*H*,9*H*-furo[3'',2'':2',3']cyclopenta[1',2':3,4]furo[2,3-*b*]pyrrol-8-yl benzoate (BB09)

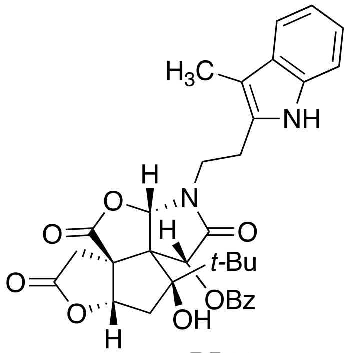

BB09

White powder, 82% yield, 45 mg; R_f = 0.15 (hexane: EtOAc = 2:1, UV detection on TLC plate) Melting point: 143.2 – 144.1 °C. ^{1}H NMR (500 MHz, Methanol-d_4) δ 7.97 (d, *J* = 7.7 Hz, 2H), 7.71 – 7.59 (m, 1H), 7.49 (t, *J* = 7.6 Hz, 3H), 7.27 – 7.17 (m, 1H), 7.10 – 6.92 (m, 2H), 6.05 (s, 1H), 5.19 (s, 1H), 5.05 (t, *J* = 7.1 Hz, 1H), 3.81 (ddd, *J* = 14.2, 6.8, 3.2 Hz, 1H), 3.55 (ddd, *J* = 14.1, 10.5, 6.1 Hz, 1H), 3.28 (ddd, *J* = 14.5, 8.3, 4.6 Hz, 1H), 3.01 – 2.74 (m, 3H), 2.56 (dd, *J* = 13.7, 7.2 Hz, 1H), 2.42 (s, 3H), 2.00 – 1.90 (m, 1H), 0.62 (s, 9H). ^{13}C NMR (126 MHz, Methanol-d_4) δ 179.48, 175.81, 169.40, 166.43, 137.22, 135.24, 133.86, 130.92, 129.90, 129.46, 121.83, 120.19, 118.25, 111.70, 107.24, 90.32, 87.35, 85.36, 72.45, 64.50, 60.48, 42.95, 42.45, 37.97, 37.17, 26.54, 22.22, 11.44. HRMS (ESI): Calcd for $C_{33}H_{34}N_2O_8$ $[M+Na]^+$: 609.22074, found: 609.22060.

(3a*S*,5a*S*,8*R*,9*R*,10a*S*)-9-(*tert*-Butyl)-6-(3,4-dihydroxyphenethyl)-9-hydroxy-2,4,7-trioxo-octahydro-4*H*,9*H*-furo[3'',2'':2',3']cyclopenta[1',2':3,4]furo[2,3-*b*]pyrrol-8-yl benzoate (BB10)

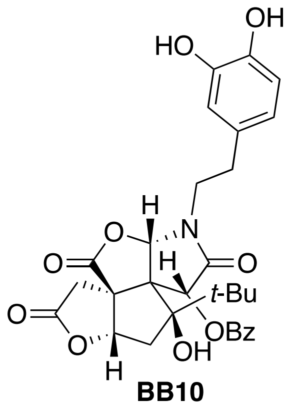

BB10

*Note: **1** (20 mg, 0.037 mmol, 1.0 equiv.) and dihydroxyphenethylamine hydrochloride (11 mg, 0.056 mmol, 1.5 equiv.) were used with 0.3 mL of CH_3CN as solvent and Et_3N (70 μL, 0.502 mmol, 14 equiv.) as the base in this reaction.*

White powder, 76% yield, 16 mg; R_f = 0.15 (hexane: EtOAc = 2:1, UV detection on TLC plate) ^{1}H NMR (700 MHz, Methanol-d_4) δ 7.96 (d, *J* = 7.6 Hz, 2H), 7.67 (t, *J* = 7.5 Hz, 1H), 7.50 (t, *J* = 7.7 Hz, 2H), 6.72 (d, *J* = 8.0 Hz, 1H), 6.70 (d, *J* = 2.3 Hz, 1H), 6.59 (dd, *J* = 8.0, 2.2 Hz, 1H), 6.18 (s, 1H), 5.65 (s, 1H), 5.11 (t, *J* = 7.2 Hz, 1H), 3.82 (m, 1H), 3.55 – 3.49 (m, 1H), 2.87 (d, *J* = 3.1 Hz, 2H), 2.82 (m, 1H), 2.69 – 2.63 (m, 1H), 2.07 – 2.03 (m, 1H), 0.91 (s, 9H). ^{13}C NMR (126 MHz, Methanol-d_4) δ 179.4, 175.8, 169.3, 166.5, 146.7, 145.3, 135.3, 130.9, 130.5, 129.9, 129.5, 121.0, 116.9, 116.7, 89.9, 89.9, 87.5, 85.4, 72.4, 64.5, 60.6, 43.1, 42.9, 38.3, 37.2, 33.3, 26.9, 14.5. HRMS (ESI): Calcd for $C_{30}H_{31}NO_{10}$ $[M+Na]^+$: 588.18402, found: 588.18439.

(3a*S*,5a*S*,8*S*,9*R*,10a*S*)-9-(*tert*-Butyl)-6-(2,4-dimethoxybenzyl)-9-hydroxy-2,4,7-trioxooct-a-hydro-4*H*,9*H*-furo[3'',2'':2',3']cyclopenta[1',2':3,4]furo[2,3-*b*]pyrrol-8-yl benzoate (BB11)

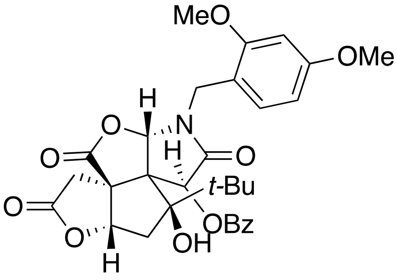

BB11

White powder, 86% yield, 47 mg; R_f = 0.2 (hexane: EtOAc = 3:1, UV detection on TLC plate) Melting point: 106.2 – 107.1 °C. ^{1}H NMR (500 MHz, $CDCl_3$) δ 7.80 (d, *J* = 7.6 Hz, 2H), 7.60 (t, *J* = 7.5 Hz, 1H), 7.42 (t, *J* = 7.7 Hz, 2H), 7.24 (d, *J* = 8.2 Hz, 1H), 6.50 (s, 1H), 6.42 (dd, *J* = 8.3, 2.3 Hz, 1H), 6.03 (s, 1H), 5.80 (s, 1H), 5.16 (t, *J* = 7.1 Hz, 1H), 4.87 (d, *J* = 14.2 Hz, 1H), 4.19 (s, 1H), 3.82 (s, 3H), 3.76 (s, 3H), 2.99 (s, 2H), 2.61 (dd, *J* = 14.3, 7.1 Hz, 1H), 2.13 (dd, *J* = 14.3, 7.2 Hz, 1H), 0.95 (s, 9H). ^{13}C NMR (126 MHz, $CDCl_3$) δ 177.85, 172.26, 167.15, 164.99, 161.26, 158.70, 133.99, 132.25, 130.15, 128.67, 128.57, 114.73, 104.17, 98.68, 90.98, 90.49, 83.54, 71.21, 61.09, 60.40, 55.58, 55.43, 42.62, 39.98, 37.26, 35.18, 26.69. HRMS (ESI): Calcd for $C_{31}H_{33}NO_{10}$ $[M+Na]^+$: 602.19967, found: 602.19961.

(3a*S*,5a*S*,8*S*,9*R*,10a*S*)-6-Benzyl-9-(*tert*-butyl)-9-hydroxy-2,4,7-trioxooctahydro-4*H*,9*H*-f-uro[3'',2'':2',3']cyclopenta[1',2':3,4]furo[2,3-*b*]pyrrol-8-yl benzoate (BB12)

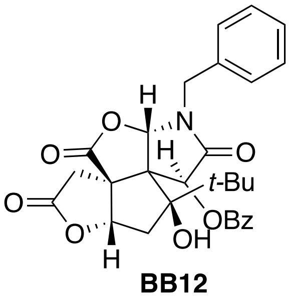

BB12

White powder, 80% yield, 39 mg; R_f = 0.35 (hexane: EtOAc = 2:1, UV detection on TLC plate); Melting point: 112.3 – 113.0 °C. ^{1}H NMR (400 MHz, $CDCl_3$) δ 7.94 – 7.87 (m, 2H), 7.67 – 7.59 (m, 1H), 7.51 – 7.45 (m, 2H), 7.38 – 7.34 (m, 2H), 7.33 – 7.28 (m, 2H), 5.91 (s, 1H), 5.88 (s, 1H), 5.16 (t, *J* = 7.2 Hz, 1H), 5.06 (d, *J* = 14.6 Hz, 1H), 4.10 (d, *J* = 14.6 Hz, 1H), 2.95 (s, 2H), 2.70 – 2.58 (m, 1H), 2.13 (dd, *J* = 14.2, 7.4 Hz, 1H), 0.92 (s, 9H). ^{13}C NMR (101 MHz, $CDCl_3$) δ 177.80, 172.25, 167.73, 165.06, 134.45, 134.33, 130.36, 129.17, 128.87,

128.73, 128.66, 128.62, 90.75, 90.22, 83.71, 77.36, 71.10, 61.25, 60.41, 44.43, 42.70, 37.36, 35.27, 26.83. HRMS (ESI): Calcd for $C_{29}H_{29}NO_8$ $[M+Na]^+$: 542.17854, found: 542.17850.

(3a*S*,5a*S*,8*S*,9*R*,10a*S*)-9-(*tert*-Butyl)-6-cyclopentyl-9-hydroxy-2,4,7-trioxooctahydro-4*H*, 9*H*-furo[3'',2'':2',3']cyclopenta[1',2':3,4]furo[2,3-*b*]pyrrol-8-yl benzoate (BB13)

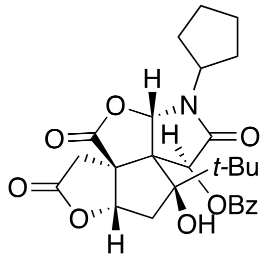

BB13

Using **1** (0.16 g, 0.30 mmol, 1.00 equiv.), amine (2.50 equiv.) in THF (2.5 mL) yielding in **BB13** as a white powder (110 mg, 74%). ^{1}H NMR (600 MHz, $CDCl_3$) δ 7.97 (d, *J* = 7.3 Hz, 2H), 7.61 (t, *J* = 7.4 Hz, 1H), 7.48 (t, *J* = 7.9 Hz, 2H), 6.27 (s, 1H), 6.02 (s, 1H), 5.16 (t, *J* = 7.1 Hz, 1H), 4.10 (p, *J* = 7.1 Hz, 1H), 3.16 (d, *J* = 18.9 Hz, 1H), 2.848 (d, *J* = 19.0 Hz, 1H), 2.73 (s, 1H), 2.63 (dd, *J* = 7.1, 13.9 Hz, 1H), 2.18 (dd, *J* = 7.1, 13.9 Hz, 1H), 1.98 – 2.05 (m, 1H), 1.88 – 1.95 (m, 1H), 1.75 – 1.88 (m, 3H), 1.64 – 1.73 (m, 1H), 1.54 – 1.63 (m, 2H), 1.05 (s, 9H); ^{13}C NMR (150 MHz, $CDCl_3$) δ 178.2, 173.3, 167.3, 165.4, 134.3, 130.2, 128.8, 127.9, 88.0, 87.5, 83.4, 71.0, 62.7, 59.6, 55.1, 42.9, 37.4, 36.3, 30.9, 28.4, 26.5, 24.0, 23.8. HRMS (ESI): Calcd for $C_{27}H_{31}NO_8$ $[M+Na]^+$: 520.19419, found 520.19347.

(3a*S*,5a*S*,8*S*,9*R*,10a*S*)-6-Amino-9-(*tert*-butyl)-9-hydroxy-2,4,7-trioxooctahydro-4*H*,9*H*-furo[3'',2'':2',3']cyclopenta[1',2':3,4]furo[2,3-*b*]pyrrol-8-yl benzoate (BB14)

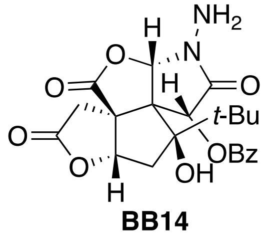

BB14

Note: the reaction for synthesis of this analogue was conducted at -10 °C. if running this reaction at room temperature too long, the product would be debenzoylated.

White powder, 78% yield, 32 mg; R_f = 0.1 (hexane: EtOAc = 1:1, UV detection on TLC plate) Melting point: 108.4 – 108.2 °C. ^{1}H NMR (500 MHz, Methanol-*d*$_4$) δ 8.09 – 7.93 (m, 2H), 7.74 – 7.61 (m, 1H), 7.52 (t, *J* = 7.8 Hz, 2H), 6.40 (s, 1H), 5.97 (s, 1H), 5.19 (t, *J* = 7.2 Hz, 1H), 3.35 (d, *J* = 17.7 Hz, 1H), 3.03 (s, 2H), 2.74 (dd, *J* = 13.7, 7.2 Hz, 1H), 2.14 (dd, *J* = 13.7, 7.2

Hz, 1H), 1.09 (s, 9H). ^{13}C NMR (126 MHz, Methanol-d_4)_δ 178.44, 174.63, 166.93, 165.13, 133.92, 129.59, 128.51, 128.05, 89.70, 86.22, 84.13, 70.04, 62.50, 59.42, 41.79, 37.02, 36.19, 25.59. HRMS (ESI): Calcd for $C_{22}H_{24}N_2O_8$ $[M+H]^+$: 445.16122, found: 445.16126.

(3a*S*,5a*S*,8*S*,9*R*,10a*S*)-6-Amino-9-(*tert*-butyl)-8,9-dihydroxytetrahydro-4*H*,9*H*-furo[3'',2'':2',3']cyclopenta[1',2':3,4]furo[2,3-*b*]pyrrole-2,4,7(3*H*,8*H*)-trione (BB15)

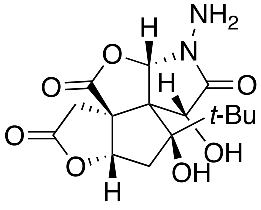

BB15

Note: if running this reaction at room temperature too long, product **BB14** would be debenzoylated to give **BB15**. The use of 5.0 equiv. of hydrazine hydrate led to the formation of **BB15** as major product.

White powder, 32% yield, 10 mg; R_f = 0.21 (dichloromethane/methanol = 50:1, staining with phosphomolybdic acid on TLC plate). ^{1}H NMR (400 MHz, Methanol-d_4): δ 5.72 (s, 1H), 5.04 (t, *J* = 7.0 Hz, 1H), 4.87 (s, 1H), 2.96 (d, *J* = 18.1 Hz, 1H), 2.69 (d, *J* = 18.1 Hz, 1H), 2.60 (dd, *J* = 7.2, 13.5 Hz, 1H), 2.31 (dd, *J* = 6.8, 13.5 Hz, 1H), 1.11 (s, 9H); ^{13}C NMR (150 MHz, Methanol-d_4): δ 180.2, 176.2, 173.0, 90.9, 87.3, 85.0, 69.3, 64.6, 60.8, 42.8, 38.4, 38.1, 27.0. HRMS (ESI): Calcd for $C_{15}H_{20}N_2O_7$ $[M+ Na]^+$: 363.11627, found 363.11541.

2.1.3 Synthesis of 3

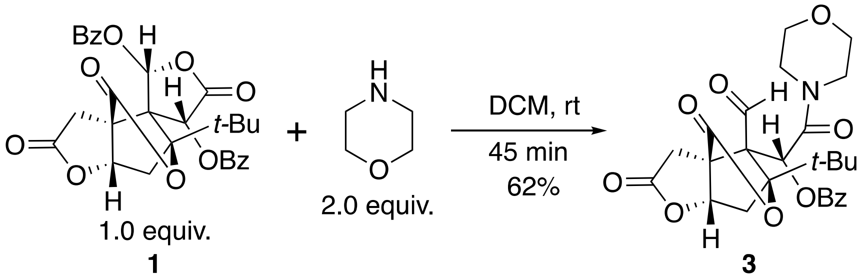

To an oven-dried round bottom flask with a magnetic stirbar was added **1** (0.10 mmol, 53.5 mg, 1.00 equiv.), followed by the addition of anhydrous tetrahydrofuran (2 mL). Morpholine (17 μL, 0.20 mmol, 2.0 equiv.) was then added to the flask. The resulting solution stirred at room temperature for 30 min. Upon completion indicated by TLC, the reaction solution was concentrated *in vacuo*. The residue was dissolved with dichloromethane (5 mL) and the organic layer was washed with brine (5 mL). After separation, the aqueous phase was

extracted two more times with dichloromethane (5 mL). The combined organic layers were dried over Mg_2SO_4, filtered and concentrated *in vacuo*. The crude product was purified via column chromatography to provide the product **BB17** as a white powder.

White powder, 62% yield, 30.9 mg; R_f = 0.43 (hexane: EtOAc = 1:1, UV detection on TLC plate). ^{1}H NMR (700 MHz, $CDCl_3$) δ 11.04 (s, 1H), 7.89 (d, *J* = 8.7 Hz, 2H), 7.62 (t, *J* = 7.2 Hz, 1H), 7.46 (t, *J* = 7.7 Hz, 2H), 6.17 (s, 1H), 4.49 (dd, *J* = 7.7, 4.3 Hz, 1H), 4.28 (d, *J* = 13.0 Hz, 1H), 4.15 (d, *J* = 13.0 Hz, 1H), 3.92 (d, *J* = 12.3 Hz, 1H), 3.87-3.80 (m, 2H), 3.59-3.51 (m, 2H), 3.11 (d, *J* = 18.4 Hz, 1H), 3.07 (dt, *J* = 11.8, 2.5 Hz, 1H), 2.78 (dd, *J* = 8.0, 15.0 Hz, 1H), 2.74 (dd, *J* = 18.4 Hz, 1H), 2.48 (dd, *J* = 4.4, 15.1 Hz, 1H), 1.12 (s, 9H). ^{13}C NMR (175 MHz, $CDCl_3$) δ 198.9, 173.4, 170.0, 165.8, 164.3, 134.6, 130.0, 129.1, 127.5, 103.3, 78.9, 72.8, 66.54, 66.47, 66.3, 63.6, 48.0, 42.7, 36.1, 36.0, 30.7, 27.3. HRMS (ESI): Calcd for $C_{26}H_{29}NO_9$ $[M+Na]^+$: 522.17384, found: 522.17378.

2.1.4 Synthesis of BB16 and BB17

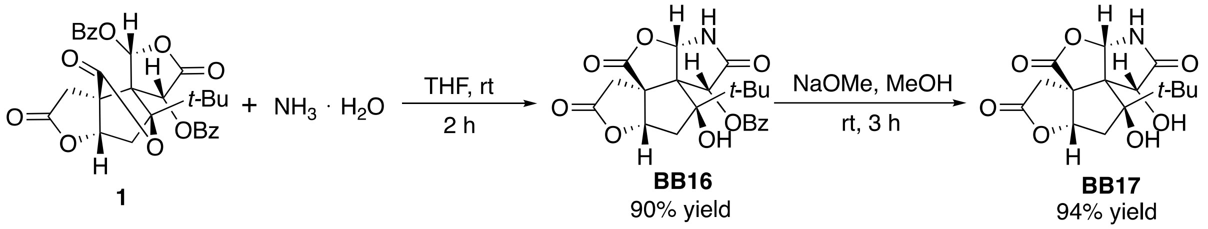

To an oven-dried flask with a magnetic stirbar was added **1** (500 mg, 0.94 mmol, 1.0 equiv.) and THF (10 mL), followed by the addition of 25%~28% ammonia solution (350 µL, 4.68 mmol, 5.0 equiv.). The resulting solution was then allowed to be stirred for 2 h at room temperature. Upon completion monitored by TLC, the reaction solution was dried over anhydrous magnesium sulfate. The reaction solution was filtered and concentrated *in vacuo*. The crude product was purified via column chromatography (hexane: EtOAc = 1:1, v/v) to provide **BB16** (361.5 mg, 0.84 mmol, 90%) as a white powder.

To an oven-dried flask with a magnetic stirbar was added **BB16** (200 mg, 0.46 mmol, 1.0 equiv.) and methanol (5 mL), followed by the addition of methoxide (126 mg, 2.33 mmol, 5.0 equiv.). The resulting mixture was allowed to be stirred at room temperature for 3 h. Once completion indicated by the TLC plate, the pH value of the reaction solution was adjusted to 5.0 with aqueous 2 N HCl solution (~1.2 mL). The mixture was extracted with ethyl acetate (7×10 mL) and the organic layer was washed 3 times with brine. The combined organic layers were dried over anhydrous magnesium sulfate, filtered and concentrated under reduced

pressure. The crude product was purified by column chromatography with elution system (hexane: EtOAc = 1:2, v/v) to give **BB17** (142.8 mg, 0.44 mmol, 94%) as a white powder.

(3a*S*,5a*S*,8*S*,9*R*,10a*S*)-9-(*tert*-Butyl)-9-hydroxy-2,4,7-trioxooctahydro-4*H*,9*H*-furo[3'',2'':2',3']cyclopenta[1',2':3,4]furo[2,3-*b*]pyrrol-8-yl benzoate (BB16)

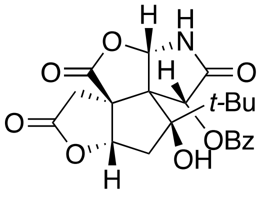

BB16

White powder, 90% yield, 31 mg; R_f = 0.34 (Hexane/EtOAc = 1:1, UV detection on TLC plate); Melting point: 237.3 - 238.5 °C. ^{1}H NMR (500 MHz, Methanol-d_4) δ 8.04 – 7.89 (m, 2H), 7.73 – 7.61 (m, 1H), 7.56 – 7.40 (m, 2H), 6.36 (s, 1H), 6.08 (s, 1H), 5.18 (t, *J* = 7.1 Hz, 1H), 3.18 – 2.94 (m, 2H), 2.72 (dd, *J* = 13.7, 7.2 Hz, 1H), 2.12 (dd, *J* = 13.7, 7.2 Hz, 1H), 1.06 (s, 9H). ^{13}C NMR (126 MHz, Methanol-d_4) δ 179.93, 175.90, 171.03, 166.48, 135.23, 130.93, 130.82, 129.85, 129.45, 128.61, 87.45, 86.39, 85.49, 72.66, 65.87, 60.89, 43.14, 38.39, 37.44, 26.95. HRMS (ESI): Calcd for $C_{22}H_{23}NO_8$ [M+Na]$^+$: 452.13159, found: 452.13155.

(3a*S*,5a*S*,8*S*,9*R*,10a*S*)-9-(*tert*-Butyl)-8,9-dihydroxytetrahydro-4*H*,9*H*-furo[3'',2'':2',3']c-yclopenta[1',2':3,4]furo[2,3-*b*]pyrrole-2,4,7(3*H*,8*H*)-trione (BB17)

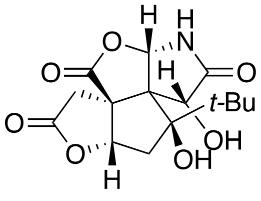

BB17

White powder, 94% yield, 34 mg; R_f = 0.10 (Hexane/EtOAc = 1:1, UV detection after heating over 2 min. on TLC plate); Melting point: 128.4 – 128.9 °C. ^{1}H NMR (500 MHz, DMSO-d_6) δ 9.19 (s, 1H), 6.67 (d, *J* = 4.7 Hz, 1H), 5.75 (s, 1H), 5.18 (s, 1H), 4.89 (t, *J* = 6.9 Hz, 1H), 4.65 (d, *J* = 4.7 Hz, 1H), 2.84 (d, *J* = 17.9 Hz, 1H), 2.70 (d, *J* = 18.0 Hz, 1H), 2.56 – 2.34 (m, 1H), 2.09 (dd, *J* = 13.2, 6.8 Hz, 1H), 1.01 (s, 9H). ^{13}C NMR (126 MHz, DMSO-d_6) δ 178.44, 174.14, 173.59, 85.34, 84.43, 82.96, 69.00, 64.88, 58.96, 41.38, 36.98, 36.40, 26.64 (3C). HRMS (ESI): Calcd for $C_{15}H_{19}NO_7$ [M+Na]$^+$: 348.10537, found: 348.10522.

2.1.5 General procedure for the synthesis of *N*-arylated analogues

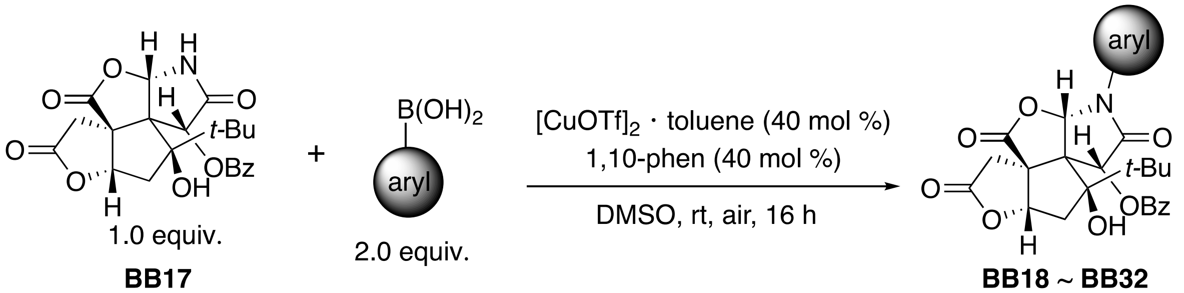

To an oven-dried round-bottom flask containing a magnetic stirbar was added **BB17** (100 mg, 1 equiv., 0.233 mmol) and aryl/hetero aryl boronic acid (2.0 equiv., 0.466 mmol). $[CuOTf]_2$· toluene (48 mg, 0.093 mmol, 40 mol%), 1,10-Phen (17 mg, 0.093 mmol, 40 mol%) and DMSO (10 mL) were then added sequentially. The reaction mixture was stirred at room temperature under open air for 16 h. Once the starting material was fully consumed, the reaction solution was diluted with 20 mL ice cold water and extracted with ethyl acetate (3 x 15 mL). The combined organic layers were washed 3 times with brine, dried over anhydrous Na_2SO_4 and concentrated under reduced pressure. The crude product was purified by column chromatography to provide the desired products **BB18** ~ **BB32**.

(3a*S*,5a*S*,8*S*,9*R*,10a*S*)-9-(*tert*-Butyl)-9-hydroxy-2,4,7-trioxo-6-phenyloctahydro-4*H*,9*H*-furo[3'',2'':2',3']cyclopenta[1',2':3,4]furo[2,3-*b*]pyrrol-8-yl benzoate (BB18)

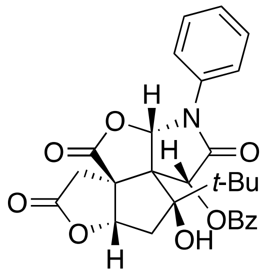

BB18

White powder, 80% yield, 94 mg; R_f = 0.2 (hexane: EtOAc = 1:1, UV detection on TLC plate); Melting point: 125.7 – 126.4 °C. ^{1}H NMR (400 MHz, $CDCl_3$) δ 8.07 – 7.90 (m, 2H), 7.71 – 7.56 (m, 3H), 7.56 – 7.48 (m, 2H), 7.47 – 7.38 (m, 2H), 7.34 – 7.28 (m, 1H), 6.47 (s, 1H), 6.46 (s, 1H), 5.22 (t, *J* = 7.1 Hz, 1H), 3.30 (d, *J* = 18.8 Hz, 1H), 2.95 (d, *J* = 18.9 Hz, 1H), 2.68 (dd, *J* = 14.0, 7.2 Hz, 1H), 2.28 (dd, *J* = 14.0, 7.1 Hz, 1H), 1.13 (s, 9H). ^{13}C NMR (101 MHz, $CDCl_3$) δ 177.75, 173.59, 166.34, 165.24, 135.32, 134.35, 133.85, 130.12, 129.46, 129.37, 128.83, 127.73, 127.32, 127.11, 121.82, 88.05, 87.46, 83.40, 70.99, 61.72, 59.36, 44.47, 42.76, 37.43, 36.27, 26.43. HRMS (ESI): Calcd for $C_{28}H_{27}NO_8$ $[M+Na]^+$: 528.16289, found: 528.16338.

(3a*S*,5a*S*,8*S*,9*R*,10a*S*)-9-(*tert*-Butyl)-8,9-dihydroxy-6-phenyltetrahydro-4*H*,9*H*-furo[3'',2'':2',3']cyclopenta[1',2':3,4]furo[2,3-*b*]pyrrole-2,4,7(3*H*,8*H*)-trione (BB19)

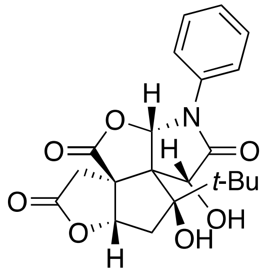

BB19

To an oven-dried flask containing a magnetic stirbar was added **BB18** (50 mg, 1 equiv., 98.90 μmol) and methanol (10 mL) , followed by the addition of potassium carbonate (2.0 equiv., 27.3 mg, 0.198 mmol). The resulting solution was allowed to be stirred at room temperature for 4 h. Once the starting material was fully consumed, the reaction solution was concentrated under reduced pressure and then the residue was resuspended with water. The pH value of the resuspension was adjusted to 7.0 using 3 N HCl aqueous solution. The mixture was extracted 3 times with ethyl acetate and washed 3 times with brine, respectively. The combined organic layers were dried over anhydrous sodium sulfate, filtered and concentrated under reduced pressure. The crude product was purified by column chromatography with elution system (hexane: EtOAc =1:2, v/v) to give **BB19** as white powder. White powder, 72% yield, 28.6 mg; ^{1}H NMR (600 MHz, Methanol-d_4): δ 7.57 (d, *J* = 7.9 Hz, 2H), 7.45 (t, *J* = 7.8 Hz, 2H), 7.32 (t, *J* = 7.4 Hz, 1H), 6.31 (s, 1H), 5.08 (t, *J* = 6.8 Hz, 1H), 5.07 (s, 1H), 3.14 (d, *J* = 17.8 Hz, 1H), 2.80 (d, *J* = 17.9 Hz, 1H), 2.66 (dd, *J* = 7.2, 13.5 Hz, 1H), 2.39 (dd, *J* = 6.8, 13.5 Hz, 1H), 1.17 (s, 9H); ^{13}C NMR (150 MHz, Methanol-d_4): δ 179.6, 176.0, 173.9, 137.0, 130.0, 127.9, 124.2, 89.5, 87.2, 85.0, 70.8, 64.6, 60.5, 42.7, 38.4, 37.7, 27.0. HRMS (ESI): Calcd for $C_{21}H_{23}NO_7$ $[M+Na]^+$: 424.13599, found 424.13667.

(3a*S*,5a*S*,8*S*,9*R*,10a*S*)-6-(4-((*tert*-Butoxycarbonyl)amino)phenyl)-9-(*tert*-butyl)-9-hydroxy-2,4,7-trioxooctahydro-4*H*,9*H*-furo[3'',2'':2',3']cyclopenta[1',2':3,4]furo[2,3-*b*]pyrrol-8-yl benzoate (BB20)

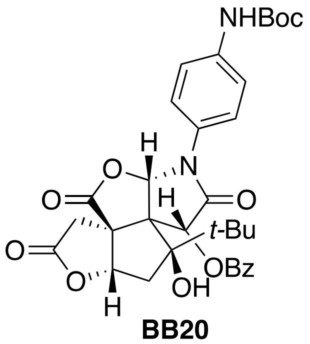

BB20

White powder, 74% yield, 107 mg; R_f = 0.4 (hexane: EtOAc = 3:1, UV detection on TLC plate); Melting point: 131.4 – 132.8 °C. ^{1}H NMR (500 MHz, $CDCl_3$) δ 8.01 (d, *J* = 7.7 Hz, 2H), 7.63 (t, *J* = 7.4 Hz, 1H), 7.56 – 7.46 (m, 4H), 7.40 (d, *J* = 8.6 Hz, 2H), 6.63 (s, 1H), 6.46 (s, 1H), 6.37 (s, 1H), 5.21 (t, *J* = 7.0 Hz, 1H), 3.29 (d, *J* = 18.8 Hz, 1H), 2.95 (d, *J* = 18.8 Hz, 1H), 2.66 (dd, *J* = 14.0, 7.1 Hz, 1H), 2.27 (dd, *J* = 14.0, 7.1 Hz, 1H), 1.52 (s, 9H), 1.12 (s, 9H). ^{13}C NMR (126 MHz, $CDCl_3$) δ 177.60, 173.48, 166.14, 165.24, 152.65, 137.31, 134.30, 130.14, 130.12, 128.80, 127.73, 122.96, 119.17, 88.24, 87.58, 83.27, 70.91, 61.74, 59.33, 42.97, 37.42, 36.31, 28.32, 26.43. HRMS (ESI): Calcd for $C_{33}H_{36}N_2O_{10}$ $[M+Na]^+$: 643.22622, found: 643.22659.

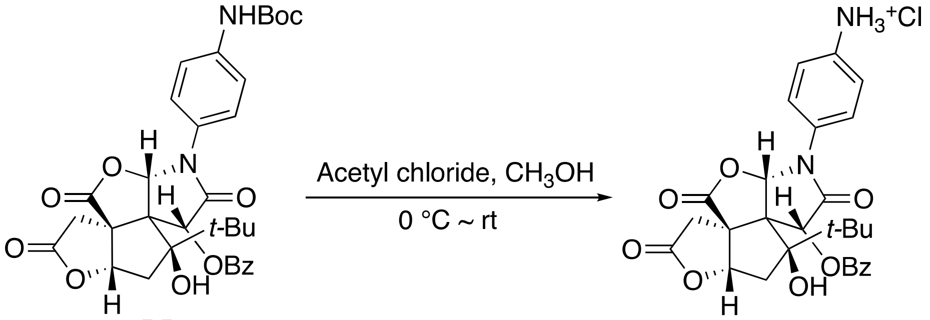

BB20 **BB21**

To an oven-dried flask containing a magnetic stirbar was added methanol (10 mL) and then acetyl chloride (0.35 mL, 5.00 mmol, 15 equiv.) was added dropwise while the flask was cooled in an ice bath. After addition, the reaction solution was stirred at room temperature for 15 minutes to generate a 0.5 N HCl solution. **BB20** (207 mg, 0.33 mmol, 1 equiv.) was added to the HCl solution and the resulting solution was allowed to be stirred for 18 hours at room temperature. Upon completion, the reaction mixture was concentrated under reduced pressure to give a yellow residue. The residue was then suspended in a mixture of hexane/ethyl acetate (v/v = 1:1, 10 mL) and sonicated to reach homogeneity, before being filtered through a sintered funnel. The resultant residue is washed several times with hexane/ethyl acetate (v/v = 1:1) to produce the desired salt **BB21** (158 mg, 85%) as a white solid.

(3a*S*,5a*S*,8*S*,9*R*,10a*S*)-6-(4-Aminophenyl)-9-(*tert*-butyl)-9-hydroxy-2,4,7-trioxooctahydro-4*H*,9*H*-furo[3'',2'':2',3']cyclopenta[1',2':3,4]furo[2,3-*b*]pyrrol-8-yl benzoate hydrochloride (BB21)

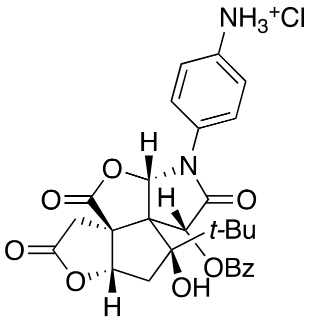

BB21

White powder, 65% yield, 79 mg; R_f = 0.1 (hexane: EtOAc = 1:1, UV detection on TLC plate) Melting point: 115.4 – 116.3 °C. ^{1}H NMR (400 MHz, Methanol-d_4) δ 8.04 – 7.98 (m, 2H), 7.94 – 7.84 (m, 2H), 7.72 – 7.65 (m, 1H), 7.59 – 7.48 (m, 4H), 6.65 (s, 1H), 6.60 (s, 1H), 5.24 (t, *J* = 7.1 Hz, 1H), 5.04 (s, 2H), 3.23 – 3.00 (m, 2H), 2.82 (dd, *J* = 13.8, 7.2 Hz, 1H), 2.22 (dd, *J* = 13.8, 7.2 Hz, 1H), 1.13 (s, 9H). ^{13}C NMR (101 MHz, Methanol-d_4) δ 176.35, 173.07, 165.86, 163.80, 134.82, 132.87, 128.41, 127.73, 127.37, 126.97, 126.65, 126.07, 122.54, 86.56, 85.34, 82.87, 65.49, 61.15, 59.54, 57.99, 41.27, 40.55, 35.91, 34.67, 24.47. HRMS (ESI): Calcd for $C_{28}H_{28}N_2O_8$ [M+Na]$^+$: 543.17379, found: 543.17396.

(3a*S*,5a*S*,8*S*,9*R*,10a*S*)-9-(*tert*-Butyl)-9-hydroxy-6-(4-methoxyphenyl)-2,4,7-trioxooctahydro-4*H*,9*H*-furo[3'',2'':2',3']cyclopenta[1',2':3,4]furo[2,3-*b*]pyrrol-8-yl benzoate (BB22)

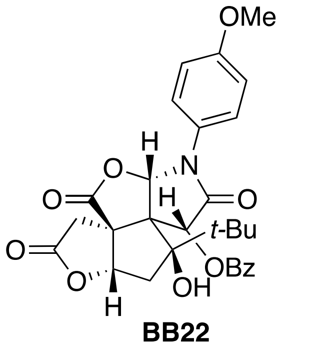

BB22

Using **BB17** (153 mg, 0.35 mmol), $(CuOTf)_2$-toluene (20 mol%, 0.07 mmol, 36.2 mg), yielding **BB22** as a white powder, 68%, 0.13 g; R_f = 0.23 (hexane:EtOAc:DCM = 3:1:1); ^{1}H NMR (600 MHz, $CDCl_3$): δ 8.00 (d, *J* = 7.6 Hz, 2H), 7.62 (t, *J* = 7.4 Hz, 1H), 7.50 (t, *J* = 7.8 Hz, 2H), 7.44 (d, *J* = 9.0 Hz, 2H), 6.90 (d, *J* = 9.0 Hz, 2H), 6.45 (s, 1H), 6.33 (s, 1H), 5.17 (t, *J* = 7.1 Hz, 1H), 3.80 (s, 3H), 3.27 (d, *J* = 18.8 Hz, 1H), 2.94 (d, *J* = 18.8 Hz, 1H), 2.70 (s, 1H), 2.63 (dd, *J* = 7.2, 13.9 Hz, 1H), 2.23 (dd, *J* = 7.1, 13.9 Hz, 1H), 1.08 (s, 9H); ^{13}C NMR (150 MHz,

$CDCl_3$): δ 177.9, 173.7, 166.4, 165.3, 158.7, 134.4, 130.2, 128.9, 127.89, 127.87, 124.5, 114,7, 88.6, 87.6, 83.5, 71.0, 62.0, 59.5, 55.6, 42.9, 37.5, 36.4, 26.5. HRMS (ESI): Calcd for $C_{29}H_{29}NO_9$ $[M+Na]^+$: 558.17345, found 558.17257.

(3a*S*,5a*S*,8*S*,9*R*,10a*S*)-9-(*tert*-Butyl)-9-hydroxy-6-(4-morpholinophenyl)-2,4,7-trioxoocta-hydro-4*H*,9*H*-furo[3'',2'':2',3']cyclopenta[1',2':3,4]furo[2,3-*b*]pyrrol-8-yl benzoate (BB23)

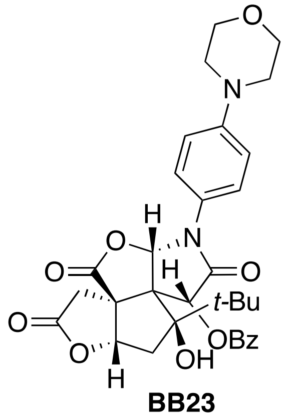

BB23

White powder, 90% yield, 124 mg; R_f = 0.15 (hexane: EtOAc = 2:1, UV detection on TLC plate); Melting point: 160.8 – 161.2 °C. ^{1}H NMR (500 MHz, Methanol-d_4) δ 8.10 – 7.95 (m, 2H), 7.77 – 7.64 (m, 1H), 7.61 – 7.49 (m, 2H), 7.49 – 7.36 (m, 2H), 7.11 – 6.90 (m, 2H), 6.59 (s, 1H), 6.42 (s, 1H), 5.22 (t, *J* = 7.1 Hz, 1H), 3.95 – 3.69 (m, 4H), 3.27 – 3.07 (m, 6H), 2.78 (dd, *J* = 13.7, 7.1 Hz, 1H), 2.30 – 2.12 (m, 1H), 1.15 (s, 9H). ^{13}C NMR (126 MHz, Methanol-d_4) δ 174.24, 170.85, 164.05, 161.99, 148.37, 132.32, 128.20, 127.17, 126.76, 125.41, 123.80, 114.86, 89.30, 87.06, 84.85, 72.62, 68.08, 64.56, 61.27, 51.26, 44.55, 40.14, 38.98, 29.17. HRMS (ESI): Calcd for $C_{32}H_{34}N_2O_9$ $[M+H]^+$: 591.23371, found: 591.23365.

(3a*S*,5a*S*,8*S*,9*R*,10a*S*)-9-(*tert*-Butyl)-8,9-dihydroxy-6-(4-morpholinophenyl)tetrahydro-4 *H*,9*H*-furo[3'',2'':2',3']cyclopenta[1',2':3,4]furo[2,3-*b*]pyrrole-2,4,7(3*H*,8*H*)-trione (BB24)

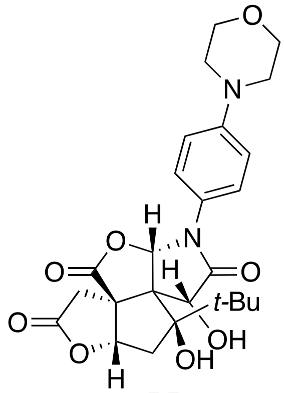

BB24

White powder, 80% yield, 66 mg; R_f = 0.1 (hexane: EtOAc = 1:2, UV detection on TLC plate); Melting point: 175.6 – 176.1 °C. ^{1}H NMR (500 MHz, Methanol-d_4) δ 7.45 – 7.28 (m, 2H), 7.09 – 6.97 (m, 2H), 6.21 (s, 1H), 5.88 (s, 1H), 5.51 (s, 1H), 5.09 (t, *J* = 7.0 Hz, 1H), 5.07 (s, 1H), 3.89 – 3.80 (m, 4H), 3.22 – 3.16 (m, 4H), 3.14 (d, *J* = 18.0 Hz, 1H), 2.81 (d, *J* = 17.9 Hz, 1H), 2.66 (dd, *J* = 13.5, 7.2 Hz, 1H), 2.40 (dd, *J* = 13.5, 6.8 Hz, 1H), 1.18 (s, 9H). ^{13}C NMR (126 MHz, Methanol-d_4) δ 179.97, 176.27, 174.29, 151.94, 128.85, 126.19, 117.03, 90.22, 87.55, 85.26, 70.90, 67.87, 65.03, 60.87, 50.29, 43.03, 38.58, 38.15, 27.36. HRMS (ESI): Calcd for $C_{25}H_{30}N_2O_8$ [M+Na]$^+$: 509.18944, found: 509.18984.

(3a*S*,5a*S*,8*S*,9*R*,10a*S*)-6-(4-Acetamidophenyl)-9-(*tert*-butyl)-9-hydroxy-2,4,7-trioxoocta-hydro-4*H*,9*H*-furo[3'',2'':2',3']cyclopenta[1',2':3,4]furo[2,3-*b*]pyrrol-8-yl benzoate (BB25)

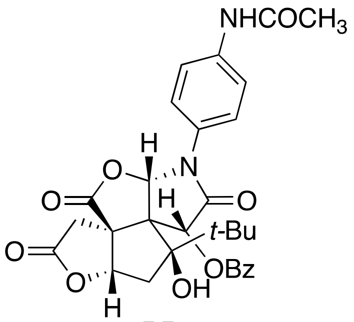

BB25

White powder, 70% yield, 92 mg; R_f = 0.3 (hexane: EtOAc = 2:1, UV detection on TLC plate); Melting point: 185.7 – 186.2 °C. ^{1}H NMR (500 MHz, DMSO-d_6) δ 10.09 (s, 1H), 8.02 – 7.90 (m, 2H), 7.78 – 7.72 (m, 1H), 7.72 – 7.63 (m, 2H), 7.60 – 7.51 (m, 4H), 6.49 (s, 1H), 6.47 (s, 1H), 5.12 (t, *J* = 7.1 Hz, 1H), 3.28 (d, *J* = 19.2 Hz, 1H), 2.98 (d, *J* = 19.2 Hz, 1H), 2.73 (dd, *J* = 13.6, 7.2 Hz, 1H), 2.11 – 2.01 (m, 4H), 1.07 (s, 9H). ^{13}C NMR (126 MHz, DMSO-d_6) δ 177.87, 174.21, 168.90, 166.61, 164.96, 138.63, 134.93, 130.19, 129.95, 129.45, 128.24, 124.89, 119.70, 88.17, 86.66, 83.87, 71.78, 62.89, 59.31, 42.10, 40.85, 37.61, 36.11, 26.82, 24.45. HRMS (ESI): Calcd for $C_{30}H_{30}N_2O_9$ [M+Na]$^+$: 585.18435, found: 585.18462.

(3a*S*,5a*S*,8*S*,9*R*,10a*S*)-6-(Benzo[*d*][1,3]dioxol-5-yl)-9-(*tert*-butyl)-9-hydroxy-2,4,7-trioxo-octahydro-4*H*,9*H*-furo[3'',2'':2',3']cyclopenta[1',2':3,4]furo[2,3-*b*]pyrrol-8-yl benzoate (BB26)

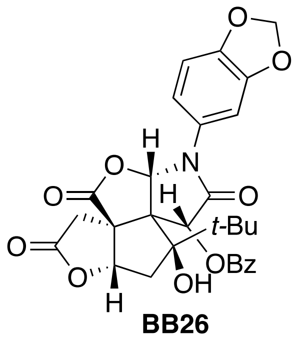

BB26

White powder, 85% yield, 128 mg; R_f = 0.2 (hexane: EtOAc = 2:1, UV detection on TLC plate); Melting point: 156.2 – 156.9 °C. ^{1}H NMR (400 MHz, $CDCl_3$) δ 8.08 – 7.90 (m, 2H), 7.70 – 7.59 (m, 1H), 7.51 (t, *J* = 7.8 Hz, 2H), 7.09 (d, *J* = 2.2 Hz, 1H), 6.97 (dd, *J* = 8.4, 2.2 Hz, 1H), 6.81 (d, *J* = 8.4 Hz, 1H), 6.45 (s, 1H), 6.31 (s, 1H), 6.00 (s, 2H), 5.21 (t, *J* = 7.1 Hz, 1H), 3.28 (d, *J* = 18.9 Hz, 1H), 2.96 (d, *J* = 18.9 Hz, 1H), 2.67 (dd, *J* = 14.0, 7.2 Hz, 1H), 2.37 – 2.17 (m, 1H), 1.13 (s, 9H). ^{13}C NMR (101 MHz, $CDCl_3$) δ 177.84, 173.64, 166.38, 165.25, 148.23, 146.81, 134.35, 130.12, 128.83, 128.73, 127.74, 116.70, 108.39, 104.90, 101.84, 88.62, 87.42, 83.41, 70.90, 61.96, 59.35, 42.76, 37.41, 36.26, 26.42. HRMS (ESI): Calcd for $C_{29}H_{27}NO_{10}$ $[M+Na]^+$: 572.15272, found: 572.15255.

(3a*S*,5a*S*,8*S*,9*R*,10a*S*)-9-(*tert*-Butyl)-9-hydroxy-2,4,7-trioxo-6-(pyridin-3-yl)octahydro-4*H*,9*H*-furo[3'',2'':2',3']cyclopenta[1',2':3,4]furo[2,3-*b*]pyrrol-8-yl benzoate (BB27)

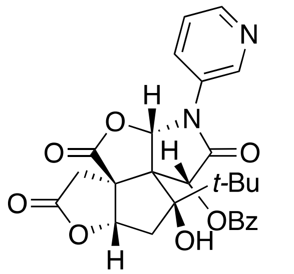

BB27

White powder, 78% yield, 92 mg; R_f = 0.2 (hexane: EtOAc = 1:1, UV detection on TLC plate) Melting point: 269.4 – 270.1 °C. ^{1}H NMR (400 MHz, DMSO-d_6) δ 8.90 (d, *J* = 2.6 Hz, 1H), 8.53 (dd, *J* = 4.7, 1.5 Hz, 1H), 8.10 (ddd, *J* = 8.4, 2.7, 1.5 Hz, 1H), 8.03 – 7.84 (m, 2H), 7.85 – 7.64 (m, 1H), 7.65 – 7.44 (m, 3H), 6.60 (s, 1H), 6.53 (s, 1H), 5.12 (t, *J* = 7.1 Hz, 1H), 3.32 (d, *J* = 19.4 Hz, 1H), 2.96 (d, *J* = 19.3 Hz, 1H), 2.74 (dd, *J* = 13.6, 7.2 Hz, 1H), 2.08 – 1.95 (dd, *J* = 13.6, 7.2 Hz, 1H), 1.07 (s, 9H). ^{13}C NMR (101 MHz, DMSO-d_6) δ 177.72, 174.21, 167.00, 164.90, 148.00, 144.92, 134.96, 132.59, 131.06, 129.97, 129.45, 128.17, 124.25, 87.56, 86.75, 83.87, 71.79, 62.90, 59.28, 42.08, 37.61, 36.13, 31.43, 31.12, 26.81, 22.54. HRMS (ESI): Calcd for $C_{27}H_{26}N_2O_8$ $[M+H]^+$: 507.17619, found: 507.17663.

(3a*S*,5a*S*,8*S*,9*R*,10a*S*)-9-(*tert*-Butyl)-9-hydroxy-2,4,7-trioxo-6-(3-(trifluoromethyl)pheny-l)octahydro-4*H*,9*H*-furo[3'',2'':2',3']cyclopenta[1',2':3,4]furo[2,3-*b*]pyrrol-8-yl benzoate (BB28)

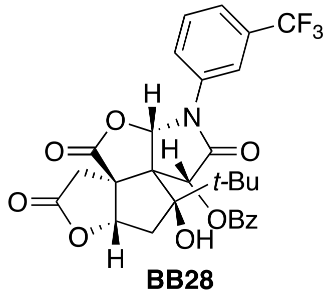

BB28

Using **BB17** (153 mg, 0.35 mmol), $(CuOTf)_2$-toluene (20 mol%, 0.07 mmol, 36.2 mg), yielding **BB28** as a White powder, 69% yield, 0.20 g; R_f = 0.29 (hexane:EtOAc:DCM = 3:1:1). ^{1}H NMR (600 MHz, $CDCl_3$): δ 7.99-8.04 (m, 3H), 7.90 (m, 1H), 7.65 (t, *J* = 7.4 Hz, 1H), 7.55 (d, *J* = 4.9 Hz, 2H), 7.51 (t, *J* = 8.0 Hz, 2H), 6.50 (s, 1H), 6.48 (s, 1H), 5.22 (t, *J* = 7.1 Hz, 1H), 3.28 (d, *J* = 18.8 Hz, 1H), 2.96 (d, *J* = 18.8 Hz, 1H), 2.69 (dd, *J* = 7.2, 14.1 Hz, 1H), 2.50 (s, 1H), 2.30 (dd, *J* = 7.1, 14.0 Hz, 1H), 1.14 (s, 9H); ^{13}C NMR (150 MHz, $CDCl_3$): δ 177.5, 173.3, 166.6, 165.3, 136.3, 134.6, 132.0 (d, *J* = 32.0 Hz), 130.3, 130.1, 129.0, 127.6, 124.6, 124.2, 123.6 (m), 118.0 (m), 87.9, 87.7, 83.3, 61.6, 59.4, 43.2, 37.6, 36.4, 26.6. HRMS (ESI): Calcd for $C_{29}H_{26}F_3NO_8$ $[M+Na]^+$: 596.15027, found 596.1491.

(3a*S*,5a*S*,8*S*,9*R*,10a*S*)-9-(*tert*-Butyl)-9-hydroxy-2,4,7-trioxo-6-(*o*-tolyl)octahydro-4*H*,9*H*-furo[3'',2'':2',3']cyclopenta[1',2':3,4]furo[2,3-*b*]pyrrol-8-yl benzoate (BB29)

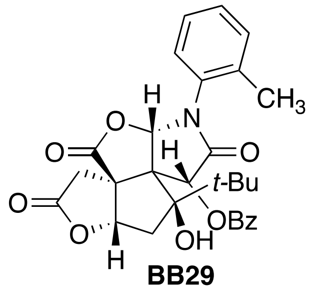

BB29

Using **BB17** (150.0 mg, 0.35 mmol), $(CuOTf)_2$-toluene (20 mol%, 0.07 mmol, 36.1 mg), yielding **BB29** as a white powder, 40% yield, 71.2 mg. ^{1}H NMR (600 MHz, $CDCl_3$) δ 7.98 (d, *J* = 7.7 Hz, 2H), 7.62 (t, *J* = 7.2 Hz, 1H), 7.49 (t, *J* = 7.5 Hz, 2H), 7.21-7.33 (m, 3H) 7.06 (d, *J* = 7.7 Hz, 1H), 6.50 (s, 1H), 6.14 (s, 1H), 5.15 (t, *J* = 7.0 Hz, 1H), 3.36 (d, *J* = 18.7 Hz, 1H), 3.02 (d, *J* = 18.7 Hz, 1H), 2.57-2.66 (m, 2H), 2.16-2.24 (m, 4H), 1.08 (s, 9H); ^{13}C NMR (150 MHz, $CDCl_3$) δ 178.2, 173.6, 166.5, 165.3, 134.4, 132.8, 131.6, 130.2, 129.7, 128.9, 127.9, 127.3, 89.6, 87.5, 83.6, 70.8, 62.9, 59.6, 43.0, 37.5, 36.8, 26.5, 18.3. HRMS (ESI): Calcd for $C_{29}H_{29}NO_8$ $[M+Na]^+$: 542.17854, found: 542.17737.

(3a*S*,5a*S*,8*S*,9*R*,10a*S*)-9-(*tert*-Butyl)-6-(4-cyanophenyl)-9-hydroxy-2,4,7-trioxooctahydro-4*H*,9*H*-furo[3'',2'':2',3']cyclopenta[1',2':3,4]furo[2,3-*b*]pyrrol-8-yl benzoate (BB30)

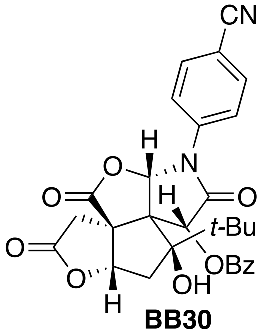

BB30

Using **BB17** (153 mg, 0.35 mmol), $(CuOTf)_2$-toluene (20 mol%, 0.07 mmol, 36.2 mg), yielding **BB30** as a white powder, 63%, 0.12 g. R_f = 0.20 (hexane:EtOAc:DCM = 3:1:1). ^{1}H NMR (600 MHz, $CDCl_3$) δ 8.02 (d, *J* = 8.5 Hz, 2H), 7.93 (d, *J* = 8.7 Hz, 2H), 7.72 (d, *J* = 8.7 Hz, 2H), 7.66 (t, *J* = 7.1 Hz, 1H), 7.53 (t, *J* = 7.7 Hz, 2H), 6.48 (s, 1H), 6.46 (s, 1H), 5.27 (t, *J* = 7.1 Hz, 1H), 3.25 (d, *J* = 18.8 Hz, 1H), 2.94 (d, *J* = 18.8 Hz, 1H), 2.69 (dd, *J* = 7.1, 14.3 Hz, 1H), 2.35 (dd, *J* = 7.2, 14.2 Hz, 1H), 2.03 (s, 1H), 1.16 (s, 9H);); ^{13}C NMR (150 MHz, $CDCl_3$) δ 176.6, 172.8, 166.0, 165.0, 139.6, 134.5, 133.5, 130.1, 128.9, 127.9, 120.6, 118.1, 109.8, 87.9, 87.0, 82.9, 70.7, 61.0, 59.0, 43.3, 37.5, 36.2, 26.4. HRMS (ESI): Calcd for $C_{29}H_{26}N_2O_8$ $[M+Na]^+$: 553.15814, found 553.15728.

(3a*S*,5a*S*,8*S*,9*R*,10a*S*)-9-(*tert*-Butyl)-9-hydroxy-6-(naphthalen-2-yl)-2,4,7-trioxooctahydro-4*H*,9*H*-furo[3'',2'':2',3']cyclopenta[1',2':3,4]furo[2,3-*b*]pyrrol-8-yl benzoate (BB31)

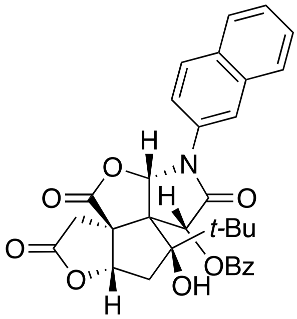

BB31

Using **BB17** (153 mg, 0.35 mmol), $(CuOTf)_2$-toluene (20 mol%, 0.07 mmol, 36.2 mg), yielding **BB31** as a white powder, 74% yield, 0.14 g; R_f = 0.23 (hexane:EtOAc:DCM = 3:1:1). ^{1}H NMR (600 MHz, $CDCl_3$): δ 8.10 (s, 1H), 8.03 (d, *J* = 8.0 Hz, 2H), 7.84 (d, *J* = 8.9 Hz, 1H), 7.80 (dd, *J* = 4.9, 9.9 Hz, 2H), 7.74 (dd, *J* = 2.0, 8.9 Hz, 1H), 7.64 (t, *J* = 7.4 Hz, 1H), 7.52 (t, *J* = 7.7 Hz, 2H), 7.45-7.49 (m, 2H), 6.55 (s, 1H), 6.51 (s, 1H), 5.20 (t, *J* = 7.1 Hz, 1H), 3.35 (d, *J* = 18.8 Hz, 1H), 2.99 (d, *J* = 18.8 Hz, 1H), 2.75 (s, 1H), 2.63 (dd, *J* = 7.2, 14.0 Hz, 1H), 2.24 (dd, *J* = 7.1, 13.9 Hz, 1H), 1.08 (s, 9H); ^{13}C NMR (150 MHz, $CDCl_3$): δ 177.9, 173.7, 166.6, 165.4,

134.5, 133.4, 132.8, 131.9, 130.2, 129.4, 128.9, 128.1, 127.8, 127.7, 127.0, 126.6, 120.5, 120.2, 88.4, 87.6, 83.5, 71.1, 61.9, 59.5, 42.9, 37.5, 36.4, 26.5. HRMS (ESI): Calcd for $C_{32}H_{29}NO_8$ $[M+Na]^+$: 578.17854, found 578.17722.

(3a*S*,5a*S*,8*S*,9*R*,10a*S*)-9-(*tert*-Butyl)-6-(3,5-difluorophenyl)-9-hydroxy-2,4,7-trioxooctahydro-4*H*,9*H*-furo[3'',2'':2',3']cyclopenta[1',2':3,4]furo[2,3-*b*]pyrrol-8-yl benzoate (BB32)

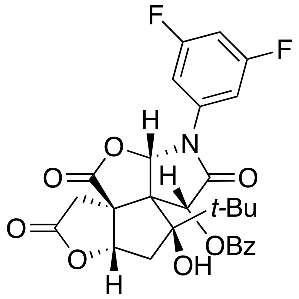

BB32

Using **BB17** (50 mg, 0.12 mmol), $(CuOTf)_2$-toluene (50 mol%, 0.06 mmol, 30.1 mg), 1,10-phenanthroline (50 mol%, 0.06 mmol, 10.5 mg), yielding **BB32** as a white powder, 42% yield, 26.7 mg; ^{1}H NMR (600 MHz, Methanol-d_4): δ 8.00 (d, J = 7.3 Hz, 2H), 7.69 (t, J = 7.4 Hz, 1H), 7.52 (t, J = 7.8 Hz, 2H), 7.47 (d, J = 6.9 Hz, 2H), 6.90 (t, J = 8.9 Hz, 1H), 6.59 (s, 1H), 6.56 (s, 1H), 5.21 (t, J = 7.1 Hz, 1H), 3.06-3.16 (m, 2H), 2.77 (dd, J = 7.1, 13.4 Hz, 1H), 2.20 (dd, J = 7.1, 13.7 Hz, 1H), 1.12 (s, 9H). ^{13}C NMR (150 MHz, Methanol-d_4): δ 178.8, 175.6, 168.5 166.3, 165.3 (d, J = 14.5 Hz), 163.7 (d, J = 14.4 Hz), 135.4, 131.0, 129.9, 129.3, 106.0 (d, J = 30.5 Hz), 102.6 (t, J = 26.3 Hz), 88.9, 88.0, 85.4, 72.6, 63.4, 60.5, 43.1, 38.5, 37.2, 27.0. HRMS (ESI): Calcd for $C_{28}H_{25}F_2NO_8$ $[M+Na]^+$: 564.14404, found 564.14307.

2.1.6 Synthesis of 4 and 5

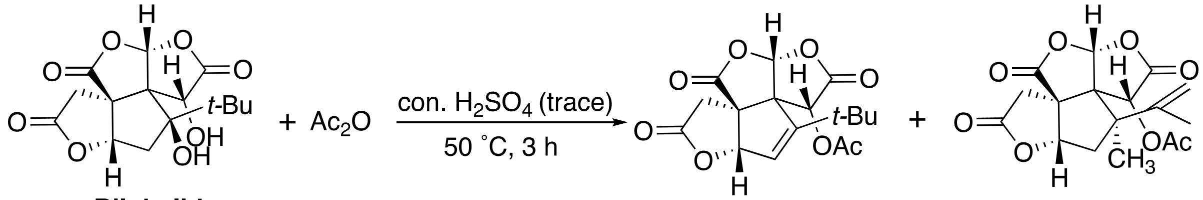

To an oven-dried flask containing a magnetic stirbar was added bilobalide (1.0 g, 3.065 mmol) and acetic anhydride (20 mL), followed by the addition of a trace of concentrated sulfuric acid (20 μL). The resulting solution was heated to 50 °C and allowed to be stirred for 3 h. Once the starting material was fully consumed, the reaction solution was quenched with saturated sodium bicarbonate solution. The mixture was extracted with ethyl acetate (3 x 50 mL) and the combined organic layers were washed three times with brine, dried over anhydrous

sodium sulfate, filtered and then concentrated under reduced pressure. The crude product was purified by column chromatography with elution system (hexane:EtOAc = 8:1, v/v) to yield **4** and **5**.

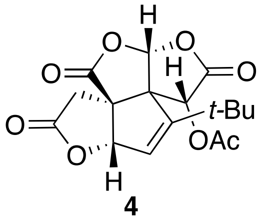

4

White powder, 54% yield, 0.58 g; R_f = 0.40 (Hexane/EtOAc = 2:1, UV detection after heating over 2 min. on TLC plate); Melting point: 127.1 – 128.0 °C. ^{1}H NMR (500 MHz, $CDCl_3$) δ 6.57 (s, 1H), 6.42 (s, 1H), 6.16 (d, *J* = 2.8 Hz, 1H), 5.24 (d, *J* = 2.8 Hz, 1H), 3.10 (d, *J* = 17.9 Hz, 1H), 2.97 (d, *J* = 17.9 Hz, 1H), 2.17 (s, 3H), 1.32 (s, 9H). ^{13}C NMR (126 MHz, $CDCl_3$) δ 174.64, 171.95, 168.84, 166.64, 156.21, 129.16, 99.29, 86.09, 68.91, 67.09, 58.35, 37.14, 35.05, 31.36, 19.90. HRMS (ESI): Calcd for $C_{17}H_{18}O_8$ $[M+Na]^+$: 373.08939, found: 373.08930.

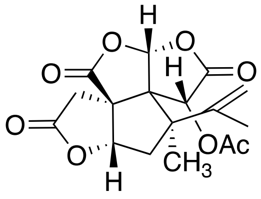

5

White powder, 42% yield, 0.45 g; R_f = 0.35 (Hexane/EtOAc = 2:1, UV detection after heating over 2 min. on TLC plate); Melting point: 170.5 – 171.2 °C. ^{1}H NMR (500 MHz, $CDCl_3$) δ 6.06 (s, 1H), 6.02 (s, 1H), 5.16 (q, *J* = 1.4 Hz, 1H), 5.00 (s, 1H), 4.96 (dd, *J* = 5.6, 1.4 Hz, 1H), 3.21 (d, *J* = 17.7 Hz, 1H), 3.07 (d, *J* = 17.7 Hz, 1H), 2.42 (dd, *J* = 15.4, 5.6 Hz, 1H), 2.26 – 2.17 (m, 4H), 1.88 (d, *J* = 1.4 Hz, 3H), 1.34 (s, 3H). ^{13}C NMR (126 MHz, $CDCl_3$) δ 174.22, 172.60, 168.48, 167.19, 145.20, 115.93, 115.75, 101.10, 86.90, 69.23, 63.53, 61.57, 52.82, 41.21, 37.63, 24.49, 21.63, 20.44. HRMS (ESI): Calcd for $C_{17}H_{18}O_8$ $[M+Na]^+$: 373.08939, found: 373.08945.

2.1.7 Synthesis of BB33

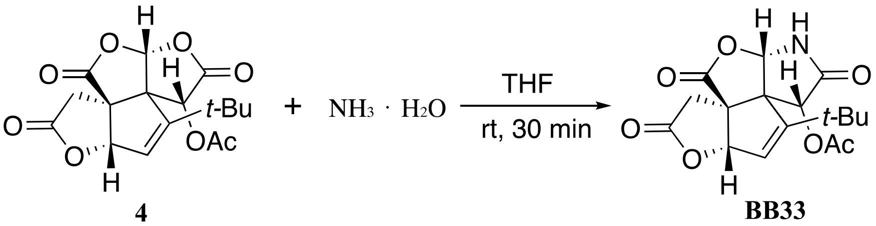

4 **BB33**

To an oven-dried flask containing a magnetic stirbar was added **5** (50 mg, 0.143 mmol, 1.0 equiv.) and anhydrous tetrahydrofuran (10 mL), followed by the addition of 25%~28% ammonia solution (11 μL, 0.286 mmol, 2.0 equiv.) at 0 °C. The resulting solution was then allowed to be stirred for 30 min at room temperature. The reaction was monitored by TLC and upon completion, the reaction solution was concentrated under reduced pressure. The residue was suspended with ethyl acetate (50 mL) and the organic layer was washed with brine, dried over anhydrous Na_2SO_4, filtered and concentrated *in vacuo*. The crude product was purified via column chromatography (hexane: EtOAc = 1:1, v/v) to provide **BB33** as a white powder.

(3a*S*,5a*S*,8*S*,10a*S*)-9-(*tert*-Butyl)-2,4,7-trioxo-2,3,5a,6,7,8-hexahydro-4*H*,10a*H*-furo[3'',2'':2',3']cyclopenta[1',2':3,4]furo[2,3-*b*]pyrrol-8-yl acetate (BB33)

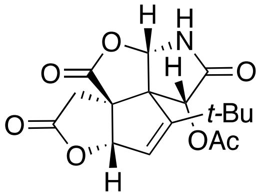

BB33

White powder, 76% yield, 38 mg; R_f = 0.15 (Hexane/EtOAc = 1:1, UV detection after heating over 2 min. on TLC plate); Melting point: 216.2 – 217.1 °C. ^{1}H NMR (500 MHz, $CDCl_3$) δ 7.52 (s, 1H), 6.13 (d, *J* = 2.7 Hz, 1H), 6.08 (s, 1H), 5.24 (d, *J* = 2.7 Hz, 1H), 5.01 (s, 1H), 3.09 – 2.90 (m, 2H), 1.74 (s, 3H), 1.31 (s, 9H). ^{13}C NMR (126 MHz, $CDCl_3$) δ 176.89, 174.61, 174.57, 157.97, 127.58, 87.03, 85.60, 70.87, 67.83, 59.52, 37.82, 34.90, 31.51, 30.01. HRMS (ESI): Calcd for $C_{17}H_{19}NO_7$ $[M+Na]^+$: 372.10537, found: 372.10513.

2.1.8 Synthesis of BB34

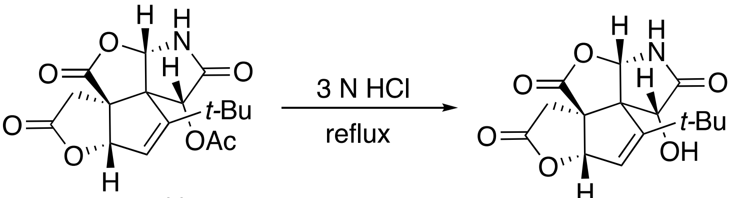

To an oven-dried flask containing a magnetic stirbar was added **BB33** (100 mg, 0.286 mmol) and 3 N aqueous HCl solution (10 mL), respectively. The resulting solution was allowed to be stirred under reflux condition for 12 h. Once the starting material was fully consumed, the reaction solution was cooled down to room temperature and then the pH value was adjusted to 7.0 using saturated sodium bicarbonate solution. The mixture was extracted with ethyl acetate (3 x 50 mL) and the combined organic layers were washed with brine, dried over anhydrous sodium sulfate and concentrated under reduced pressure. The crude product was

purified by column chromatography with elution system (hexane:EtOAc = 1:2, v/v) to give **BB34** as white powder.

(3a*S*,5a*S*,8*S*,10a*S*)-9-(*tert*-Butyl)-8-hydroxy-5a,6-dihydro-4*H*,10a*H*-furo[3'',2'':2',3']cyclopenta[1',2':3,4]furo[2,3-*b*]pyrrole-2,4,7(3*H*,8*H*)-trione (BB34)

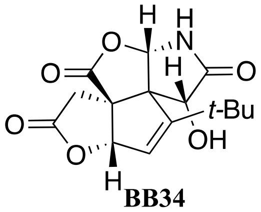

BB34

White powder, 70% yield, 62 mg; R_f = 0.1 (Hexane/EtOAc = 1:2, UV detection after heating over 2 min. on TLC plate); Melting point: 230.4 – 231.0 °C. ^{1}H NMR (500 MHz, CD_3OD) δ 6.19 (s, 1H), 6.18 (d, *J* = 2.8 Hz, 1H), 5.23 (s, 1H), 5.14 (d, *J* = 2.8 Hz, 1H), 2.86 (d, *J* = 17.5 Hz, 1H), 2.77 (d, *J* = 17.5 Hz, 1H), 1.31 (s, 9H). ^{13}C NMR (126 MHz, CD_3OD) δ 179.54, 176.67, 175.39, 159.30, 128.89, 88.00, 87.59, 71.41, 69.09, 60.94, 37.61, 36.10, 31.87. HRMS (ESI): Calcd for $C_{15}H_{17}NO_6$ $[M+Na]^+$: 330.09445, found: 330.09451.

2.1.9 Synthesis of BB35

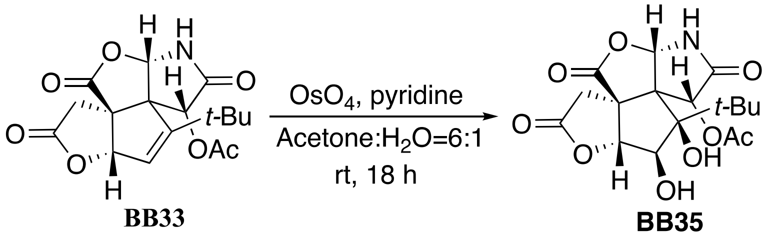

To an oven-dried flask containing a magnetic stirbar was added **BB33** (200 mg, 0.573 mmol, 1.0 equiv.) and 14 mL of acetone/H_2O (v/v=6:1), followed by the addition of pyridine (1 mL) and osmium (VIII) oxide (241 mg, 0.630 mmol, 1.1 equiv.). The resulting solution was allowed to be stirred at room temperature for 18 h. Once completion indicated by TLC, acetone was removed under reduced pressure and the resultant was diluted with ethyl acetate and washed 3 times with 10% aqueous sodium sulfite solution and brine, respectively. The combined organic layers was dried over anhydrous sodium sulfate, filtered and concentrated under reduced pressure. The crude was purified by column chromatography with elution system (hexane:EtOAc=1:2, v/v) to give **BB35** as white powder.

(3a*S*,5a*S*,8*S*,9*R*,10*S*,10a*R*)-9-(*tert*-Butyl)-9,10-dihydroxy-2,4,7-trioxooctahydro-4*H*,9*H*-furo[3'',2'':2',3']cyclopenta[1',2':3,4]furo[2,3-*b*]pyrrol-8-yl acetate (BB35)

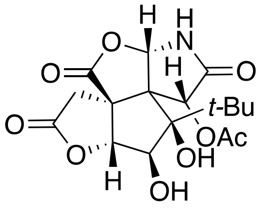

BB35

White powder, 86% yield, 189 mg; R_f = 0.1 (Hexane/EtOAc = 1:2, UV detection after heating over 2 min. on TLC plate); Melting point: 201.2 – 201.9 °C. ^{1}H NMR (500 MHz, Methanol-*d*$_4$) δ 9.37 (s, 1H), 6.11 (s, 1H), 6.07 (s, 1H), 5.37 (s, 1H), 4.83 (d, *J* = 5.0 Hz, 1H), 4.62 (s, 1H), 4.23 (d, *J* = 5.0 Hz, 1H), 2.90 (q, *J* = 18.2 Hz, 2H), 2.14 (s, 3H), 1.16 (s, 9H). ^{13}C NMR (126 MHz, Methanol-*d*$_4$) δ 177.57, 174.17, 171.62, 169.85, 89.84, 84.64, 84.21, 81.34, 70.33, 62.52, 55.61, 53.42, 37.13, 36.73, 25.76, 19.47. HRMS (ESI): Calcd for $C_{17}H_{21}NO_9$ [M+Na]$^+$: 406.11085, found: 406.11079.

2.1.10 Synthesis of BB36

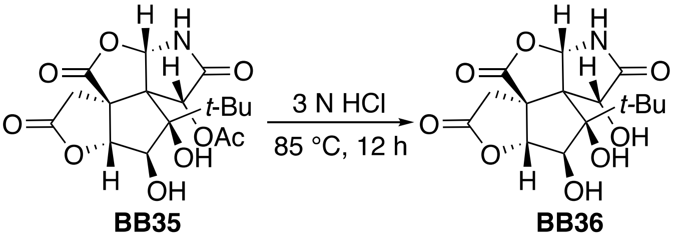

To an oven-dried flask containing a magnetic stirbar was added **BB35** (100 mg, 0.261 mmol, 1.0 equiv.) and 3 N HCl in H_2O (20 mL), respectively. The resulting solution was allowed to be stirred at 85 °C for 12 h. Once the starting material was fully consumed, the reaction solution was cooled down to room temperature and then the pH value was adjusted to 7.0 using saturated sodium bicarbonate solution. The mixture was extracted with ethyl acetate tate (3 x 50 mL) and the combined organic layers were washed with brine, dried over anhydrous sodium sulfate, filtered and concentrated under reduced pressure. The crude was purified by column chromatography with elution system (dichloromethane:MeOH=30:1, v/v) to give **BB36** as white powder.

(3a*S*,5a*S*,8*S*,9*R*,10*S*,10a*R*)-9-(*tert*-Butyl)-8,9,10-trihydroxytetrahydro-4*H*,9*H*-furo[3'',2'':2',3']cyclopenta[1',2':3,4]furo[2,3-*b*]pyrrole-2,4,7(3*H*,8*H*)-trione (BB36)

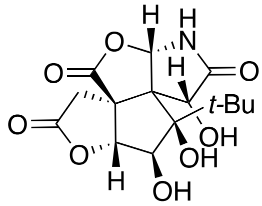

BB36

White powder, 86% yield, 71 mg; R_f = 0.2 (DCM/MeOH = 30:1, UV detection after heating over 2 min. on TLC plate); Melting point: 206.7 – 207.5 ºC. ^{1}H NMR (400 MHz, Acetone-d_6) δ 8.29 (s, 1H), 6.02 (s, 1H), 5.90 (d, J = 3.6 Hz, 1H), 5.30 (d, J = 5.9 Hz, 1H), 4.97 (d, J = 3.7 Hz, 1H), 4.71 (d, J = 5.1 Hz, 1H), 4.62 (t, J = 5.5 Hz, 1H), 4.31 (s, 1H), 3.05 (d, J = 17.9 Hz, 1H), 2.63 (d, J = 17.9 Hz, 1H), 1.25 (s, 9H). ^{13}C NMR (101 MHz, Acetone-d_6) δ 178.21, 173.67, 173.31, 89.65, 84.40, 81.18, 69.75, 63.66, 55.06, 37.21, 36.86, 26.35. HRMS (ESI): Calcd for $C_{15}H_{19}NO_8$ [M+Na]$^+$: 364.10029, found: 364.09999.

2.2 X-ray Crystallographic Data

Figure S1 X-Ray Crystallographic Data for 1 (CCDC: 2267683)

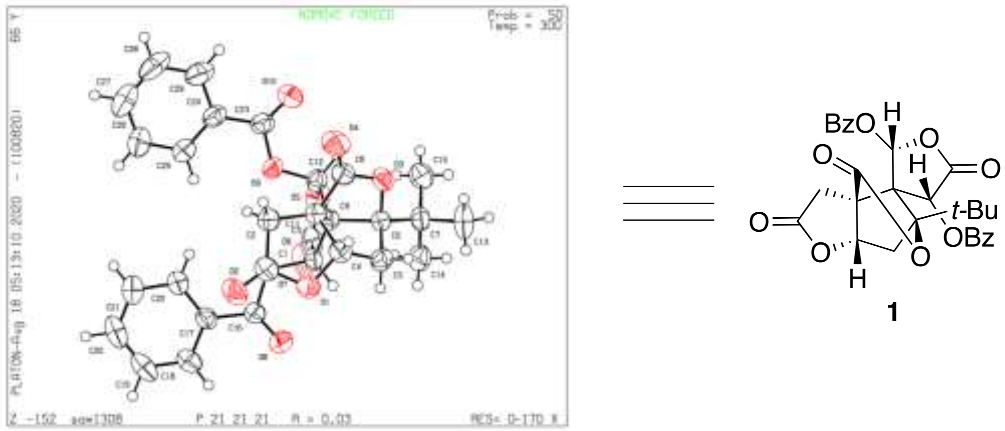

1

Bond precision:	C-C = 0.0032 A		Wavelength=0.71073
Cell:	a=8.4654(3)	b=11.8950	(4) c=26.1665(10)
	alpha=90	beta=90	gamma=90
Temperature:	300 K		
	Calculated		Reported
Volume	2634.86(16)		2634.86(16)
Space group	P 21 21 21		P 2ac 2ab
Hall group	P 21 21 21		P 2ac 2ab
Moiety formula	C29 H26 O10		C29 H26 O10
Sum formula	C29 H26 O10		C29 H26 O10
Mr	534.50		534.50
Dx,g cm-3	1.347		1.347
Z	4		4
Mu (mm-1)	0.102		0.102
F000	1120.0		1120.0
F000’	1120.68		
h,k,lmax	11,15,34		11,15,34
Nref	6303[3568]		6269
Tmin,Tmax	0.970,0.980		0.699,0.746
Tmin’	0.970		

Correction method= # Reported T Limits: Tmin=0.699 Tmax=0.746

AbsCorr = MULTI-SCAN

Data completeness= 1.76/0.99 Theta(max)= 27.903

R(reflections)= 0.0333(5503) wR2(reflections)= 0.0858(6269)

S = 1.050 Npar= 356

Empirical formula	$C_{29}H_{26}O_{10}$
Formula weight	534.50
Temperature/K	300.0
Crystal system	orthorhombic
Space group	$P2_12_12_1$
a/Å	8.4654(3)
b/Å	11.8950(4)
c/Å	26.1665(10)
α/°	90
β/°	90
γ/°	90
Volume/Å^3	2634.86(16)
Z	4
ρ_{calc}g/cm^3	1.347
μ/mm^{-1}	0.102
F(000)	1120.0
Crystal size/mm^3	0.3 × 0.3 × 0.2
Radiation	MoKα (λ = 0.71073)
2Θ range for data collection/°	5.792 to 55.806
Index ranges	$-11 \le h \le 11$, $-13 \le k \le 15$, $-34 \le l \le 34$
Reflections collected	42747
Independent reflections	6269 [R_{int} = 0.0280, R_{sigma} = 0.0171]
Data/restraints/parameters	6269/0/356
Goodness-of-fit on F^2	1.050
Final R indexes [I>=2σ (I)]	R_1 = 0.0333, wR_2 = 0.0784
Final R indexes [all data]	R_1 = 0.0425, wR_2 = 0.0858
Largest diff. peak/hole / e Å^{-3}	0.18/-0.14
Flack parameter	0.15(17)

Figure S2 X-Ray Crystallographic Data for 2 (CCDC: 2267685)

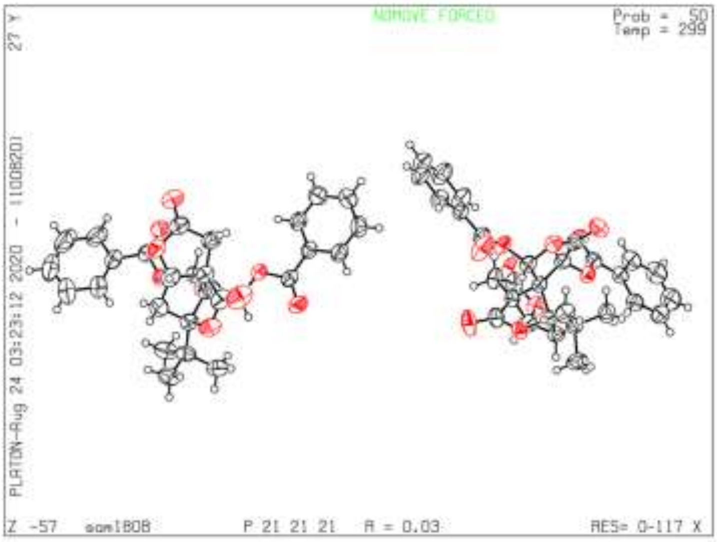

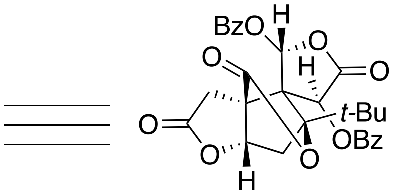

2

Bond precision:	C-C = 0.0039 A		Wavelength=0.71073
Cell:	a=9.7221(4)	b=21.3322(9)	c=25.3225(11)
	alpha=90	beta=90	gamma=90
Temperature:	299 K		
	Calculated		Reported
Volume	5251.7(4)		5251.7(4)
Space group	P 21 21 21		P 21 21 21
Hall group	P 2ac 2ab		P 2ac 2ab
Moiety formula	C29 H26O10		C29 H26O10
Sum formula	C29 H26O10		C29 H26O10
Mr	534.50		534.50
Dx,g cm-3	1.352		1.352
Z	8		8
Mu (mm-1)	0.103		0.103
F000	2240.0	2240.0	
F000’	2241.37		
h,k,lmax	112530		112530
Nref	9513[5302]		9469
Tmin,Tmax	0.964, 0.980		0.692, 0.746
Tmin’	0.960		

Correction method= # Reported T Limits: Tmin=0.692 Tmax=0.746

AbsCorr = MULTI-SCAN

Data completeness= 1.79/1.00 Theta(max)= 25.248

R(reflections)= 0.0303(8260) wR2(reflections)= 0.0774(9469)

S = 1.052 Npar= 710

Empirical formula	$C_{29}H_{26}NO_{10}$
Formula weight	534.50
Temperature/K	299.0
Crystal system	orthorhombic
Space group	$P2_12_12_1$
a/Å	9.7221(4)
b/Å	21.3322(9)
c/Å	25.3225(11)
α/°	90
β/°	90
γ/°	90
Volume/Å^3	5251.7(0)
Z	8
ρ_{calc}g/cm^3	1.352
μ/mm^{-1}	0.103
F(000)	2240.0
Crystal size/mm^3	0.4 × 0.3 × 0.2
Radiation	MoKα (λ = 0.71073)
2Θ range for data collection/°	4.994 to 50.496
Index ranges	$-11 \le h \le 11$, $-25 \le k \le 25$, $-30 \le l \le 30$
Reflections collected	75867
Independent reflections	9469 [R_{int} = 0.0340, R_{sigma} = 0.0194]
Data/restraints/parameters	9469/0/710
Goodness-of-fit on F^2	1.052
Final R indexes [I>=2σ (I)]	R_1 = 0.0303, wR_2 = 0.0702
Final R indexes [all data]	R_1 = 0.0394, wR_2 = 0.0774
Largest diff. peak/hole / e Å^{-3}	0.14/-0.12
Flack parameter	-0.16(18)

Figure S3 X-Ray Crystallographic Data for BB03 (CCDC: 2267684)

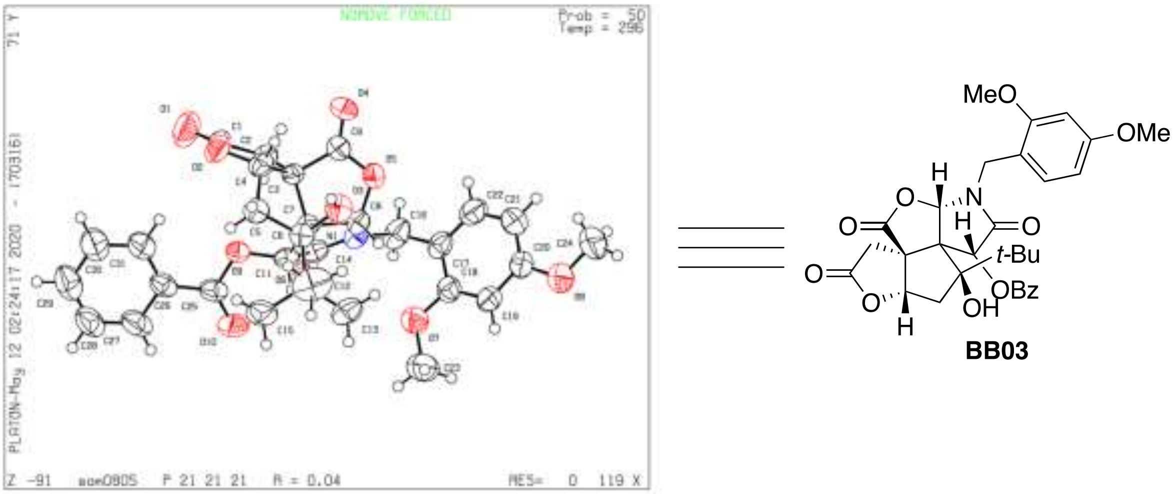

BB03

Bond precision: C-C = 0.0042 A Wavelength=1.54178

Cell:	a= 8.2499(4)	b= 17.6593(8)	c=20.0803(10)
	alpha=90	beta=90	gamma=90
Temperature:	296 K		

	Calculated	Reported
Volume	2925.5 (2)	2925.4 (2)
Space group	P 21 21 21	P 21 21 21
Hall group	P 2ac 2ab	P 2ac 2ab
Moiety formula	C31 H33 N O10 [+solvent]	C31 H33 N O10
Sum formula	C31 H33 N O10 [+solvent]	C31 H33 N O10
Mr	579.58	579.58
Dx,g cm-3	1.316	1.316
Z	4	4
Mu (mm-1)	0.823	0.823
F000	1224.0	1224.0
F000’	1228.19	
h,k,lmax	9, 21, 24	9, 21, 24
Nref	5392[3064]	5333
Tmin,Tmax	0.744, 0.848	0.285, 0.753
Tmin’	0.663	

Correction method= # Reported T Limits: Tmin=0.285 Tmax=0.753

AbsCorr = MULTI-SCAN

Data completeness= 1.74/0.99 Theta(max)= 68.652

R(reflections)= 0.0367(4303) wR2(reflections)= 0.1072(5333)

S = 1.040 Npar= 385

Empirical formula	$C_{31}H_{33}NO_{10}$
Formula weight	579.58
Temperature/K	296.15
Crystal system	orthorhombic
Space group	$P2_12_12_1$
a/Å	8.2499 (4)
b/Å	17.6593(8)
c/Å	20.0803(10)
α/°	90
β/°	90
γ/°	90
Volume/Å^3	2925.4(2)
Z	4
ρ_{calc}g/cm^3	1.316
μ/mm^{-1}	0.823
F(000)	1224.0
Crystal size/mm^3	0.5 × 0.3 × 0.2
Radiation	CuKα (λ = 1.54178)
2Θ range for data collection/°	10.134 to 137.304
Index ranges	$-9 \leq h \leq 8$, $-21 \leq k \leq 21$, $-24 \leq l \leq 24$
Reflections collected	43218
Independent reflections	5333 [R_{int} = 0.0986, R_{sigma} = 0.0479]
Data/restraints/parameters	5333/0/385
Goodness-of-fit on F^2	1.040
Final R indexes [I>=2σ (I)]	R_1 = 0.0367, wR_2 = 0.1018
Final R indexes [all data]	R_1 = 0.0570, wR_2 = 0.1072
Largest diff. peak/hole / e Å^{-3}	0.19/-0.19
Flack parameter	0.11(4)

Figure S4 X-Ray Crystallographic Data for BB15 (CCDC: 2267686)

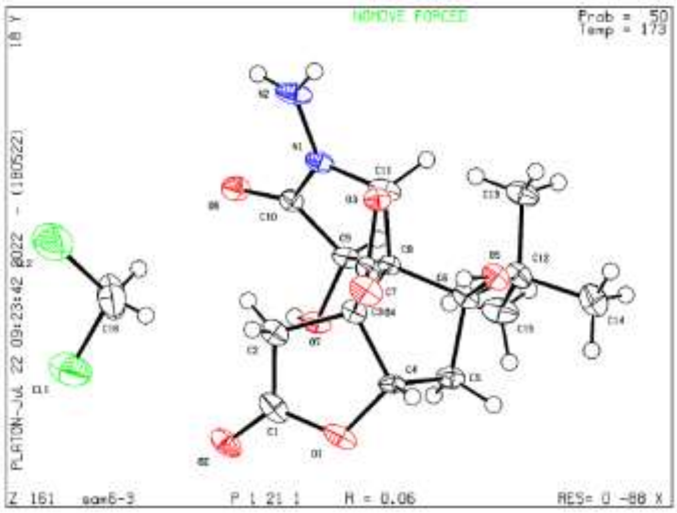

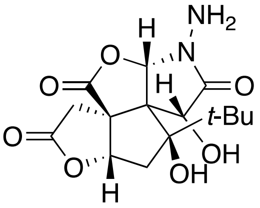

BB15

Bond precision: C-C = 0.0110 A Wavelength=1.54178

Cell: a= 10.4781(13) b= 8.0633(10) c=11.2947(14)

alpha=90 beta=107.493 (3) gamma=90

Temperature: 173 K

	Calculated	Reported
Volume	910.1 (2)	910.1 (2)
Space group	P 21	P 1 21 1
Hall group	P 2yb	P 2yb
Moiety formula	C15 H20 N2 O7, C H2 O12	C15 H20 N2 O7, C H2 Cl2
Sum formula	C16 H22 Cl2 N2 O7	C16 H22 C12 N2 O7
Mr	425.26	425.25
Dx,g cm-3	1.552	1.552
Z	2	2
Mu (mm-1)	3.605	3.605
F000	444.0	444.0
F000’	446.81	
h,k,lmax	12, 9, 13	12, 9, 13
Nref	3391[1826]	3092
Tmin,Tmax	0.314, 0.339	0.460, 0.753
Tmin’	0.206	

Correction method= # Reported T Limits: Tmin=0.460 Tmax=0.753

AbsCorr = MULTI-SCAN

Data completeness= 1.69/0.91 Theta(max)= 68.928

R(reflections)= 0.0630(2538) wR2(reflections)= 0.2276(3092)

S = 1.156 Npar= 250

Empirical formula	$C_{16}H_{22}Cl_2N_2O_7$
Formula weight	425.25
Temperature/K	173.00
Crystal system	monoclinic
Space group	$P2_1$
a/Å	10.4781 (13)
b/Å	8.0633(10)
c/Å	11.2947(14)
α/°	90
β/°	107.493(3)
γ/°	90
Volume/Å^3	910.1(2)
Z	2
ρ_{calc}g/cm^3	1.552
μ/mm^{-1}	3.605
F(000)	444.0
Crystal size/mm^3	0.4 × 0.3 × 0.3
Radiation	CuKα (λ = 1.54178)
2Θ range for data collection/°	8.208 to 137.856
Index ranges	$-12 \leq h \leq 12$, $-9 \leq k \leq 9$, $-13 \leq l \leq 13$
Reflections collected	7506
Independent reflections	3092 [R_{int} = 0.0832, R_{sigma} = 0.1317]
Data/restraints/parameters	3092/1/250
Goodness-of-fit on F^2	1.156
Final R indexes [I>=2σ (I)]	R_1 = 0.0630, wR_2 = 0.1683
Final R indexes [all data]	R_1 = 0.1268, wR_2 = 0.2276
Largest diff. peak/hole / e Å^{-3}	0.84/-0.89
Flack parameter	0.5

Figure S5 X-Ray Crystallographic Data for BB17 (CCDC: 2325065)

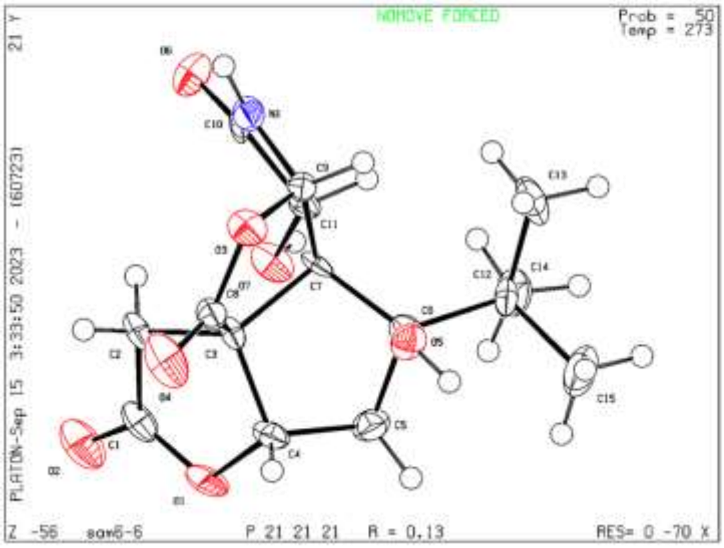

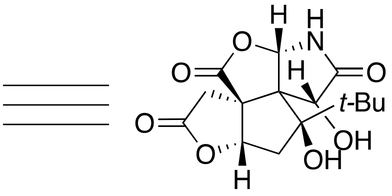

BB17

Bond precision:	C-C = 0.0113 A		Wavelength=0.71073
Cell:	a= 8.6129(7)	b= 10.4118 (9)	c=16.2131 (12)
	alpha=90	beta=90	gamma=90
Temperature:	273 K		

	Calculated	Reported
Volume	1453.9 (2)	1453.9 (2)
Space group	P 21 21 21	P 21 21 21
Hall group	P 2ac 2ab	P 2ac 2ab
Moiety formula	C15 H19 N O7	C15 H19 N O7
Sum formula	C15 H19 N O7	C15 H19 N O7
Mr	325.31	325.31
Dx,g cm-3	1.486	1.486
Z	4	4
Mu (mm-1)	0.119	0.119
F000	688.0	688.0
F000’	688.43	
h,k,lmax	10, 12, 19	10, 12, 19
Nref	2635[1533]	2577
Tmin,Tmax	0.944, 0.965	0.220, 0.746
Tmin’	0.942	

Correction method= # Reported T Limits: Tmin=0.220 Tmax=0.746

AbsCorr = MULTI-SCAN

Data completeness= 1.68/0.98 Theta(max)= 25.236

R(reflections)= 0.1300(2233) wR2(reflections)= 0.3211(2577)

S = 1.303 Npar= 215

Empirical formula	$C_{15}H_{19}NO_7$
Formula weight	325.31
Temperature/K	273.15
Crystal system	orthorhombic
Space group	$P2_12_12_1$
a/Å	8.6129 (7)
b/Å	10.4118 (9)
c/Å	16.2131(12)
α/°	90
β/°	90
γ/°	90
Volume/Å^3	1453.9(2)
Z	4
ρ_{calc}g/cm^3	1.486
μ/mm^{-1}	0.119
F(000)	688.0
Crystal size/mm^3	0.5 × 0.4 × 0.3
Radiation	MoKα (λ = 0.71073)
2Θ range for data collection/°	4.65 to 50.472
Index ranges	-10≤ h ≤ 10, -12 ≤ k ≤ 12, -19 ≤ l ≤ 19
Reflections collected	11463
Independent reflections	2577 [R_{int} = 0.1484, R_{sigma} = 0.1129]
Data/restraints/parameters	2577/0/215
Goodness-of-fit on F^2	1.303
Final R indexes [I>=2σ (I)]	R_1 = 0.1300, wR_2 = 0.3143
Final R indexes [all data]	R_1 = 0.1362, wR_2 = 0.3211
Largest diff. peak/hole / e Å^{-3}	0.98/-0.97
Flack parameter	-3(4)

Figure S6 X-Ray Crystallographic Data for 5 (CCDC: 2267681)

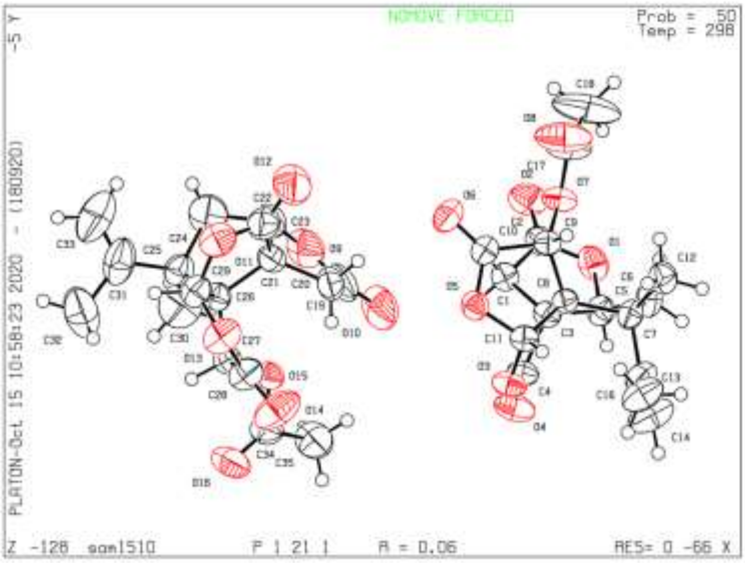

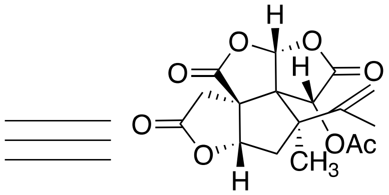

5

Bond precision:	C-C = 0.0069 A		Wavelength=0.71073
Cell:	a=9.4653(8)	b=14.0313(14)	c=12.4803(12)
	alpha=90	beta=90.658 (3)	gamma=90
Temperature:	298 K		
	Calculated		Reported
Volume	1657.4(3)		1657.4(3)
Space group	P 21		P 1 21 1
Hall group	P 2yb		P 2yb
Moiety formula	C17 H18 N O8		C17 H18 N O8
Sum formula	C17 H18 N O8		C17 H18 N O8
Mr	350.31		350.31
Dx,g cm-3	1.404		1.404
Z	4		4
Mu (mm-1)	0.113		0.113
F000	736.0	736.0	
F000’	736.49		
h,k,lmax	12, 18, 16		12, 18, 16
Nref	7970[4147]		7936
Tmin,Tmax	0.967, 0.978		0.711, 0.746
Tmin’	0.967		

Correction method= # Reported T Limits: Tmin=0.711 Tmax=0.746

AbsCorr = MULTI-SCAN

Data completeness= 1.91/1.00 Theta(max)= 27.949

R(reflections)= 0.0554(6221) wR2(reflections)= 0.1626(7936)

S = 1.031 Npar= 458

Empirical formula	C17H18O8
Formula weight	350.31
Temperature/K	298.0
Crystal system	monoclinic
Space group	$P2_1$
a/Å	9.4653(8)
b/Å	14.0313(14)
c/Å	12.4803(12)
α/°	90
β/°	90.658(3)
γ/°	90
Volume/Å^3	1657.4(3)
Z	4
ρ_{calc}g/cm^3	1.404
μ/mm^{-1}	0.113
F(000)	736.0
Crystal size/mm^3	0.3 × 0.2 × 0.2
Radiation	MoKα (λ = 0.71073)
2Θ range for data collection/°	4.368 to 55.898
Index ranges	$-12 \leq h \leq 12$, $-18 \leq k \leq 18$, $-16 \leq l \leq 16$
Reflections collected	56549
Independent reflections	7936 [R_{int} = 0.0596, R_{sigma} = 0.0299]
Data/restraints/parameters	7936/1/458
Goodness-of-fit on F^2	1.031
Final R indexes [I>=2σ (I)]	R_1 = 0.0554, wR_2 = 0.1455
Final R indexes [all data]	R_1 = 0.0744, wR_2 = 0.1626
Largest diff. peak/hole / e Å^{-3}	0.65/-0.30
Flack parameter	0.3(3)

Figure S7 X-Ray Crystallographic Data for BB33 (CCDC: 2267682)

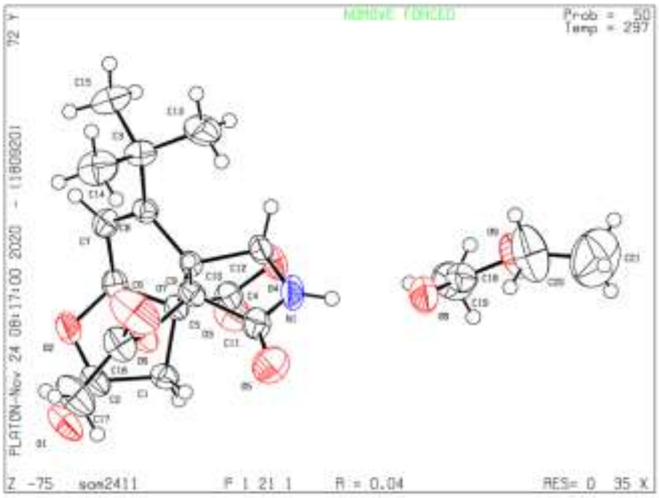

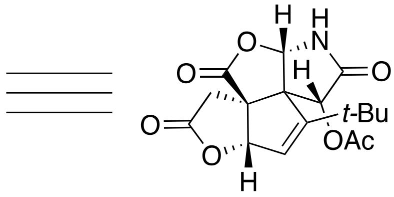

BB33

Bond precision:	C-C = 0.0038 A		Wavelength=0.71073
Cell:	a= 7.8907(8)	b= 16.0611(17)	c=8.887(1)
	alpha=90	beta=103.966 (3)	gamma=90
Temperature:	297 K		

	Calculated	Reported
Volume	1093.0 (2)	1093.0 (2)
Space group	P 21	P 1 21 1
Hall group	P 2yb	P 2yb
Moiety formula	C17 H19 N O7, C4 H8 O2	C17 H19 N O7, C4 H8 O2
Sum formula	C21 H27 N O9	C21 H27 N O9
Mr	437.44	437.43
Dx,g cm-3	1.329	1.329
Z	2	2
Mu (mm-1)	0.104	0.104
F000	464.0	464.0
F000’	464.28	
h,k,lmax	10, 21, 11	10, 21, 11
Nref	5259[2723]	5207
Tmin,Tmax	0.963, 0.969	0.688, 0.746
Tmin’	0.959	

Correction method= # Reported T Limits: Tmin=0.688 Tmax=0.746

AbsCorr = MULTI-SCAN

Data completeness= 1.91/0.99 Theta(max)= 27.915

R(reflections)= 0.0383(4677) wR2(reflections)= 0.1015(5207)

S = 1.018 Npar= 287

Empirical formula	C21H27NO9
Formula weight	437.43
Temperature/K	297
Crystal system	monoclinic
Space group	$P2_1$
a/Å	7.8907 (8)
b/Å	16.0611(17)
c/Å	8.8870(10)
α/°	90
β/°	109.966(3)
γ/°	90
Volume/Å^3	1093.0(2)
Z	2
ρ_{calc}g/cm^3	1.329
μ/mm^{-1}	0.104
F(000)	464.0
Crystal size/mm^3	0.4 × 0.3 × 0.3
Radiation	MoKα (λ = 0.71073)
2Θ range for data collection/°	5.894 to 55.83
Index ranges	-10 ≤ h ≤ 10, -21 ≤ k ≤ 21, -11 ≤ l ≤ 10
Reflections collected	18691
Independent reflections	5207 [R_{int} = 0.0246, R_{sigma} = 0.0235]
Data/restraints/parameters	5207/1/287
Goodness-of-fit on F^2	1.018
Final R indexes [I>=2σ (I)]	R_1 = 0.0383, wR_2 = 0.0973
Final R indexes [all data]	R_1 = 0.0447, wR_2 = 0.1015
Largest diff. peak/hole / e Å^{-3}	0.19/-0.21
Flack parameter	0.1(2)

2.3 Copies of NMR Spectra

^{1}H NMR (500 MHz, $CDCl_3$) spectrum of 1

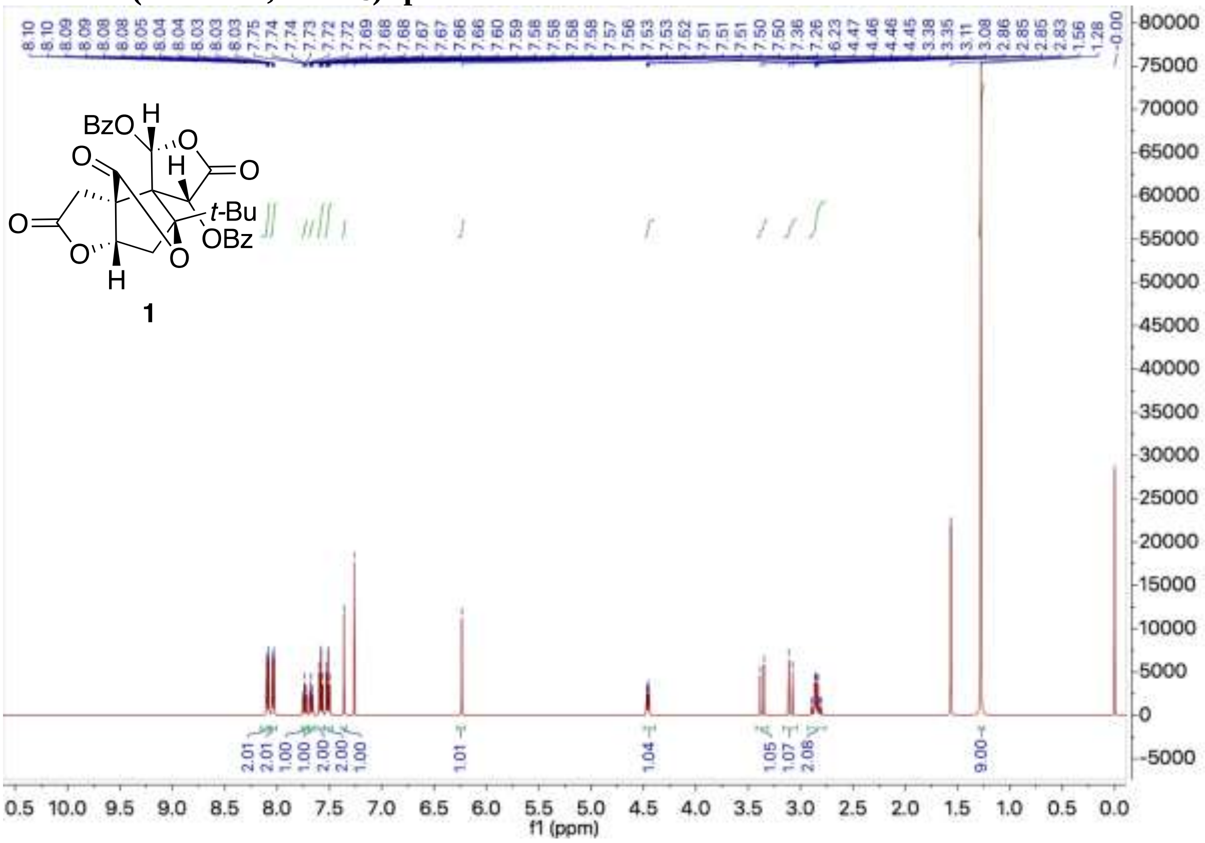

^{13}C NMR (126 MHz, $CDCl_3$) spectrum of 1

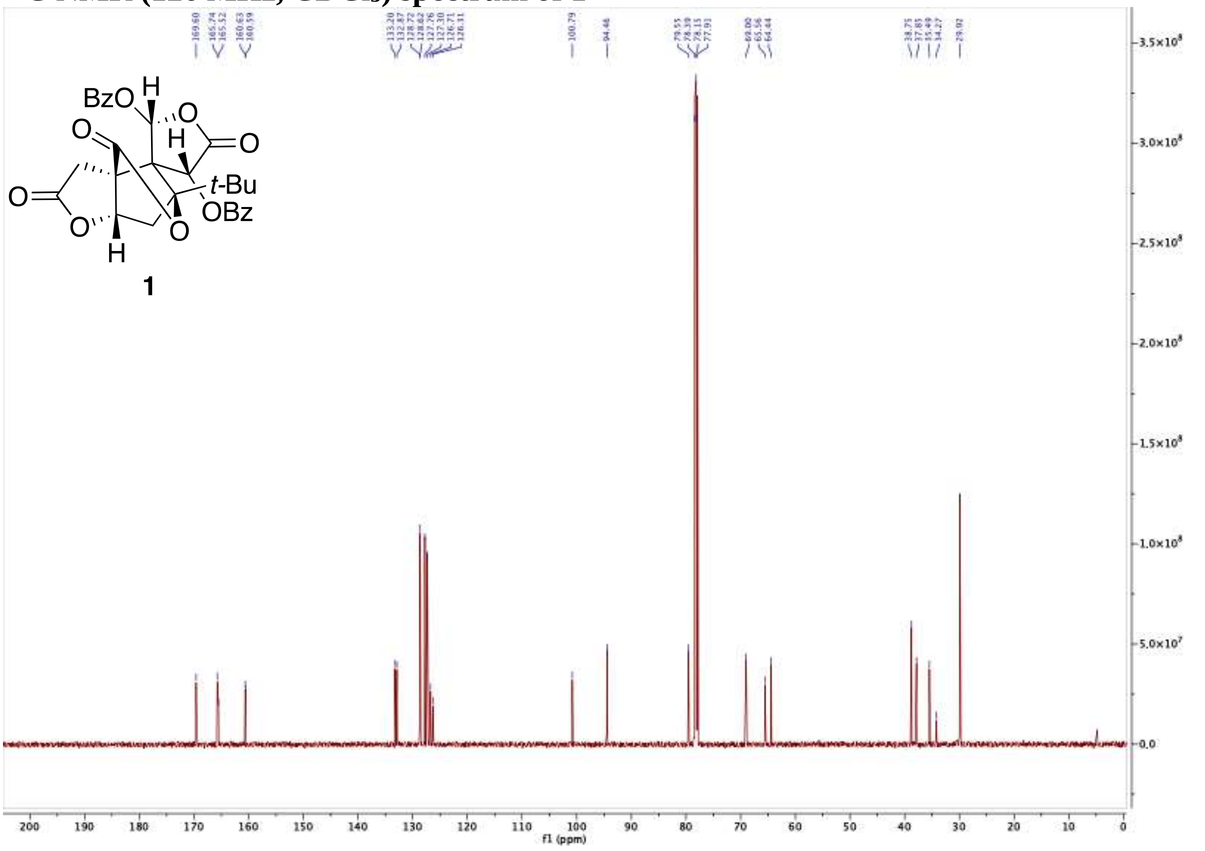

^{1}H NMR (500 MHz, CDCl$_3$) spectrum of 2

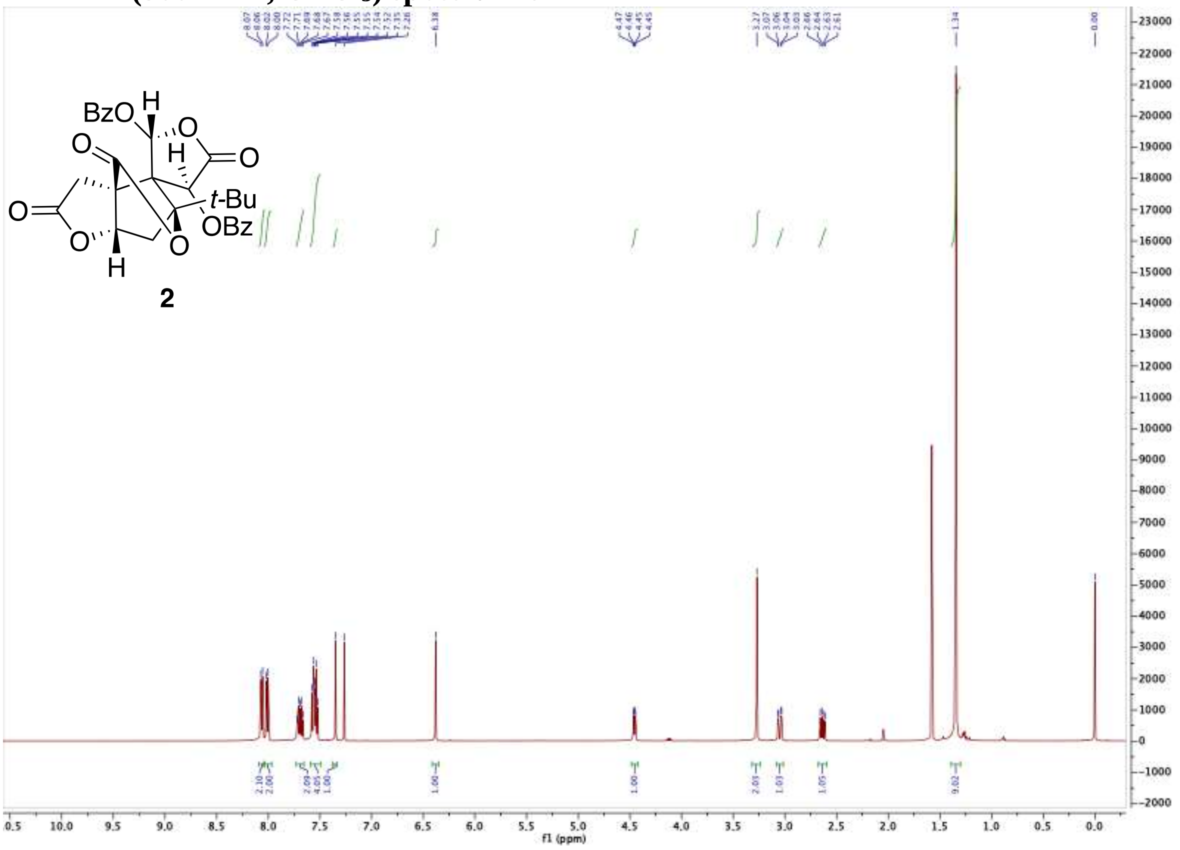

^{13}C NMR (126 MHz, CDCl$_3$) spectrum of 2

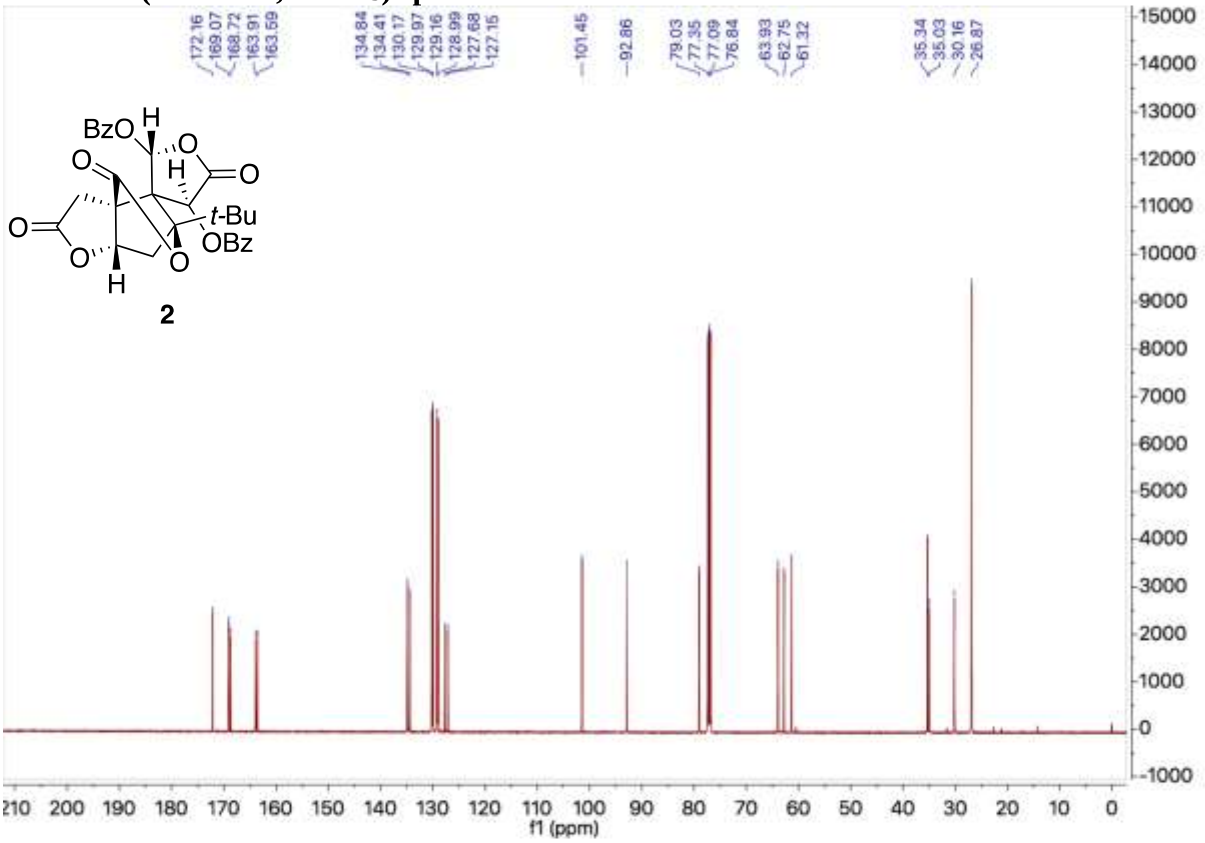

^{1}H NMR (400 MHz, CDCl$_3$) spectrum of BB01

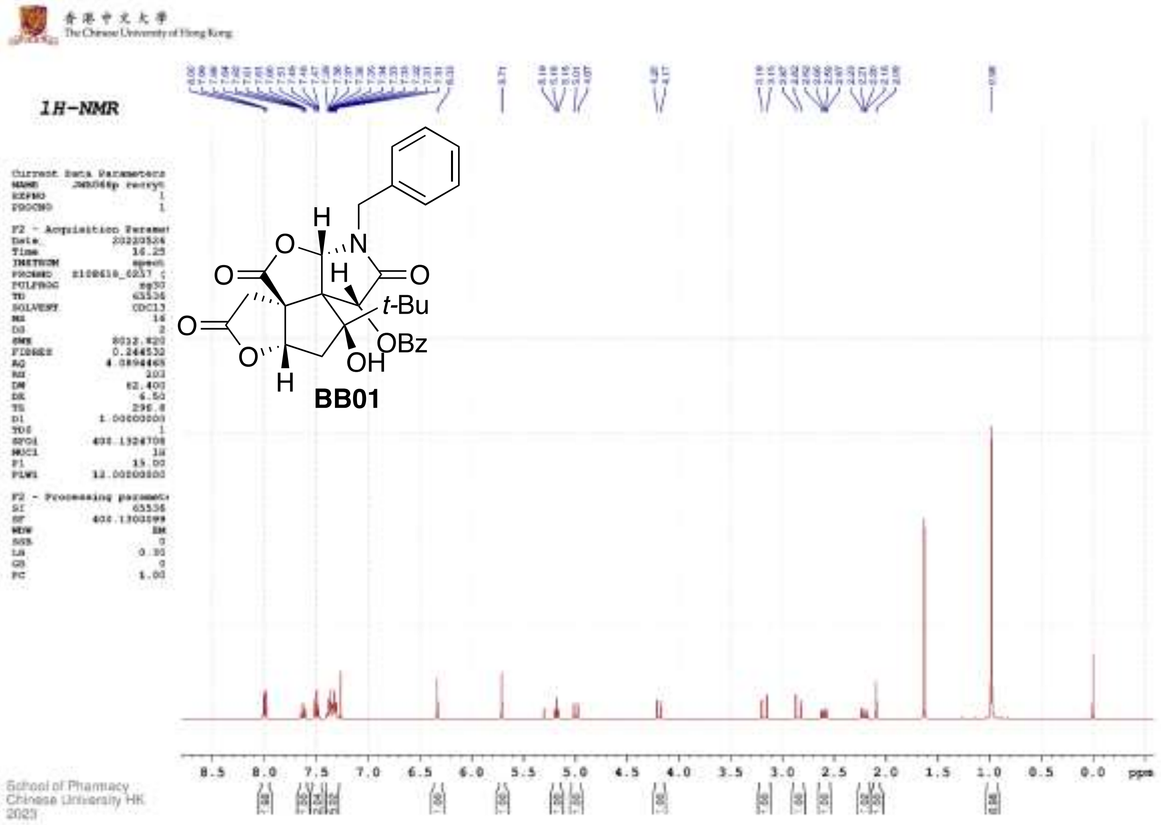

^{13}C NMR (126 MHz, CDCl$_3$) spectrum of BB01

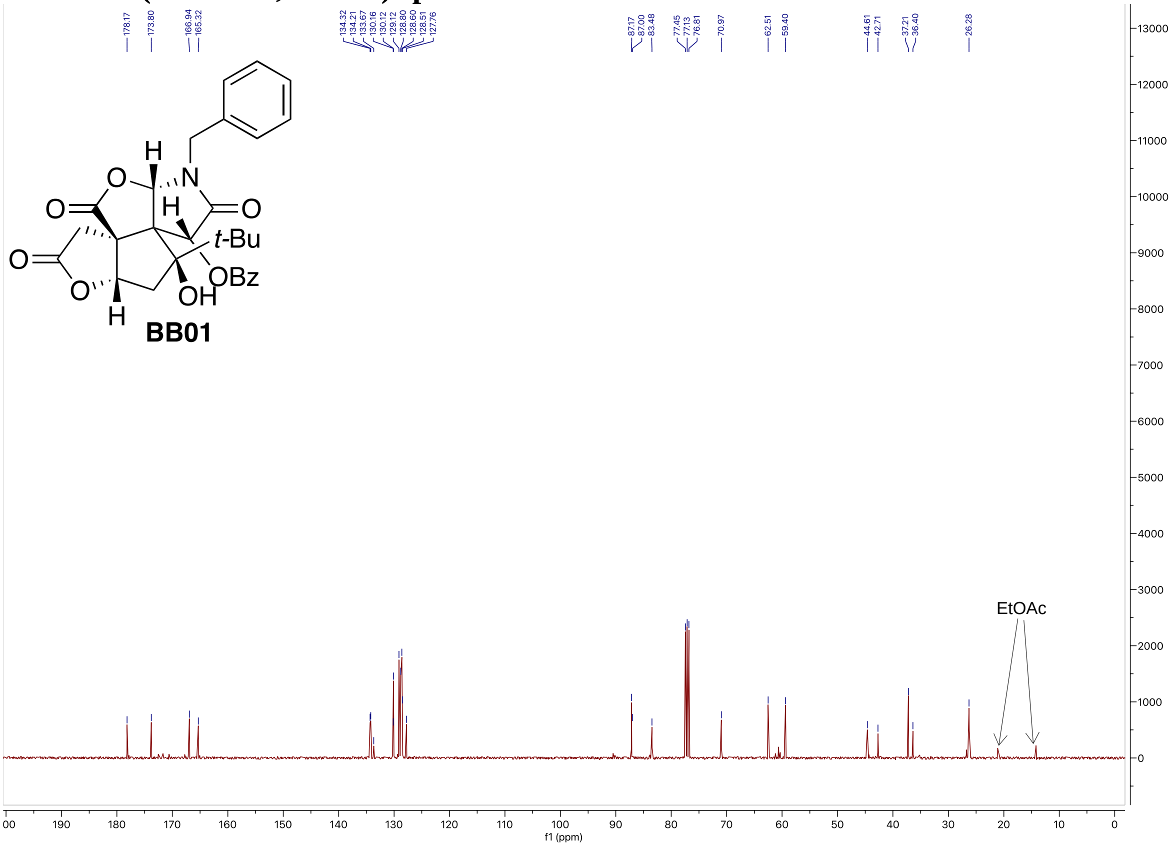

^{1}H NMR (500 MHz, Methanol-d_4) spectrum of BB02

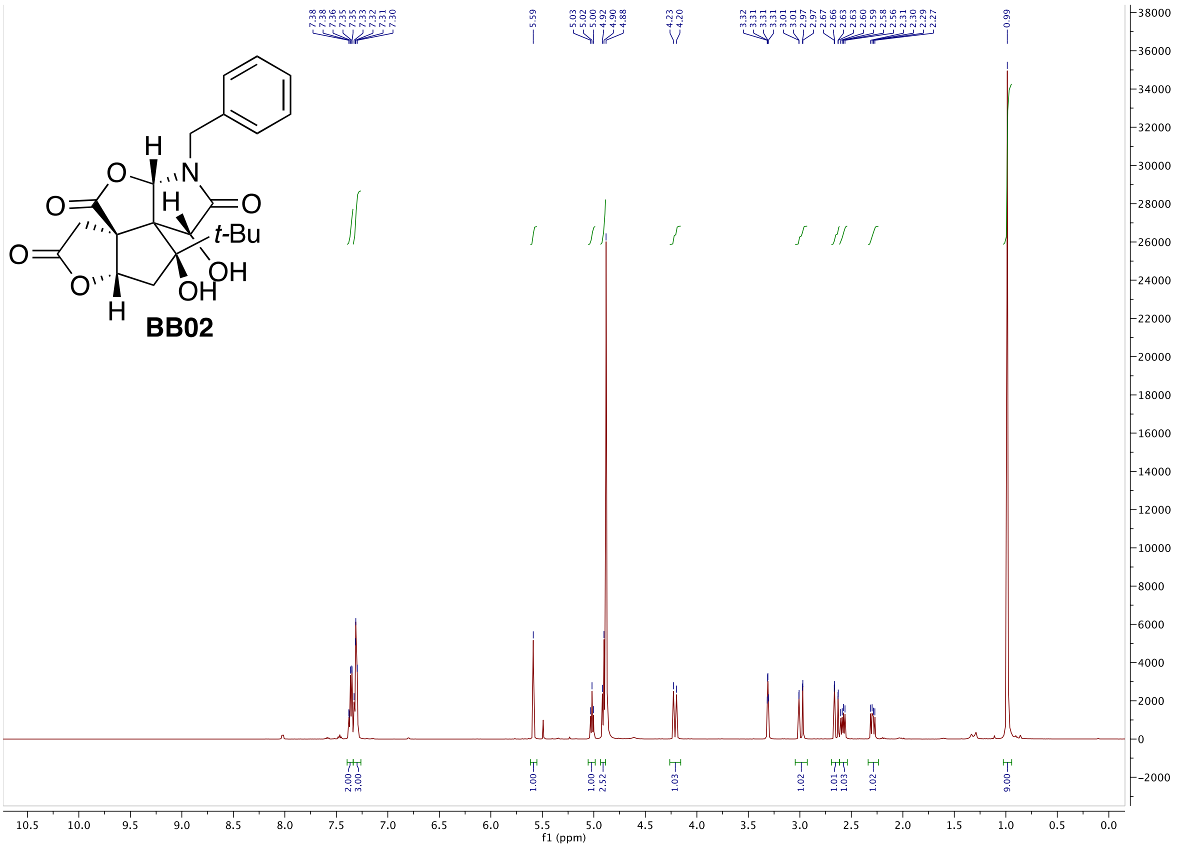

^{13}C NMR (126 MHz, Methanol-d_4) spectrum of BB02

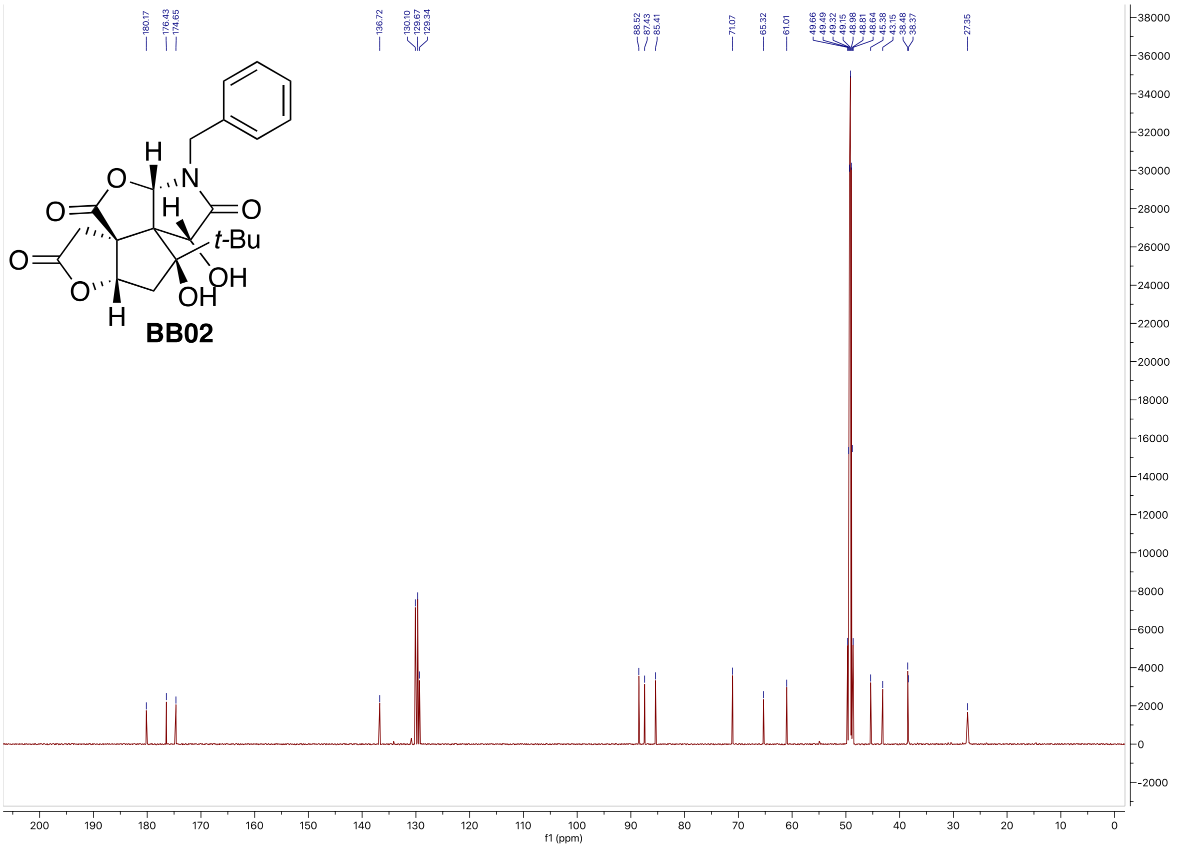

^1H NMR(500 MHz, $CDCl_3$) spectrum of BB03

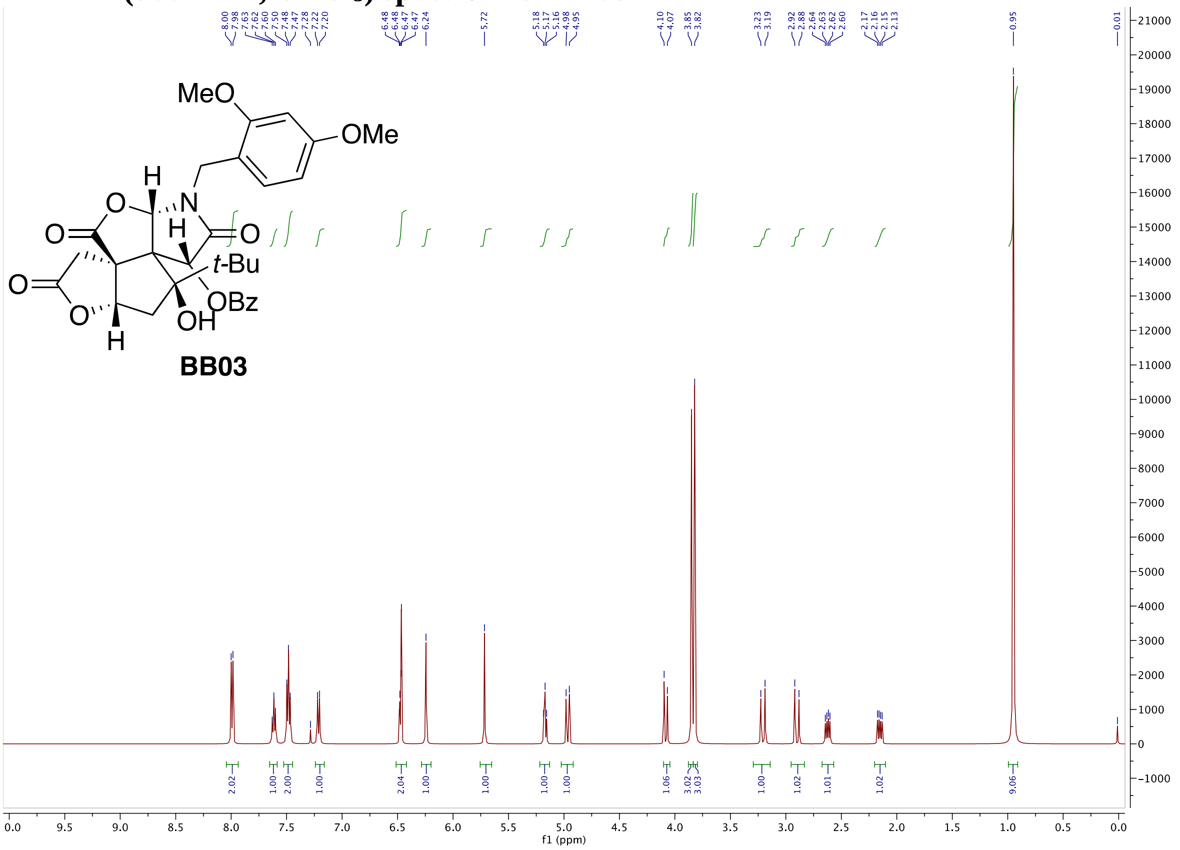

^{13}C NMR (126 MHz, $CDCl_3$) spectrum of BB03

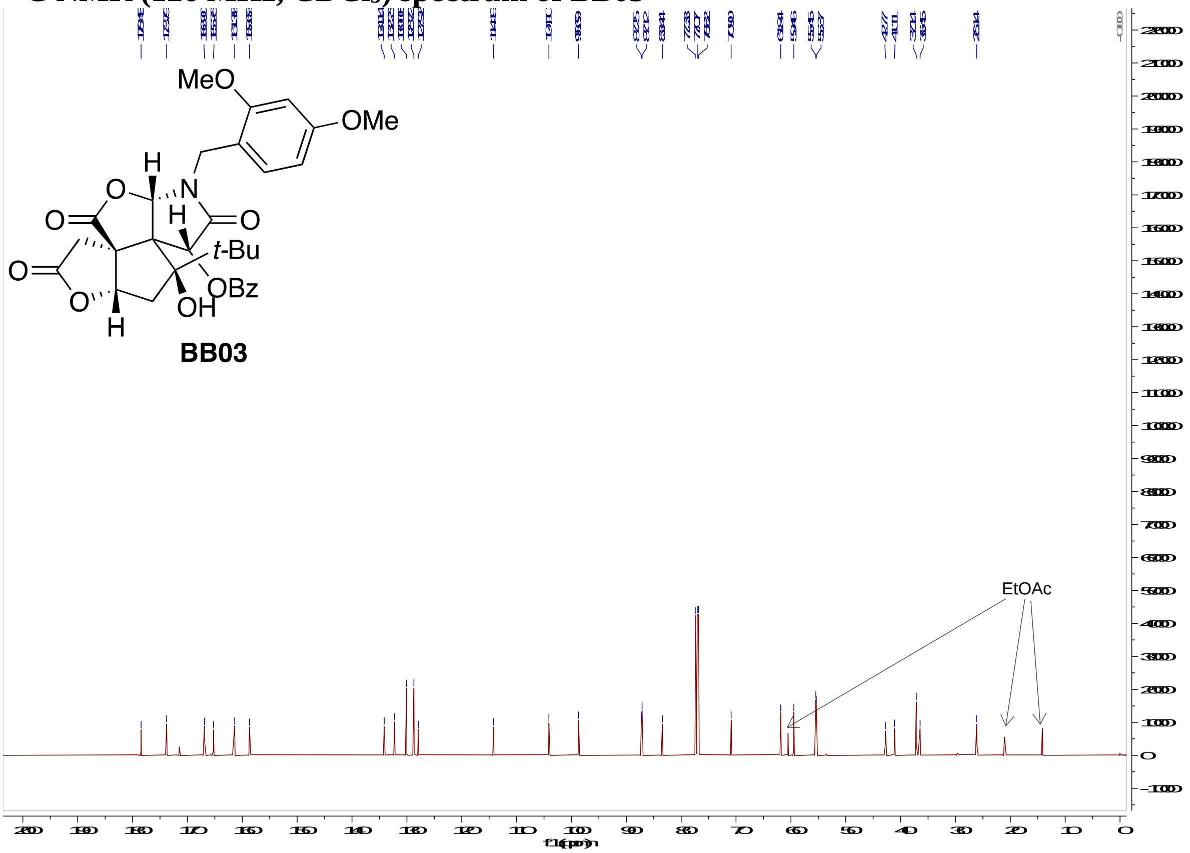

HMBC (126 MHz, CDCl₃) spectrum of BB03

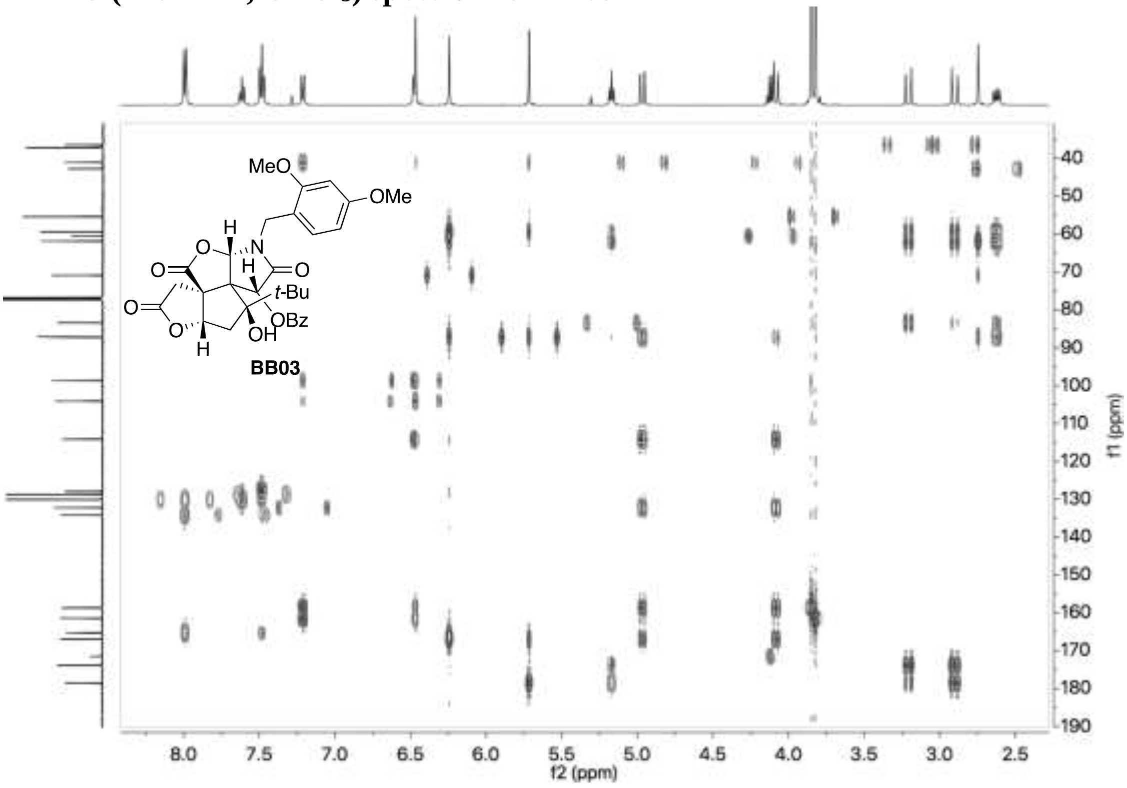

HSQC (126 MHz, CDCl₃) spectrum of BB03

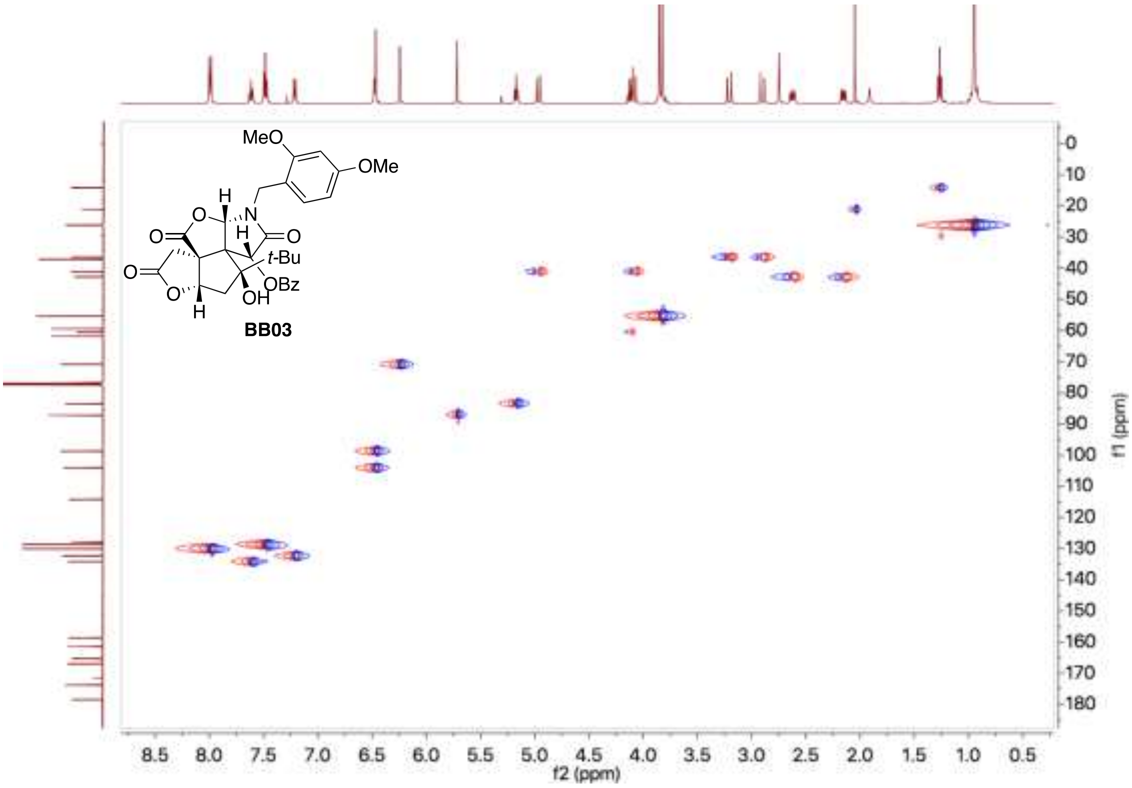

^{1}H NMR (500 MHz, CDCl$_3$) spectrum of BB04

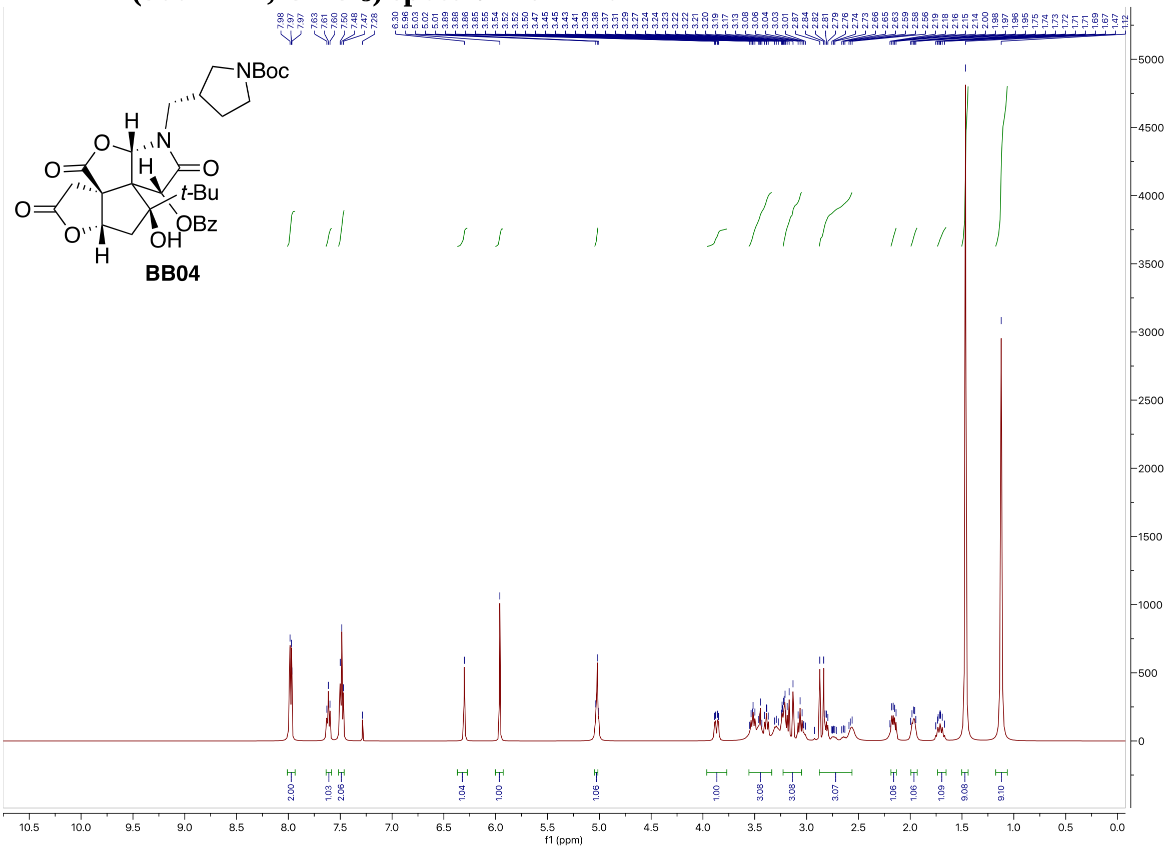

^{13}C NMR (126 MHz, CDCl$_3$) spectrum of BB04

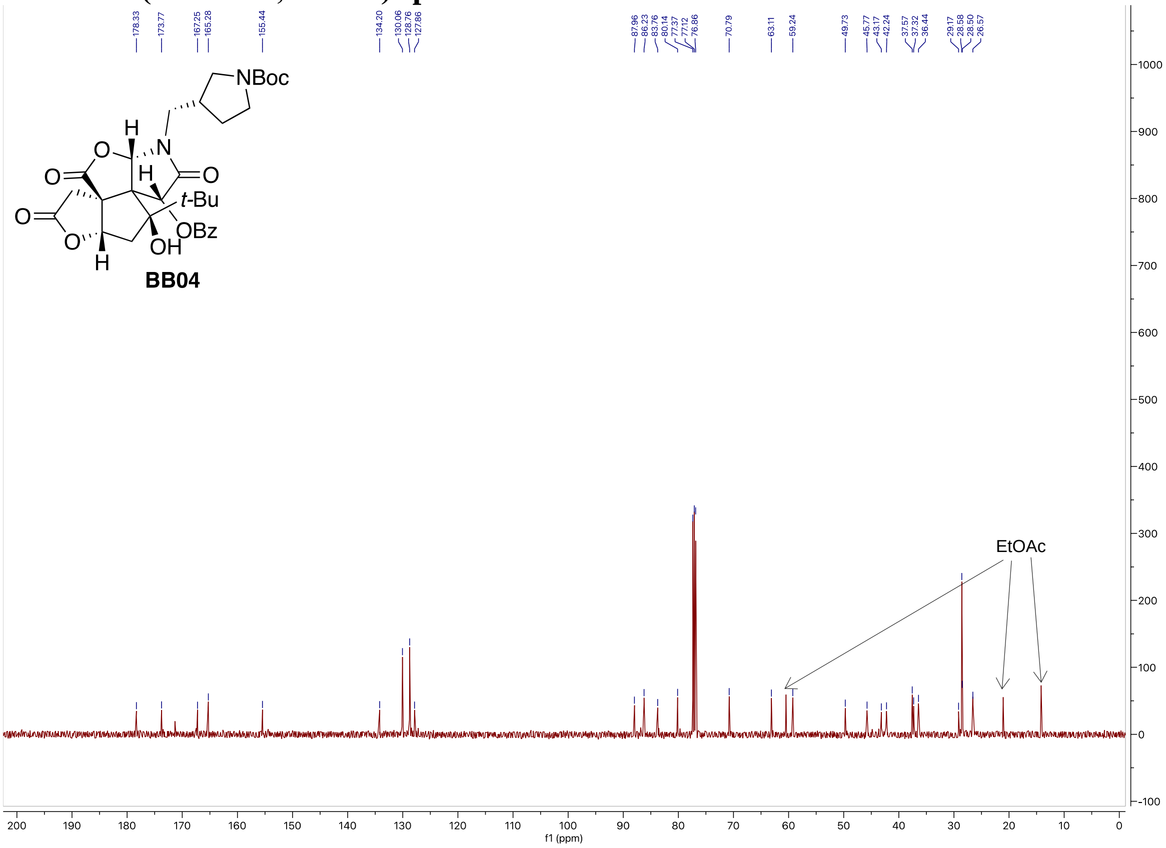

^{1}H NMR (500 MHz, CDCl$_3$) spectrum of BB05

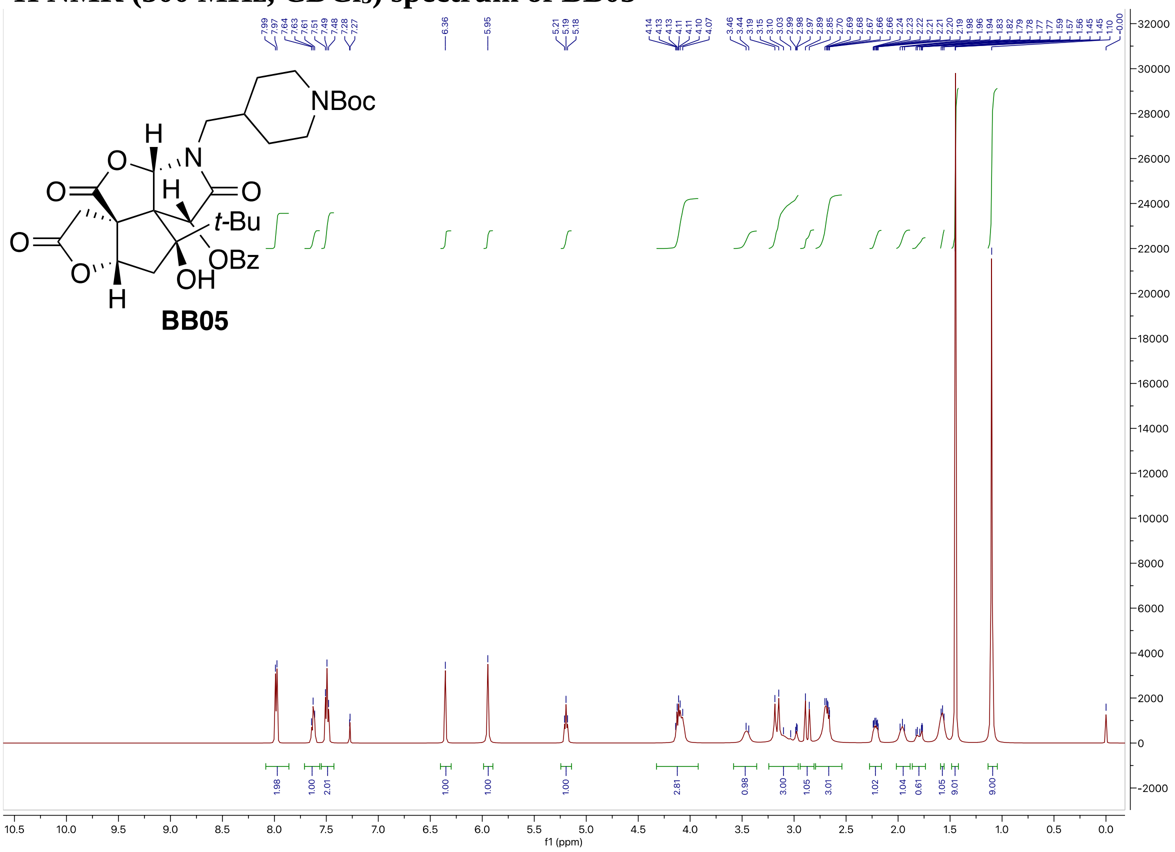

^{13}C NMR (126 MHz, CDCl$_3$) spectrum of BB05

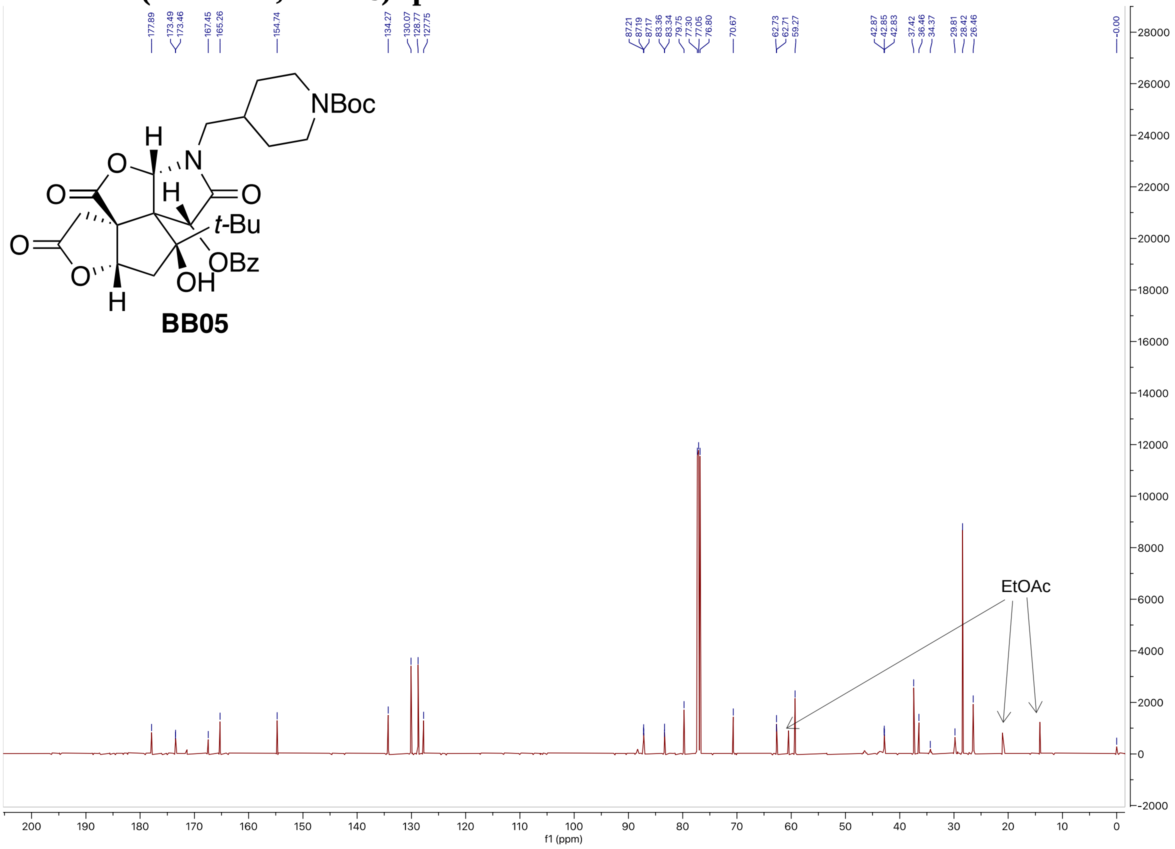

^{1}H NMR (400 MHz, $CDCl_3$) spectrum of BB06

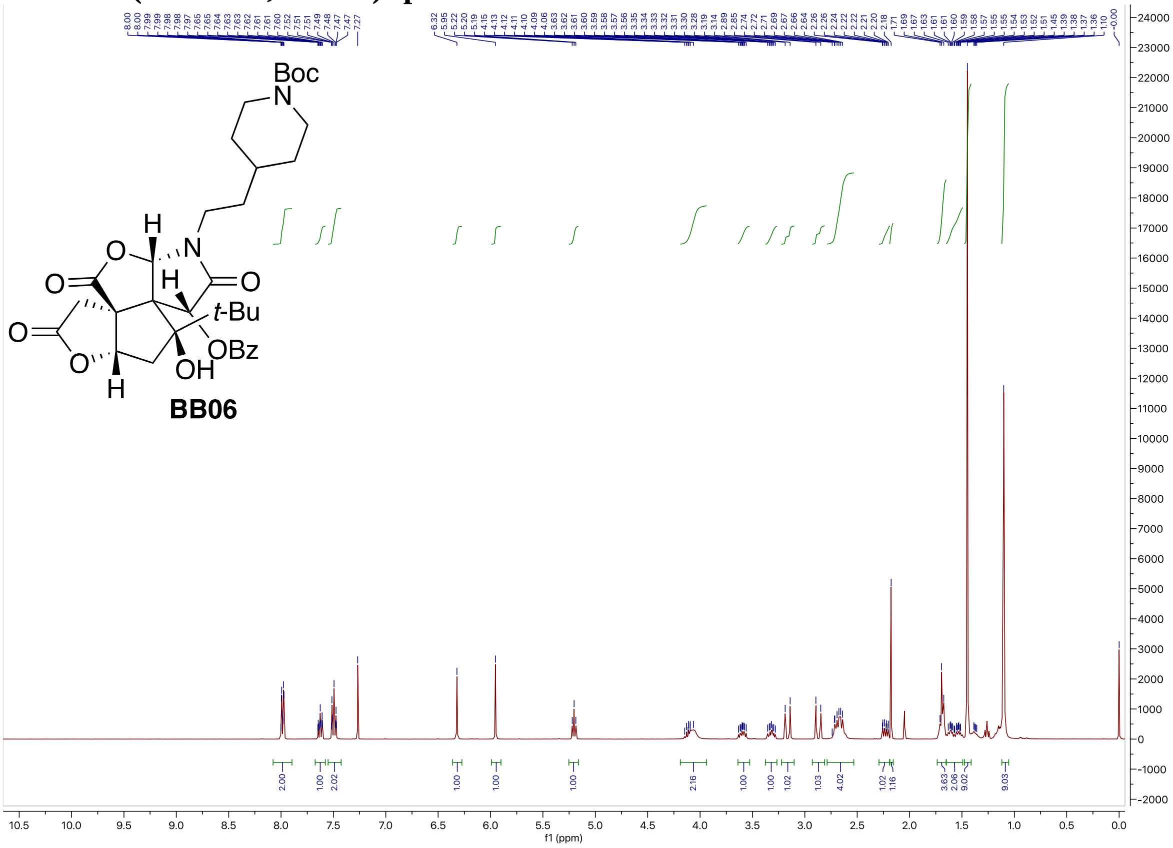

^{13}C NMR (101 MHz, $CDCl_3$) spectrum of BB06

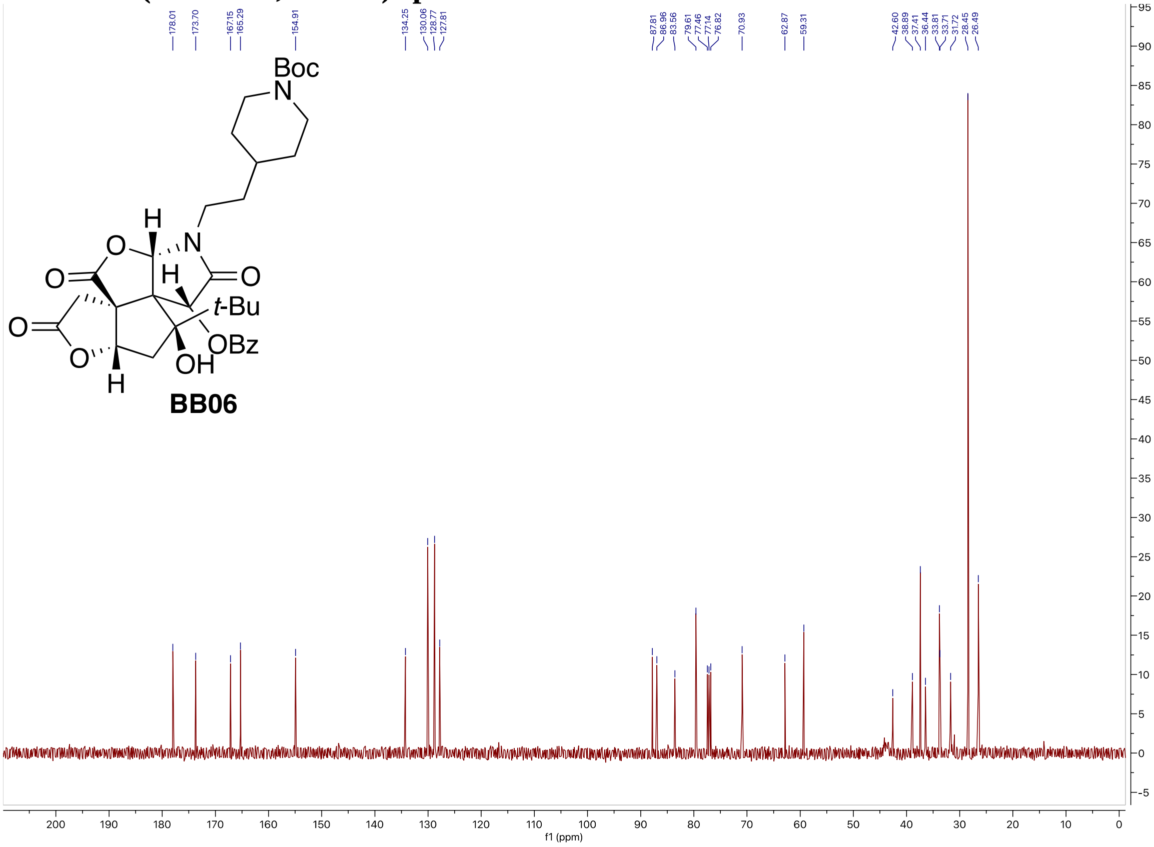

^{1}H NMR (400 MHz, CDCl3) spectrum of BB07

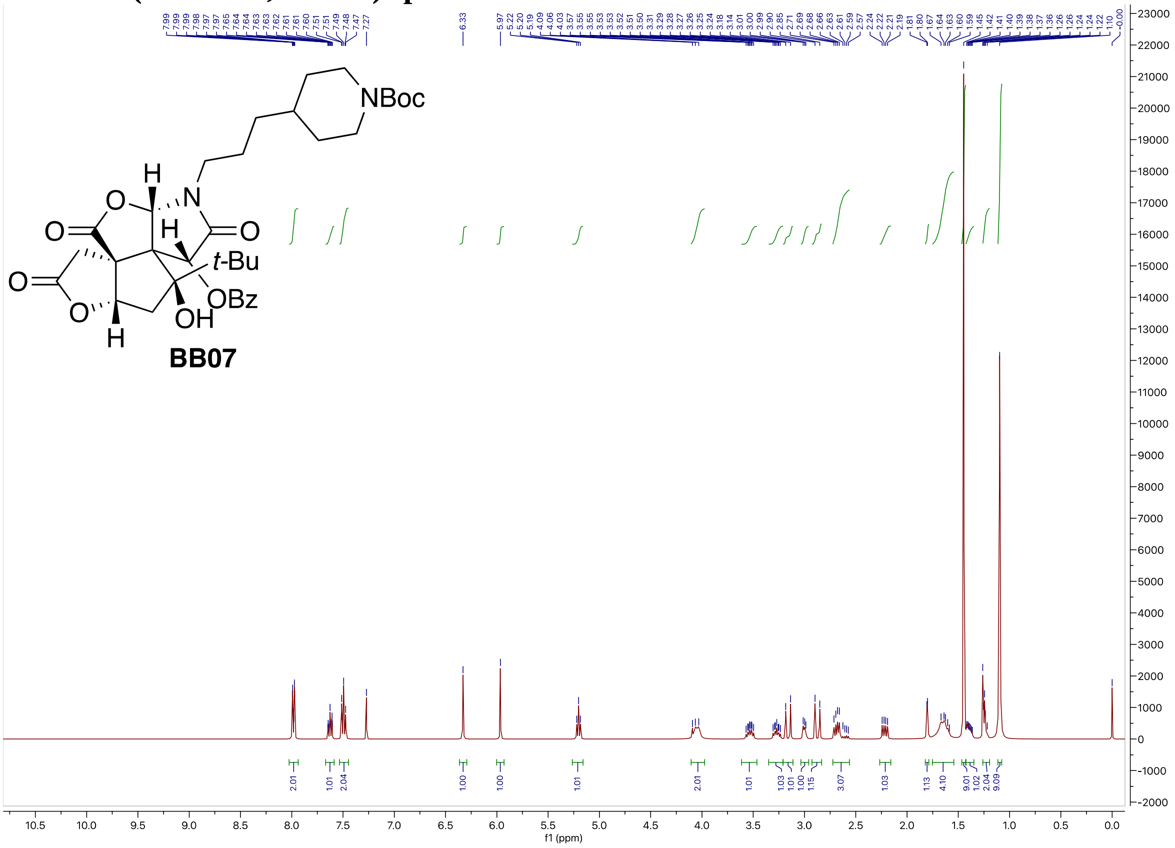

^{13}C NMR (101 MHz, CDCl3) spectrum of BB07

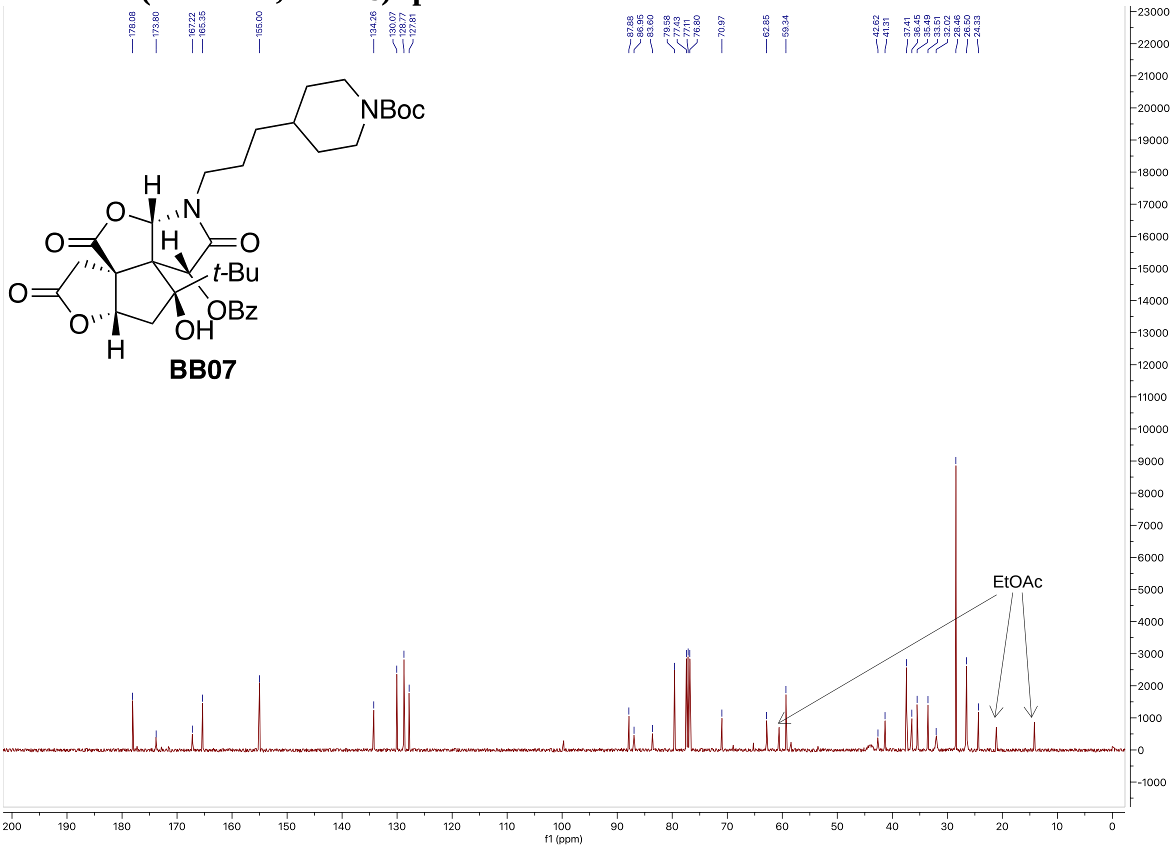

^{1}H NMR (400 MHz, $CDCl_3$) spectrum of BB08

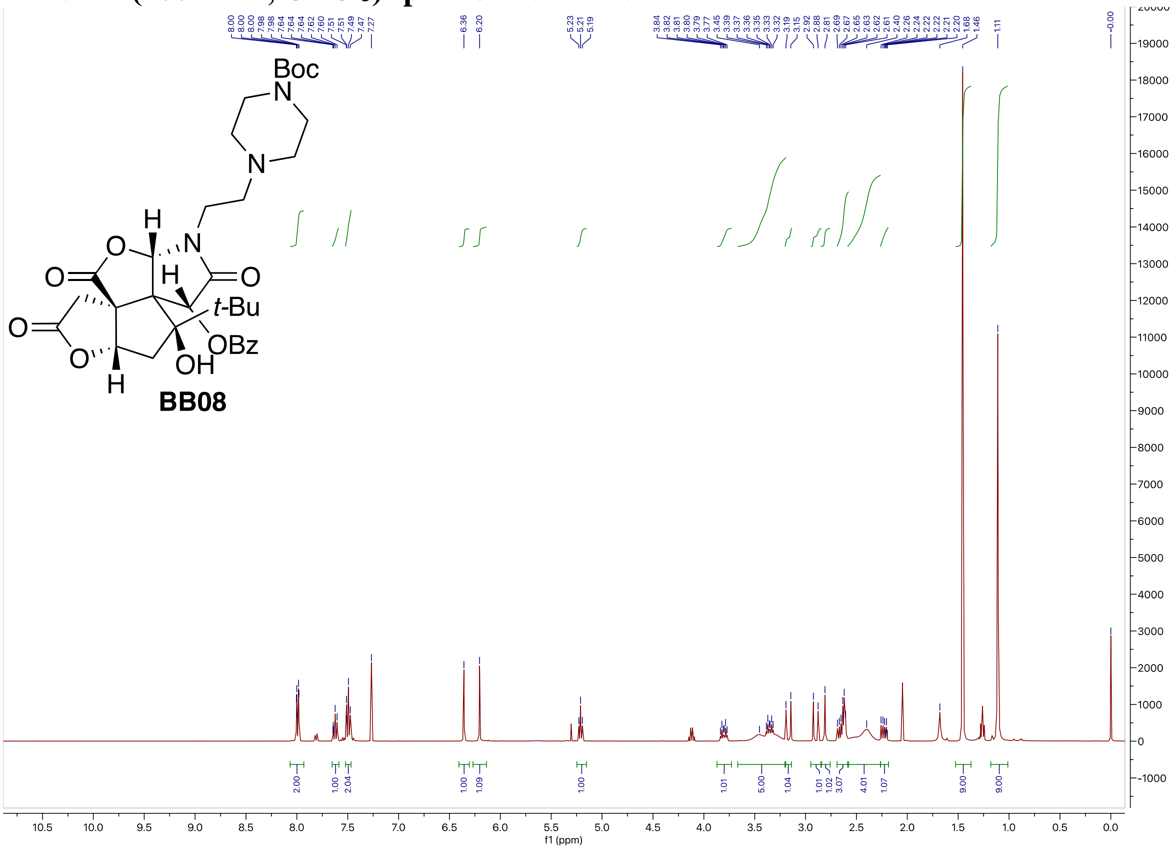

^{13}C NMR (101 MHz, $CDCl_3$) spectrum of BB08

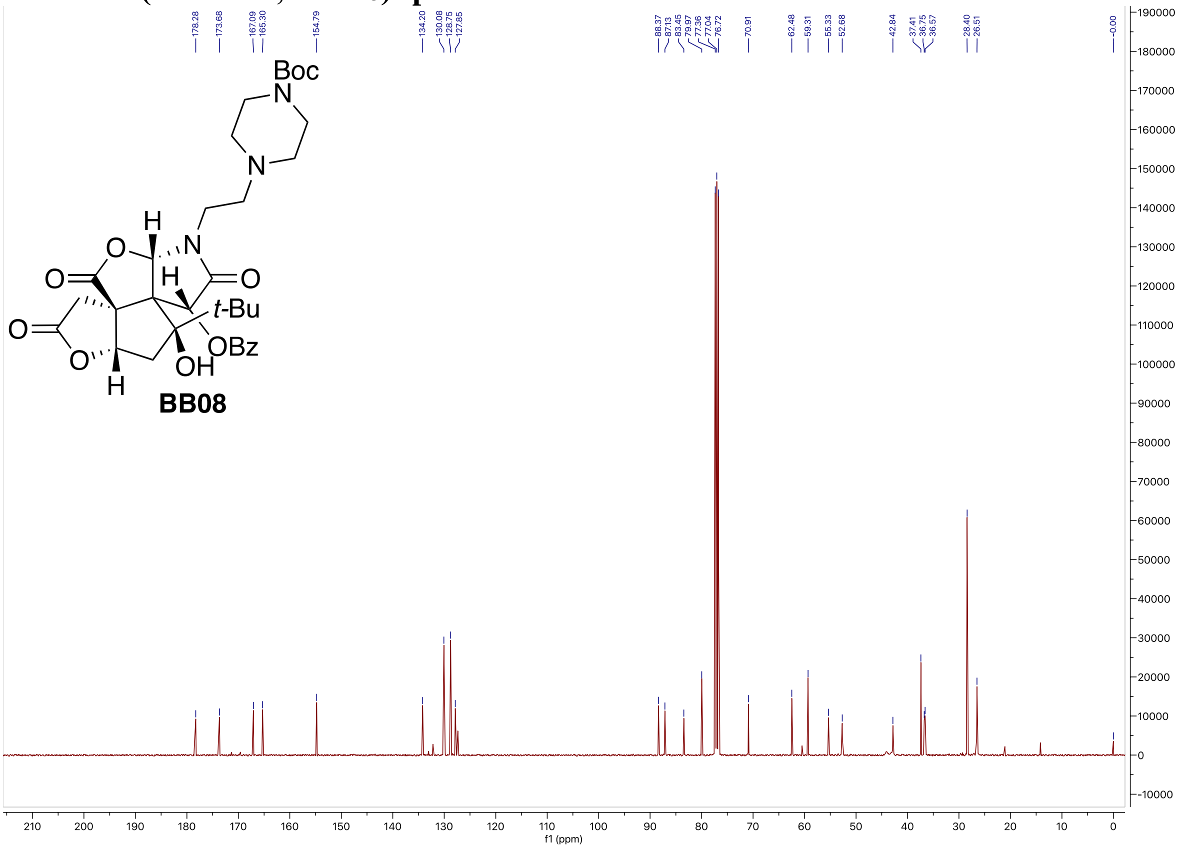

^{1}H NMR (500 MHz, Methanol-*d*$_4$) spectrum of BB09

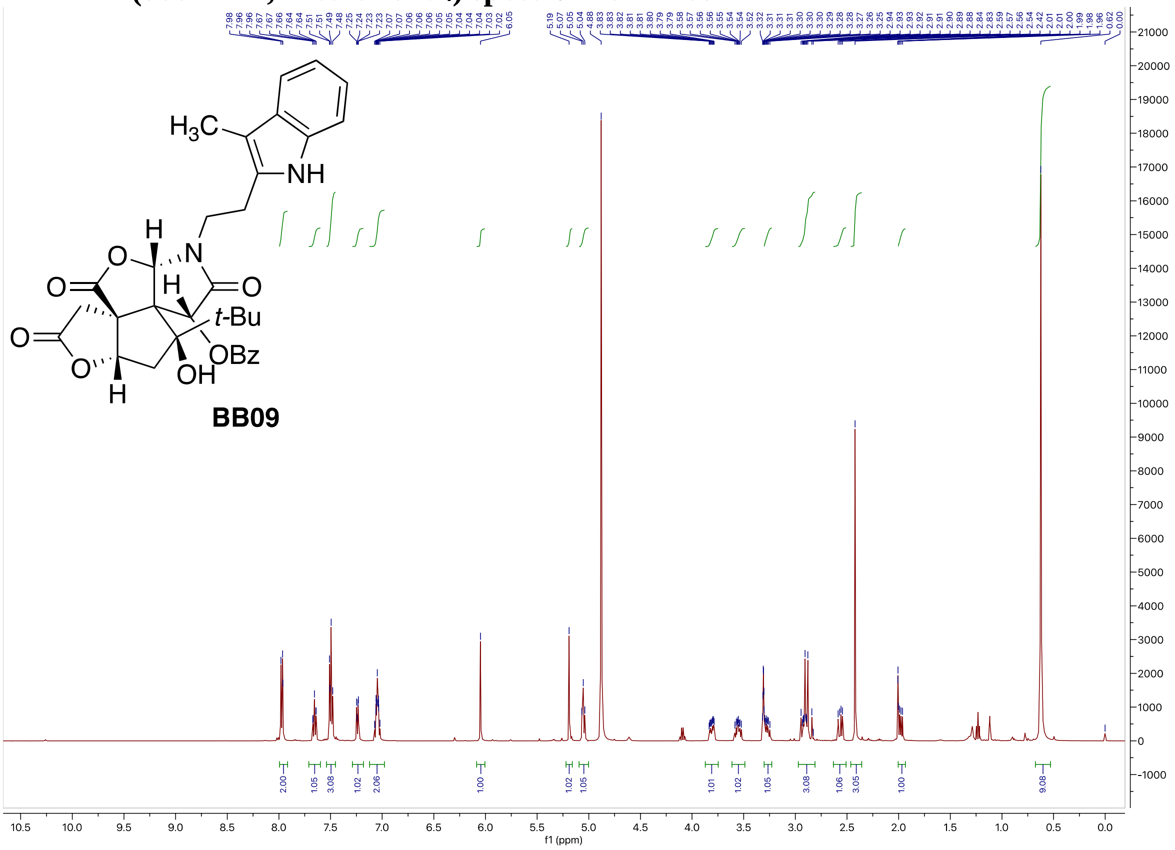

^{13}C NMR (126 MHz, Methanol-*d*$_4$) spectrum of BB09

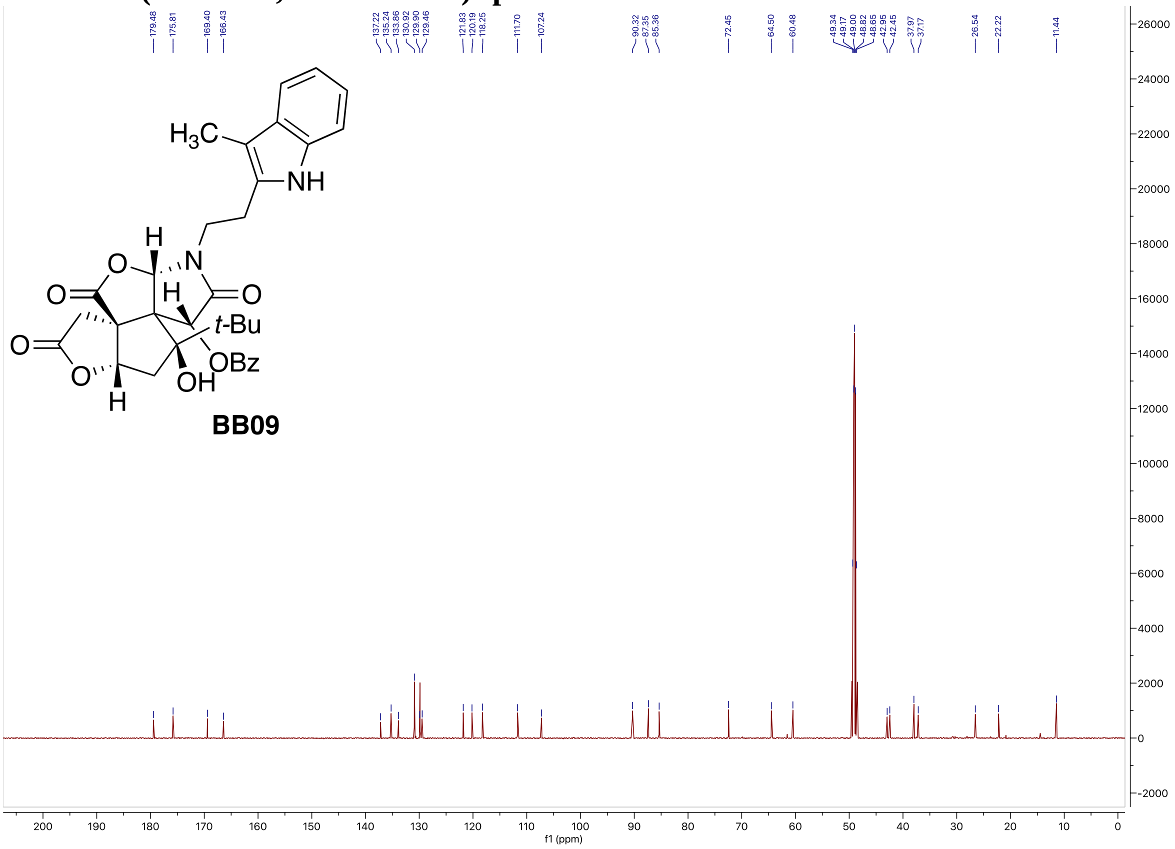

^{1}H NMR (700 MHz, Methanol-d_4) spectrum of BB10

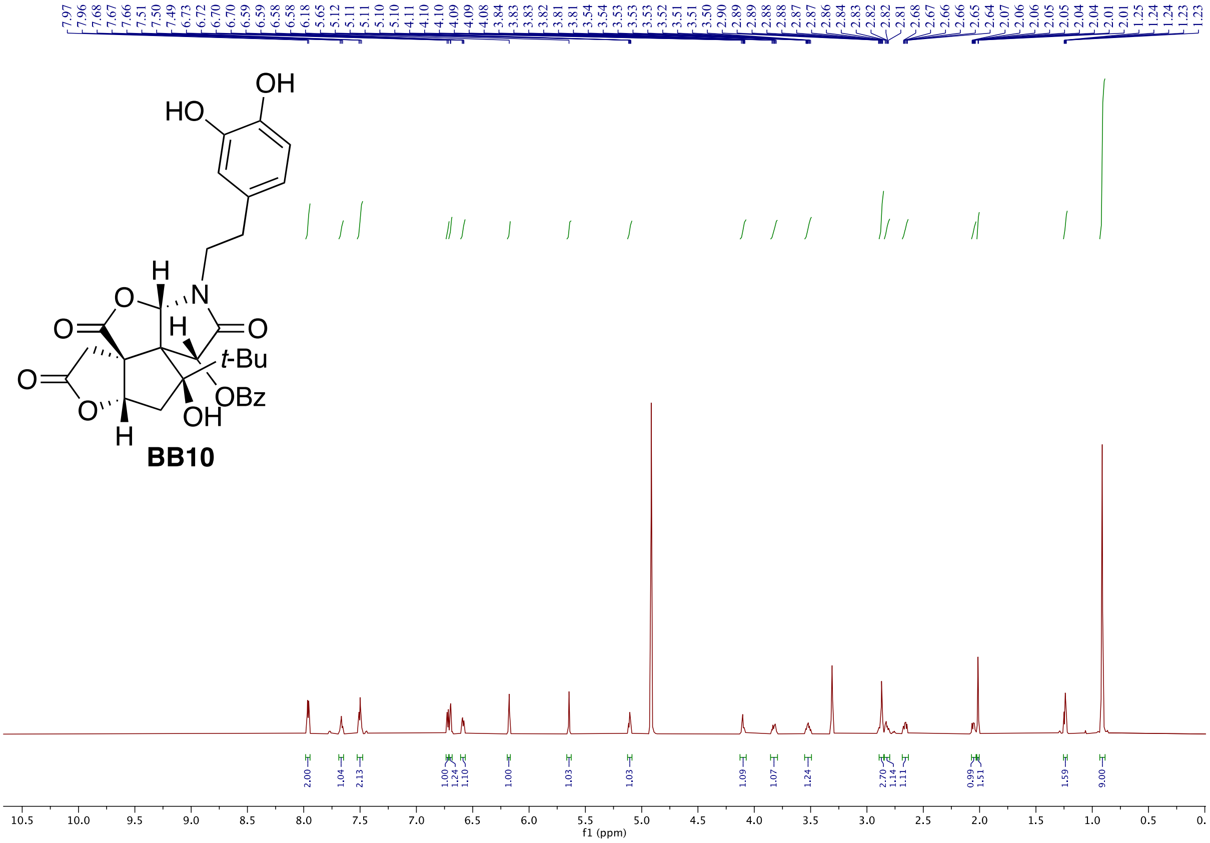

^{13}C NMR (126 MHz, $CDCl_3$) spectrum of BB10

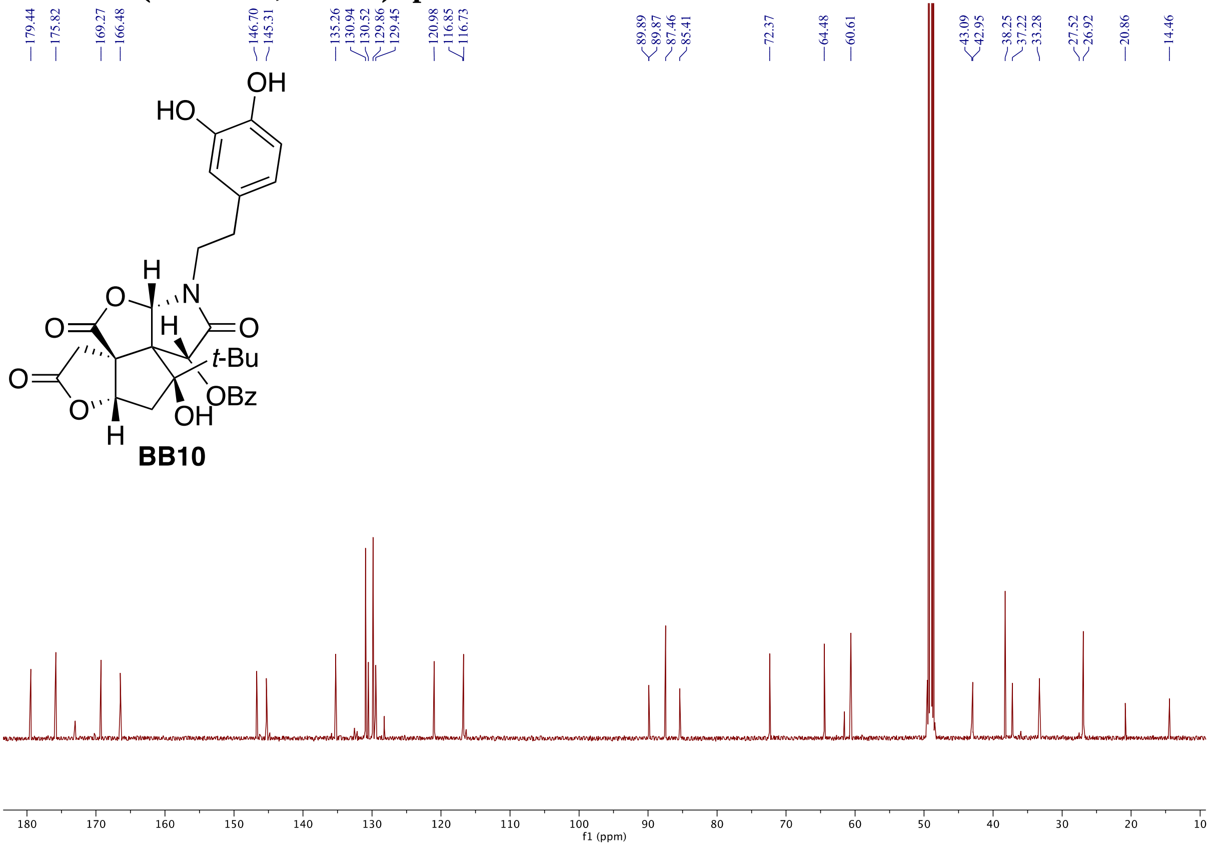

^{1}H NMR (500 MHz, CDCl$_3$) spectrum of BB11

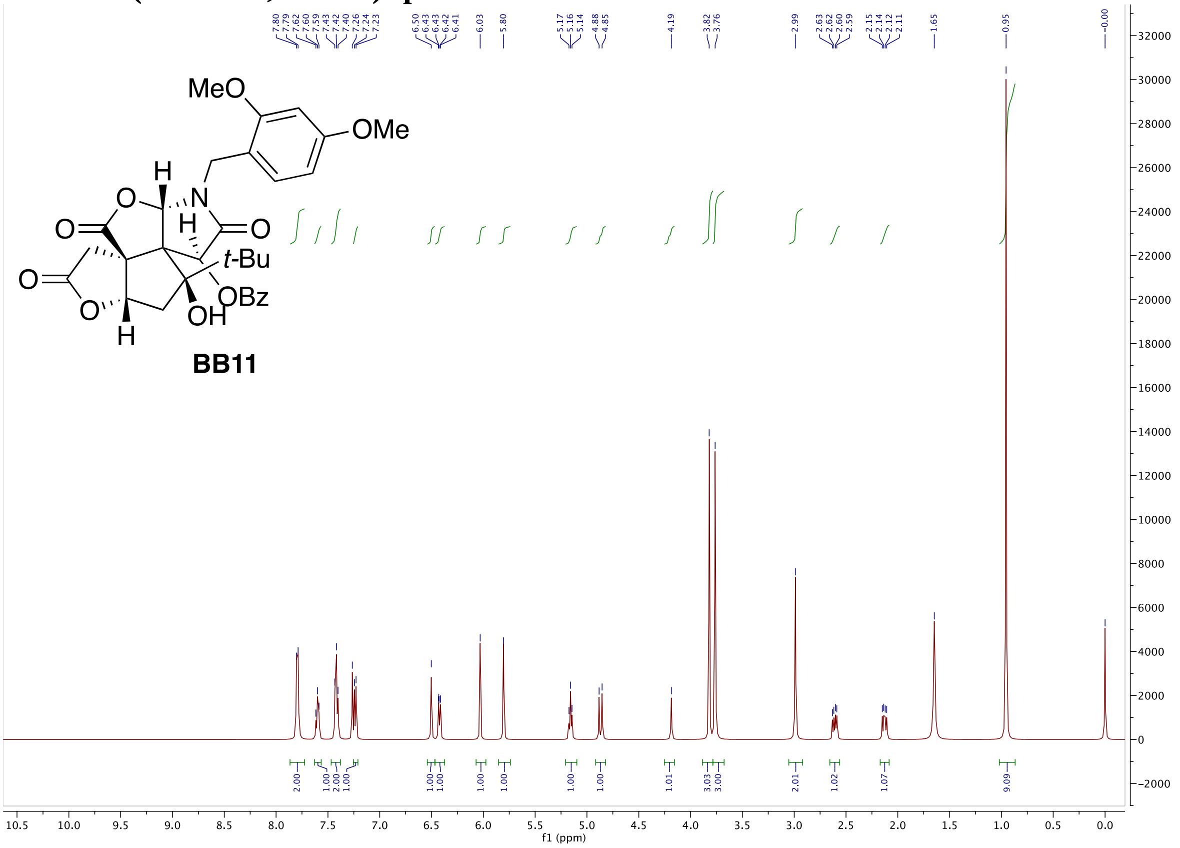

^{13}C NMR (126 MHz, CDCl$_3$) spectrum of BB11

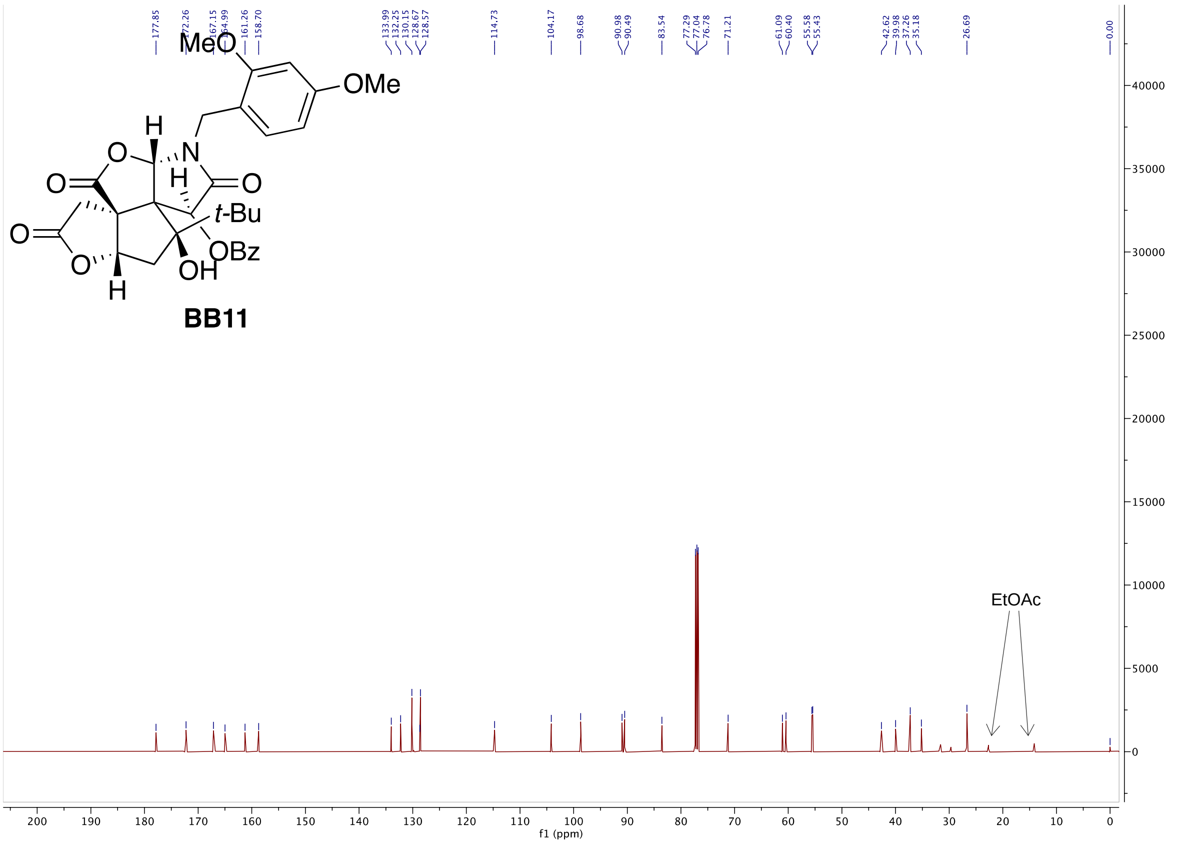

^{1}H NMR (500 MHz, $CDCl_3$) spectrum of BB12

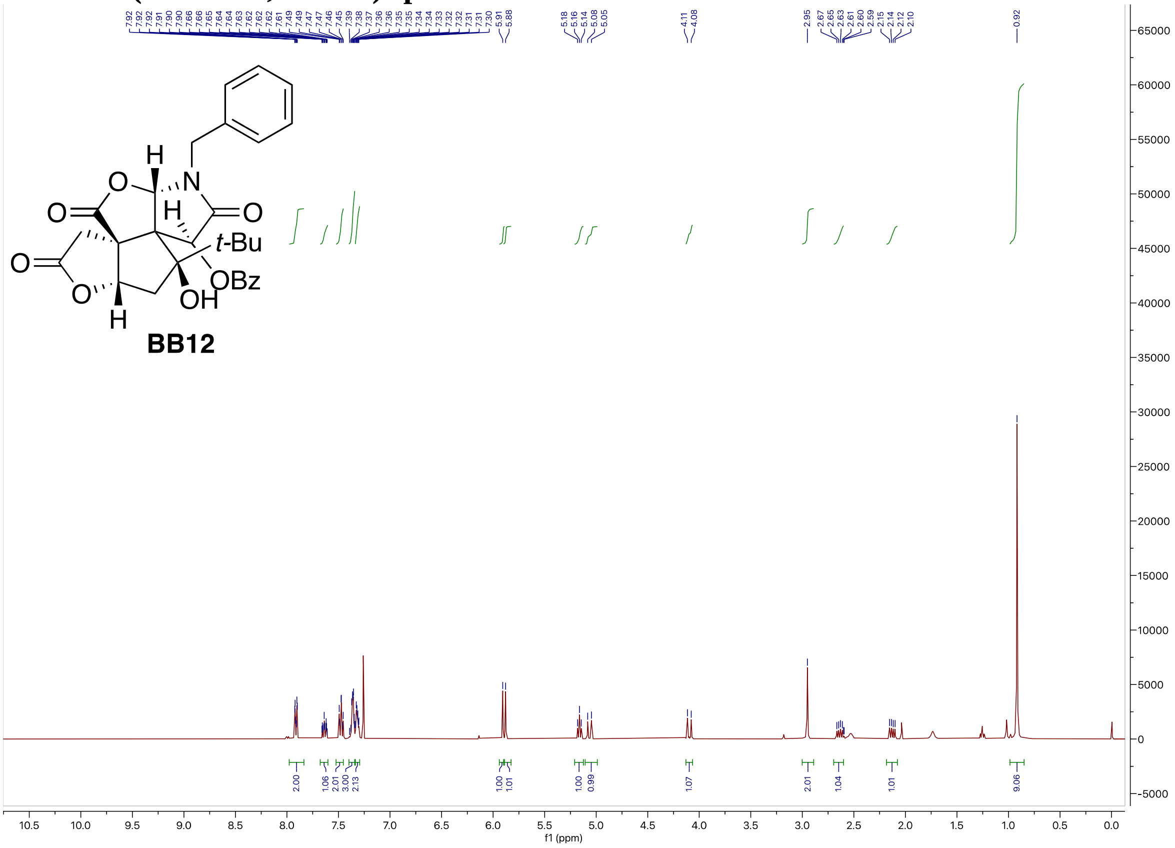

^{13}C NMR (126 MHz, $CDCl_3$) spectrum of BB12

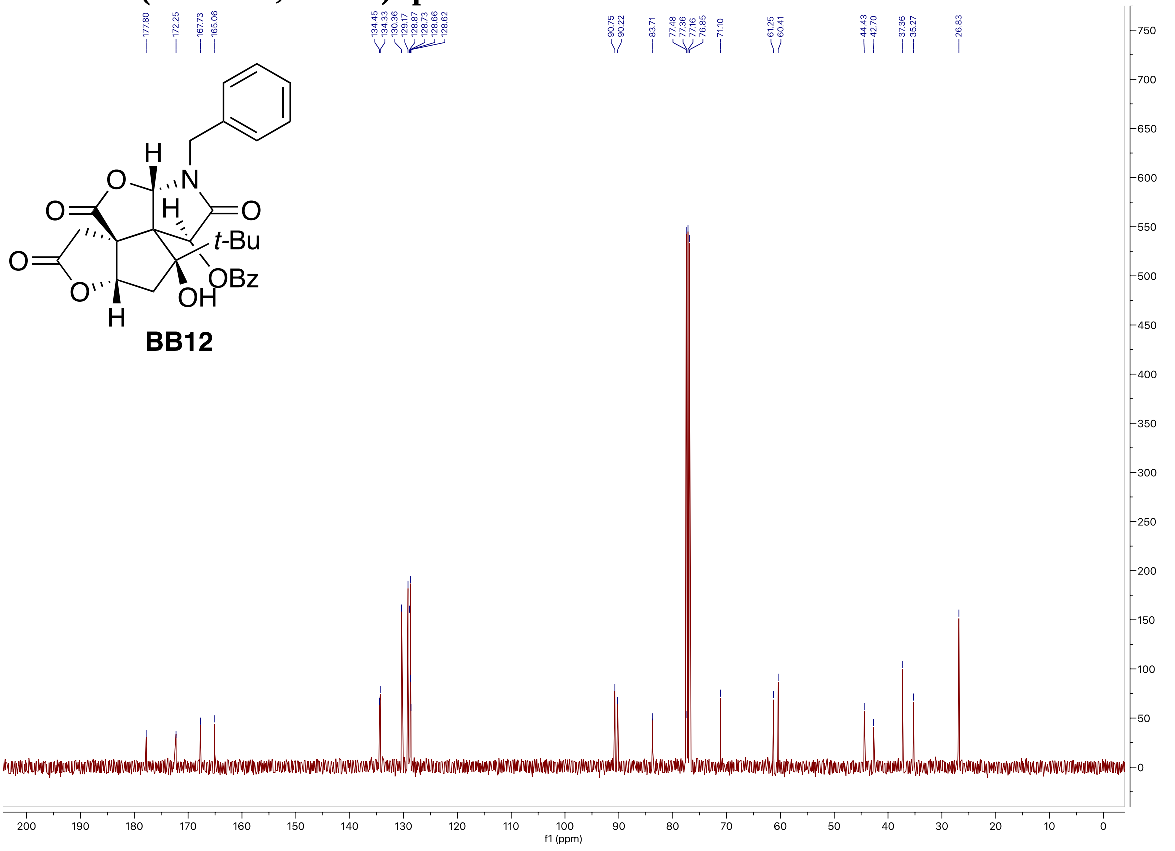

^{1}H NMR (600 MHz, $CDCl_3$) spectrum of BB13

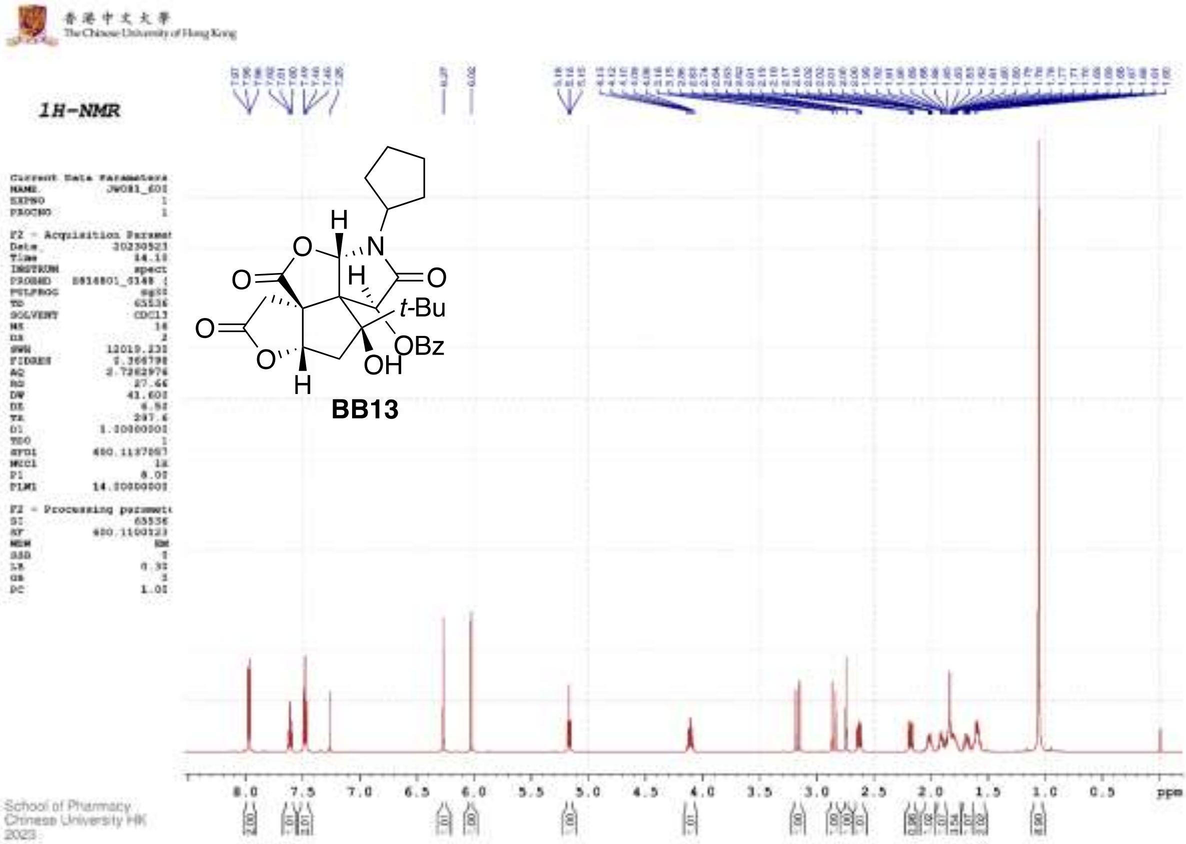

^{13}C NMR (150 MHz, $CDCl_3$) spectrum of BB13

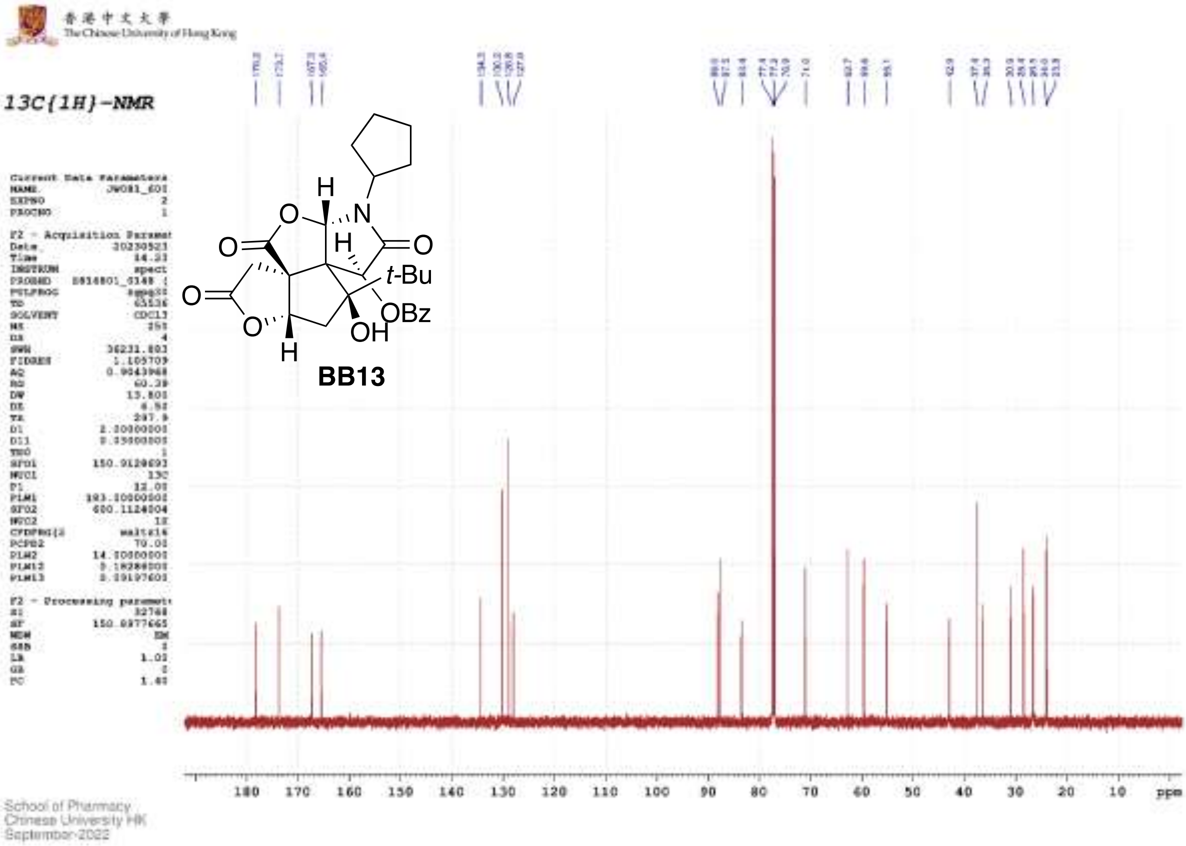

^{1}H NMR (400 MHz, Methanol-d_4) spectrum of BB14

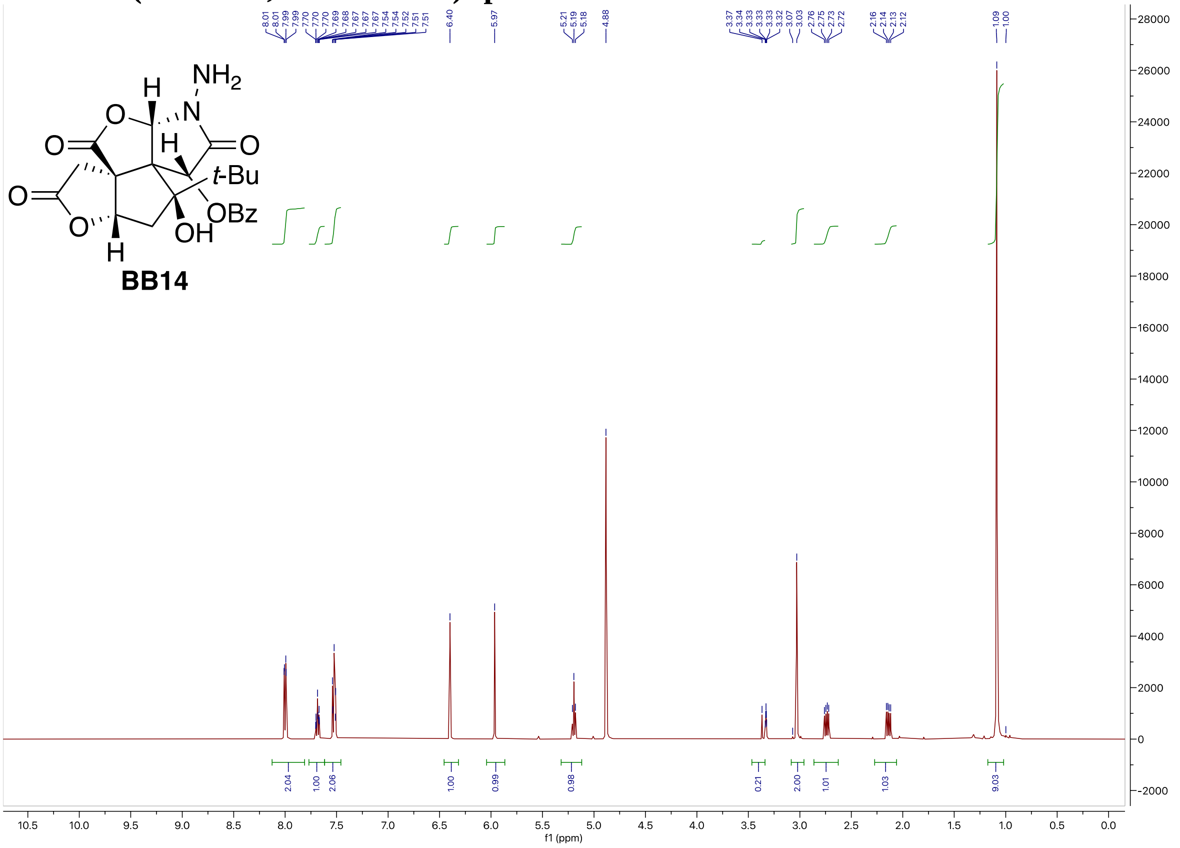

^{13}C NMR (100 MHz, Methanol-d_4) spectrum of BB14

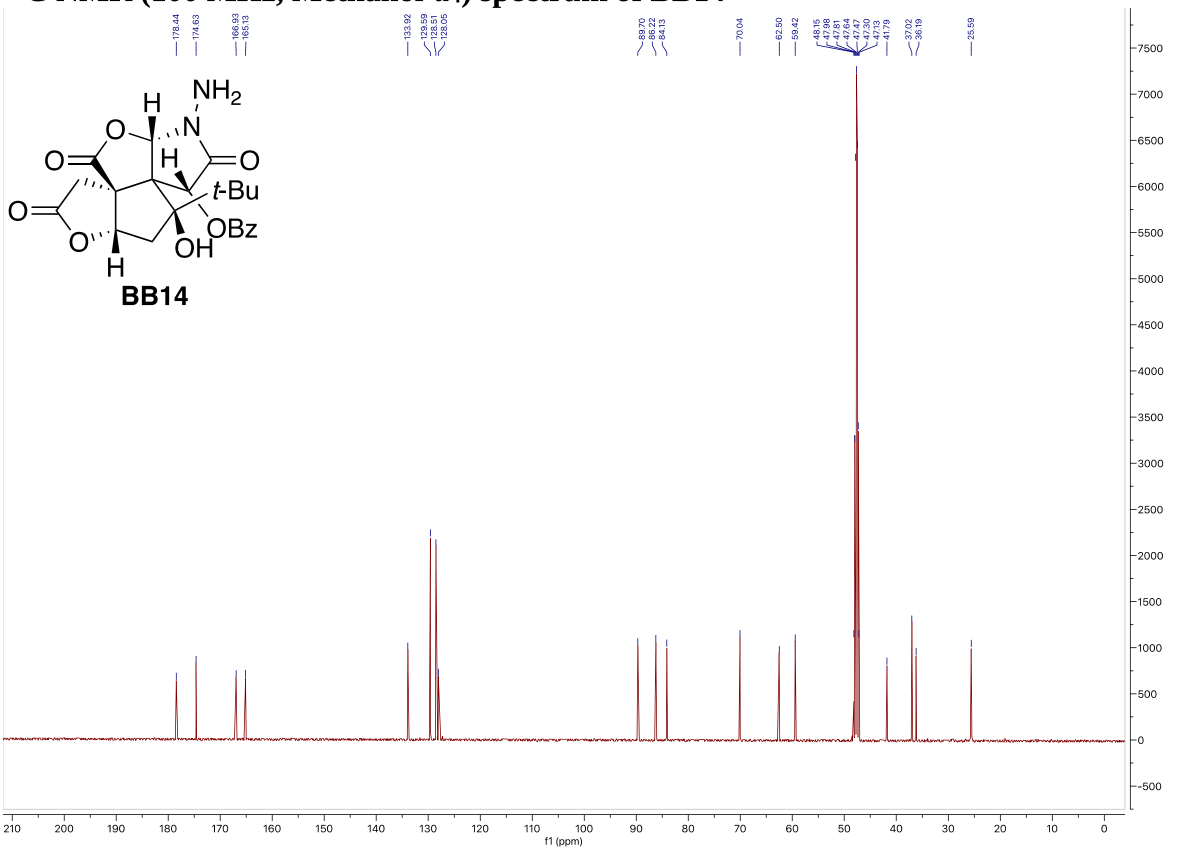

HSQC (126 MHz, Methanol-d_4) spectrum of BB14

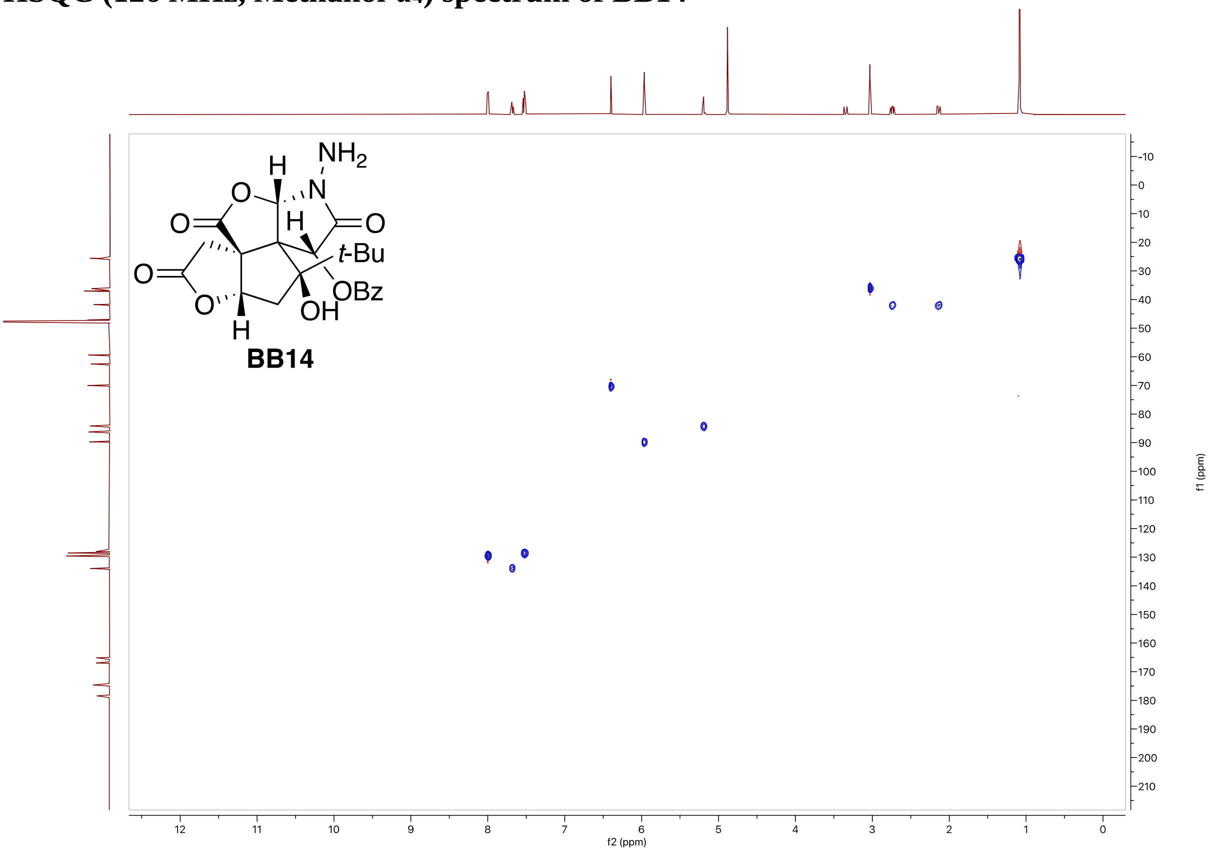

HMBC (126 MHz, Methanol-d_4) spectrum of BB14

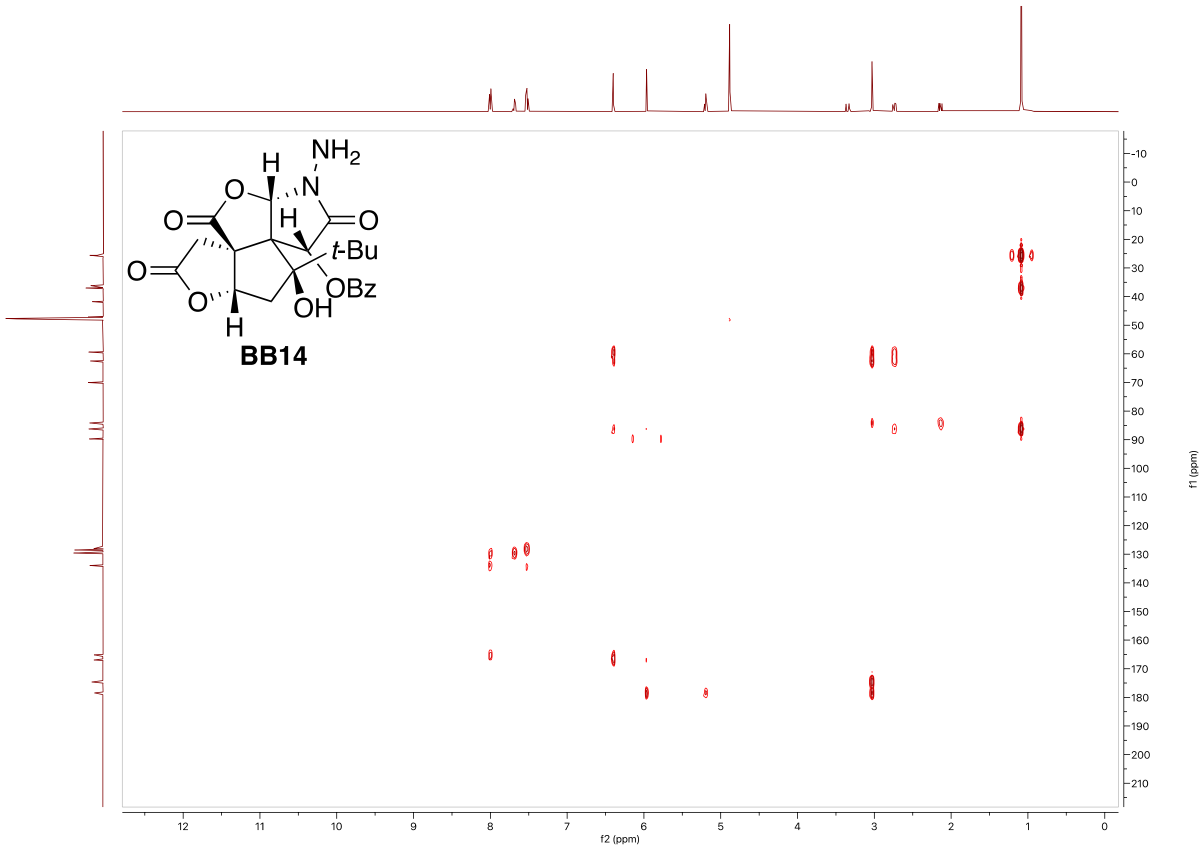

^{1}H NMR (600 MHz, Methanol-d_4) spectrum of BB15

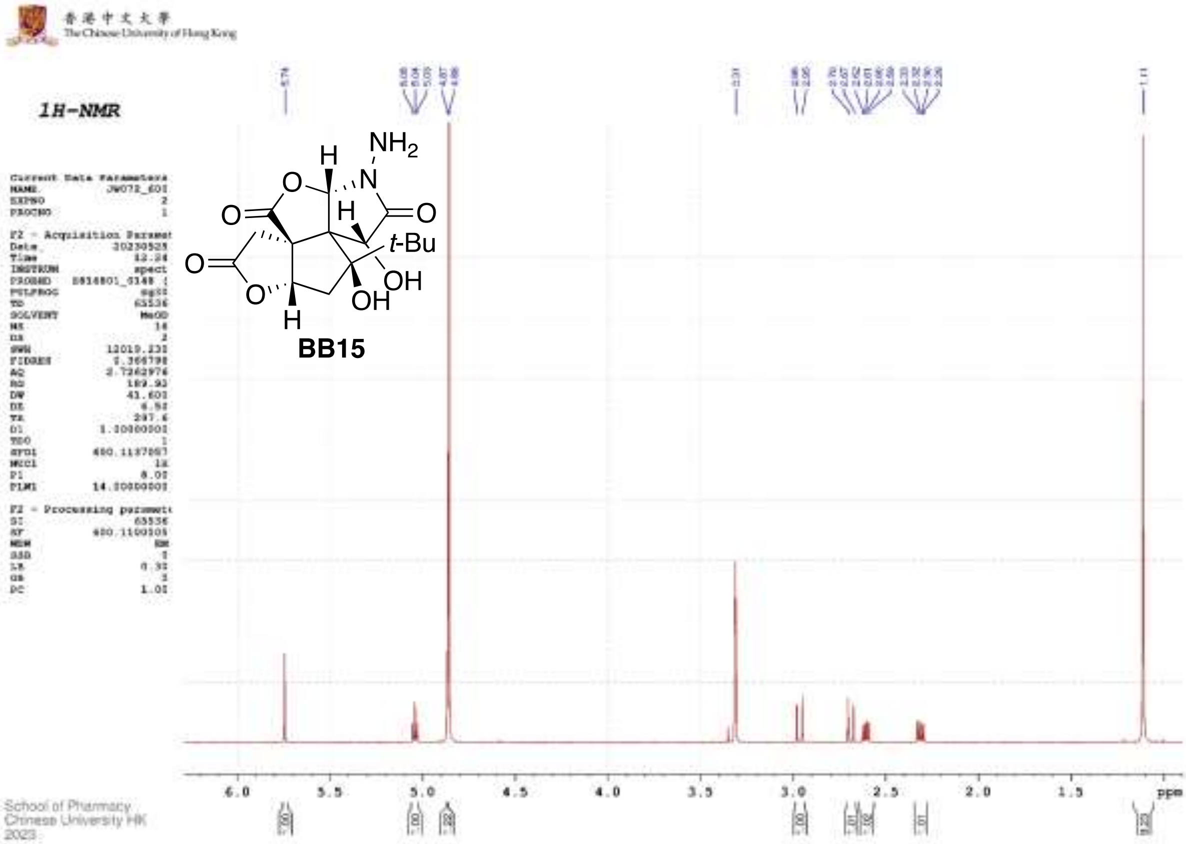

HSQC (150 MHz, Methanol-d_4) spectrum of BB15

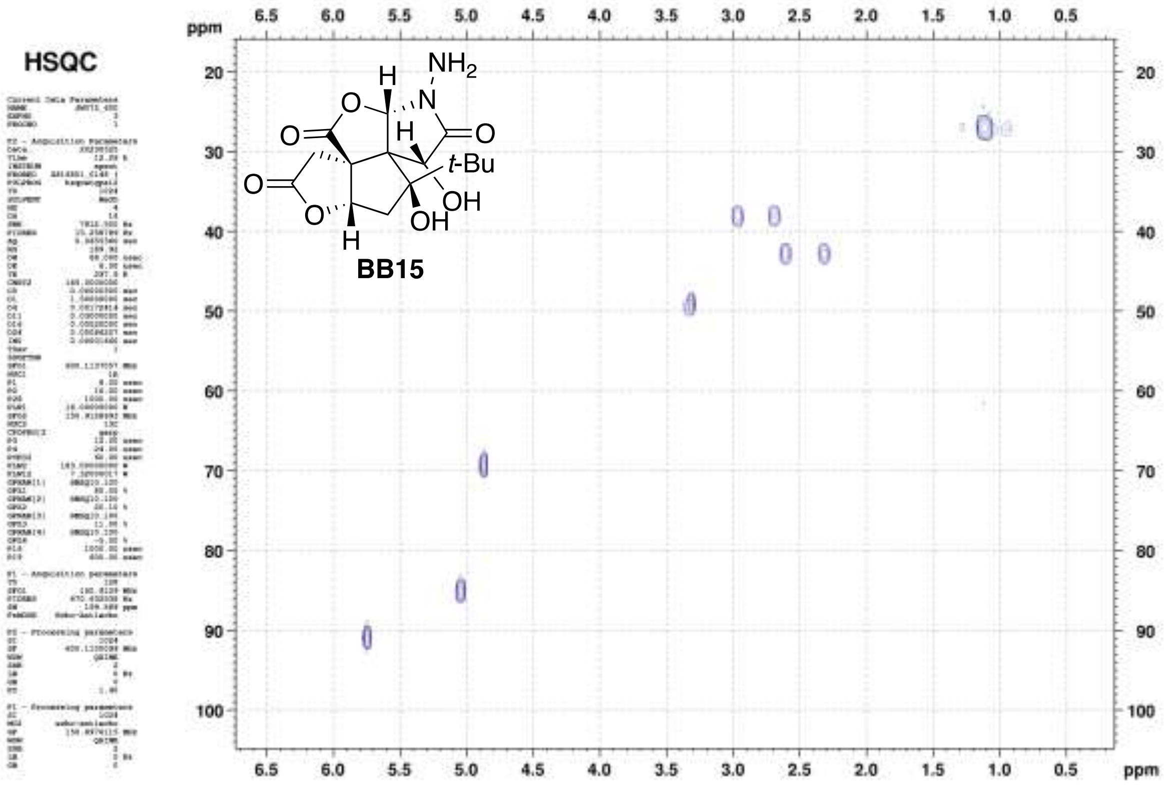

HMBC (150 MHz, Methanol-d_4) spectrum of BB15

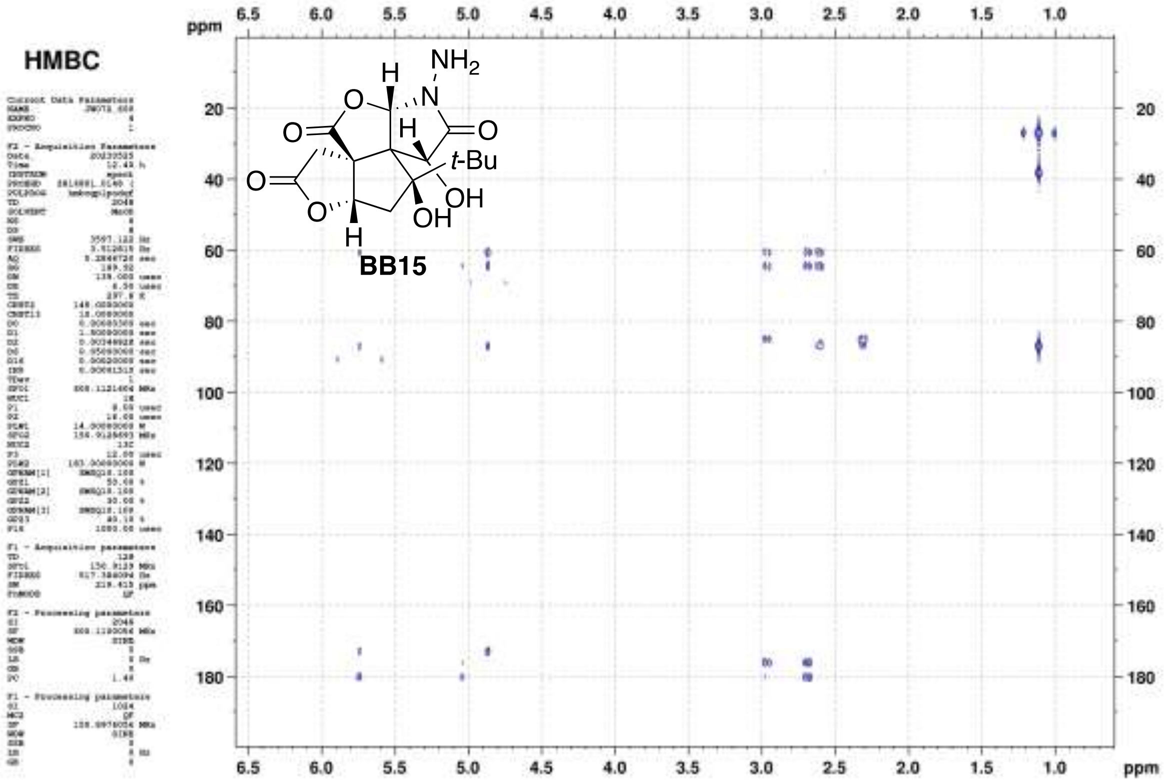

^{1}H NMR (700 MHz, $CDCl_3$) spectrum of 3

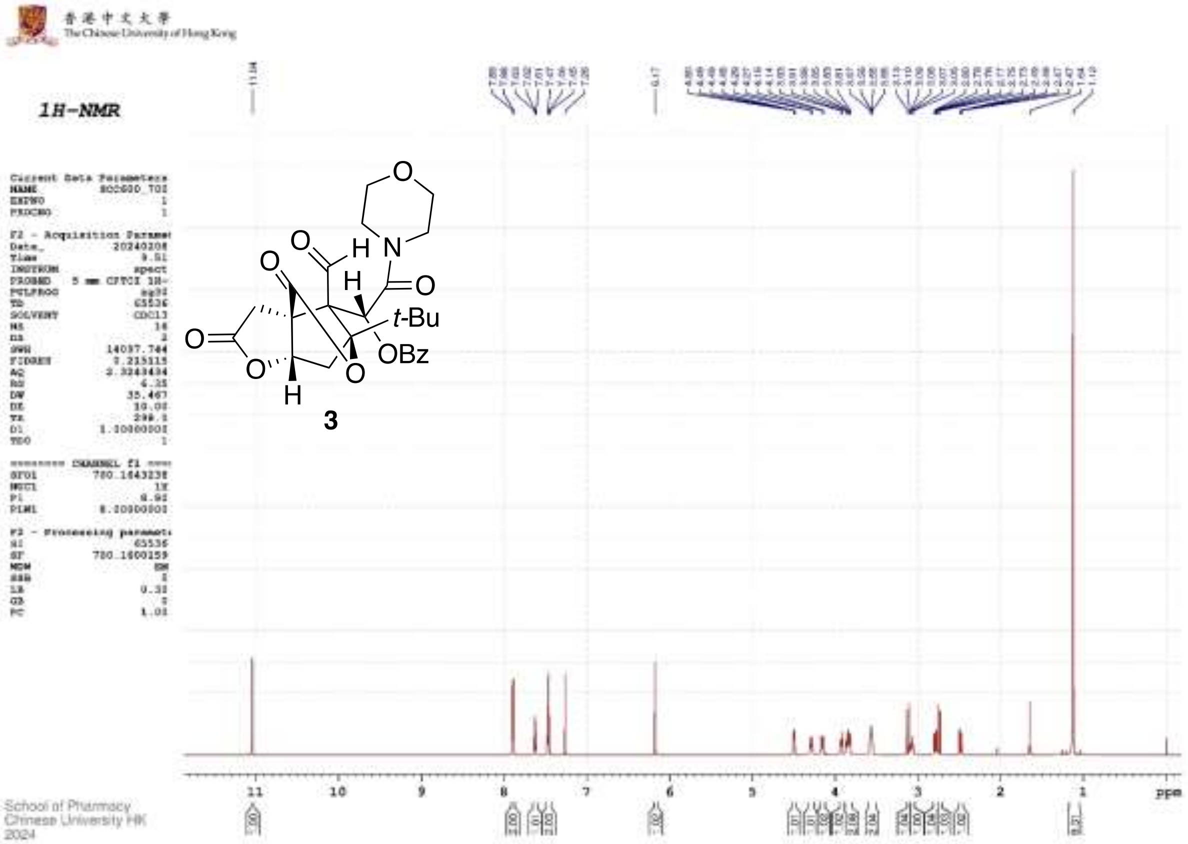

^{13}C NMR (175 MHz, $CDCl_3$) spectrum of 3

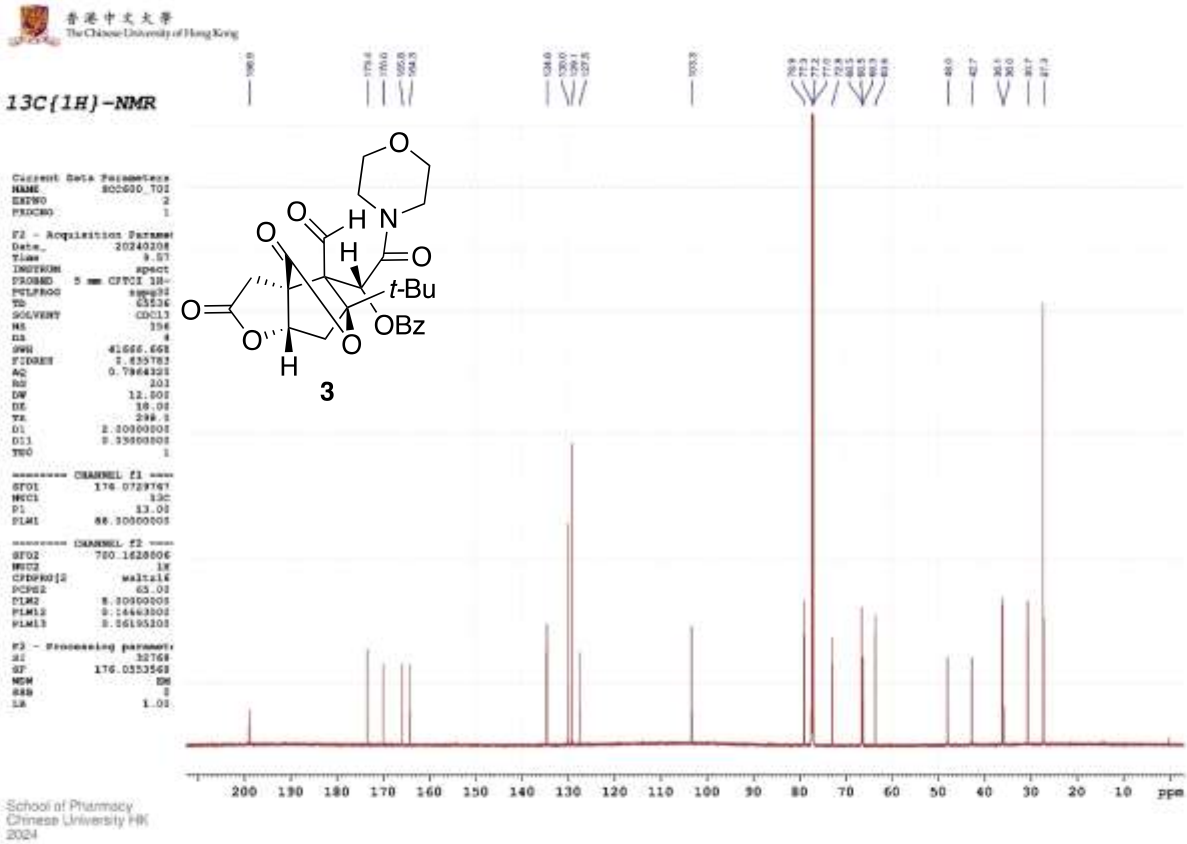

^{1}H NMR (500 MHz, Methanol-d_4) spectrum of BB16

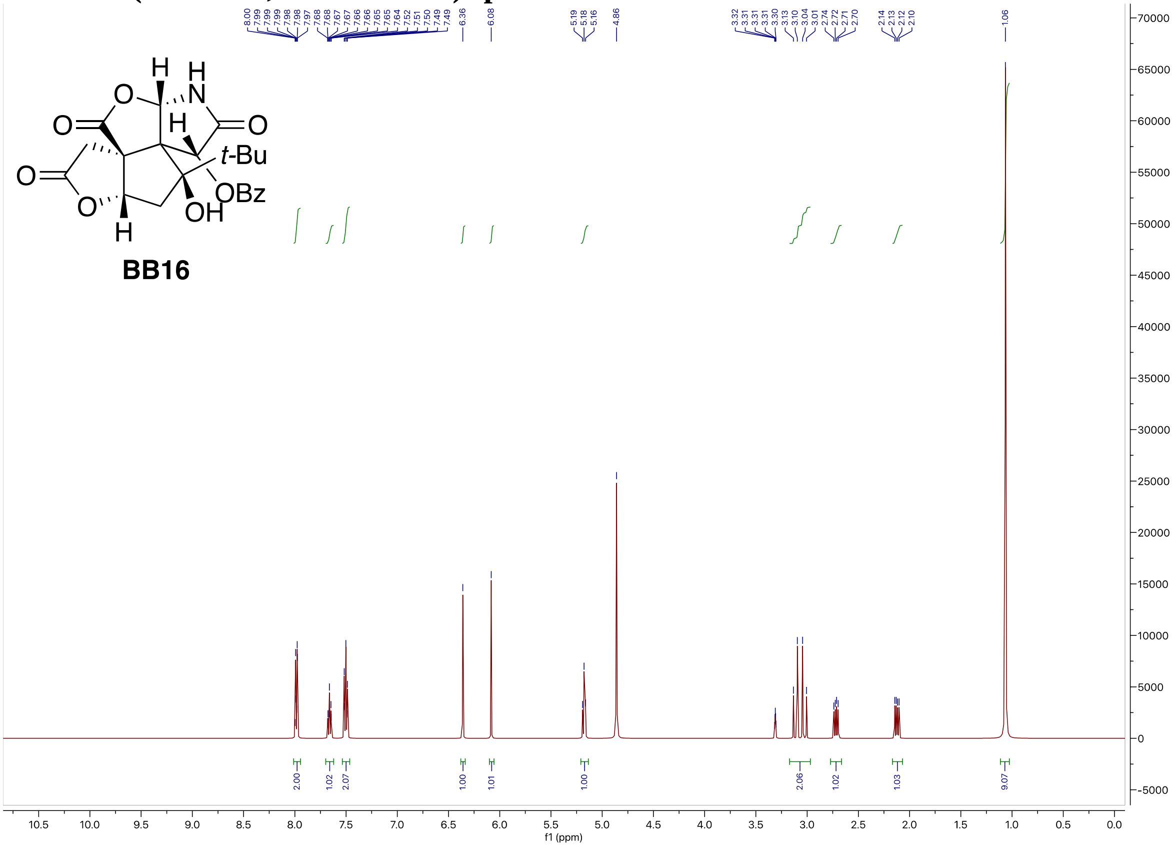

^{13}C NMR (126 MHz, Methanol-d_4) spectrum of BB16

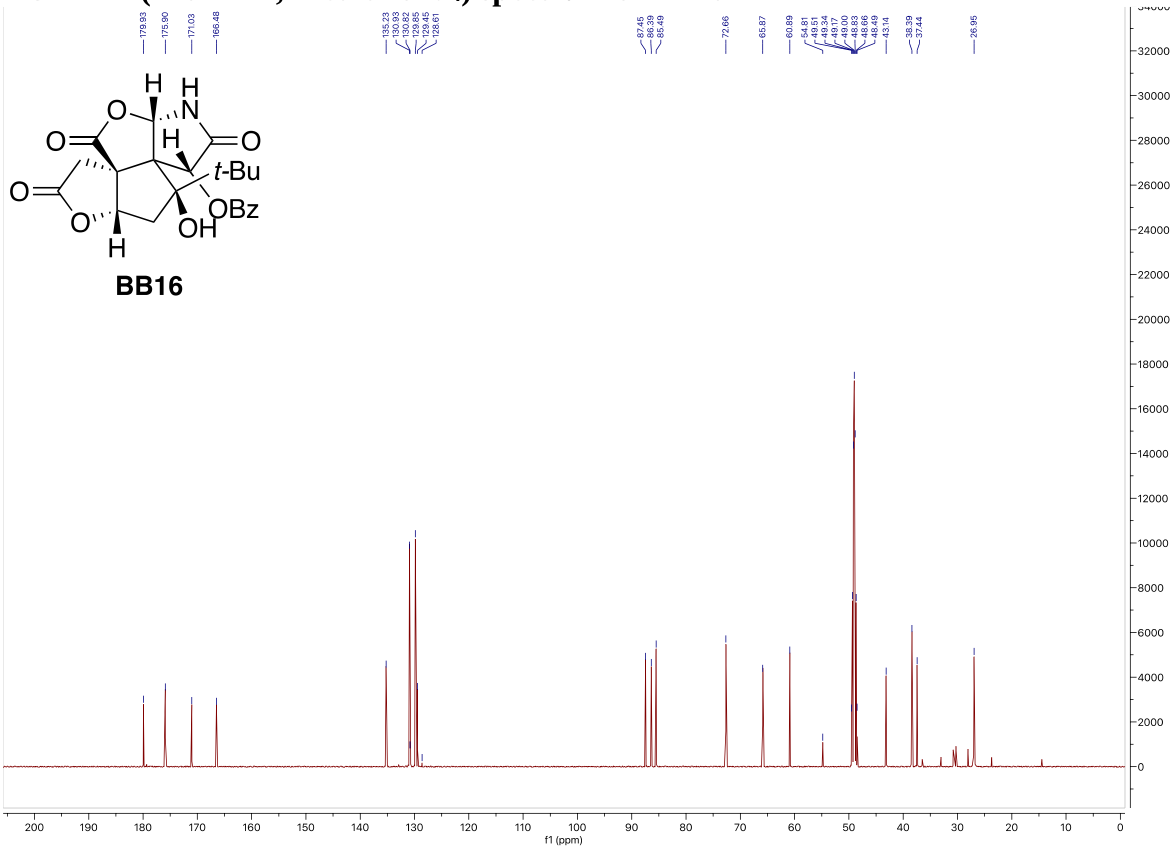

^{1}H NMR (500 MHz, DMSO-d_6) spectrum of BB17

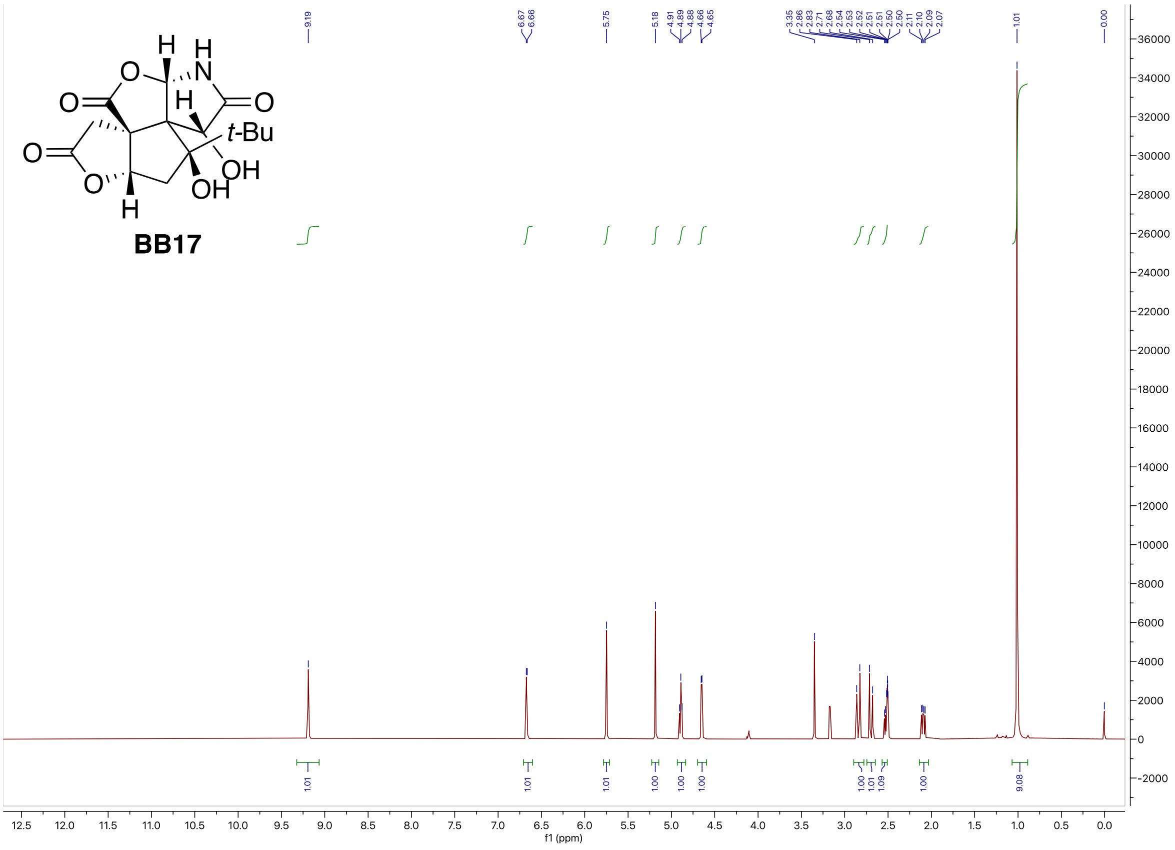

^{13}C NMR (126 MHz, DMSO-d_6) spectrum of BB17

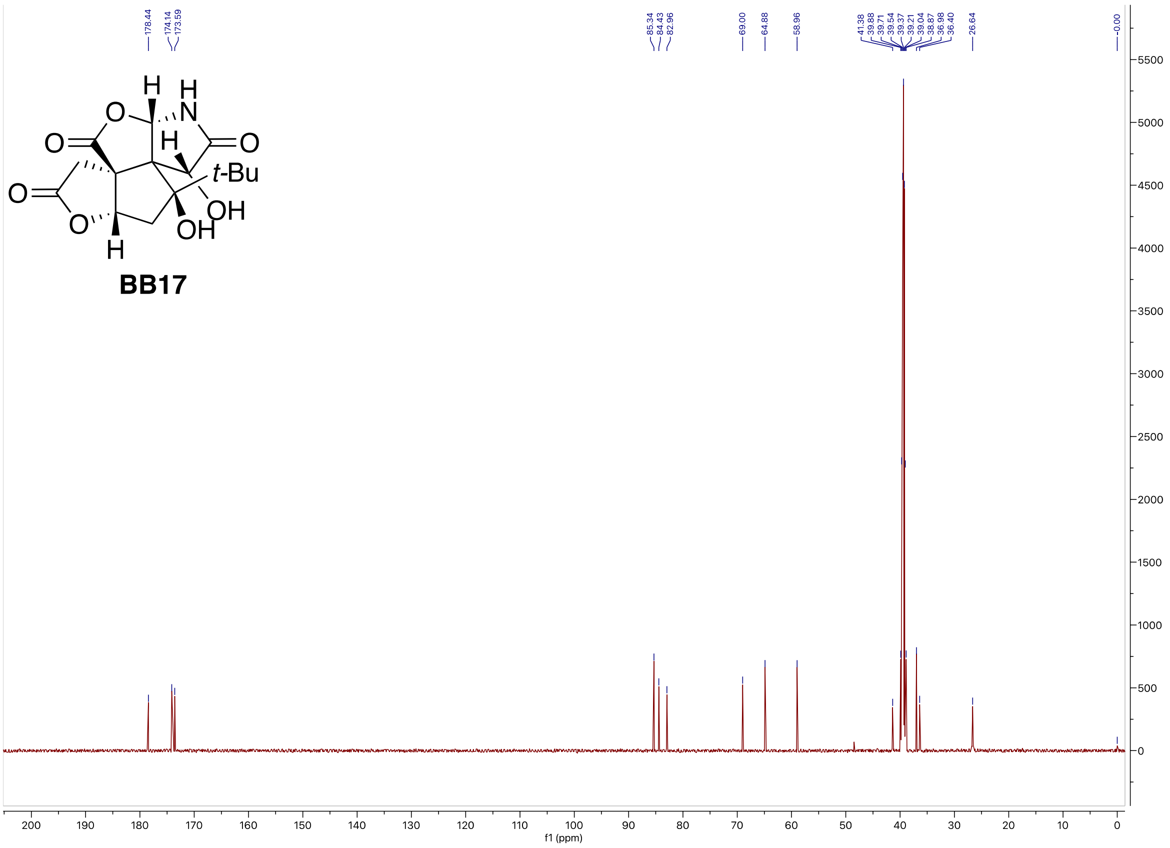

^{1}H NMR (400 MHz, $CDCl_3$) spectrum of BB18

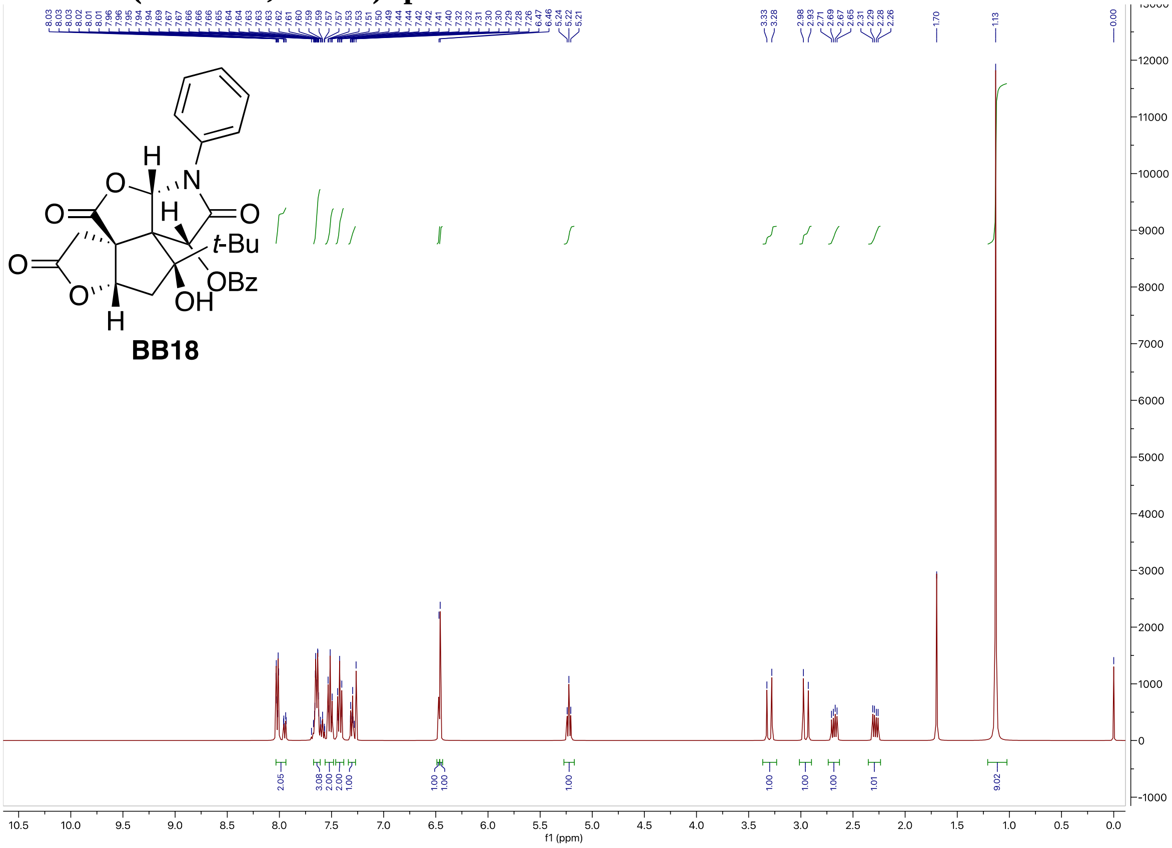

^{13}C NMR (100 MHz, $CDCl_3$) spectrum of BB18

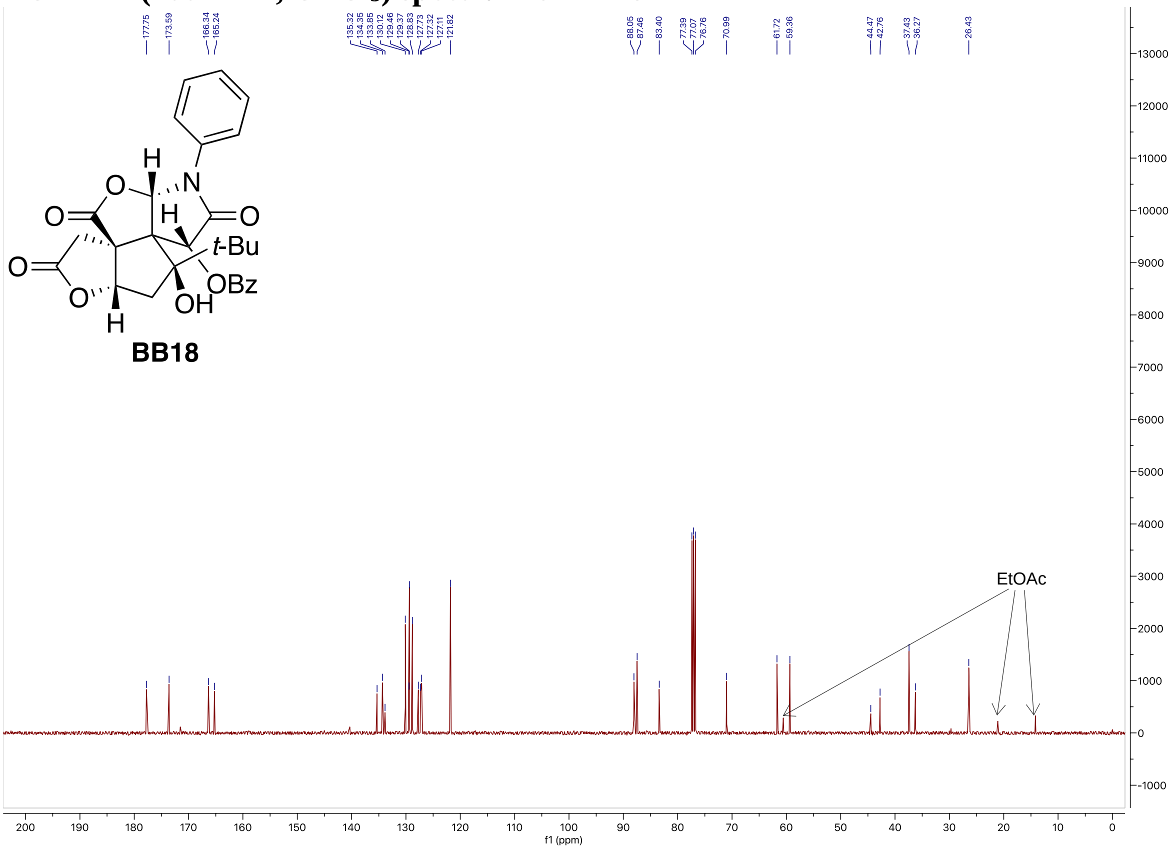

^{1}H NMR (600 MHz, Methanol-d_4) spectrum of BB19

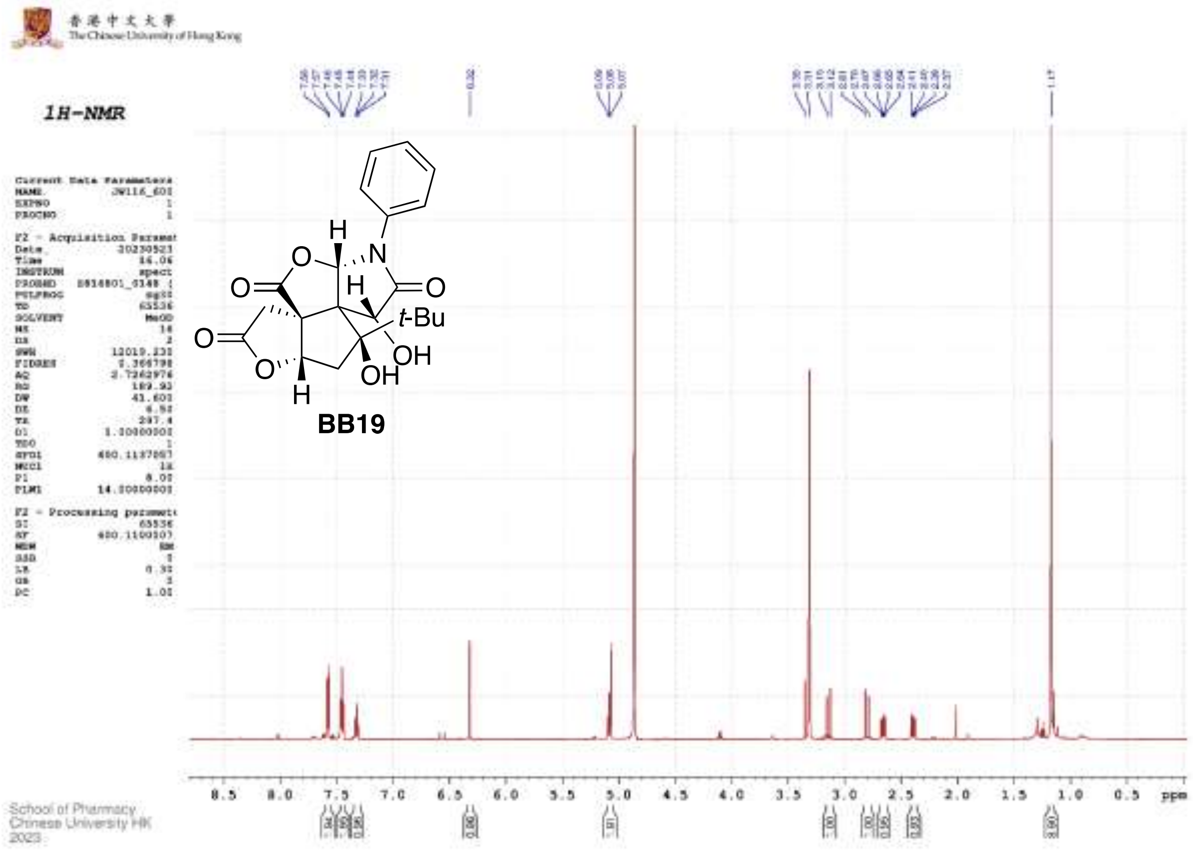

HSQC (150 MHz, Methanol-d_4) spectrum of BB19

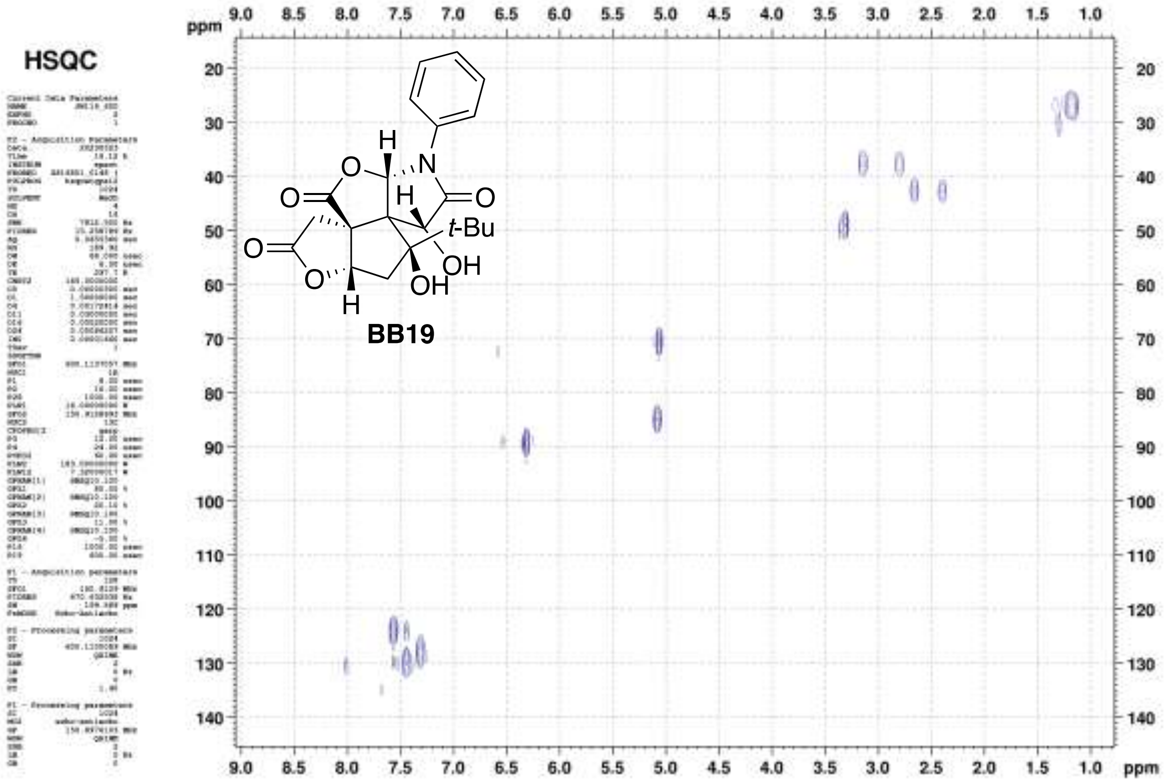

HMBC (150 MHz, Methanol-d_4) spectrum of BB19

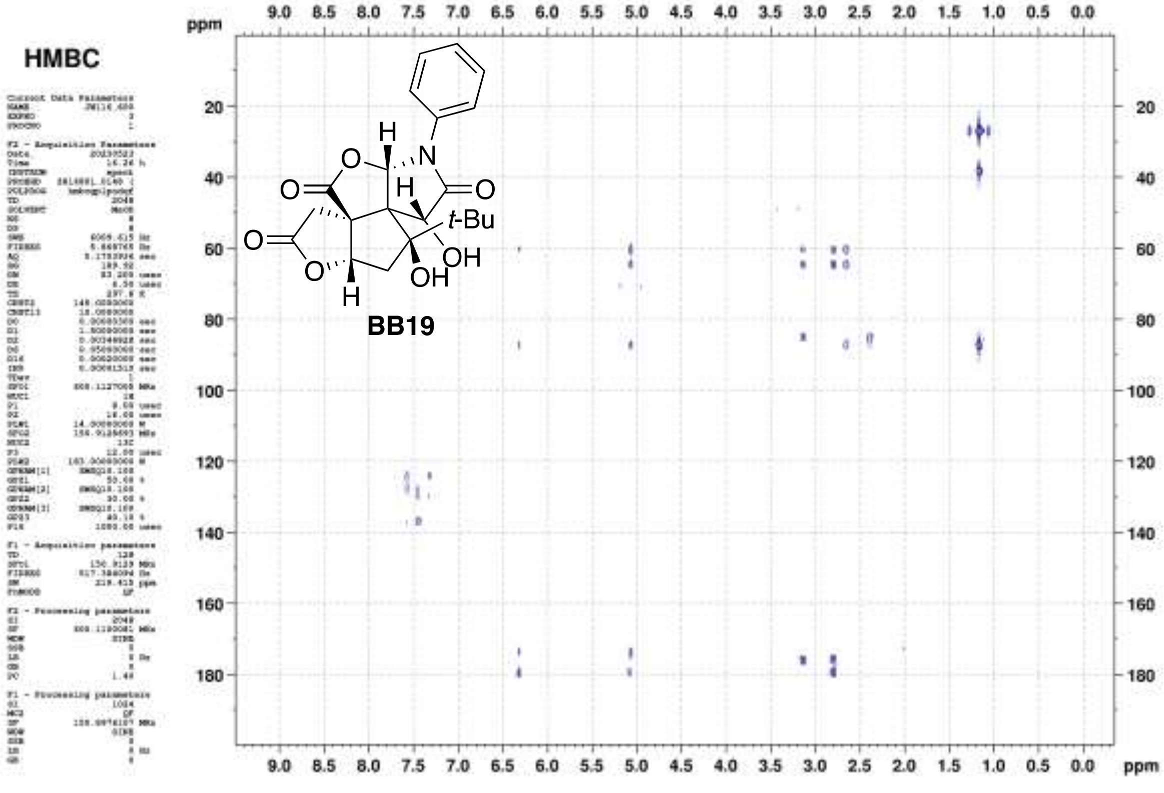

^{1}H NMR (500 MHz, $CDCl_3$) spectrum of BB20

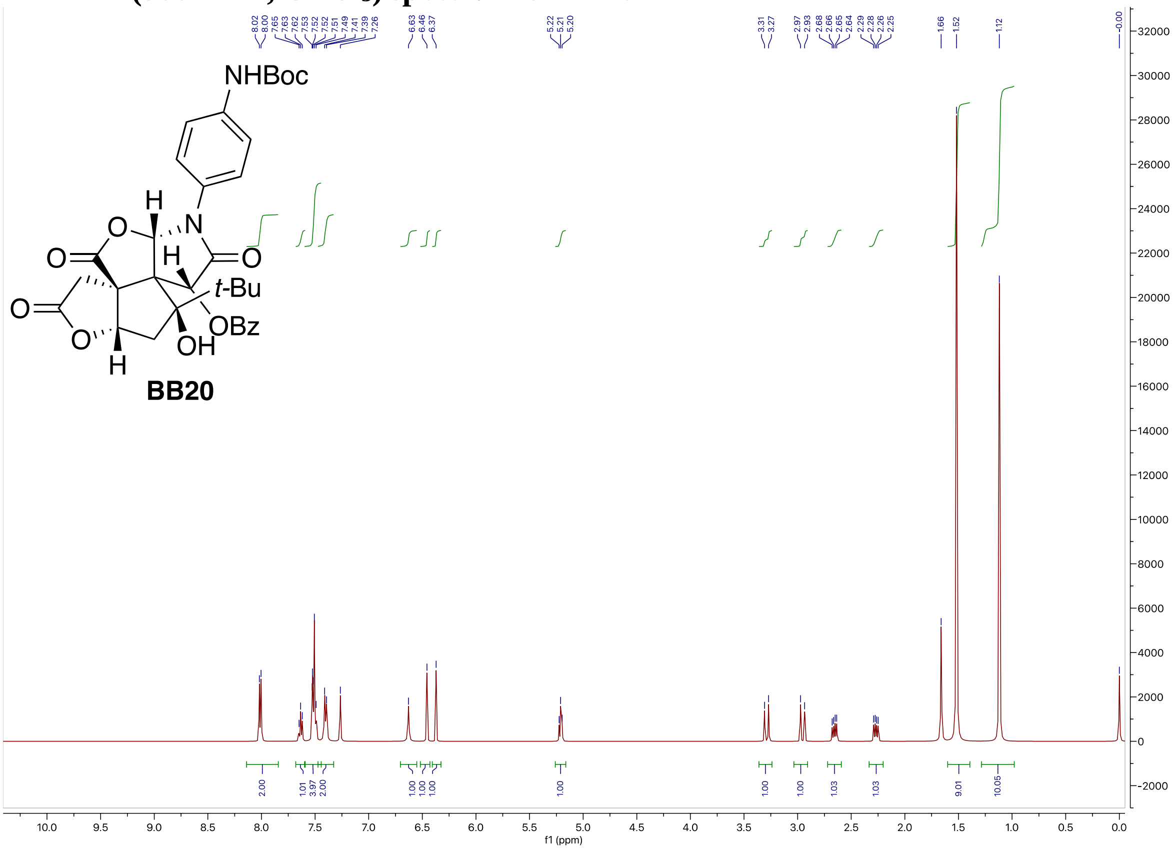

^{13}C NMR (126 MHz, $CDCl_3$) spectrum of BB20

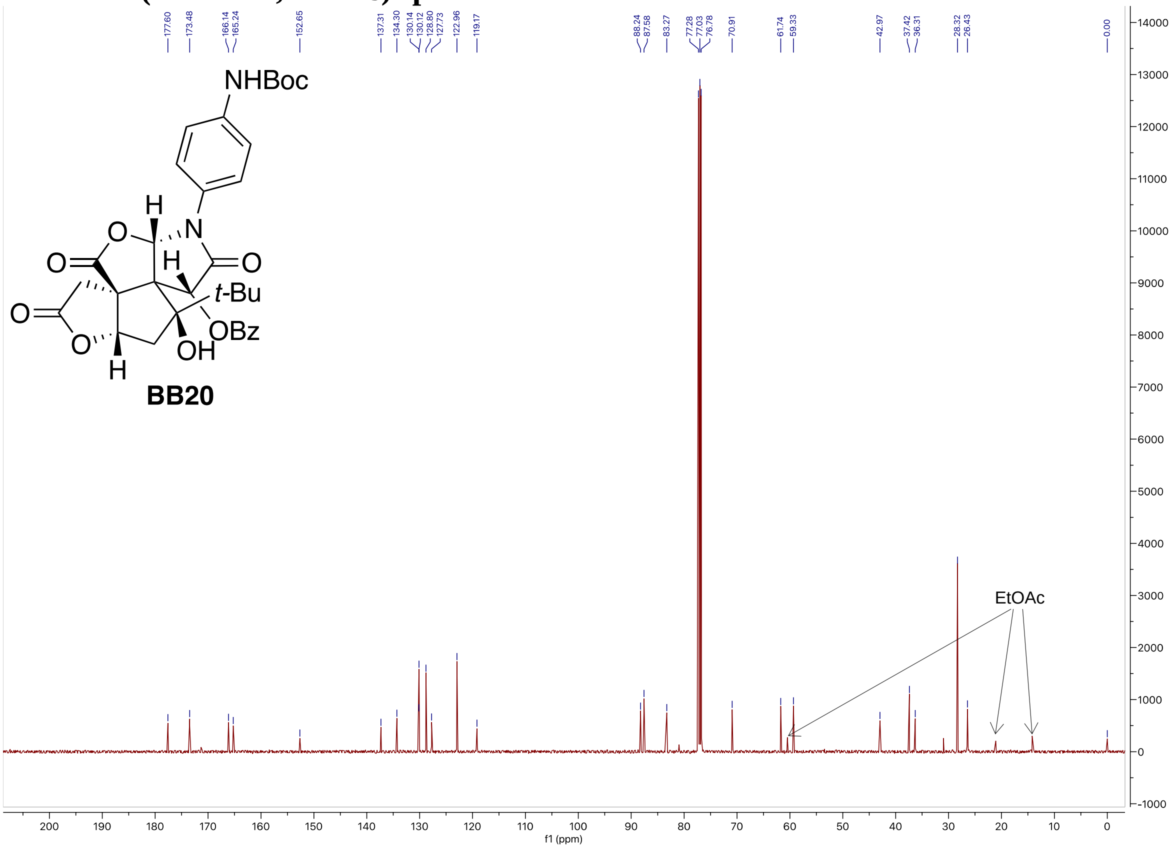

^{1}H NMR (400 MHz, Methanol-d_4) spectrum of BB21

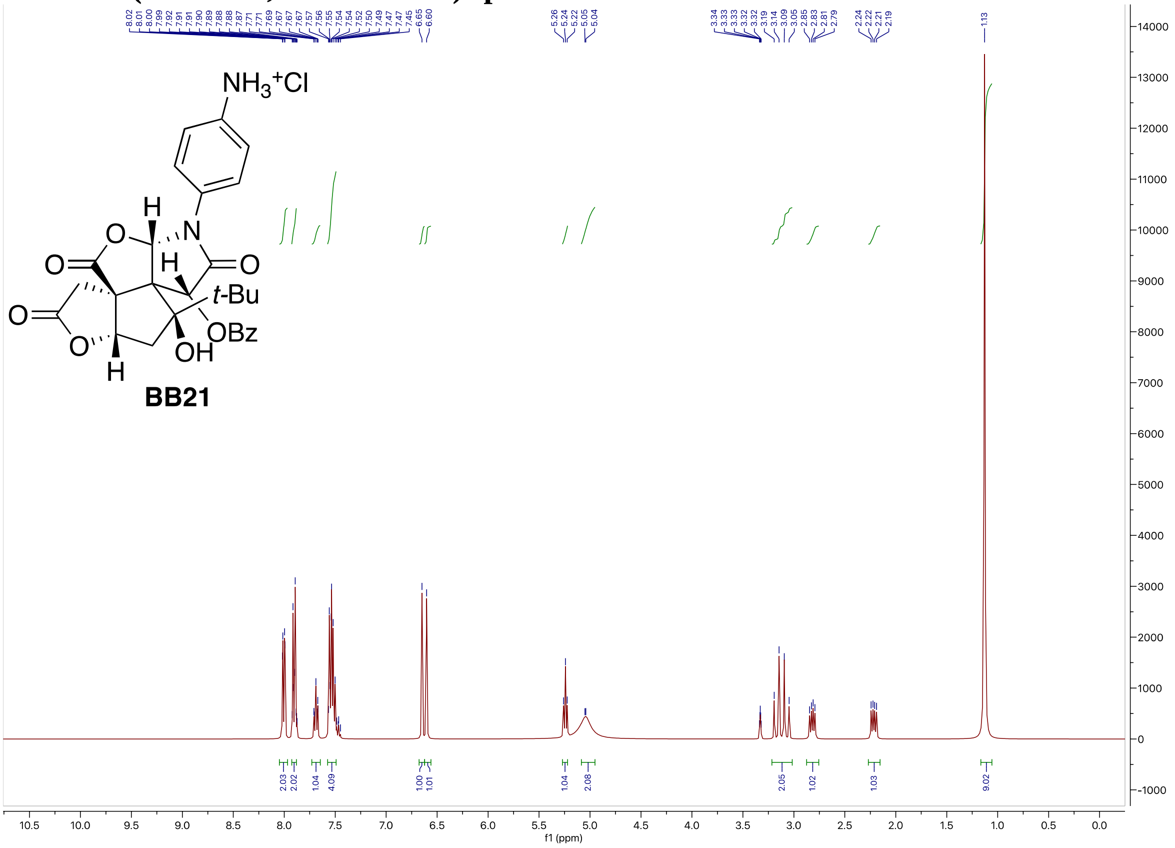

^{13}C NMR (101 MHz, Methanol-d_4) spectrum of BB21

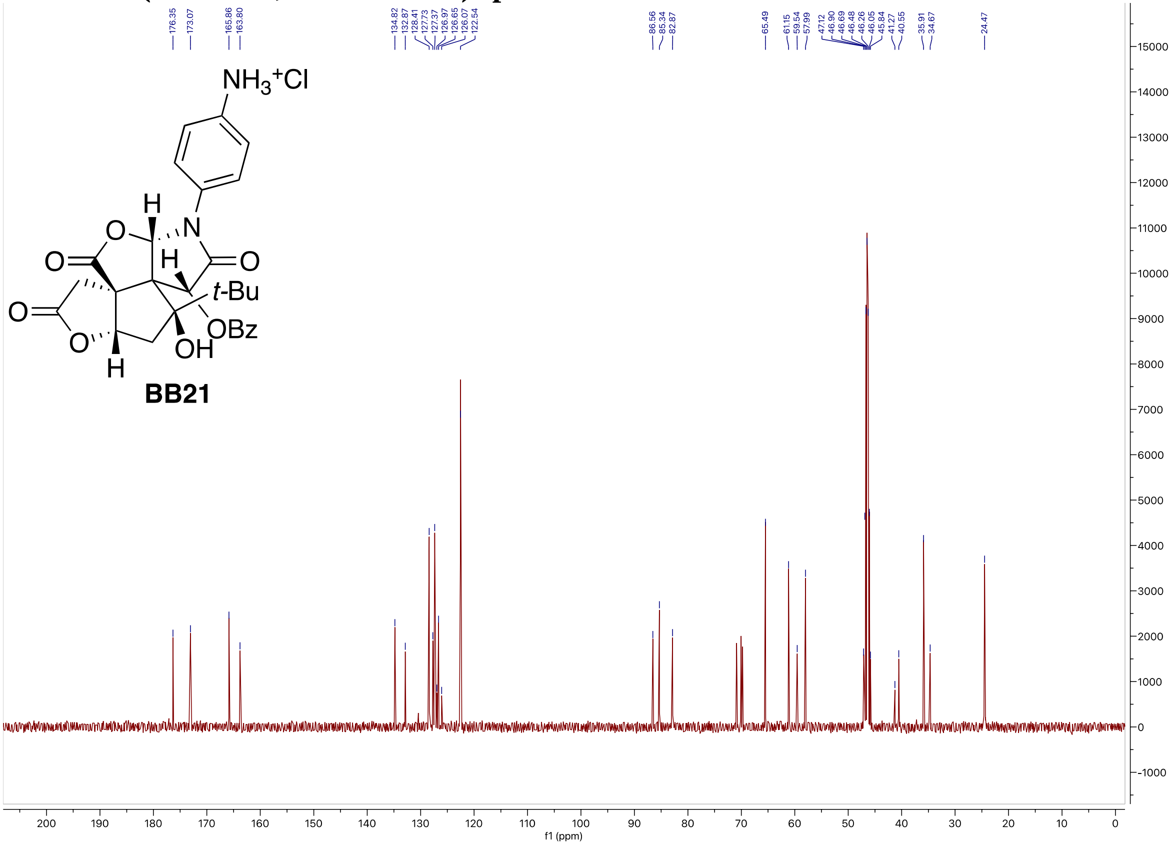

^{1}H NMR (600 MHz, $CDCl_3$) spectrum of BB22

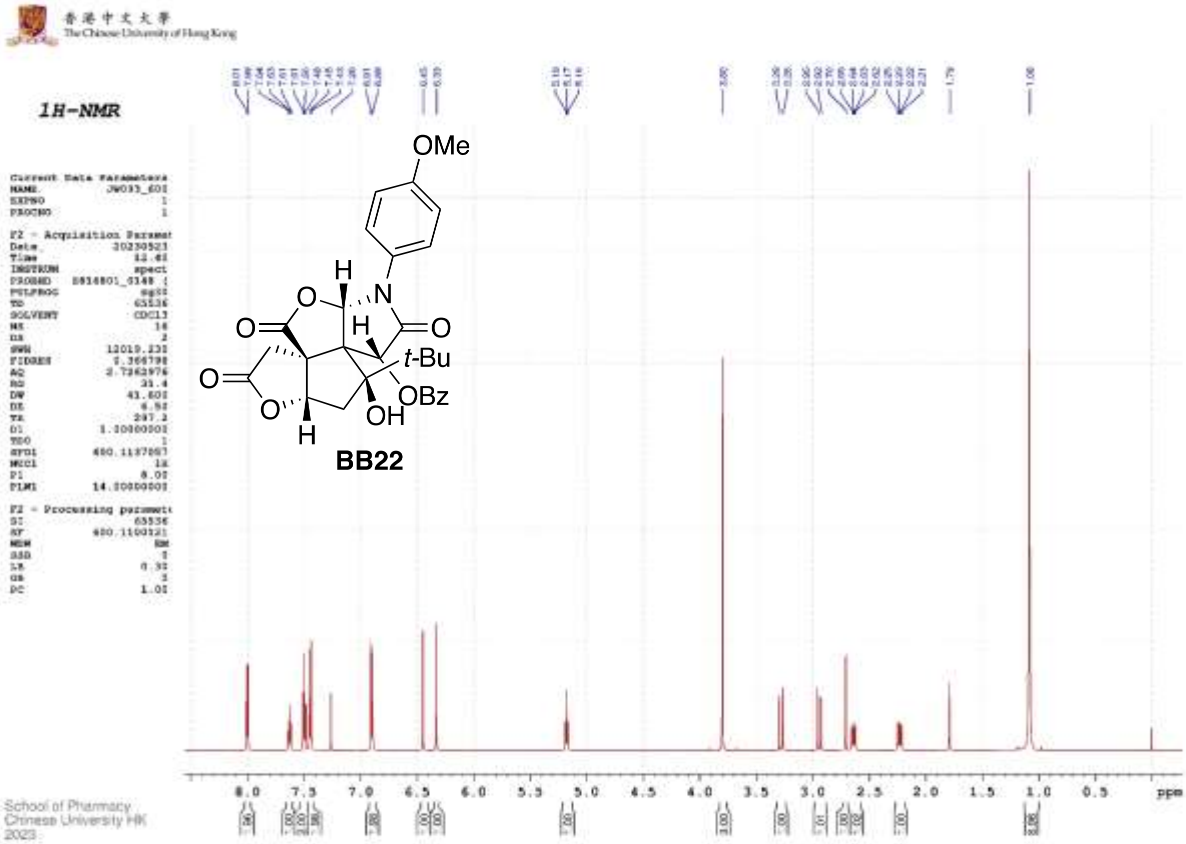

^{13}C NMR (150 MHz, $CDCl_3$) spectrum of BB22

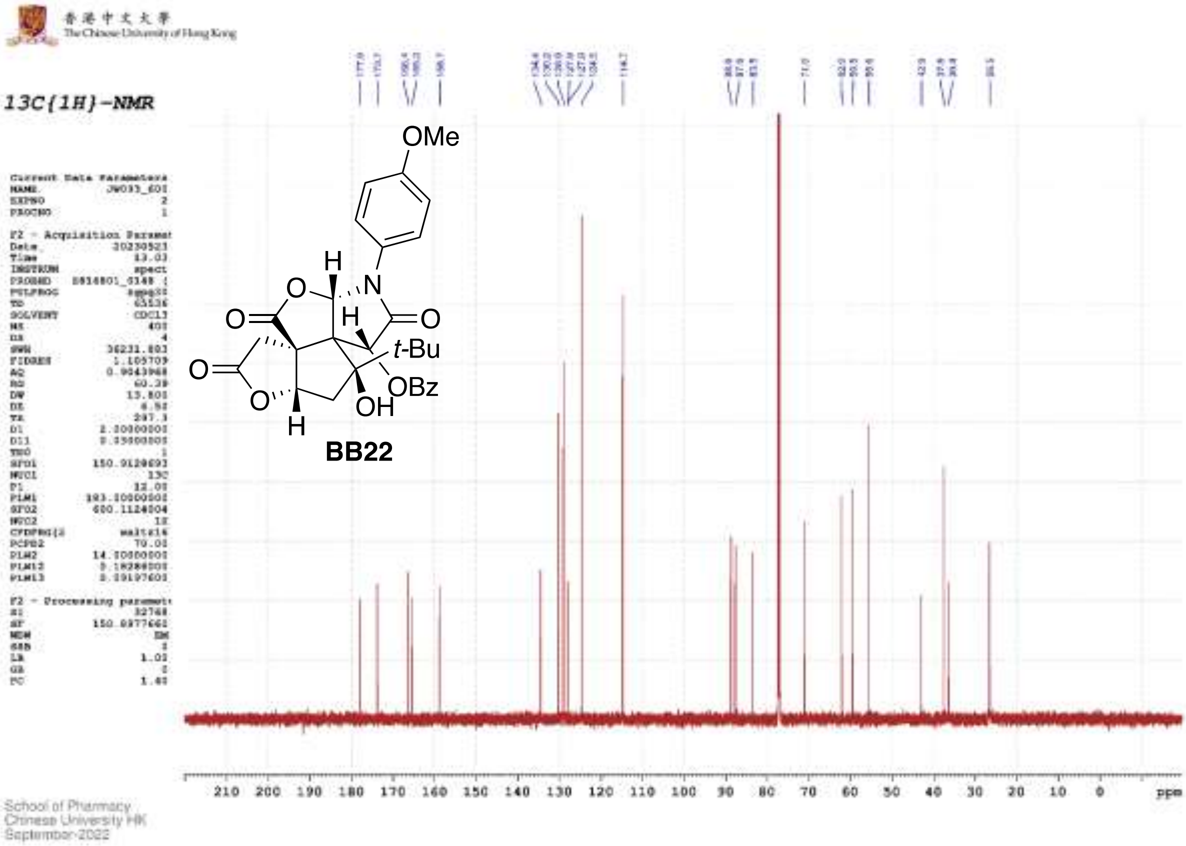

^{1}H NMR (500 MHz, Methanol-d_4) spectrum of BB23

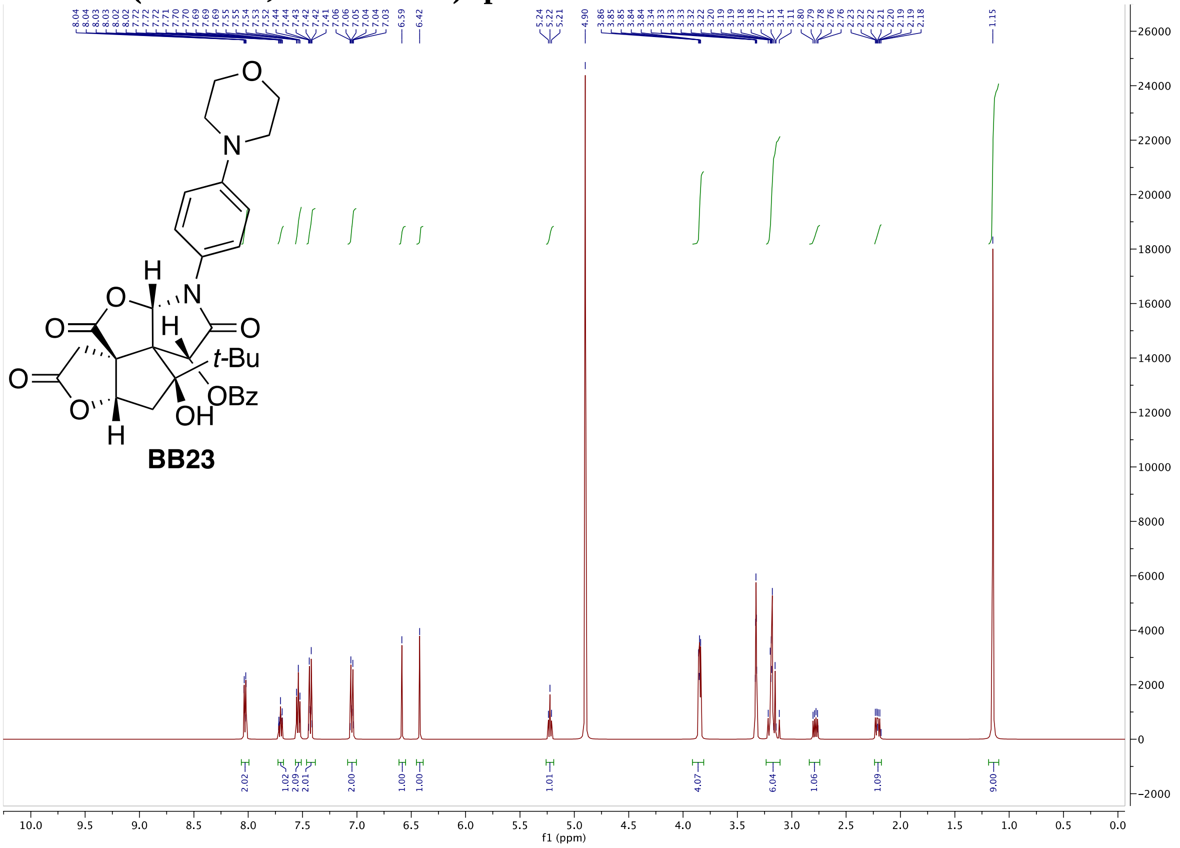

^{13}C NMR (126 MHz, Methanol-d_4) spectrum of BB23

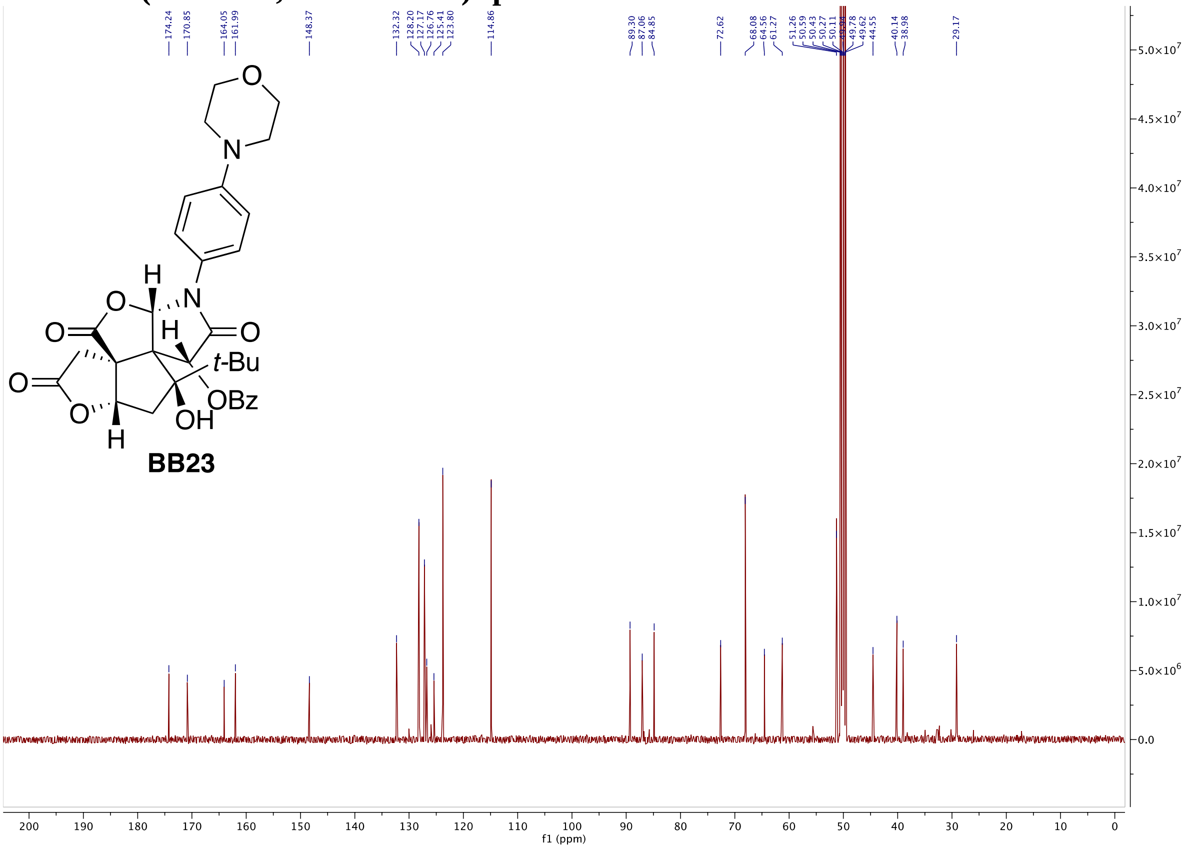

^{1}H NMR (500 MHz, Methanol-d_4) spectrum of BB24

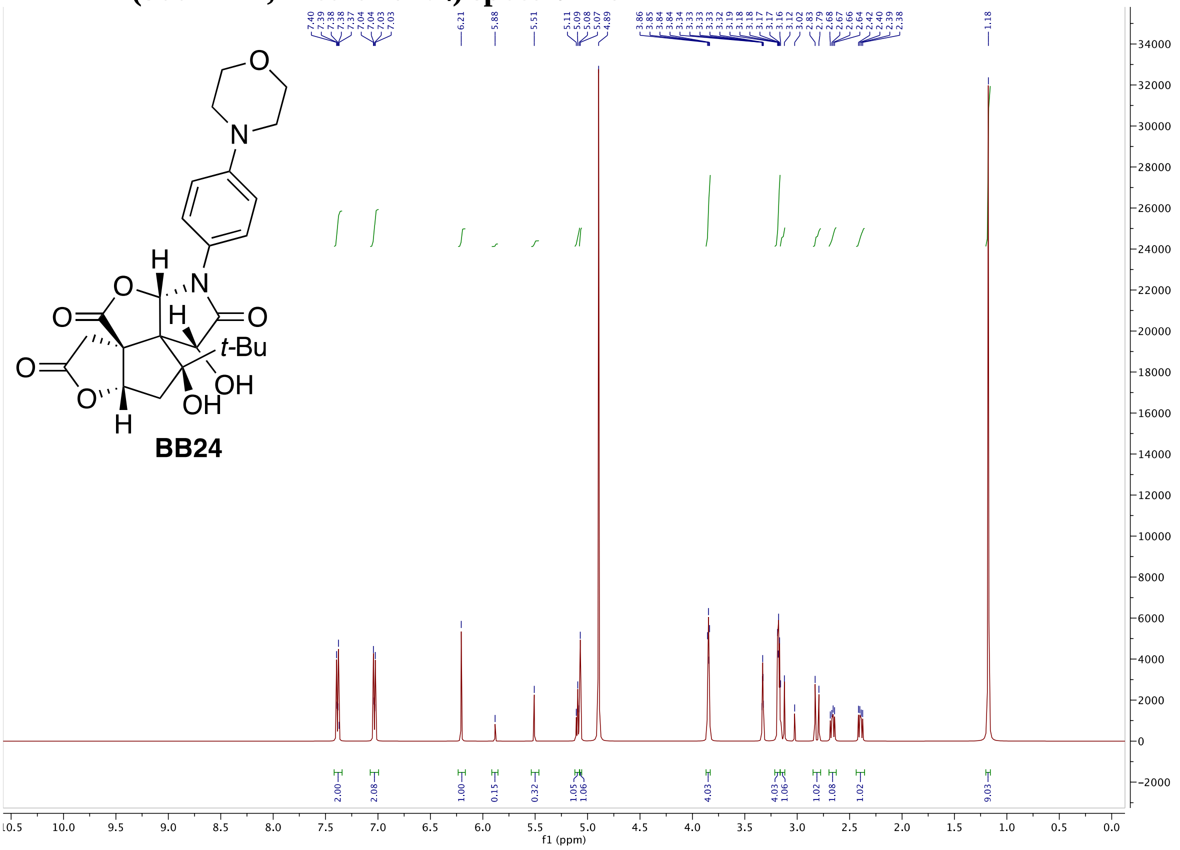

^{13}C NMR (126 MHz, Methanol-d_4) spectrum of BB24

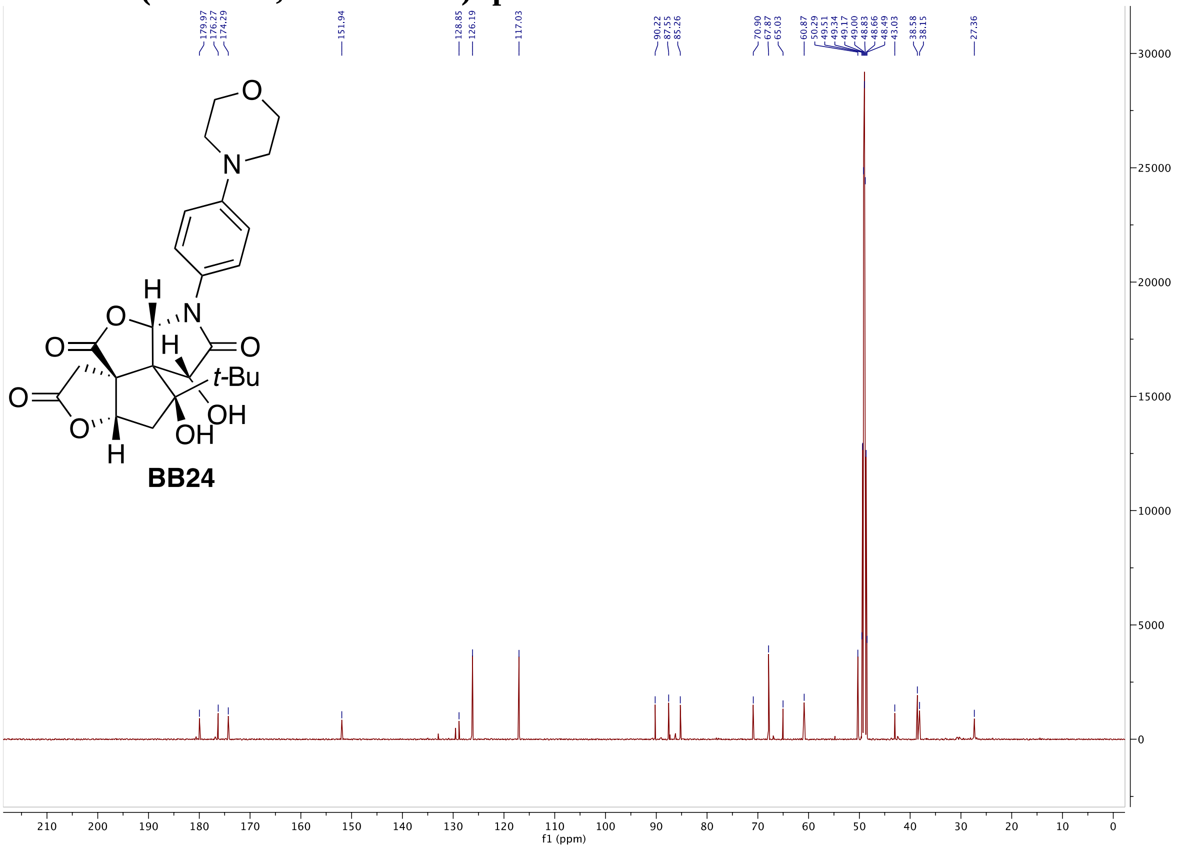

^{1}H NMR (500 MHz, DMSO-d_6) spectrum of BB25

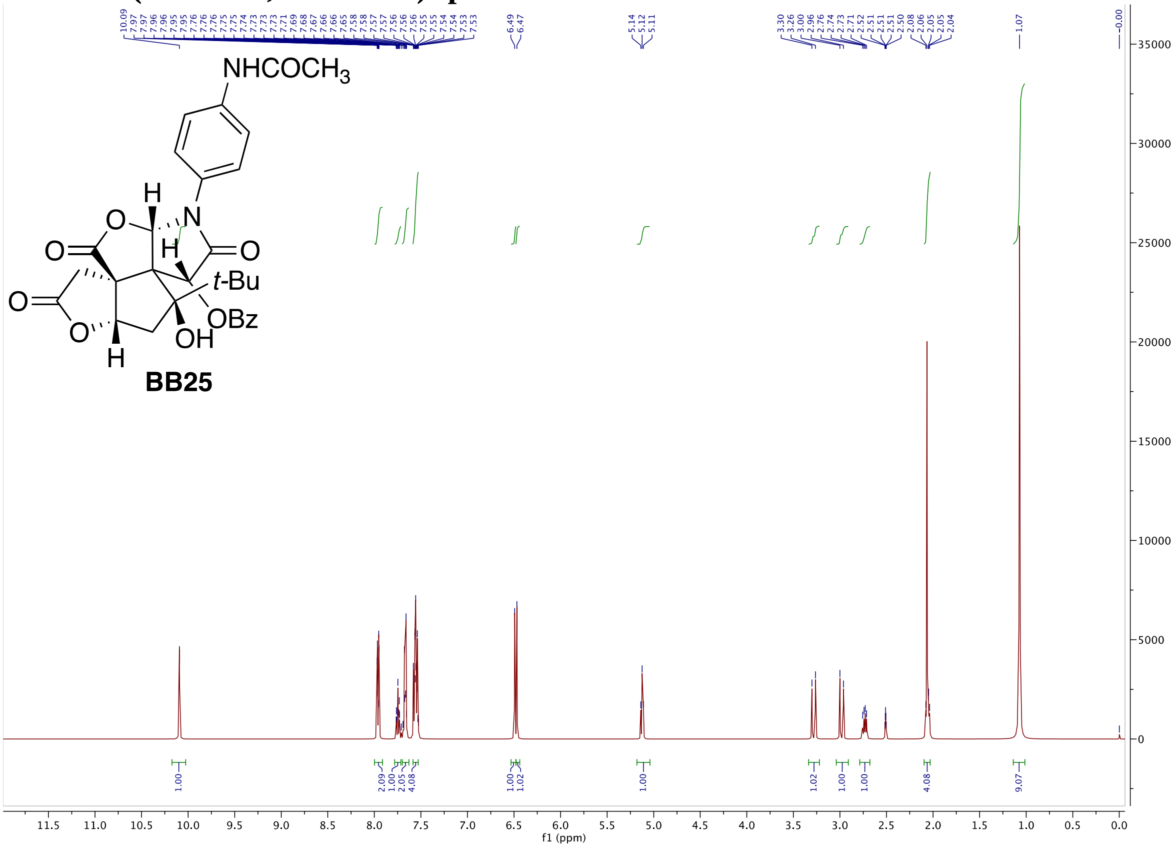

^{13}C NMR (126 MHz, DMSO-d_6) spectrum of BB25

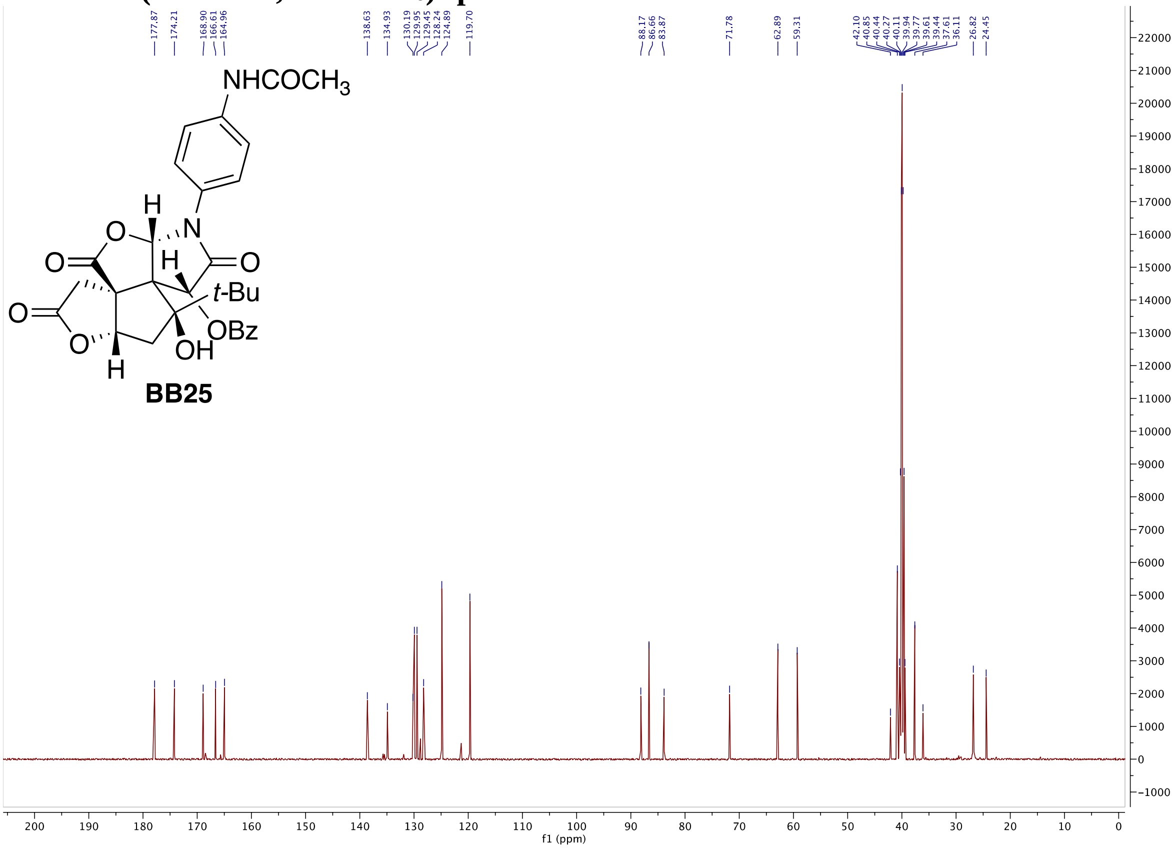

^{1}H NMR (400 MHz, $CDCl_3$) spectrum of BB26

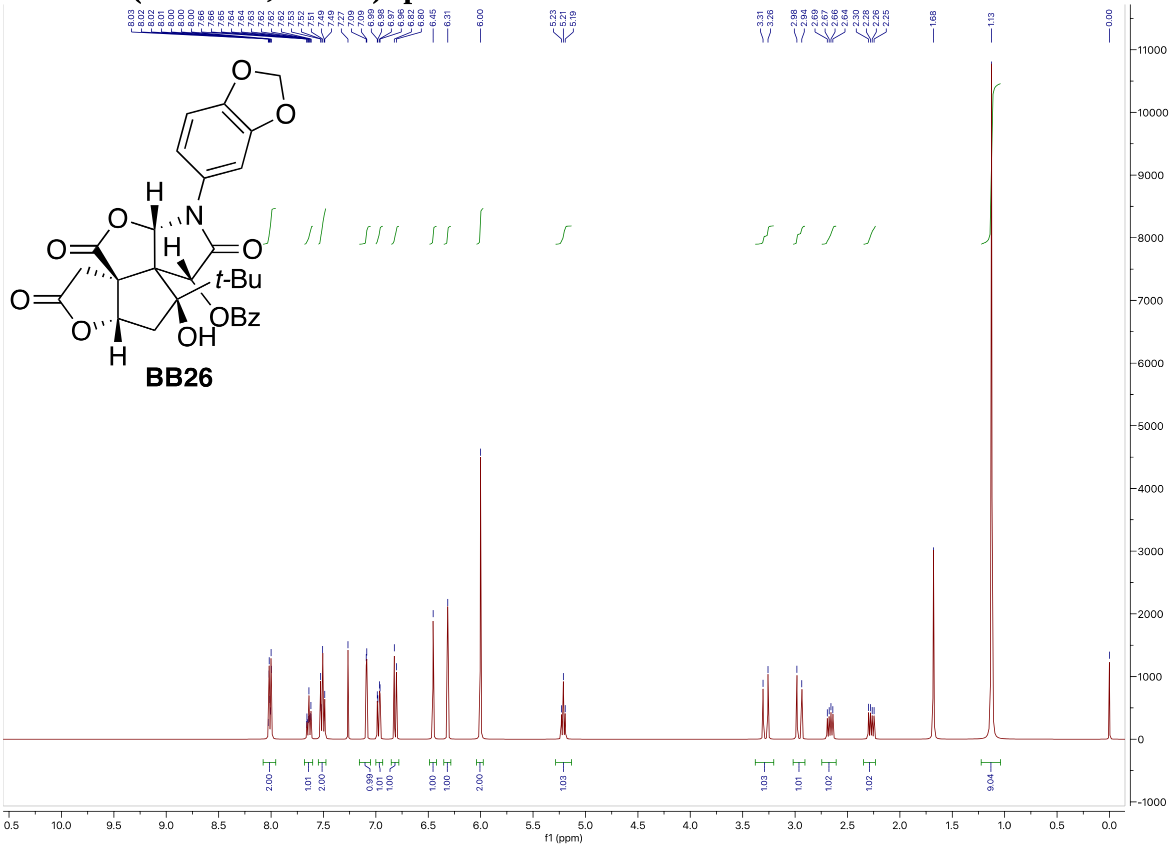

^{13}C NMR (101 MHz, $CDCl_3$) spectrum of BB26

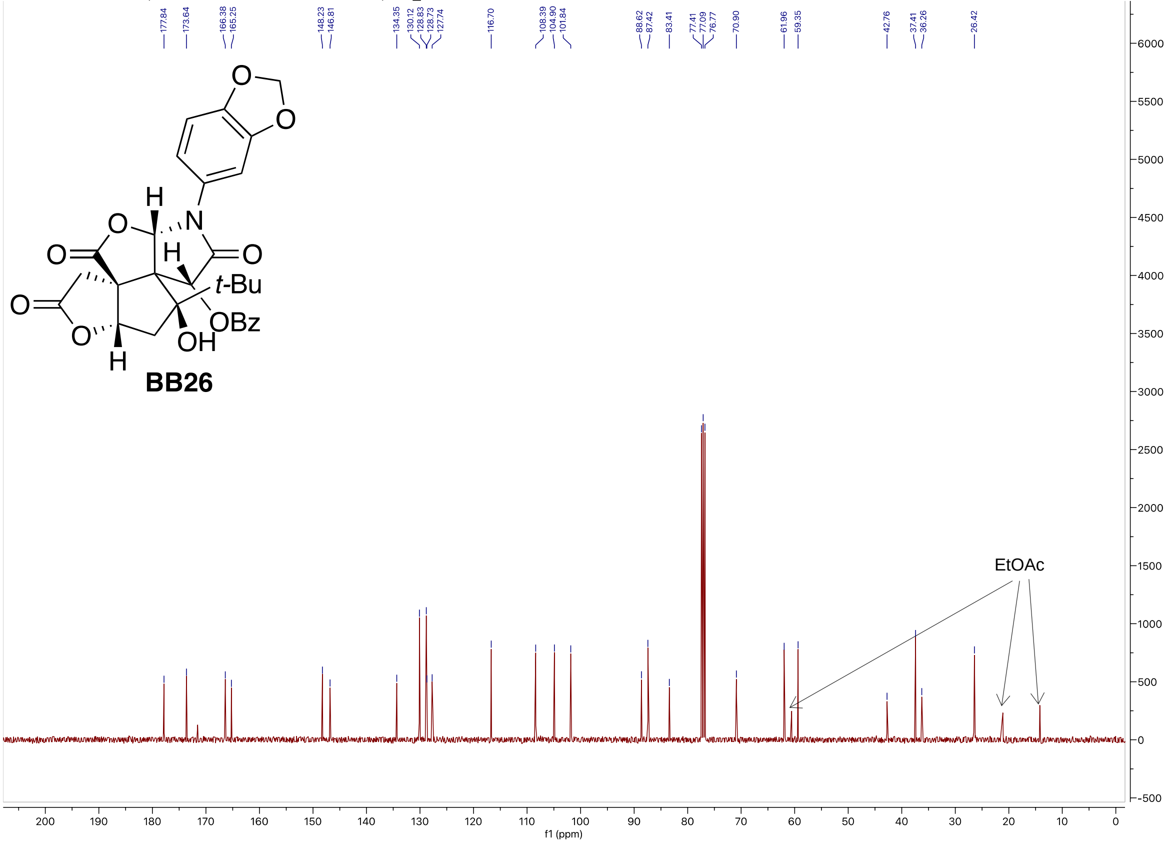

^{1}H NMR (400 MHz, DMSO-d_6) spectrum of BB27

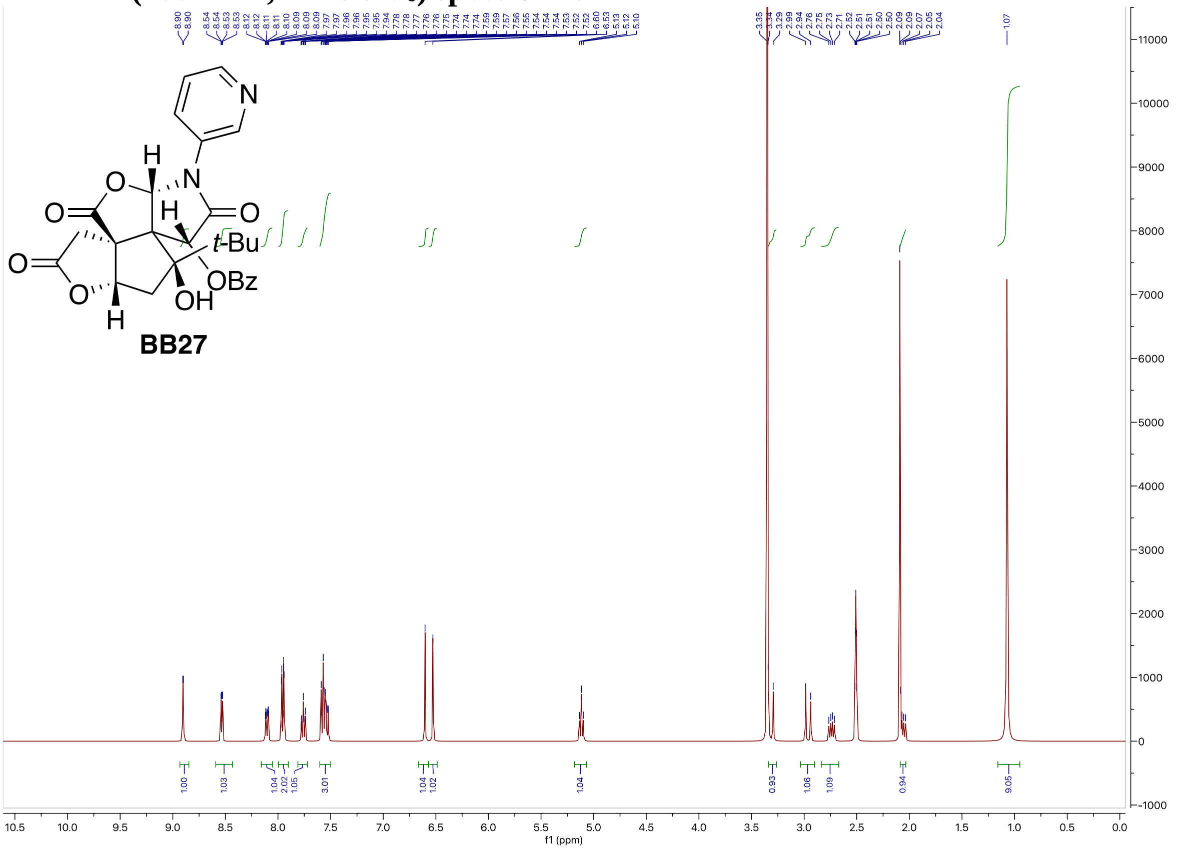

^{13}C NMR (101 MHz, DMSO-d_6) spectrum of BB27

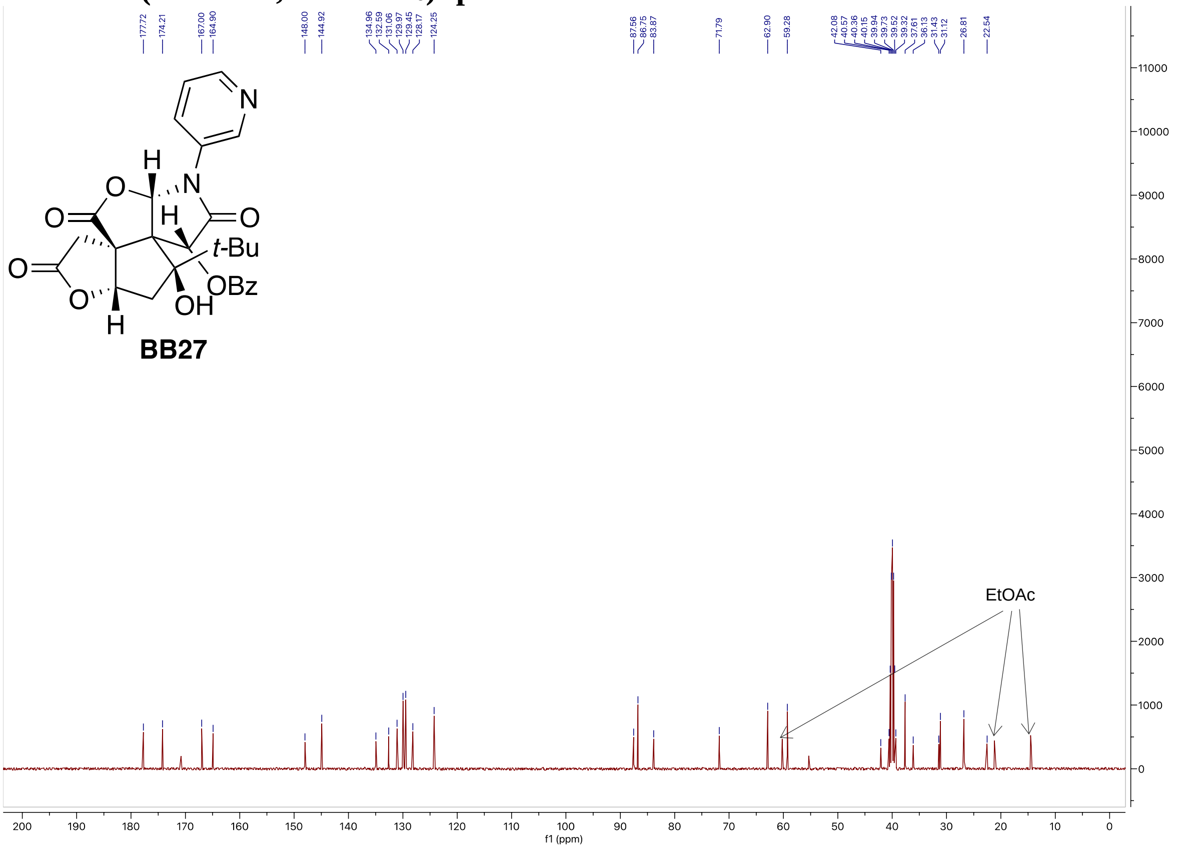

^{1}H NMR (600 MHz, CDCl$_3$) spectrum of BB28

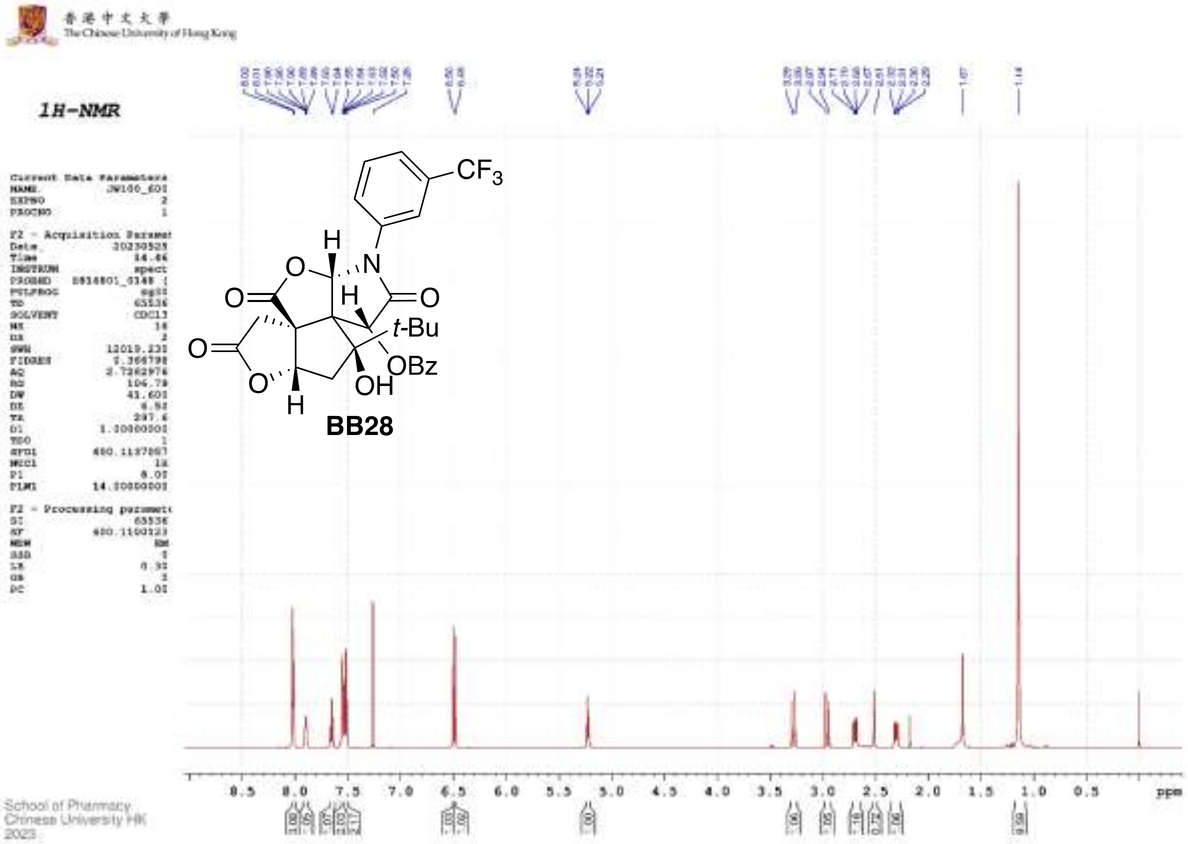

^{13}C NMR (150 MHz, CDCl$_3$) spectrum of BB28

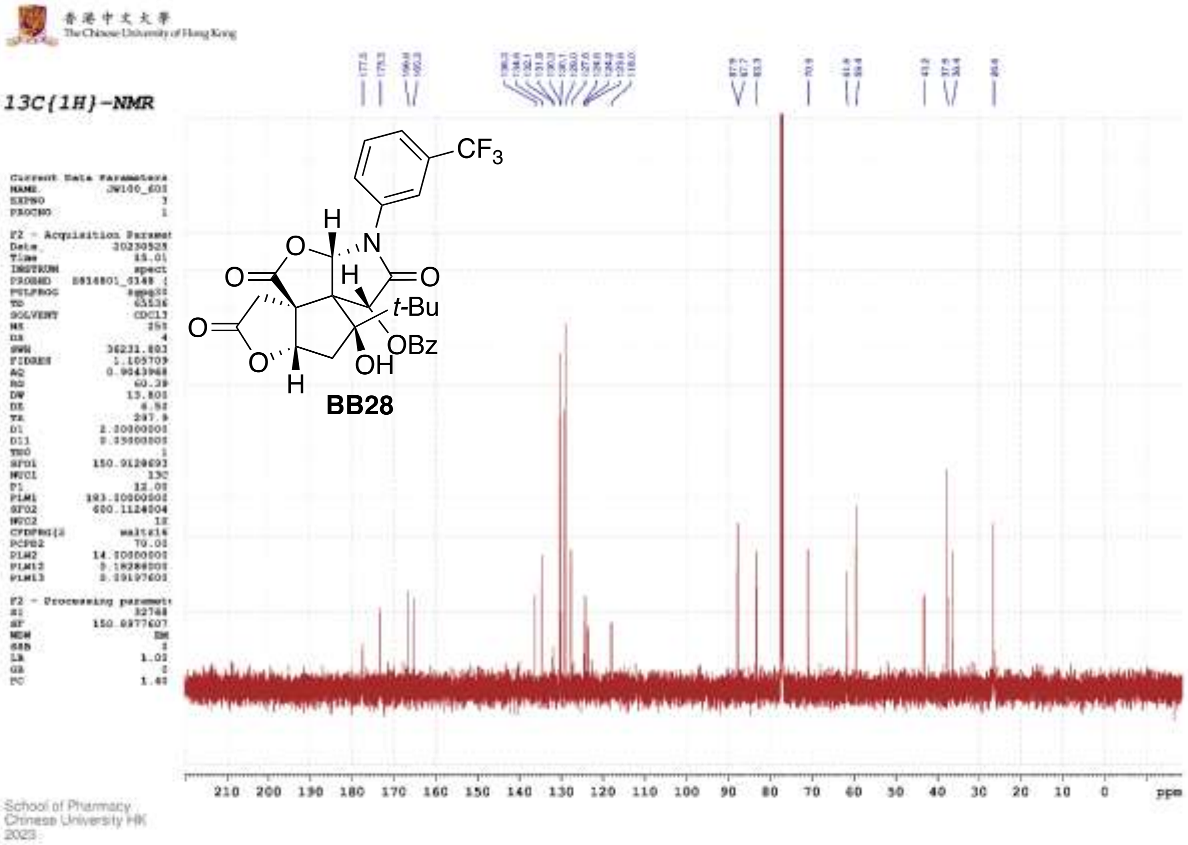

^{1}H NMR (600 MHz, $CDCl_3$) spectrum of BB29

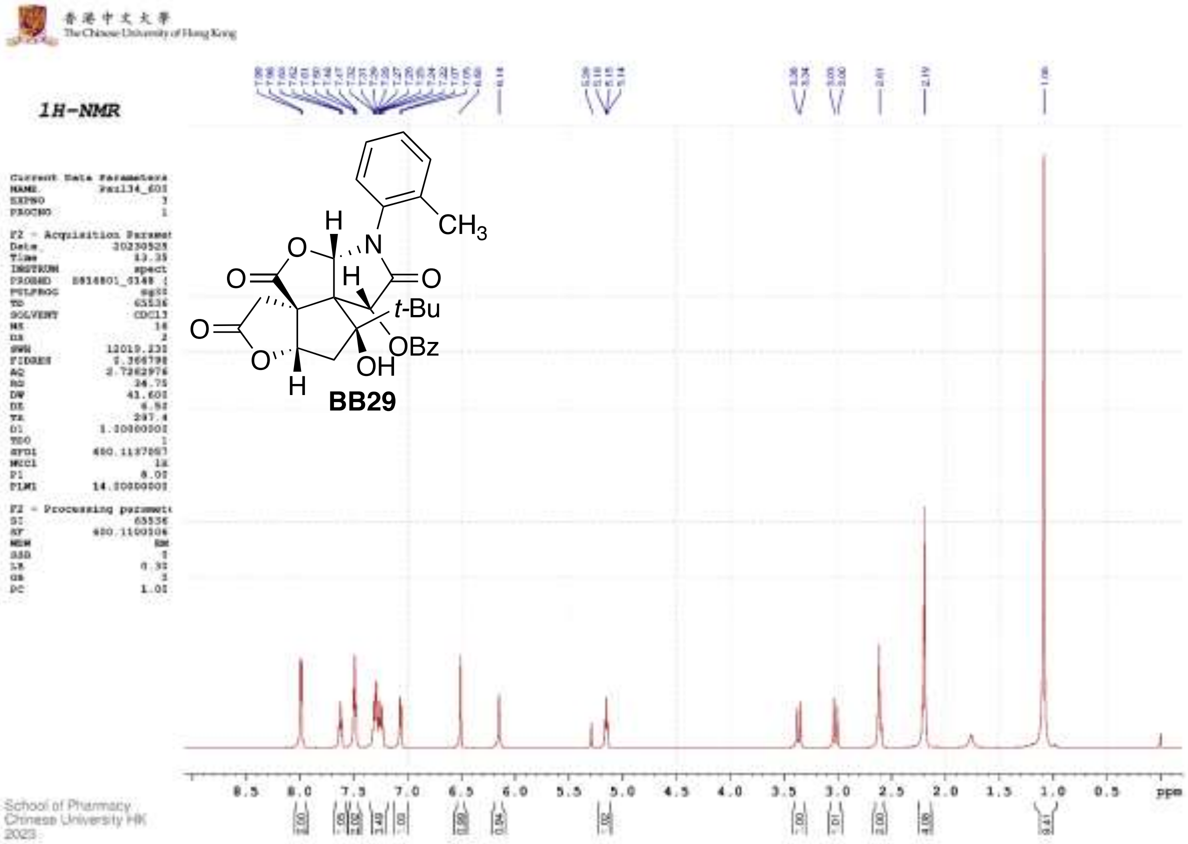

^{13}C NMR (150 MHz, $CDCl_3$) spectrum of BB29

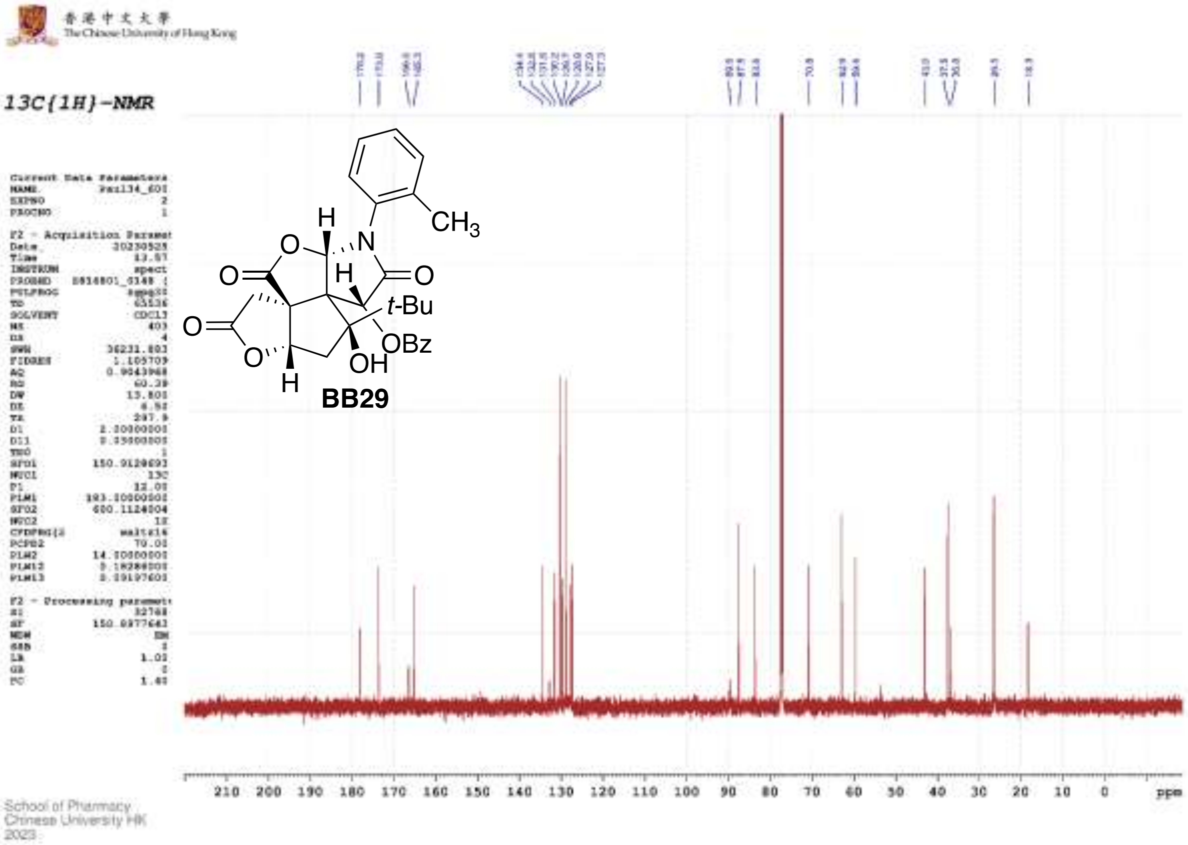

^{1}H NMR (600 MHz, $CDCl_3$) spectrum of BB30

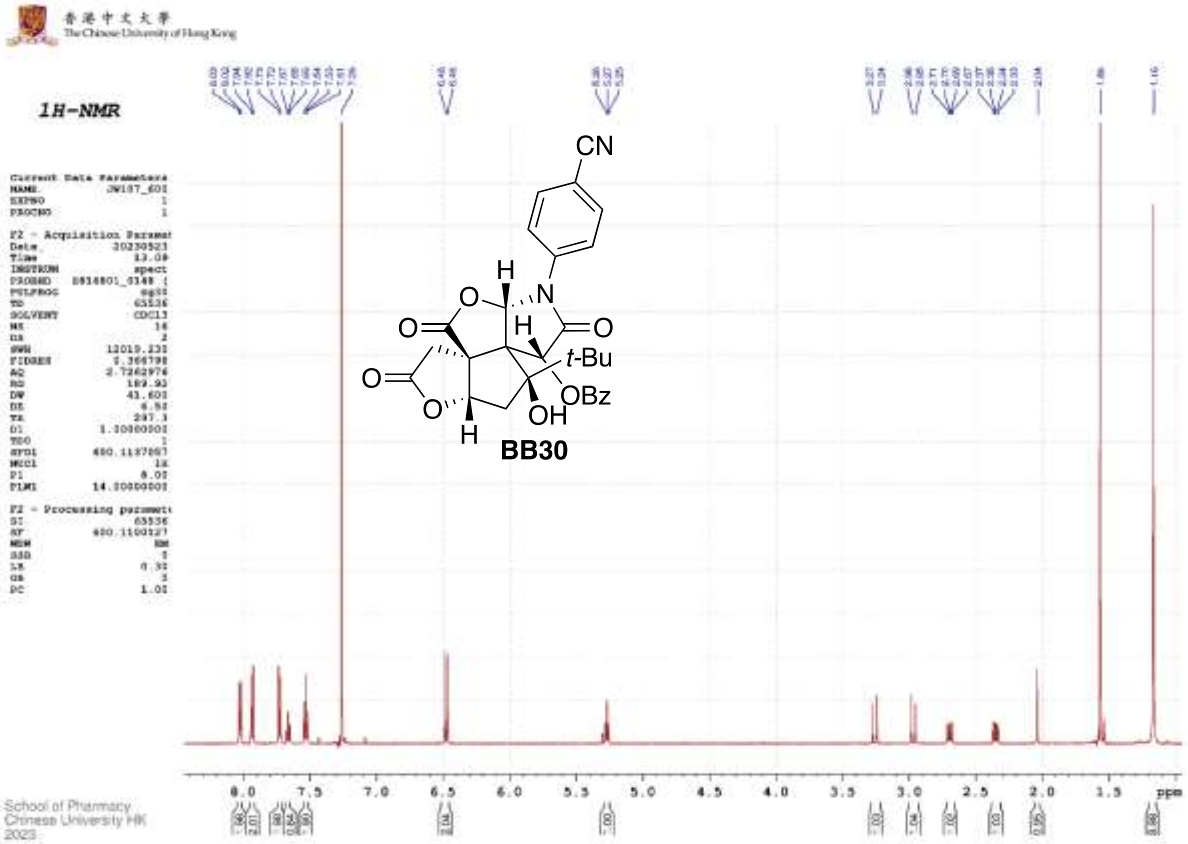

HSQC (150 MHz, Methanol-d_4) spectrum of BB30

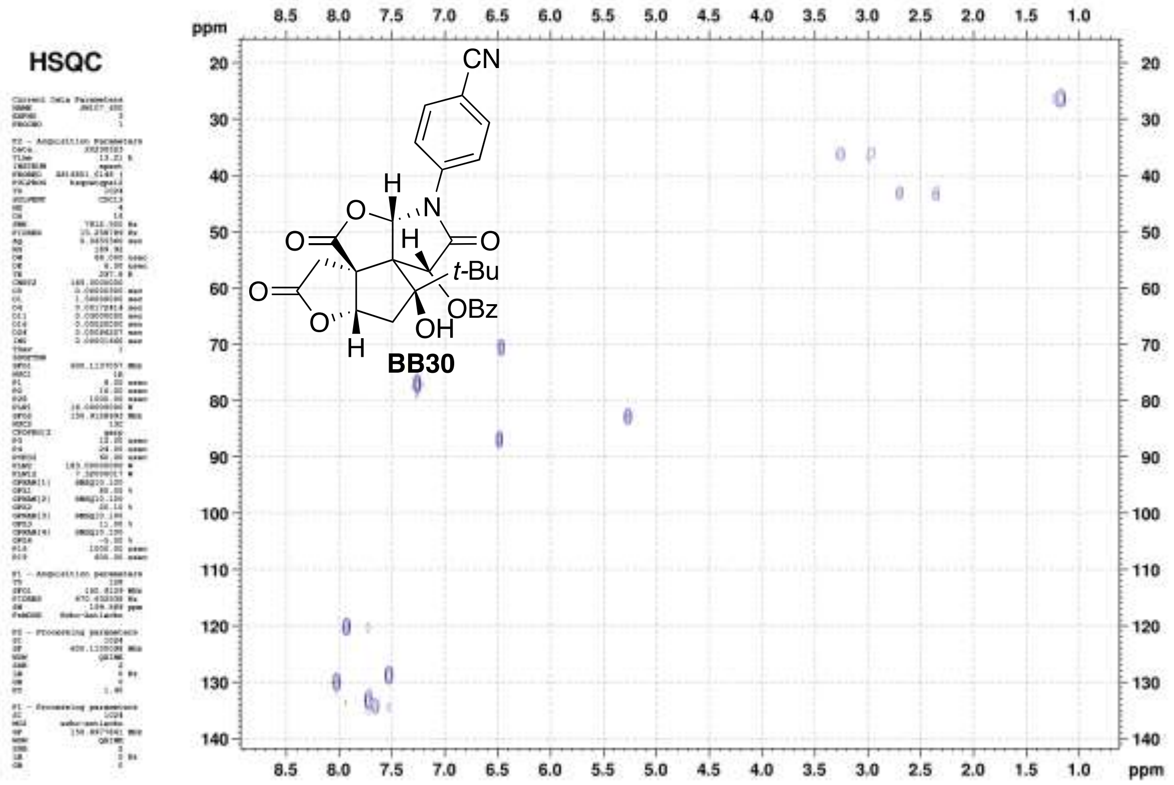

HMBC (150 MHz, Methanol-d_4) spectrum of BB30

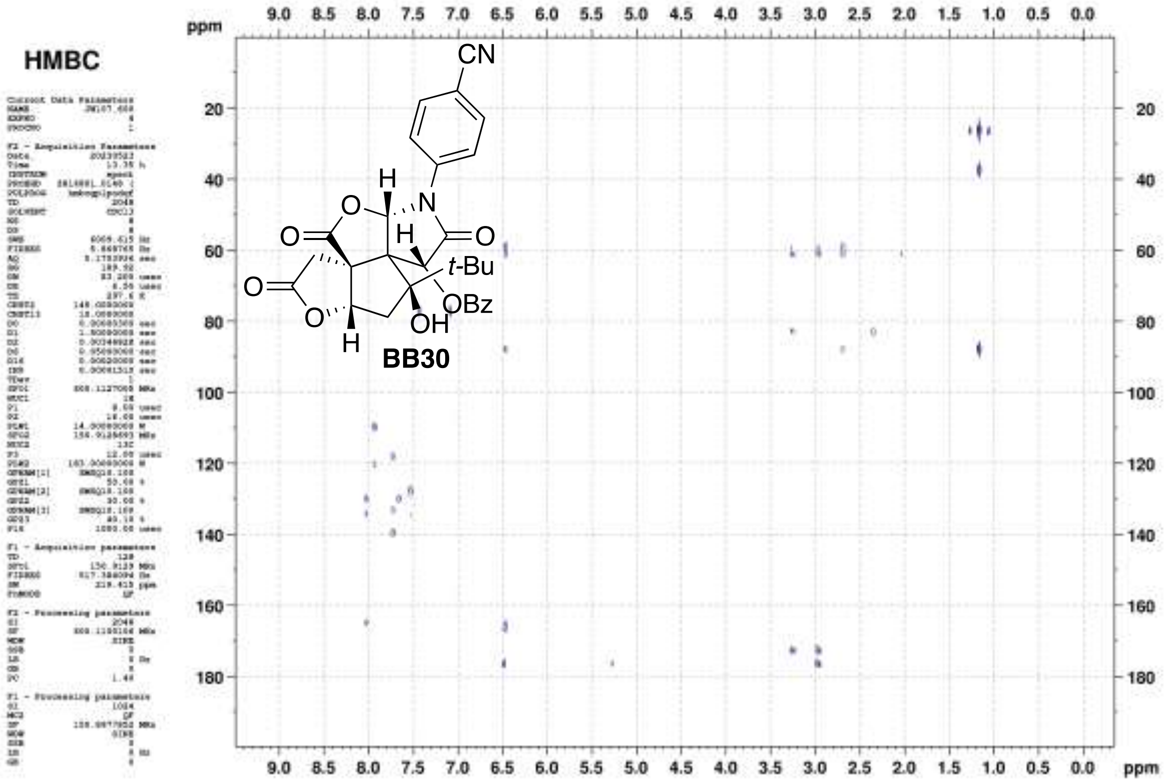

^{1}H NMR (600 MHz, $CDCl_3$) spectrum of BB31

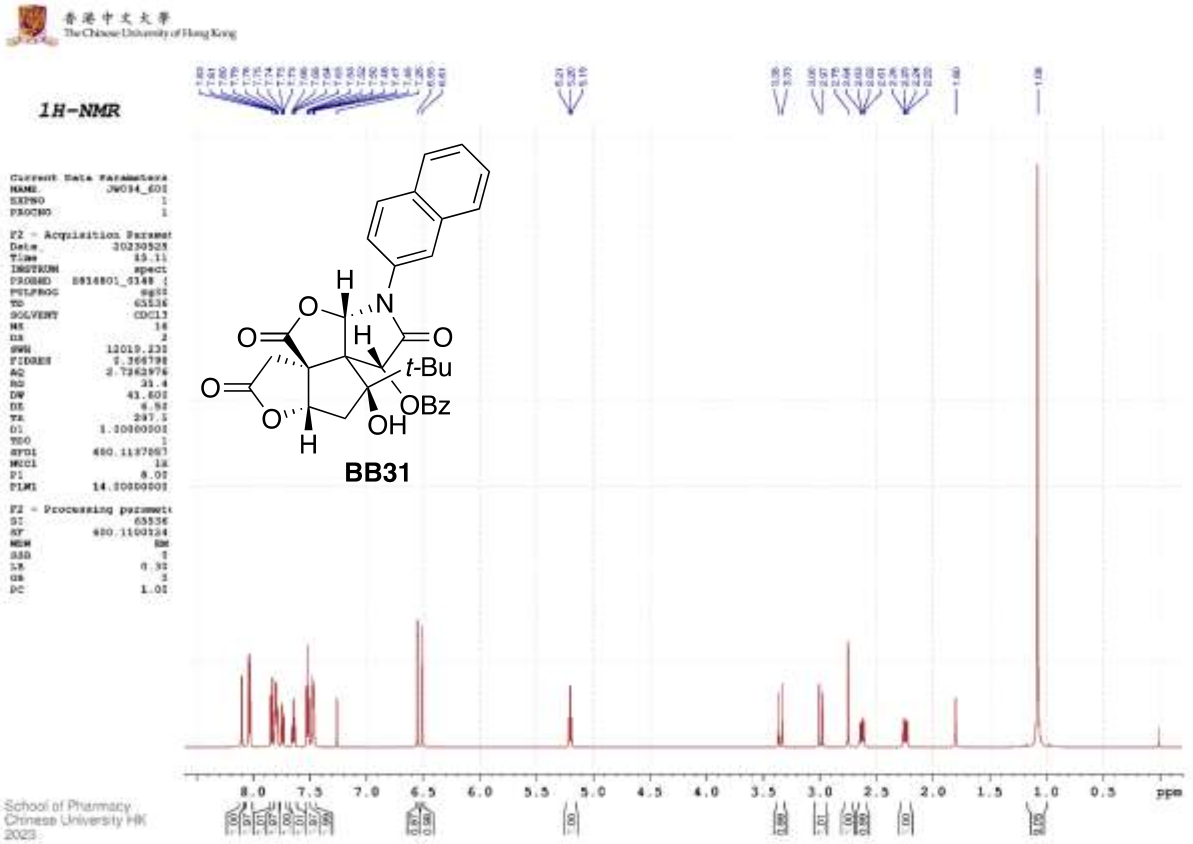

^{13}C NMR (150 MHz, $CDCl_3$) spectrum of BB31

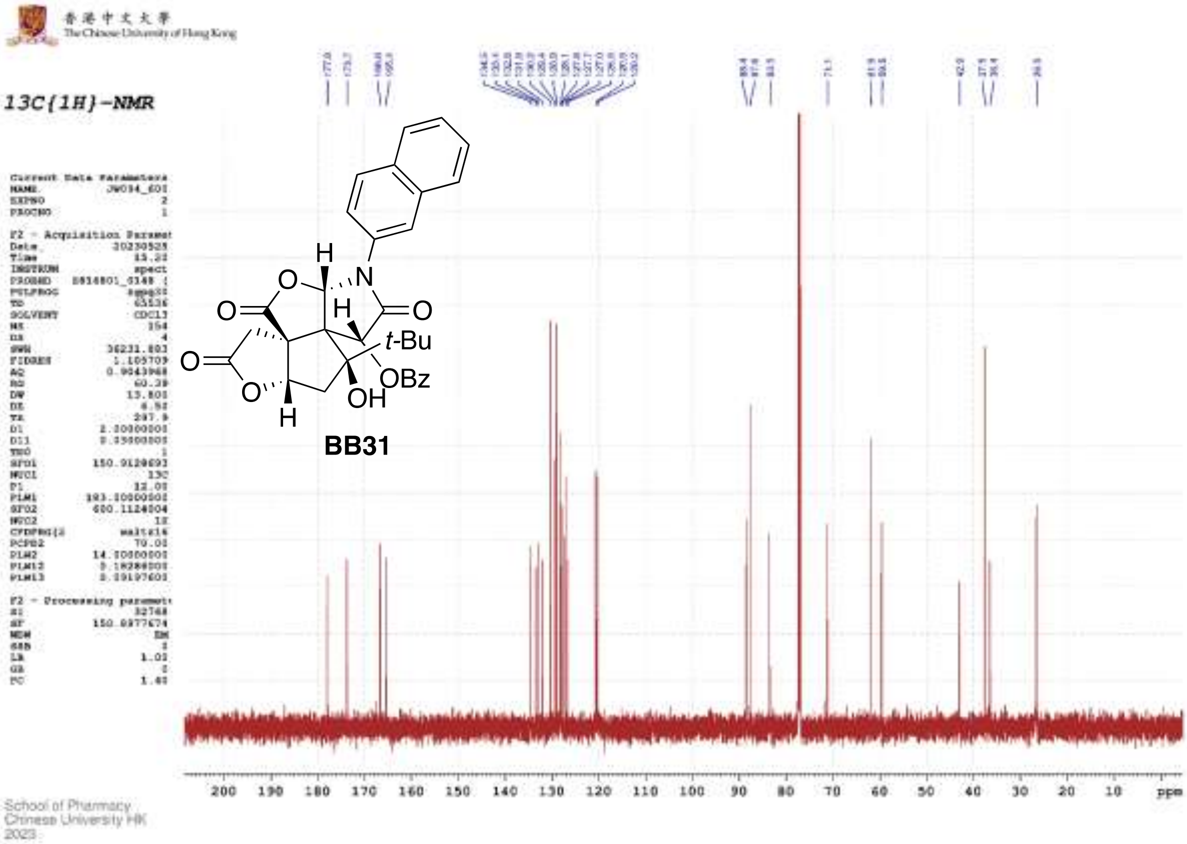

^{1}H NMR (600 MHz, MeOH-d_4) spectrum of BB32

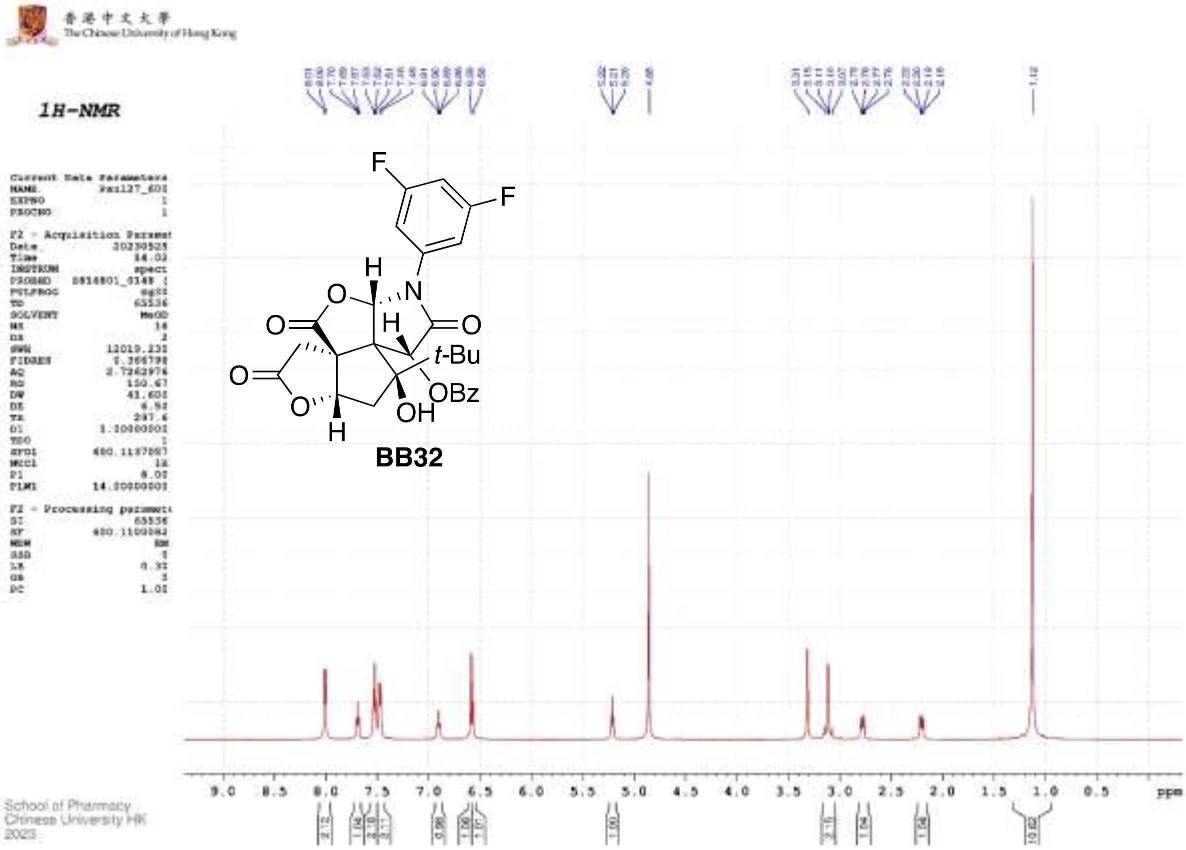

^{13}C NMR (150 MHz, MeOH-d_4) spectrum of BB32

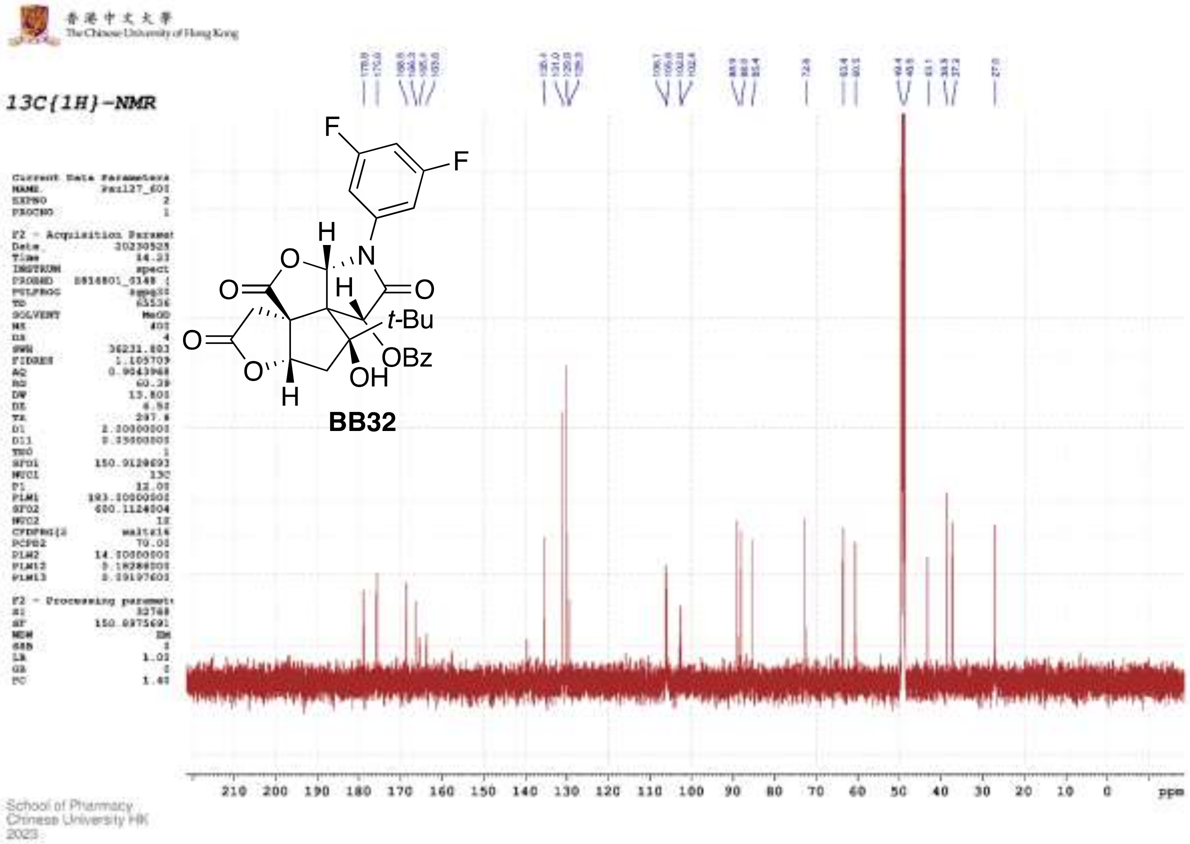

^{1}H NMR (500 MHz, CDCl$_3$) spectrum of 4

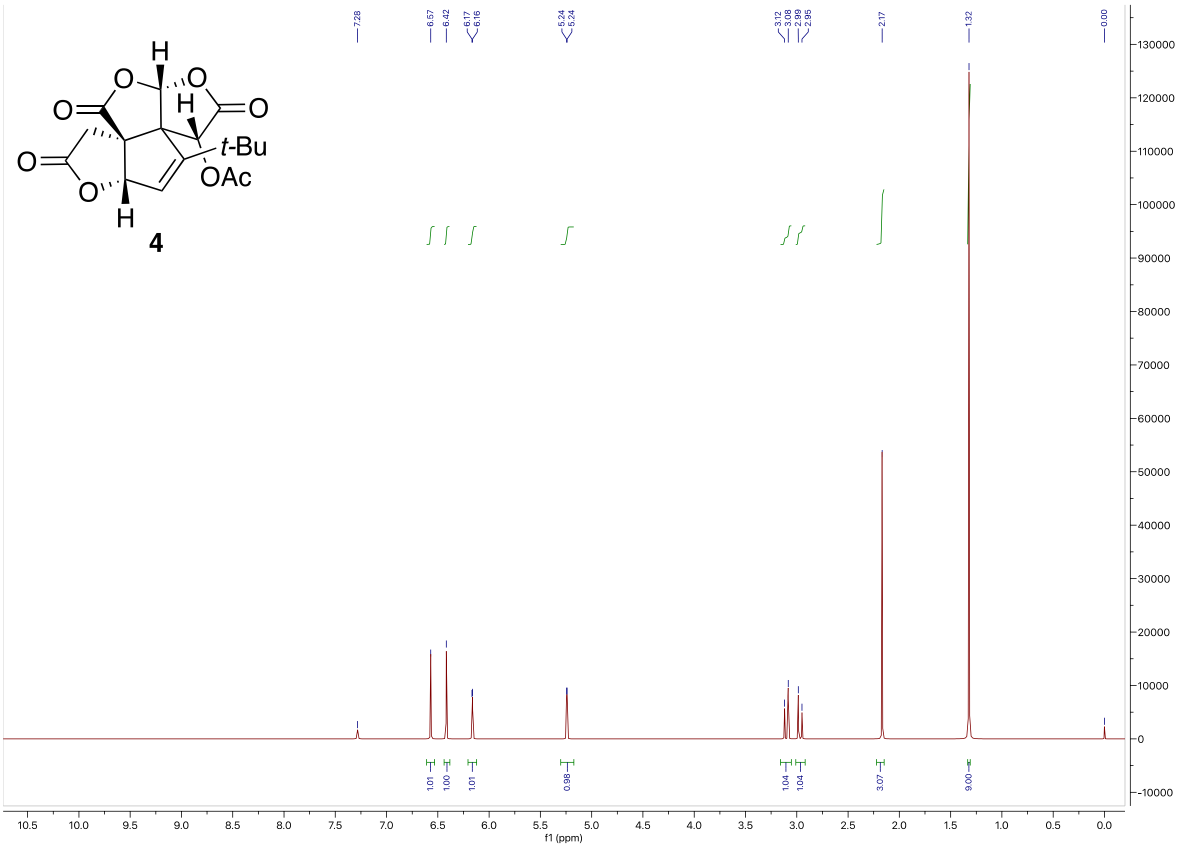

^{13}C NMR (126 MHz, $CDCl_3$) spectrum of 4

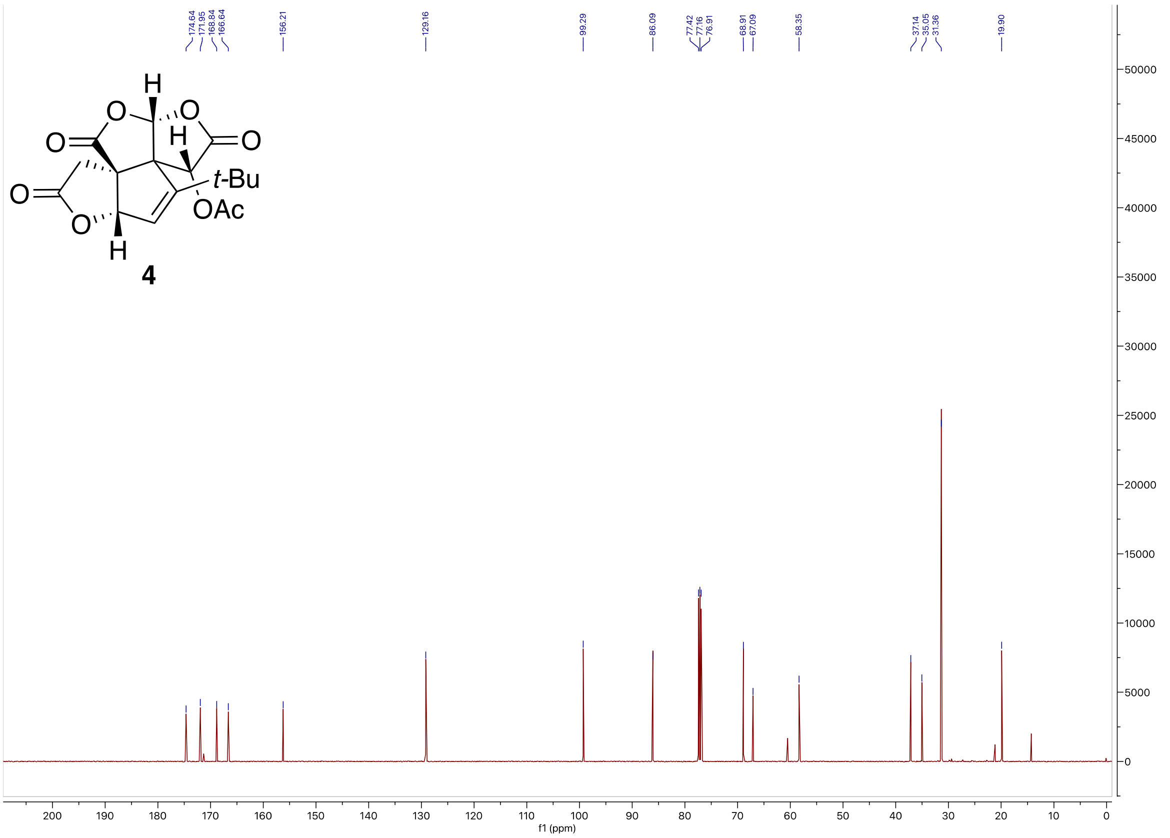

^{1}H NMR (500 MHz, $CDCl_3$) spectrum of 5

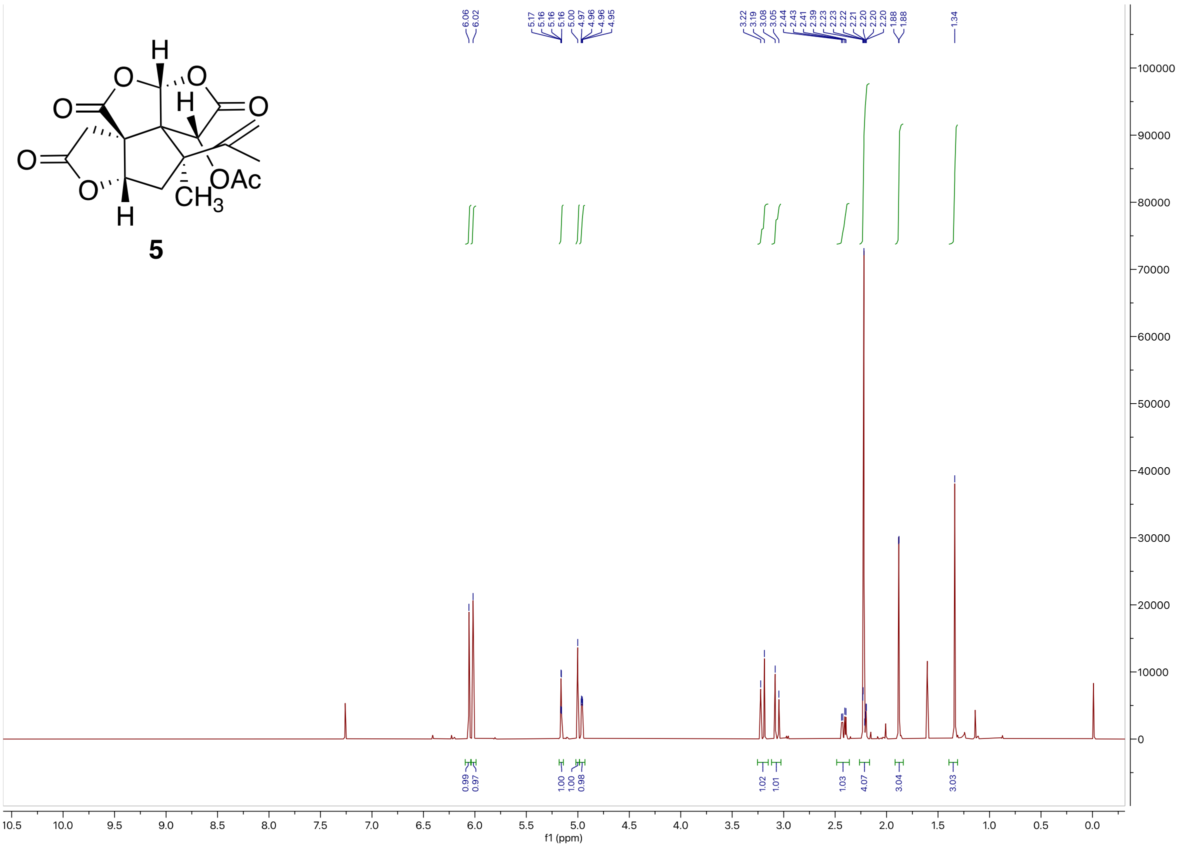

^{13}C NMR (126 MHz, $CDCl_3$) spectrum of 5

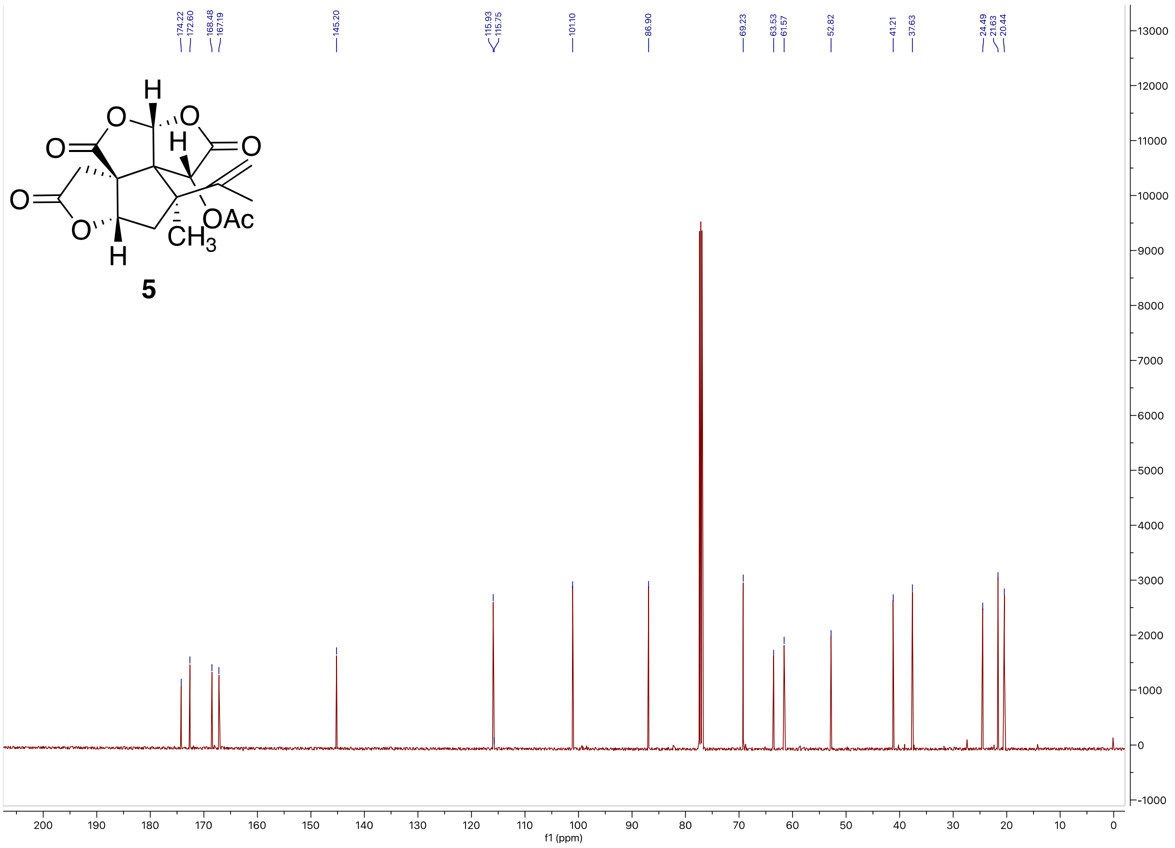

^{1}H NMR (500 MHz, $CDCl_3$) spectrum of BB33

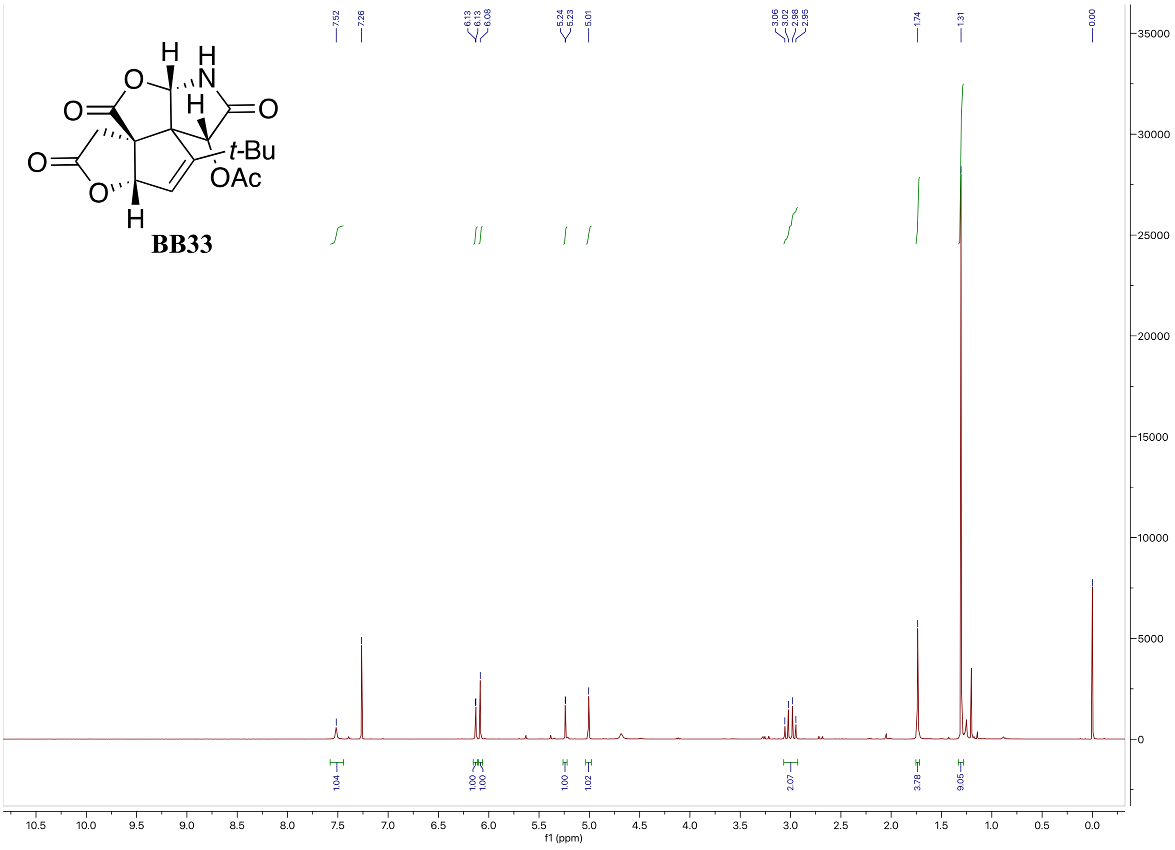

^{13}C NMR (126 MHz, $CDCl_3$) spectrum of BB33

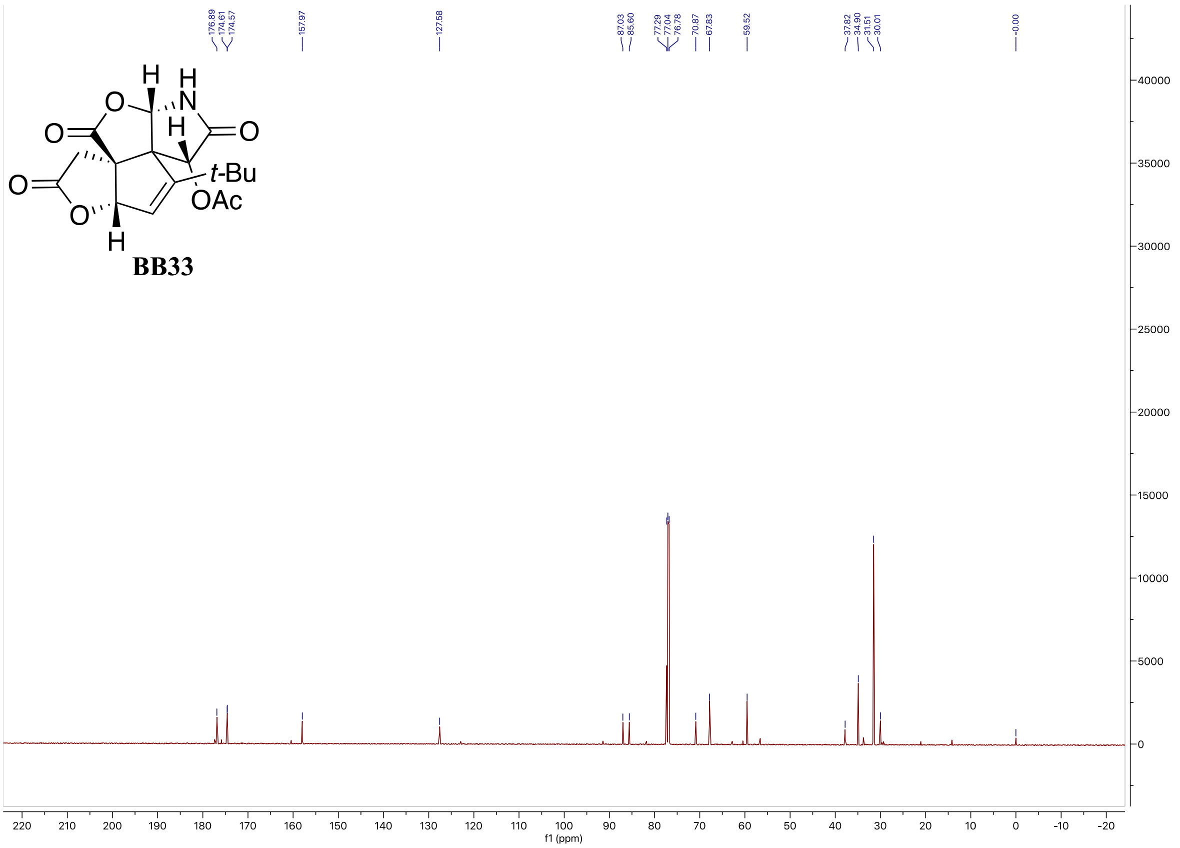

^{1}H NMR (400 MHz, MeOH-d_4) spectrum of BB34

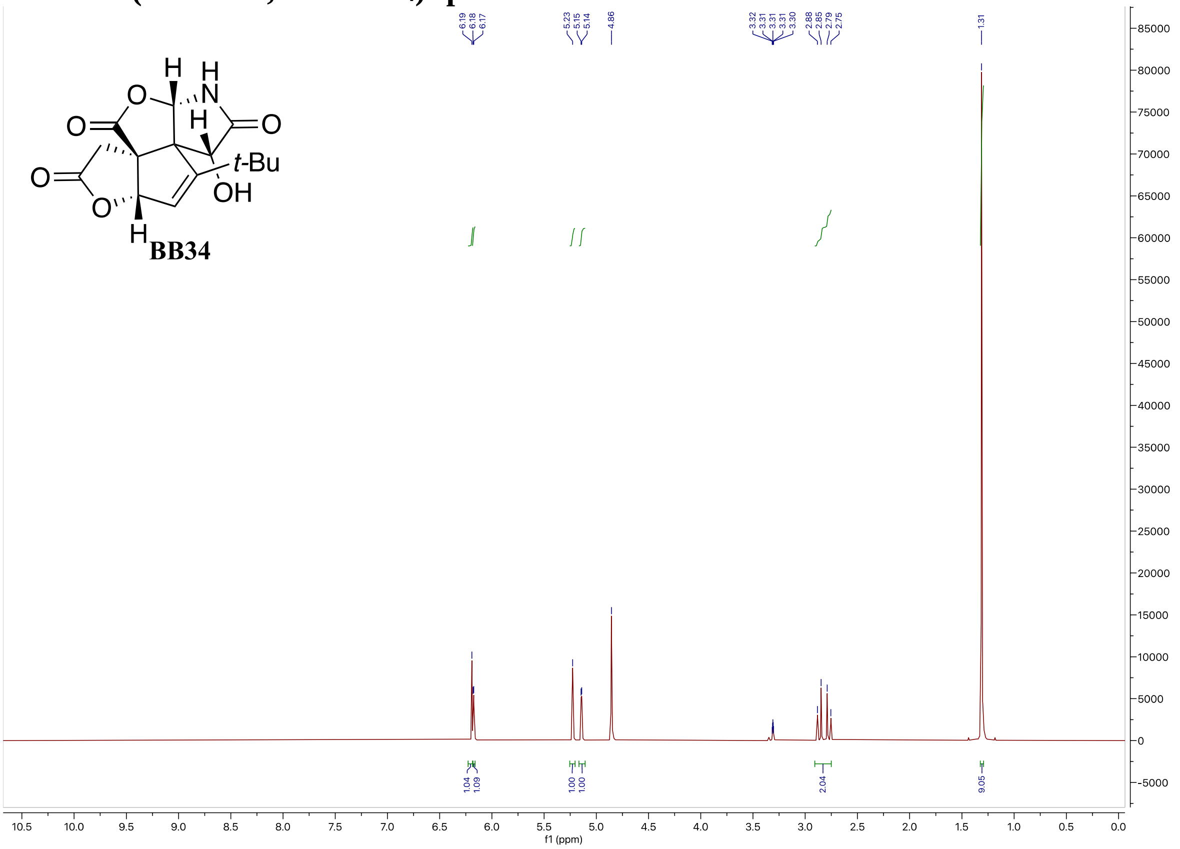

^{13}C NMR (126 MHz, MeOH-d_4) spectrum of BB34

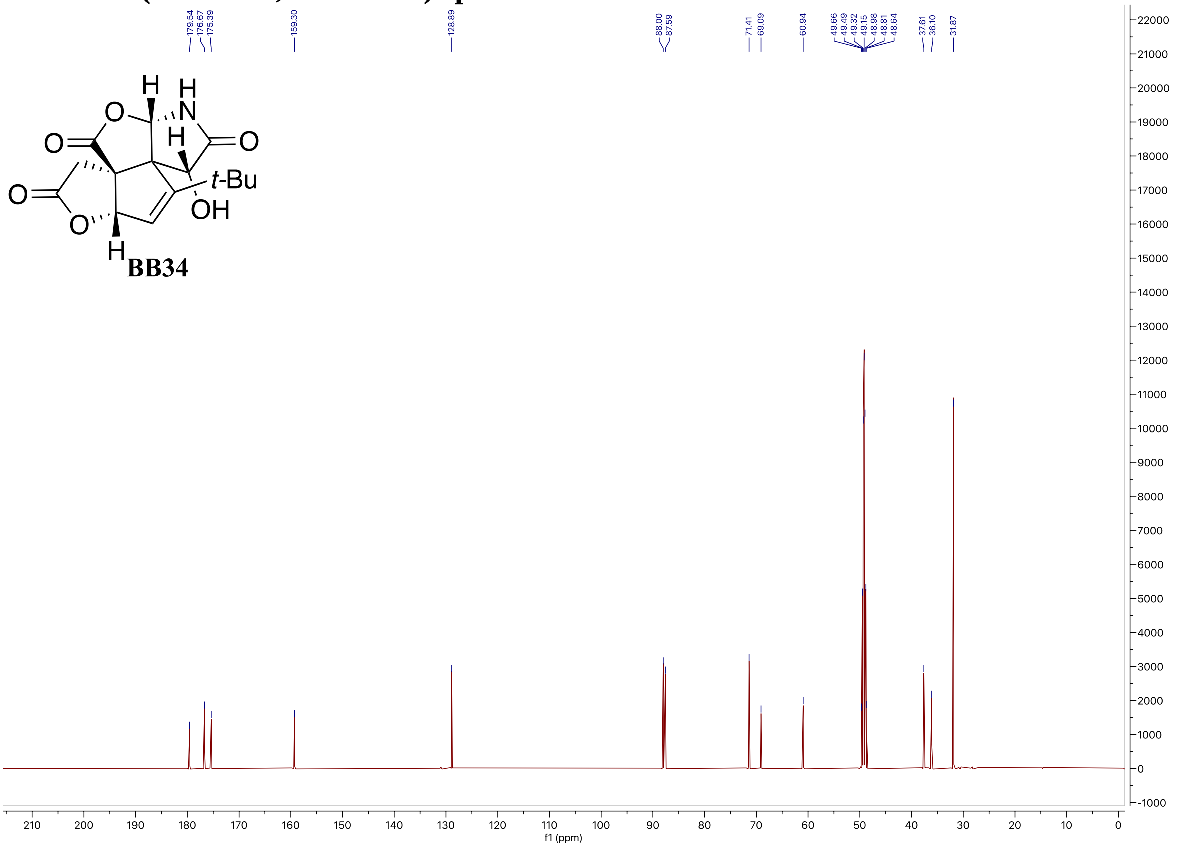

^{1}H NMR (500 MHz, Methanol-d_4) spectrum of BB35

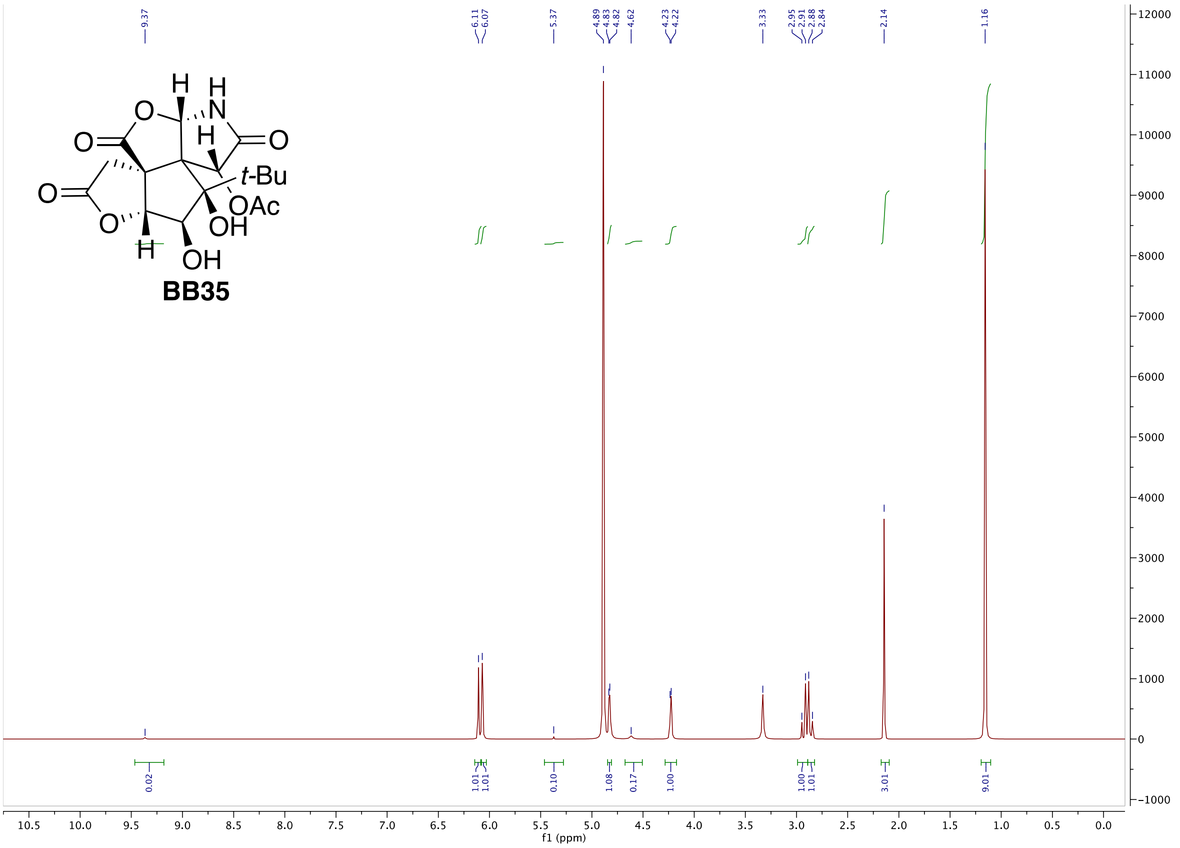

^{13}C NMR (126 MHz, Methanol-d_4) spectrum of BB35

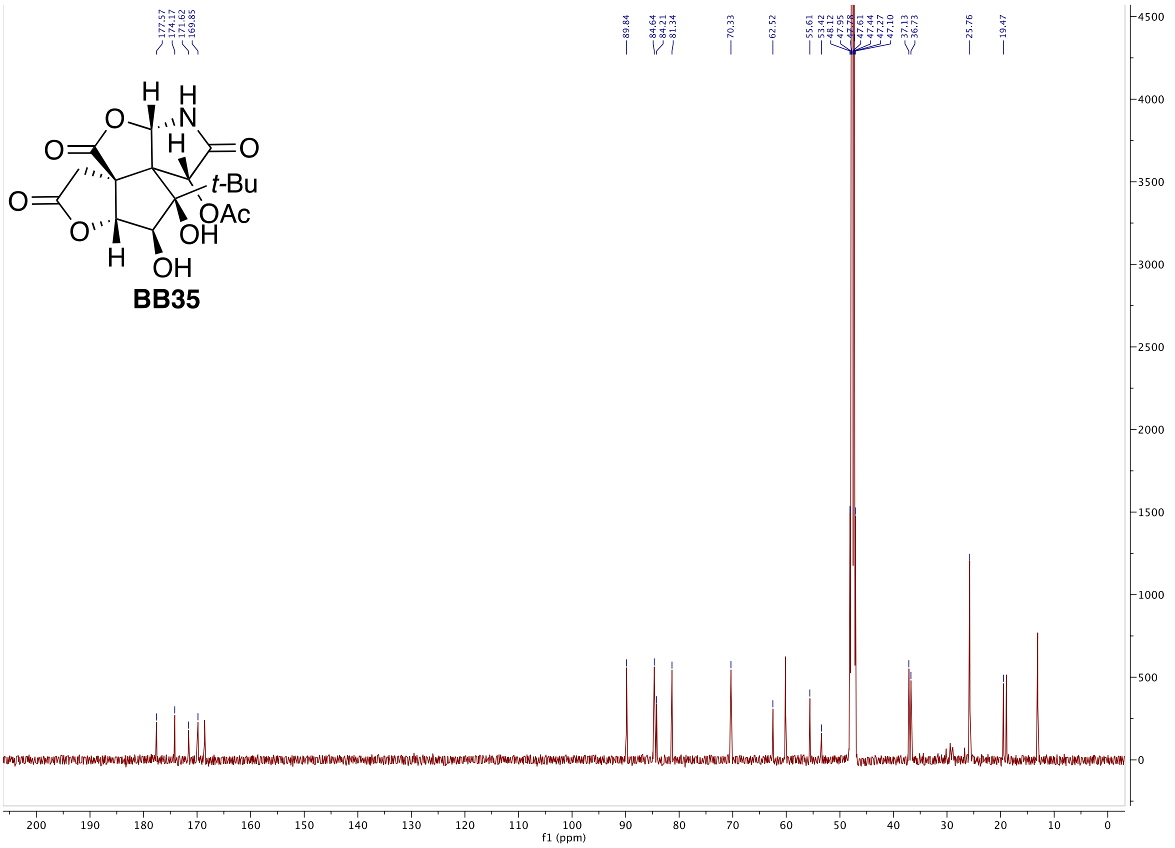

^{1}H NMR (400 MHz, Methanol-d_4) spectrum of BB36

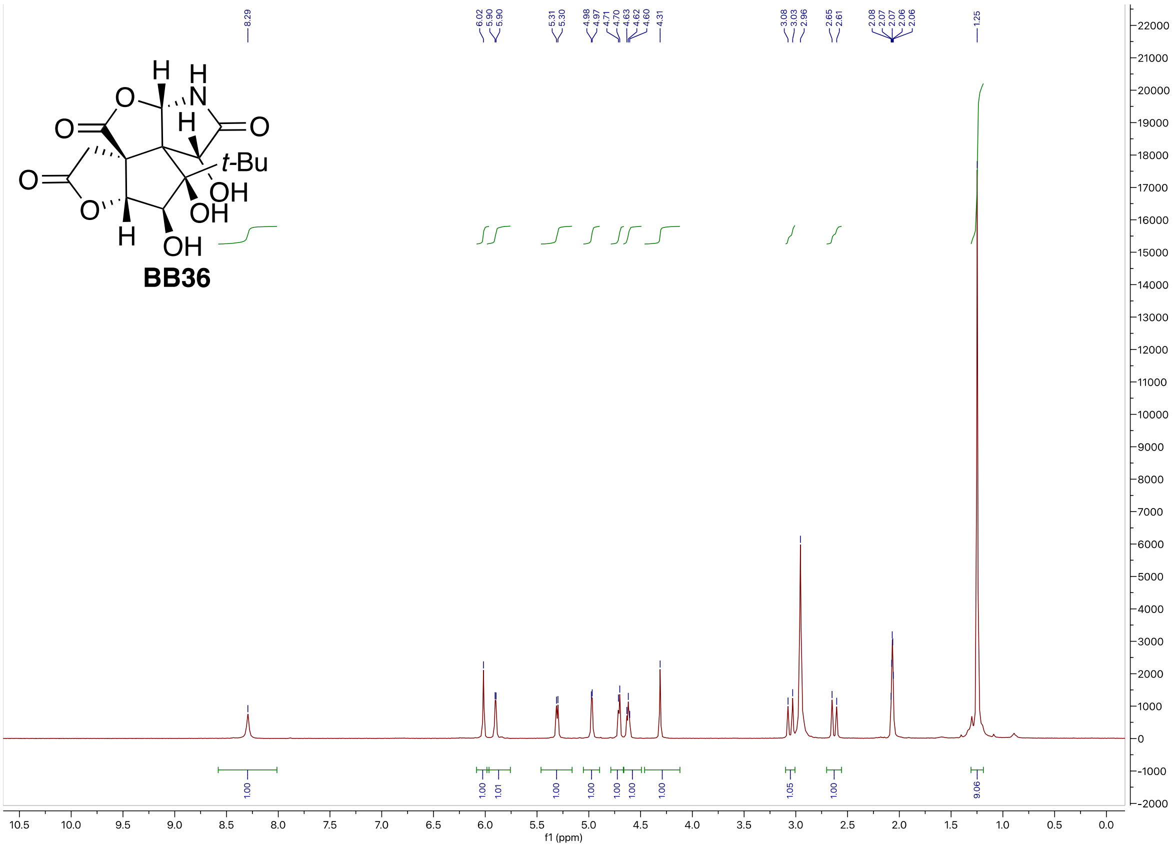

^{13}C NMR (101 MHz, Acetone-d_6) spectrum of BB36

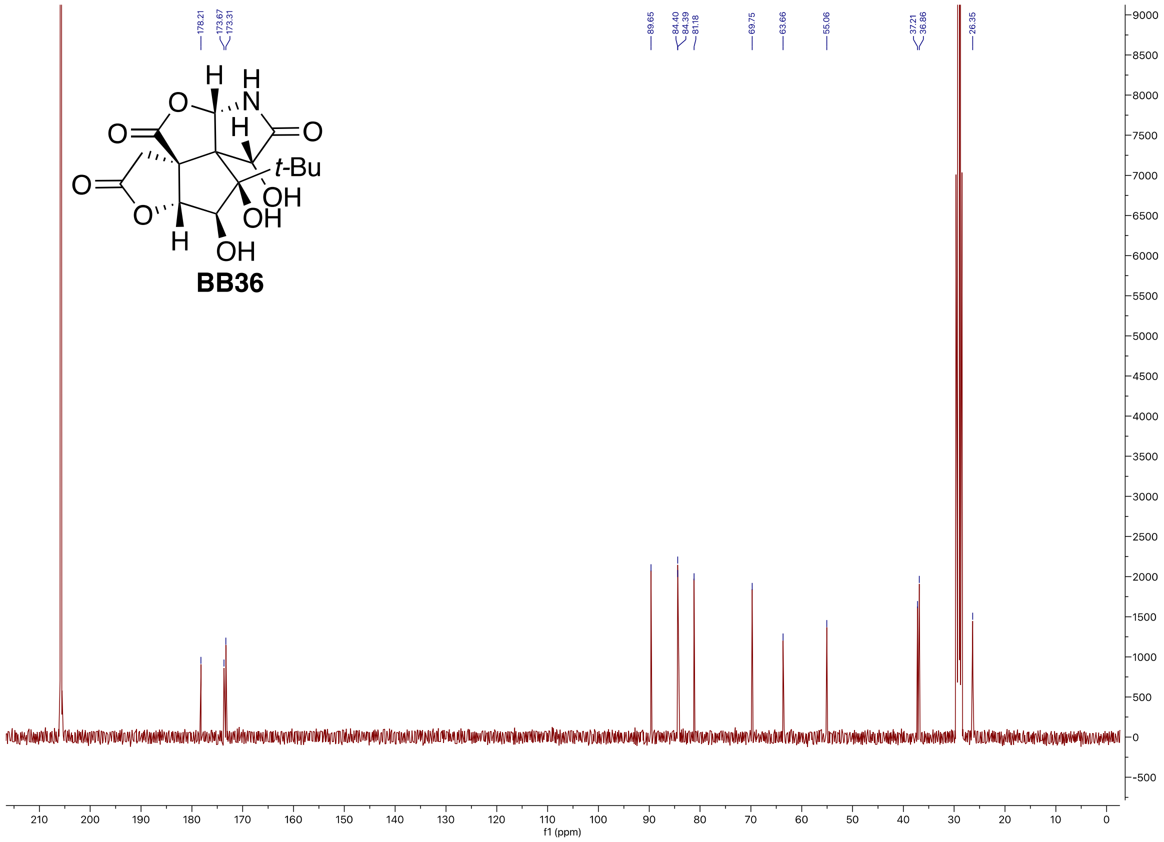

2.4 Method for the investigation of chemical stability

Bilobalide and **BB17** were dissolved in DMSO at 1 mg/mL respectively. 60 µL of DMSO solution was mixed with 30 mL PBS (pH=6.8 or 7.4) and shaken at 37 °C. At each time point, 0.5 mL of the mixture was transferred into test bottle and mixed with the same volume of methanol, subsequently measured on Agilent LC-MS/MS system consisting of two Agilent 1290 series pumps and auto-sampler, coupled with 6430 triple quadrupole mass spectrometer equipped with and ESI source (Agilent Technologies, Inc., Santa Clara, CA, USA). Sample injection volume was 10 µL. LC was performed by a Waters ACQUITY UPLC BEH C18 analytical column (2.1x50 mm, 1.7 µm) kept at ambient, using 0.1% formic acid (FA) in water (A) and 0.1% FA in acetonitrile (B) as mobile phases. The gradient elution (at 0.2 mL/min) started at an equilibration of 10% B for 1 min and increased proportions of B from 10% to 70% from 1 min to 2 min. Then the proportion of B decreased to 10% from 2 min to 5 min, and an additional 1 min was used to wash and re-equilibrate the column at 10%. Positive electrospray ionization (ESI) in multiple reaction monitoring (MRM) mode was used for MS operation. Precursor-to-product ion transitions at m/z 327.1 to 309.1 for bilobalide, and 326.1 to 308.1 for **BB17**.

2.5 HPLC analysis method and spectrum of BB10 and BB21

The purity of **BB10** and **BB21** was examined by Agilent 1260 HPLC (Santa Clara, USA) with Agilent 5 HC-C18 (2) 150 x 4.6 mm column kept at ambient temperature, using 0.1% formic acid (FA) in water (A) and 0.1% FA in acetonitrile (B) as mobile phases. The gradient elution (at 1 mL/min) started at an equilibration of 30% (**BB10**) or 50% (**BB21**) B and increased proportions of B to 90% (**BB10**) or 95% (**BB21**) from 1 min to 15 min, and an additional 5 min was used to wash the column at 95%.

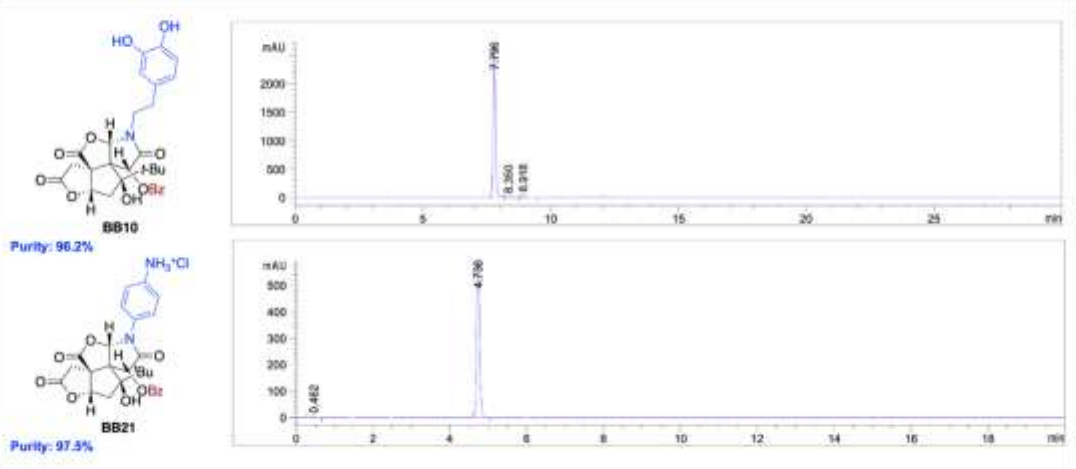

Entry #	ReTime [min]	Width [min]	Area [mAU*s]	Height [mAU]	Area %
1	7.796	0.1010	16801.3	2612.96362	96.1957
2	8.350	0.1839	306.10468	22.61995	1.7526
3	8.918	0.1177	385.33804	42.78968	2.0517

Entry #	ReTime [min]	Width [min]	Area [mAU*s]	Height [mAU]	Area %
1	0.462	0.1239	38.45996	4.40381	1.2356
2	4.736	0.0851	3074.10815	568.76184	98.7644

3. Biology part

3.1 General methods

3.1.1 Cell culture

HMC3 cell line was cultured in Minimum Essential Medium (MEM) (Gibco) supplemented with 1x non-essential amino acid (NEAA, Gibco), 1 mM sodium pyruvate (Gibco), 10% fetal bovine serum (FBS) (Gibco) and 100 U ml^{-1} penicillin/streptomycin (Gibco). BV-2 cell line was maintained in Roswell Park Memorial Institute (RPMI) 1640 medium (Gibco) supplemented with 10% FBS and 100 U ml^{-1} penicillin/streptomycin, and HT22 cell line was maintained in Dulbecco's Modified Eagle's Medium (DMEM) (Gibco) supplemented with 10% FBS and 100 U ml^{-1} penicillin/streptomycin. All cells were incubated at 37 °C in a humidified atmosphere with 5% CO_2. HMC3 and BV-2 cell lines were purchased from iCell Bioscience; HT22 cell line was a gift from Prof. CHOW Hei Man Kim.

3.1.2 Cell viability assay

For phenotypic screening of bilobalide analogues, 5,000 cells per well (100 µL volume) of HT22, HMC3 or BV-2 cells were placed in 96-well plate and allowed to adhere overnight. Afterwards, cells were pre-treated with 200 nM (on HT22, HMC3) or 500 nM (on BV-2) of RSL3 (Bidepharm) for 2 h respectively. Medium was then replaced by test compounds and followed by 22 h incubation. For cytotoxicity assay, HMC3 cells (seeded on 96-well plates at 5,000 cells per well overnight) were treated with multiple concentrations of **BB10**, **BB21** or bilobalide for 24 h. For evaluation of BB10 against ferroptosis inducers, HMC3 cells were pre-treated with multiple concentrations of FIN56, ML162 or ML210 for 2 h and followed by treatment with or without **BB10** (10 μM) for 22 h; or co-treated with multiple concentrations of erastin with or without **BB10** (10 μM) for 22 h. Cell viability was measured by Cell Counting Kit-8 (CCK-8).

3.1.3 Lipid peroxidation assay

For flow cytometry, HMC3 cells (seeded 50,000 cells per well on 24-well plate overnight) were pre-treated with 50 nM RSL3 for 2 h. After RSL3 treatment, medium was replaced by DMSO (0.1%) only or **BB10** (10, 5 and 2.5 µM) following by 3 h treatment. After which medium was replaced with BODIPY™ 581/591 C11 (10 µM, Invitrogen) for 30 min staining. Cells were then washed 3 times with PBS and collected. Fluorescence was measured by BD FACSymphony A5.2 SORP Flow Cell Analyzer (BD Biosciences).

3.1.4 Western blot

HMC3 (seeded 100,000 cells per well on 12-well plate overnight) was co-treated with RSL3 (1 μM) with or without **BB10** (10, 5, 2.5 μM) for 3 h. After incubation, cells were washed 3 times with ice-cold PBS and lysed in RIPA buffer containing 1x protease inhibitor cocktail (MCE) and nuclease (Beyotime) on ice for 30 min. All samples were centrifuged and then quantified by bicinchoninic acid (BCA) assay (Pierce). Cell lysates were diluted with Laemmli Sample Buffer (Bio-Rad) and heated at 95 °C for 5 min. Samples were separated by 12% SDS-PAGE (Vazyme) and transferred to a polyvinylidene difluoride membrane (Thermo Fisher). Membrane was incubated with 3% bovine serum albumin (BSA, Sigma-Aldrich) for 1 h, and subsequently incubated with indicated primary antibody overnight at 4 °C. After incubation, membrane was washed by wash buffer (containing 20 mM Tris, 150 mM NaCl and 0.1% Tween 20), and incubated with secondary antibody at room temperature for 1 h. Antibodies for GPX4 (52455, Cell Signaling Technology) was used at 1:500 dilution, GAPDH (sc-32233, Santacruz Biotechnology) was used at 1:3000 dilution, LC3B (A19665, ABclonal Technology) was used at 1:1000 dilution. Goat anti-rabbit IgG HRP-linked antibody (7074, Cell Signaling Technology) was used at 1:2500 dilution, goat anti-mouse IgG (H+L) secondary antibody DyLight™ 488 (35502, Invitrogen) was used at 1:5000 dilution. Blot was washed and visualized using Clarity Western ECL Substrate (Bio-Rad) with ChemiDoc MP Imaging System (Bio-Rad). Band intensity was normalized by image J software.

3.1.5 Antagonistic activity on $GABA_A$ receptor

Fluorescent measurements-based approach, $FLIPR^{TETRA}$ for α1β2γ2 $GABA_A$ receptor antagonist evaluation was performed by Charles River Laboratories International. Briefly, antagonistic effects of compounds were measured using FLIPR membrane potential red dye kit (Molecular Devices) according to the manufacturer's instructions on $GABA_A$ α1β2γ2 ionotropic receptor (human GABRA1, GABRB2, and GABRG2 genes) expressed in HEK293 cells. For dye loading, growth media was removed and replaced with 20 μL of membrane potential dye in HB-PS for 30 min at 37 ºC, then dye-loading solution was removed and replaced with 20 μL of low Cl^--HB-PS of the following composition (in mM): 137 Na-gluconate, 4 K-gluconate, 1 $MgCl_2$; 1.8 Ca-gluconate, 10 HEPES, 10 Glucose; pH adjusted to 7.4 with NaOH. The antagonist effects of the test articles will be evaluated after application of GABA 5.2 μM for stimulation $GABA_A$ receptors in low Cl^--HB-PS of the following composition (in mM): 137 Na-gluconate, 4 K-gluconate, 1 $MgCl_2$; 1.8 Ca-gluconate, 10 HEPES, 10 Glucose; pH value was adjusted to 7.4 with 1 N NaOH.

3.1.6 Iron chelation activity evaluation

BB10 or deferiprone (DFP) (Bidepharm) were dissolved in DMSO, and then diluted to indicated concentration by 5 µM $FeCl_2$ solution, mixture was incubated at room temperature for 2 min. 50 µL 5 mM ferrozine solution was then mixed to the solution, and then the absorbance at 562 nm was measured by CLARIOstar multimode plate reader.

3.1.7 Radical scavenger activity evaluation

Radical scavenger activity evaluation was carried out by ABTS [2,2'-azinobis-(3-ethylbenzothiazoline-6-sulfonate)] assay as reported.[1] Briefly, 10 mM ABTS was mixed with 2.45 mM ammonium persulfate, the mixture was incubated for 16 h at room temperature to form $ABTS^{\bullet+}$ radical. $ABTS^{\bullet+}$ was then diluted by ethanol before testing. 1 µL test compounds (dissolved in DMSO) was mixed with 99 µL $ABTS^{\bullet+}$ and the mixture was incubated in the dark for 30 min, the absorbance was measured on a CLARIOstar plate reader at 734 nm.

3.2 Supplementary figures

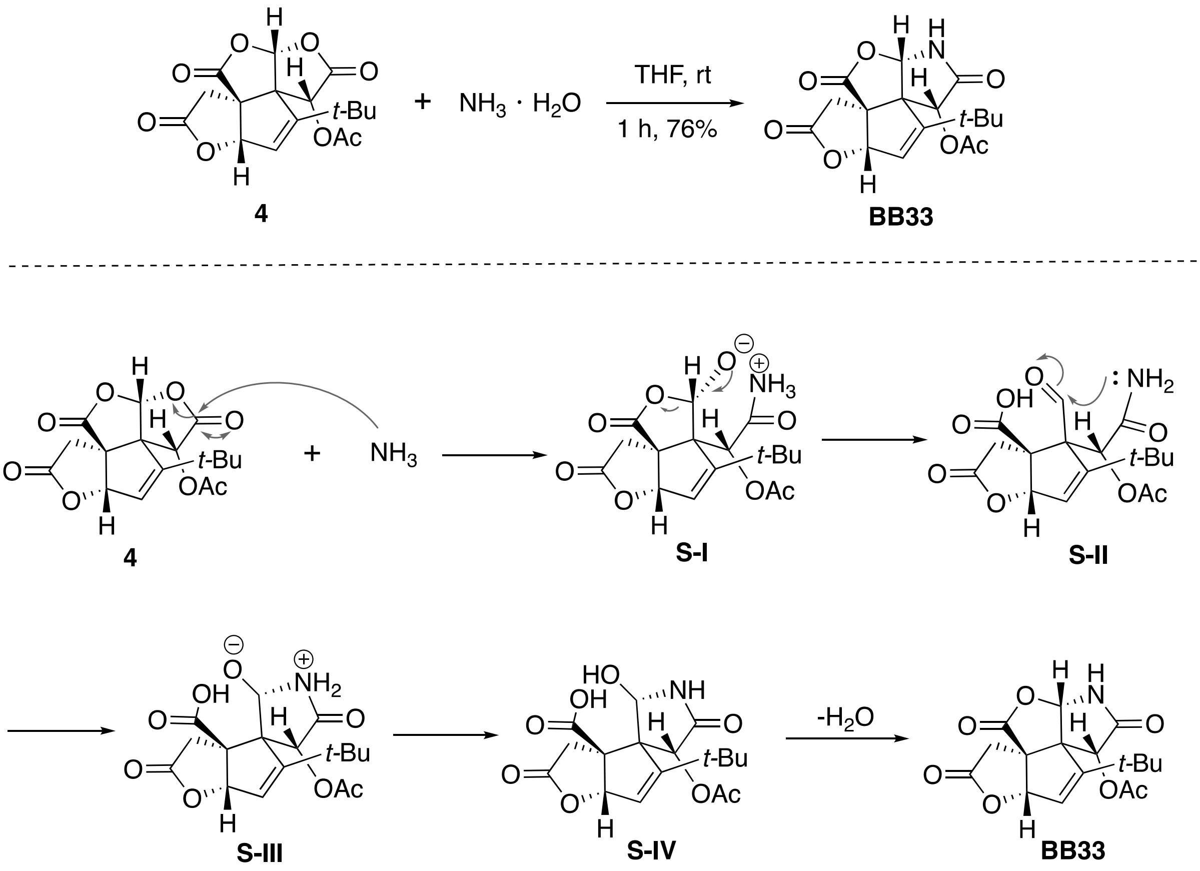

Figure S8 Proposed reaction mechanism of the transformation of the intermediate **4** to **BB33**.

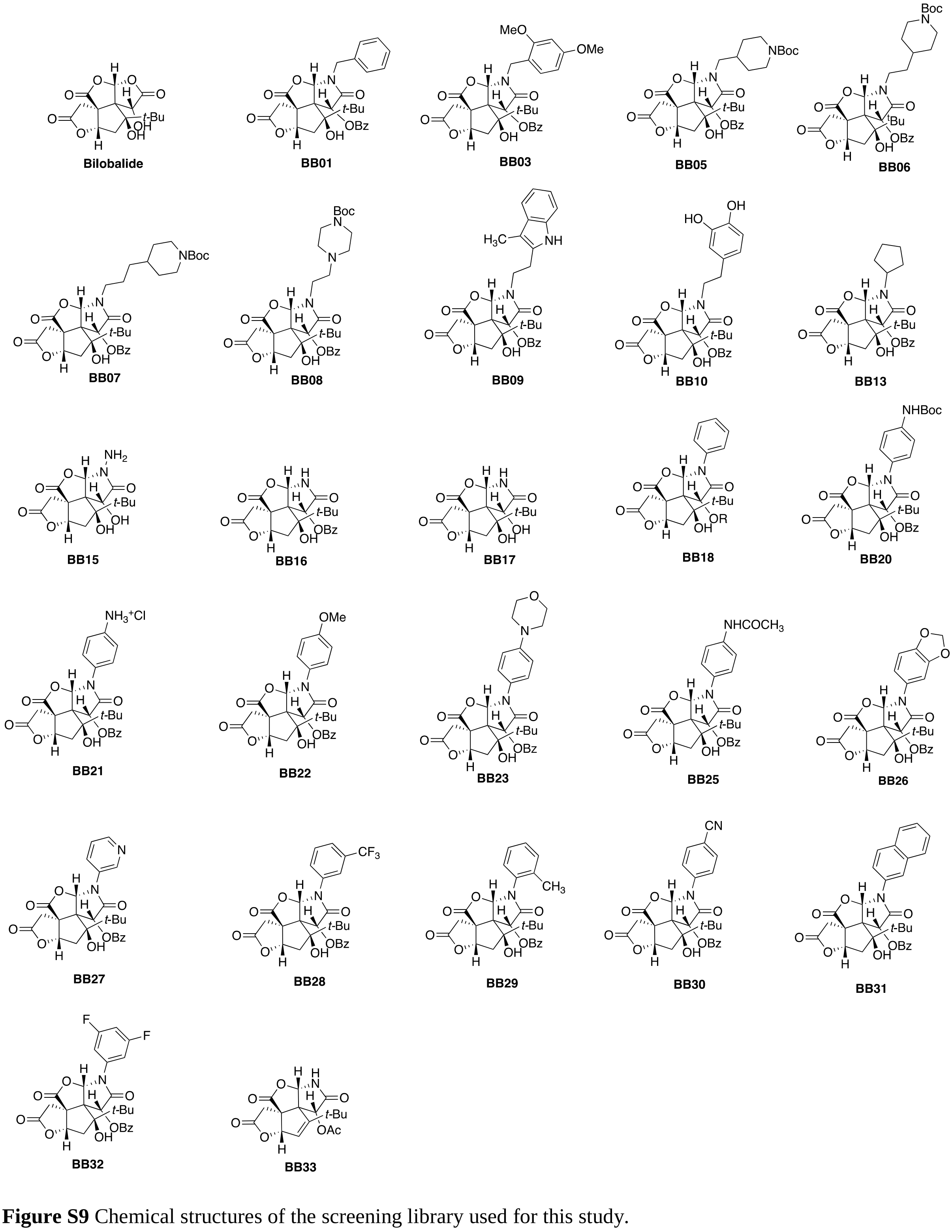

Figure S9 Chemical structures of the screening library used for this study.

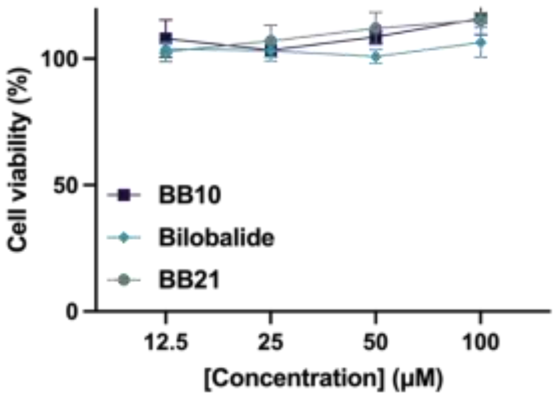

Figure S10 Bilobalide and analogues demonstrated no cytotoxicity. HMC3 cells were treated with multiple concentration of bilobalide, **BB10** or **BB21** for 24 h. Data are plotted as mean ± s.d., n = 3 technical replicates.

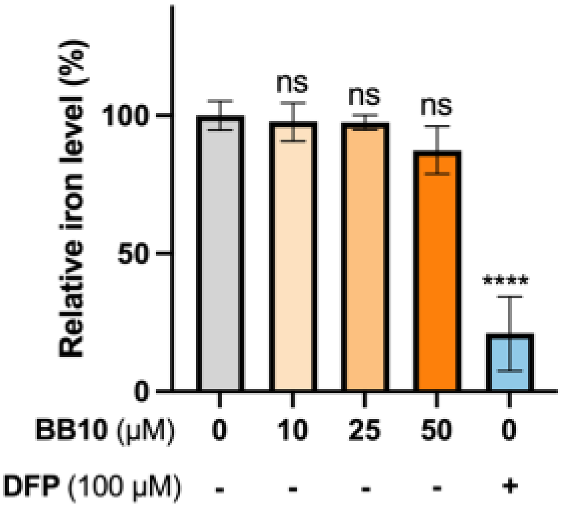

Figure S11 BB10 lacks iron chelator activity. Deferiprone (DFP) is the positive control of this assay. Data are plotted as mean ± s.d., n = 3 technical replicates. Statistical analyses were performed by one-way ANOVA with multiple comparisons; ns, no significance; ****p <0.0001 versus control group.

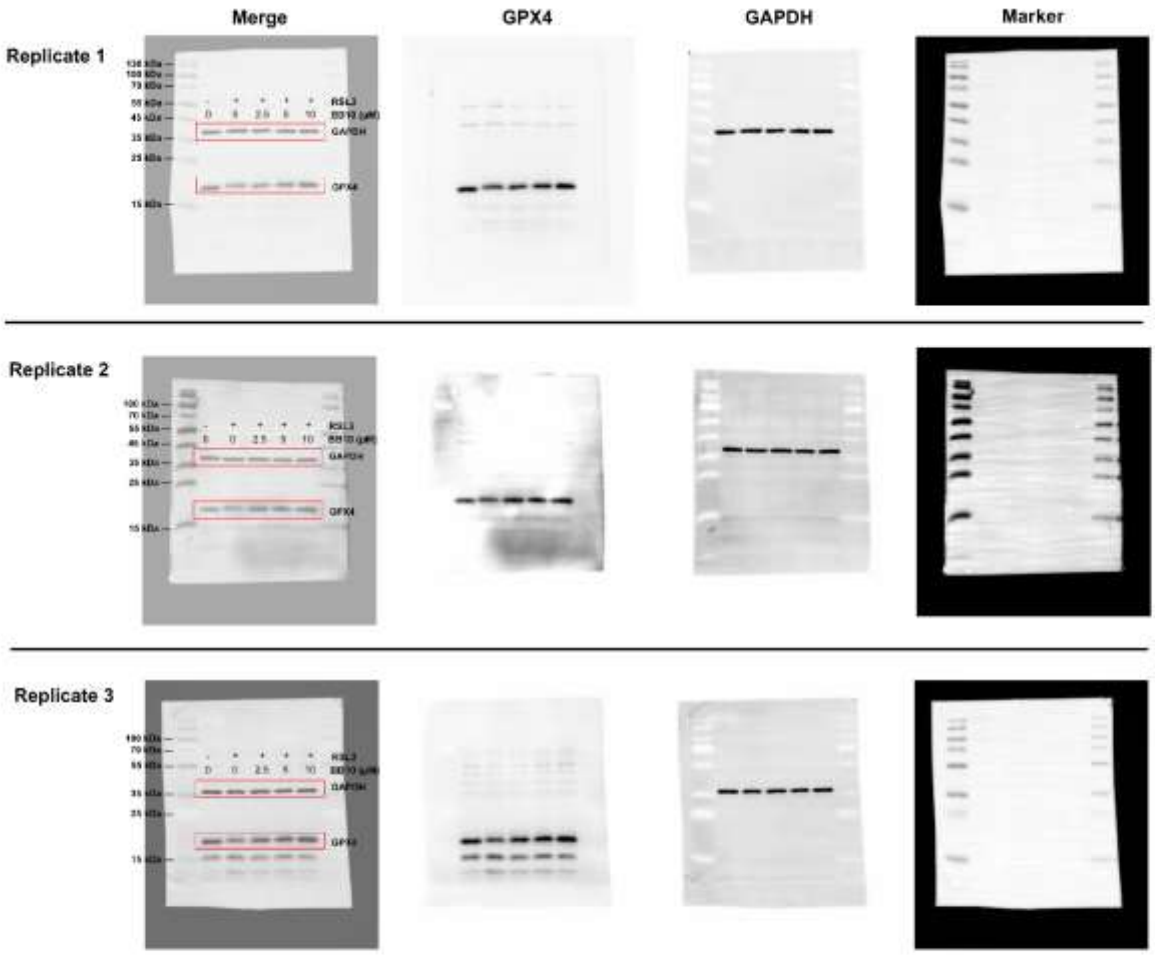

Figure S12 Unprocessed Western blots of 3 biological independent assays in main Figure 6b-c.

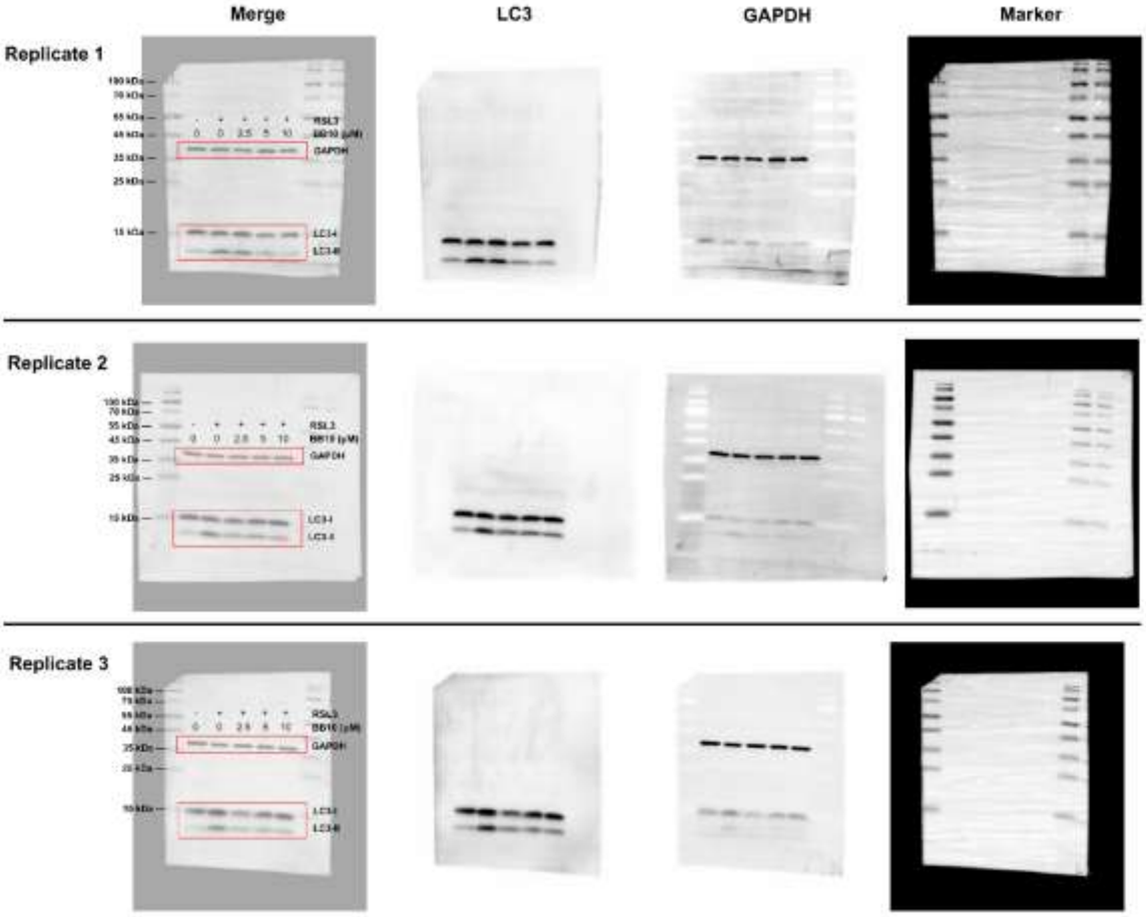

Figure S13 Unprocessed Western blots of 3 biological independent assays in main Figure 6d-e.

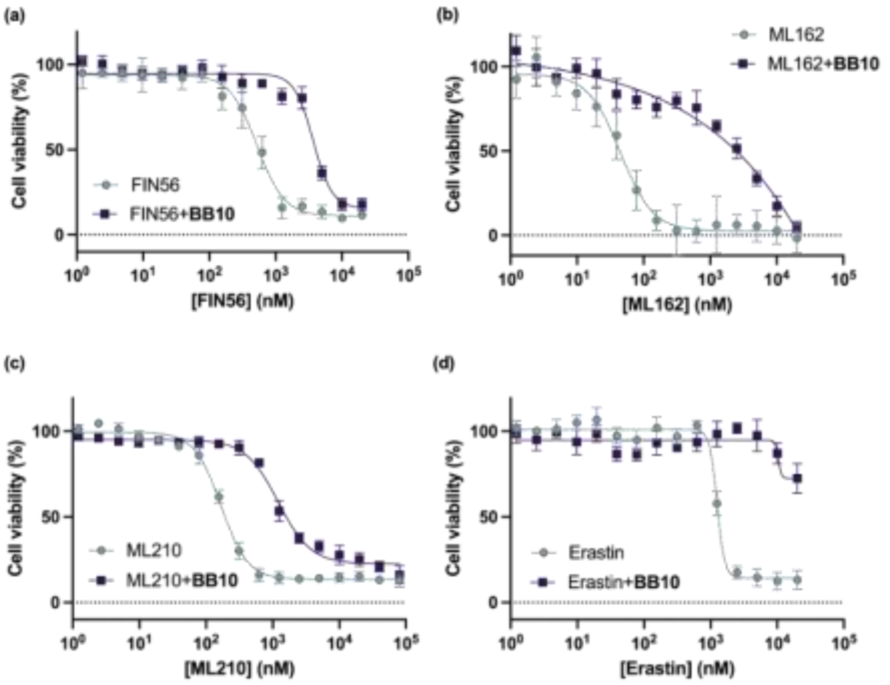

Figure S14 Attenuation of the lethality of ferroptosis inducers by **BB10**. HMC3 cells were pretreated with ferroptosis inducers: FIN56 (a), ML162 (b), and ML210 (c), followed by co-treatment with either **BB10** (10 μM) for 22 hours. For erastin (d), cells were co-treated with ferroptosis inducers alongside **BB10**. **BB10** notably inhibited ferroptosis, as demonstrated by a more pronounced rightward shift in the dose-response curve. Data are plotted as mean ± s.d., n = 3 technical replicates.

Reference

(1) Re, R.; Pellegrini, N.; Proteggente, A.; Pannala, A.; Yang, M.; Rice-Evans, C. Antioxidant Activity Applying an Improved ABTS Radical Cation Decolorization Assay. Free Radic. Biol. Med. 1999, 26 (9–10), 1231–1237. https://doi.org/10.1016/S0891-5849(98)00315-3.
